# Genetic diversity and population structure of ridge gourd (*Luffa acutangula*) accessions in a Thailand collection using SNP markers

**DOI:** 10.1038/s41598-021-94802-4

**Published:** 2021-07-28

**Authors:** Grimar Abdiel Perez, Pumipat Tongyoo, Julapark Chunwongse, Hans de Jong, Anucha Wongpraneekul, Waraporn Sinsathapornpong, Paweena Chuenwarin

**Affiliations:** 1 Program of Agricultural Sciences, Faculty of Agriculture at Kamphaeng Saen, Kasetsart University, Kamphaeng Saen Campus, Nakhon Pathom, Thailand; 2 Center for Agricultural Biotechnology, Kasetsart University, Kamphaeng Saen Campus, Nakhon Pathom, Thailand; 3 Center of Excellence on Agricultural Biotechnology: (AG-BIO/MHESI), Bangkok, Thailand; 4 Department of Horticulture, Faculty of Agriculture at Kamphaeng Saen, Kasetsart University, Kamphaeng Saen Campus, Nakhon Pathom, Thailand; 5 Laboratory of Genetics, Wageningen University and Research (WUR), Wageningen, The Netherlands; 6 Tropical Vegetable Research Center (TVRC), Department of Horticulture, Faculty of Agriculture at Kamphaeng Saen, Kasetsart University, Kamphaeng Saen Campus, Nakhon Pathom, Thailand

**Keywords:** Biotechnology, Computational biology and bioinformatics, Genetics, Plant sciences

## Abstract

This study explored a germplasm collection consisting of 112 *Luffa acutangula* (ridge gourd) accessions, mainly from Thailand. A total of 2834 SNPs were used to establish population structure and underlying genetic diversity while exploring the fruit characteristics together with genetic information which would help in the selection of parental lines for a breeding program. The study found that the average polymorphism information content value of 0.288 which indicates a moderate genetic diversity for this *L. acutangula* germplasm. STRUCTURE analysis (*ΔK* at *K* = 6) allowed us to group the accessions into six subpopulations that corresponded well with the unrooted phylogenetic tree and principal coordinate analyses. When plotted, the STRUCTURE bars to the area of collection, we observed an admixed genotype from surrounding accessions and a geneflow confirmed by the value of *F*_ST_ = 0.137. AMOVA based on STRUCTURE clustering showed a low 12.83% variation between subpopulations that correspond well with the negative inbreeding coefficient value (*F*_IS_ =  − 0.092) and low total fixation index (*F*_IT_ = 0.057). There were distinguishing fruit shapes and length characteristics in specific accessions for each subpopulation. The genetic diversity and different fruit shapes in the *L. acutangula* germplasm could benefit the ridge gourd breeding programs to meet the demands and needs of consumers, farmers, and vegetable exporters such as increasing the yield of fruit by the fruit width but not by the fruit length to solve the problem of fruit breakage during exportation.

## Introduction

*Luffa acutangula*, commonly known as ridge gourd, angled loofah, or Chinese okra, is a domesticated vegetable of the Cucurbitaceae originating from India^1–4^. Immature fruits use as a vegetable, which can be cooked or fried. In South-East Asia, sweet juiciness and soft texture are preferred characteristics^2^. Its use in traditional medicine is especially prevalent in Asia, middle America^5,6^, and India to treat jaundice and urinary bladder stones^7,8^. Ridge gourd production is of great importance to smallholder farmers and exporters in Asia. People in specific areas demand different ridge gourd fruit types. Nowadays, commercial ridge gourds have very long fruit that causes the fruit to break during packing. As the demand for ridge gourds in Asia^9^ steadily increases, the vegetable will be put through further selection by breeding programs leading to decreased genetic diversity, as documented in many crop species like durum wheat^10^. To prevent the loss of diversity, creating a germplasm that contains natural genetic resources will be of paramount importance in future breeding initiatives for introgressing biotic or abiotic stress tolerance or resistance factors into elite cultivars.

Most of the genetic diversity in germplasms have been established by describing the morphological variation of the accessions^11^. Still, such a limited approach is subject to environmental as well as developmental conditions of the plant^12^. Instead, molecular markers provide stabler sources of information for carrying out genetic diversity studies and are more suitable for establishing genetic diversity in and between populations. Identifying population structure is essential for understanding the genetic diversity, evolutionary forces, and geographic distribution of the germplasm^13^. This information would be of great help to plant breeders in selecting potential gene pools in breeding programs^14^.

Various marker techniques have been developed for acquiring accurate and reliable information on population structure and genetic diversity of germplasms^15^. Molecular markers used for population structure and genetic diversity studies in ridge gourd include Inter Simple Sequence Repeat (ISSR)^16^, Random Amplified Polymorphism Detection (RAPD)^17^, Simple Sequence Repeats (SSRs)^18^, and Directed Amplification of Minisatellite DNA (DAMD)^19^. These techniques can be labor-intensive, costly and produce a low number of markers. Newer genotyping by sequencing (GBS) methods can cut down genome complexity and provide important genotype information. An example is the DArTseq method that can select the genome fractions corresponding to active genes^20–23^.

The present study aims to identify population structure, genetic diversity, and association of fruit traits with subpopulations of a *L. acutangula* germplasm in Thailand using DArTseq based SNPs. The study will provide essential information for *Luffa* improvement programs for breeders worldwide for future on-farm problems.

## Results

### Population structure analysis

**Figure 1:**
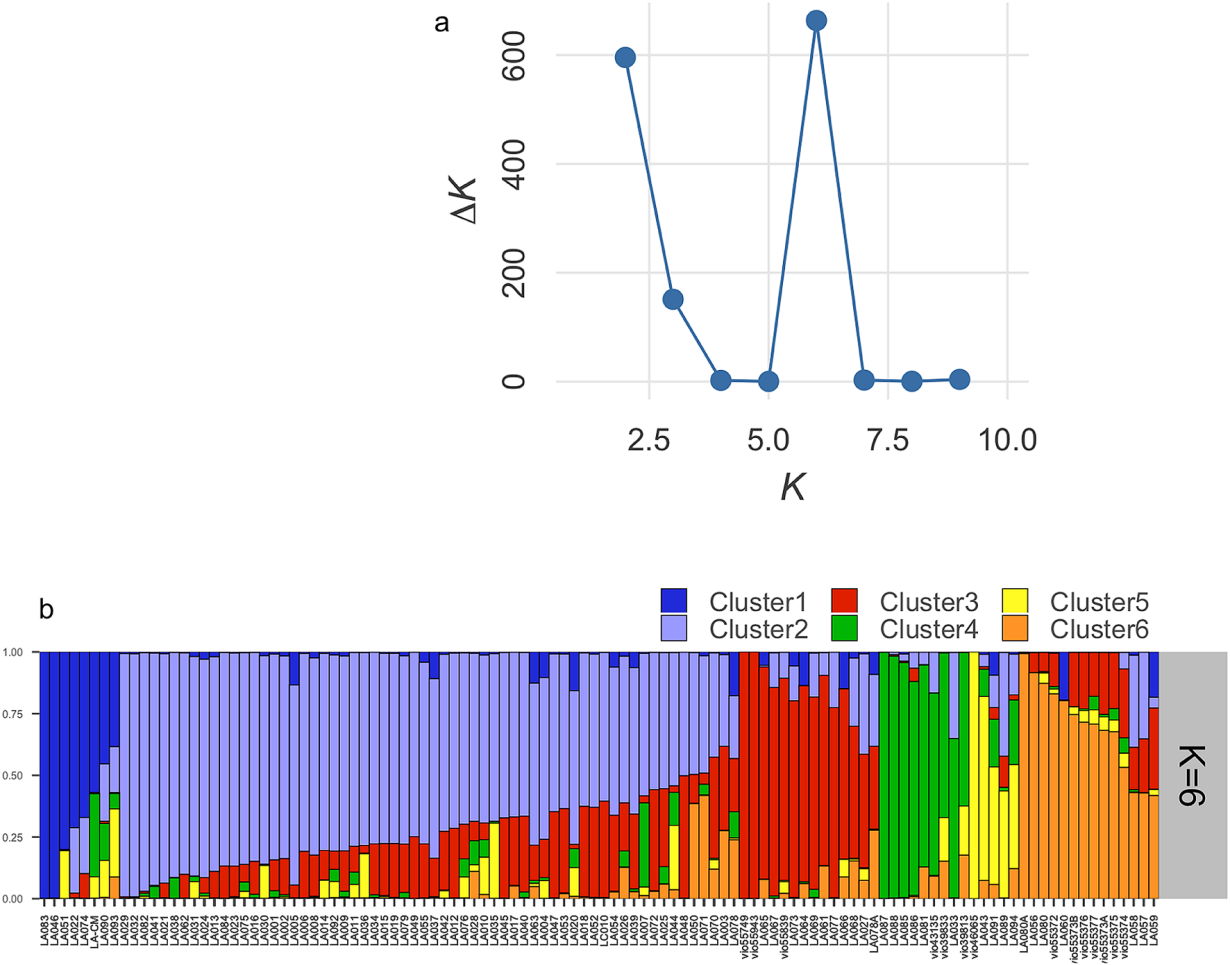
Population structure of 112 *Luffa acutangula* accessions using DArTseq-based SNP markers inferred by STRUCTURE program. (**a**) Number of subpopulations indicated by the highest *ΔK*; (**b**) Proportion of clustering of individuals to six subpopulations.

**Figure 2:**
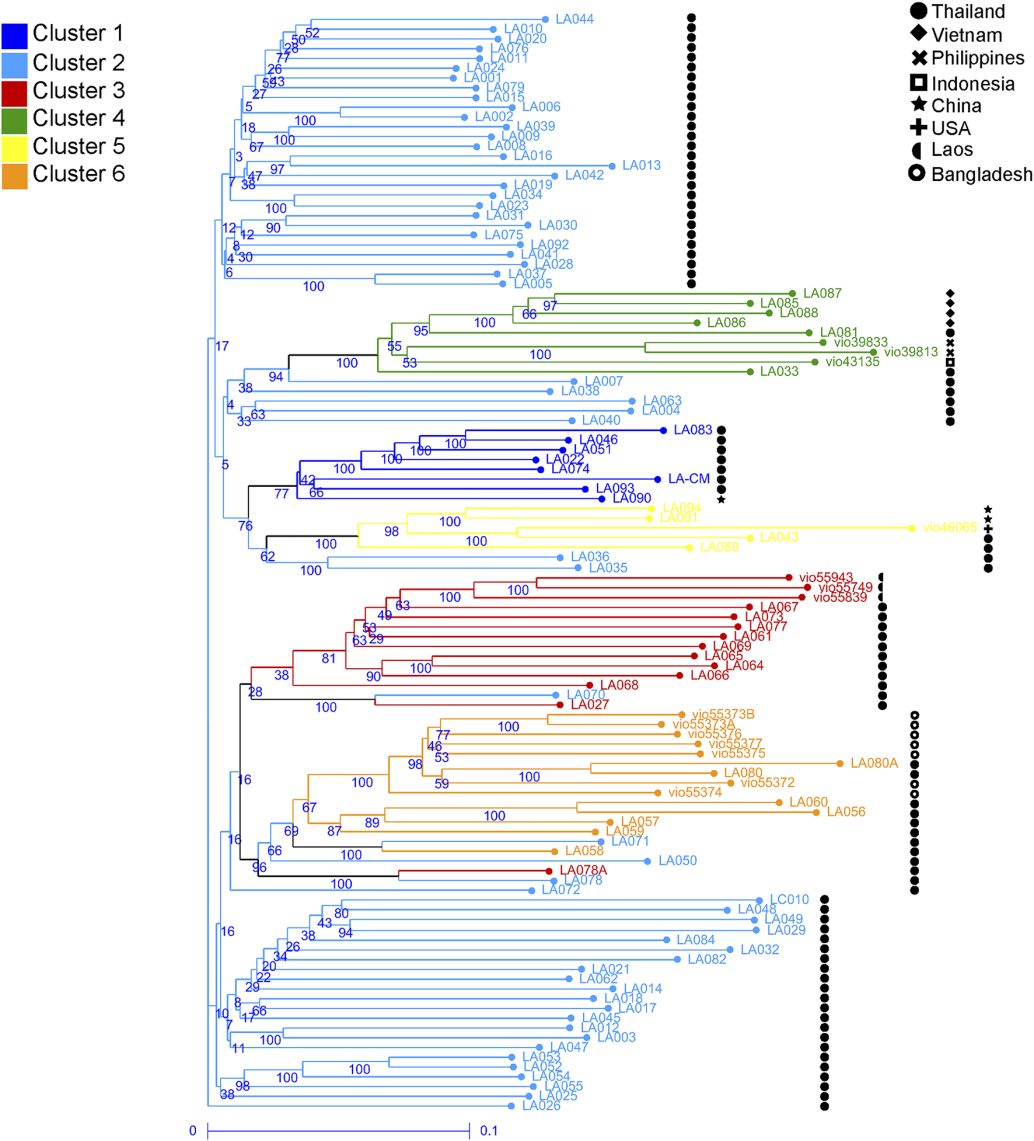
Weighted neighbor-joining dendrogram of 112 *Luffa acutangula* accessions based on DArTseq SNPs. Branch colors correspond to clustering by STRUCTURE analysis. Symbols indicate accession’s country of origin.

*L. acutangula* has an estimated genome size of 760 Mb, spread over 13 chromosomes. It has approximately 42,211 predicted gene models, of which 32,233 are protein-coding genes with an average gene size of 2886 nt^22^. The 112 *L. acutangula* accessions of this study originated from two genetic resources. Based on the analysis of 2834 SNPs, we observed significant intra-chromosomal SNP pairs (*p* < 0.05) with an average r^2^ of 0.217 and a mean distance of 11,824,426 nt. STRUCTURE software used a model-based Bayesian algorithm to infer population structure (*K* > 1). The actual number of clusters (*K*) was determined by the ad hoc statistic ∆*K* based on the log probability of data with respect to *K* values^23,24^. STRUCTURE analysis of the 2834 SNPs reveals the highest value of *ΔK* at *K* = 6 (Fig. 1). This value indicates a total of six informative subpopulations found across all *L. acutangula* accessions. Each subpopulation written as Cluster 1–6, represents 7%, 55%, 13%, 8%, 4% and 13% of the total number of accessions, respectively. Cluster 1 consists of seven accessions from Thailand and one from China (Fig. 2). All 62 accessions in Cluster 2 are from Thailand. Cluster 3 consists of eleven accessions from Thailand and three from Laos. Cluster 4 comprises two, four, two and one accessions from Thailand, Vietnam, the Philippines and Indonesia, respectively. Cluster 5 includes two accessions from Thailand, two from China and one from the USA. Cluster 6 comprises seven accessions from Thailand and seven from Bangladesh.

**Figure 3:**
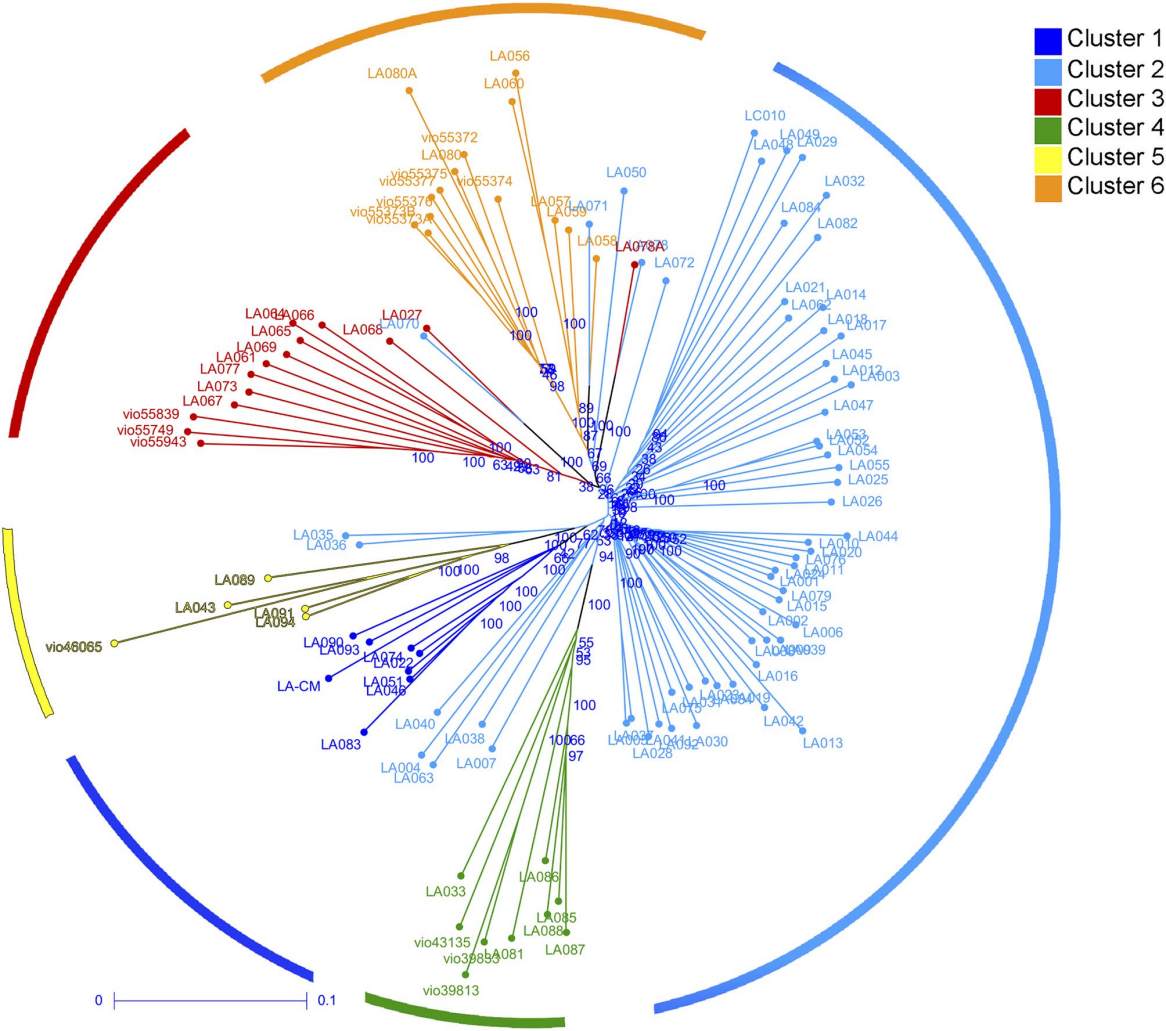
Phylogenetic tree among the 112 *Luffa acutangula* accessions. The colors of branches illustrate accessions belonging to different clusters acquired from STRUCTURE analysis. Six clades were identified as indicated by circle colors.

**Figure 4:**
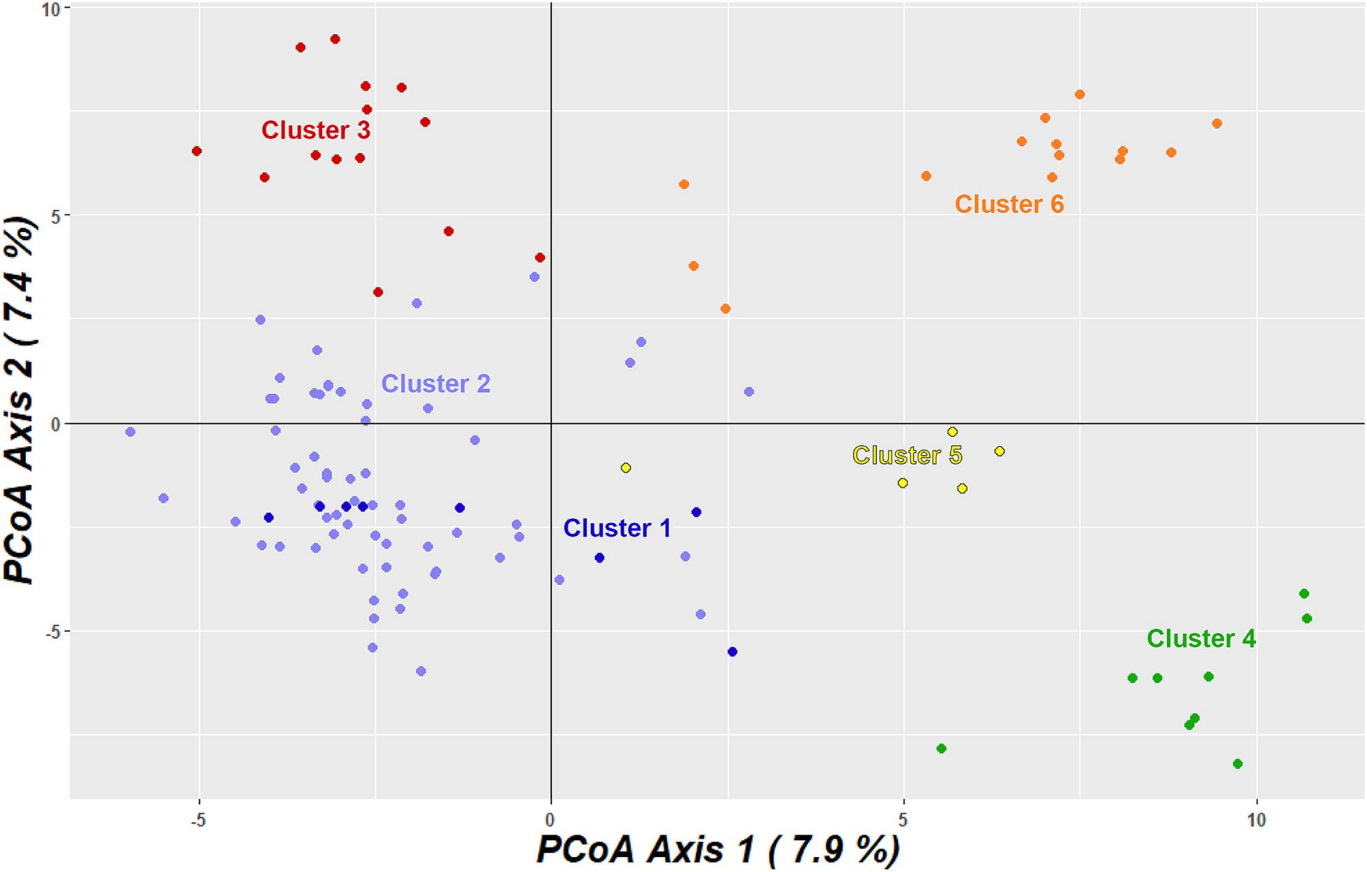
Principal coordinate analysis (PCoA) of 112 *Luffa acutangula* accessions using DArTseq-based SNPs.

The unrooted phylogenetic tree differentiates the 112 *L. acutangula* accessions into six clades consistent with the *ΔK* at *K* = 6 (Fig. 3). At the same time, Principal Coordinate Analysis (PCoA) can only distinguish five groups in which the accessions of cluster 1 overlapped with those of cluster 2 (Fig. 4). Axes 1 and 2 of PCoA explains 15.3% of the total variance. Cluster 1 and 2 have some accessions falling in two or three quadrants, suggesting a wide diversity in these two clusters, made of Thailand's accessions.

### Genetic diversity and geographic distribution

Heterozygosity can be measured by expected heterozygosity (*H*_*E*_) and observed heterozygosity (*H*_*O*_). *H*_*E*_ gives information about the probability of an individual’s portion of heterozygosity for all analyzed loci, while *H*_*O*_ is the portion of heterozygous genes in the analyzed population^25^. The *H*_*E*_ as measure of genetic diversity ranges from 0.338 (Cluster 2) to 0.272 (Cluster 3) in the six clusters; the remaining cluster values are the following: Cluster 1 (0.274), Cluster 4 (0.295), Cluster 5 (0.287) and cluster 6 (0.334) (Table 1). The high *H*_*E*_ in Cluster 2 also corresponds with the highest Shannon & Weiner diversity index (H) of 4.127. The *H*_*O*_ for the STRUCTURE grouping is as follows: Cluster 1 (0.378), Cluster 2 (0.388), Cluster 3 (0.191), Cluster 4 (0.289), Cluster 5 (0.363) and Cluster 6 (0.410) (Table 1). The *H*_*O*_ > *H*_*E*_ occurs in Cluster 1, Cluster 2, Cluster 5 and Cluster 6 (Table 1). The *H*_*O*_ in Cluster 4 (0.289) is lower in value than the *H*_*E*_ (0.295). Cluster 3 has the lowest *H*_*E*_ (0.272) and *H*_*O*_ (0.191) from all six subpopulations. The mean SNP polymorphism information content (PIC) has a range of values from 0.125 to 0.375, with a mean of 0.288 (Table 1). F-statistics is useful for inferring genetic diversity among and within populations. Moderate differentiation occurs among populations (*F*_ST_ = 0.137) and in the total fixation index (*F*_IT_ = 0.057), while a low fixation index occurs within populations (*F*_IS_ =  − 0.092) (Table 1).

**Table 1:** Genetic diversity among 112 *Luffa acutangula* accessions based on STRUCTURE analysis.

	Cluster 1	Cluster 2	Cluster 3	Cluster 4	Cluster 5	Cluster 6	Total
N	8	62	14	9	5	14	112
H	2.079	4.127	2.639	2.197	1.609	2.639	4.718
*H*_*O*_	0.378	0.388	0.191	0.289	0.363	0.410	0.357
*H*_*E*_	0.274	0.338	0.272	0.295	0.287	0.334	0.359
PIC		Min PIC = 0.125		Max PIC = 0.375		Mean PIC = 0.288	
*F*-statistics		*F*_ST_ = 0.137		*F*_IT_ = 0.057		*F*_IS_ = − 0.092	

*N* number of accessions, *H* Shannon & Weiner diversity index, *H*_*O*_ observed heterozygosity, *H*_*E*_ expected heterozygosity, *PIC* polymorphism information content, *F*_ST_ differentiation among populations, *F*_IT_ total fixation index, *F*_IS_ fixation index within populations.

Pairwise genetic differentiation is highest between Cluster 3 and Cluster 4 (*F*_ST_ = 0.262), while the lowest value occurs between Cluster 1 and Cluster 2 (*F*_ST_ = 0.089), and also between Cluster 2 and Cluster 3 (*F*_ST_ = 0.086) (Table 2). The Analysis of Molecular Variance (AMOVA) method approximates the population differentiation directly from the SNP data^26,27^. AMOVA based on STRUCTURE clustering shows 12.83% variation among subpopulations and a negative AMOVA variance within subpopulations (− 10.59%) (Table 3). Phi-statistics (ϕ) gives an overview of the level of differentiation between clusters. According to the phi-statistics, a moderate degree of differentiation occurs between STRUCTURE clusters (ϕ = 0.128)^28,29^.

**Table 2:** Pairwise *F*_ST_ (genetic differentiation) values among clusters identified by STRUCTURE analysis.

STRUCTURE clustering	Cluster 2	Cluster 1	Cluster 3	Cluster 4	Cluster 5
Cluster 1	0.089				
Cluster 3	0.086	0.180			
Cluster 4	0.164	0.230	0.262		
Cluster 5	0.147	0.203	0.202	0.210	
Cluster 6	0.123	0.186	0.127	0.193	0.174

**Table 3:** Analysis of molecular variance (AMOVA) of 112 *Luffa acutangula* accessions.

Grouping of accessions	Sample	Df	Mean Sq	Variance	%	Phi statistics (ϕ)	P-value
Structure clustering	Between populations	5	4312.218	123.098	12.83	0.128	0.001
	Between individuals within populations	106	734.459	− 101.542	− 10.59		
	Within individuals	112	937.543	937.543	97.75		
	Total	223	916.675	959.099	100.00		

*Df* degrees of freedom, *Sq* square.

**Figure 5:**
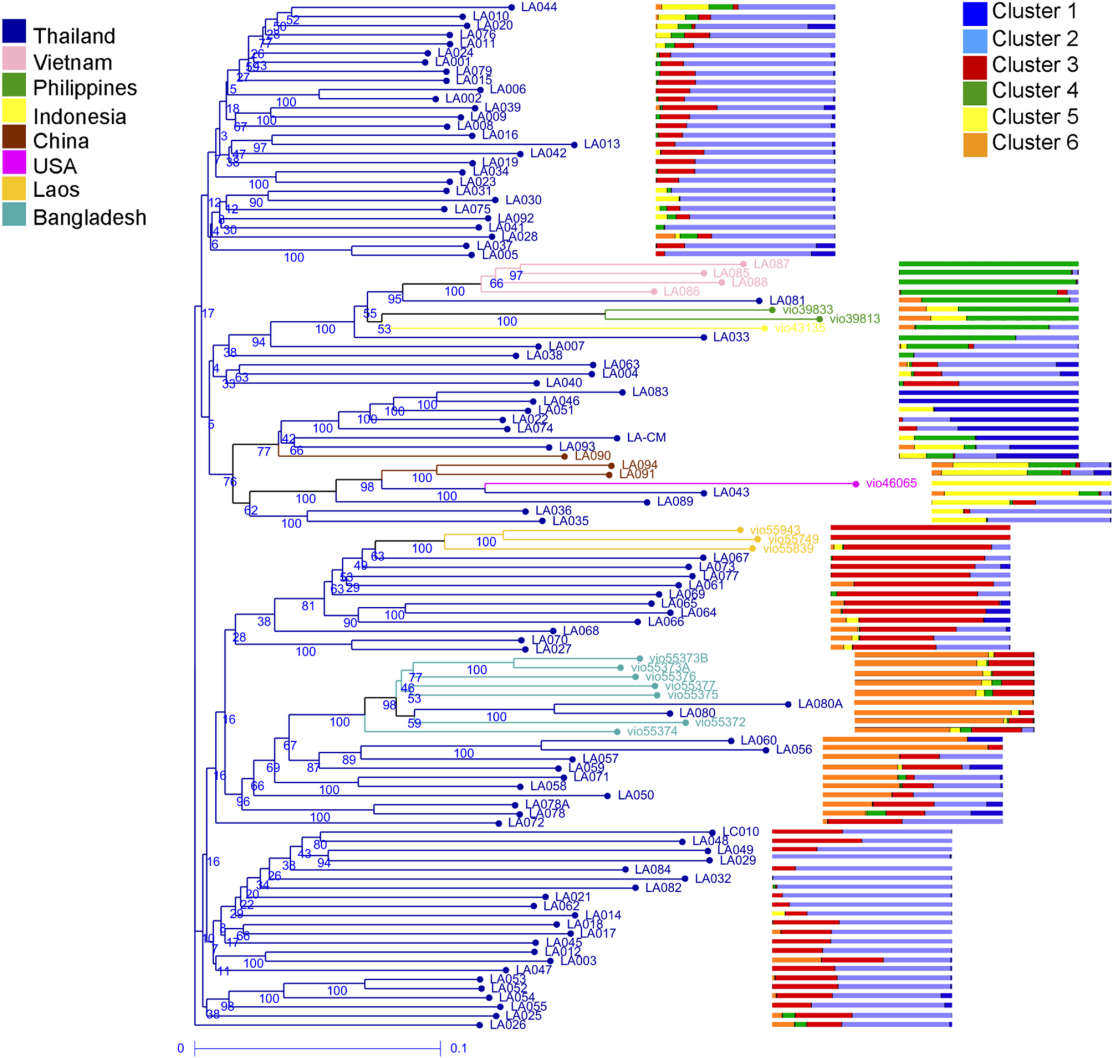
Genetic diversity proportion of 112 *Luffa acutangula* accessions based on country of origin. Branch colors indicate country of origin. Side bars correspond to proportion clustering from Fig. 1.

**Figure 6:**
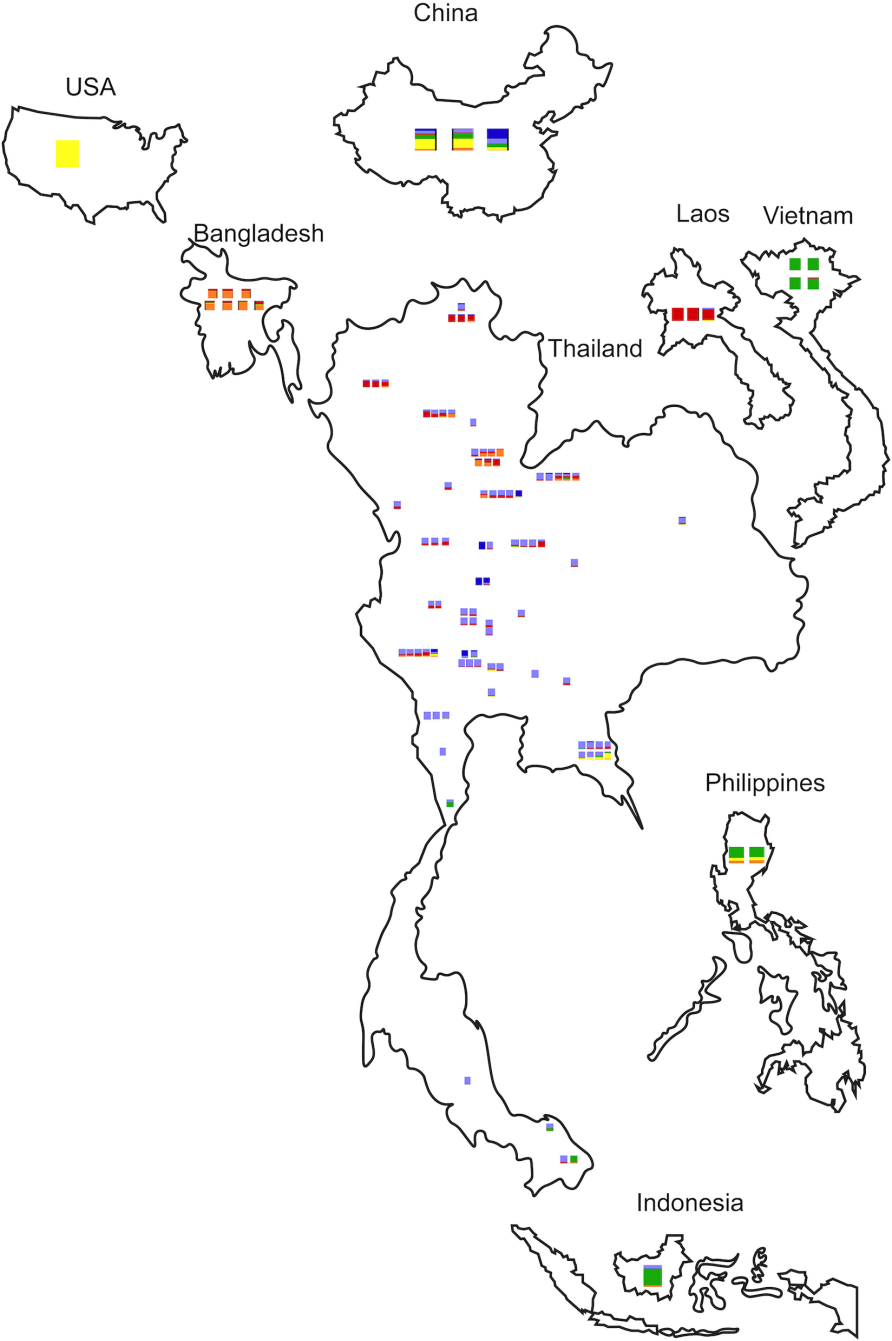
Distribution of *Luffa acutangula* accessions and their corresponding countries. Colors are based on STRUCTURE analysis bars from Fig. 1 (Maps created in Adobe Illustrator 2021 version 25.1).

Cluster 1 and 2 represent the genetic admixture found in Thailand, while Cluster 3 exhibits the genotype from Laos, as seen from the STRUCTURE analysis (Fig. 5). Accessions from Vietnam, the Philippines and Indonesia are closely related and have the main admixture proportion from Vietnam, Cluster 4. The genetic admixture for Cluster 5 is from the USA, China and Thailand, while Cluster 6 accessions are from either Bangladesh or Thailand. In this study, the *L. acutangula* accessions from Thailand shows the diversity that includes the admixture proportions of *L. acutangula* accessions from other countries (Fig. 6). However, the accessions from Laos and Vietnam are highly uniform and have low admixture proportions from accessions belonging to other countries. Both the Philippines and Indonesia have accessions with genetic admixture from Vietnam and Bangladesh. Still, they differ from that of the Philippines, which contains admixed genotypes from the USA, while accessions from Indonesia have from Thailand.

### Association of fruit trait and STRUCTURE clustering

**Figure 7:**
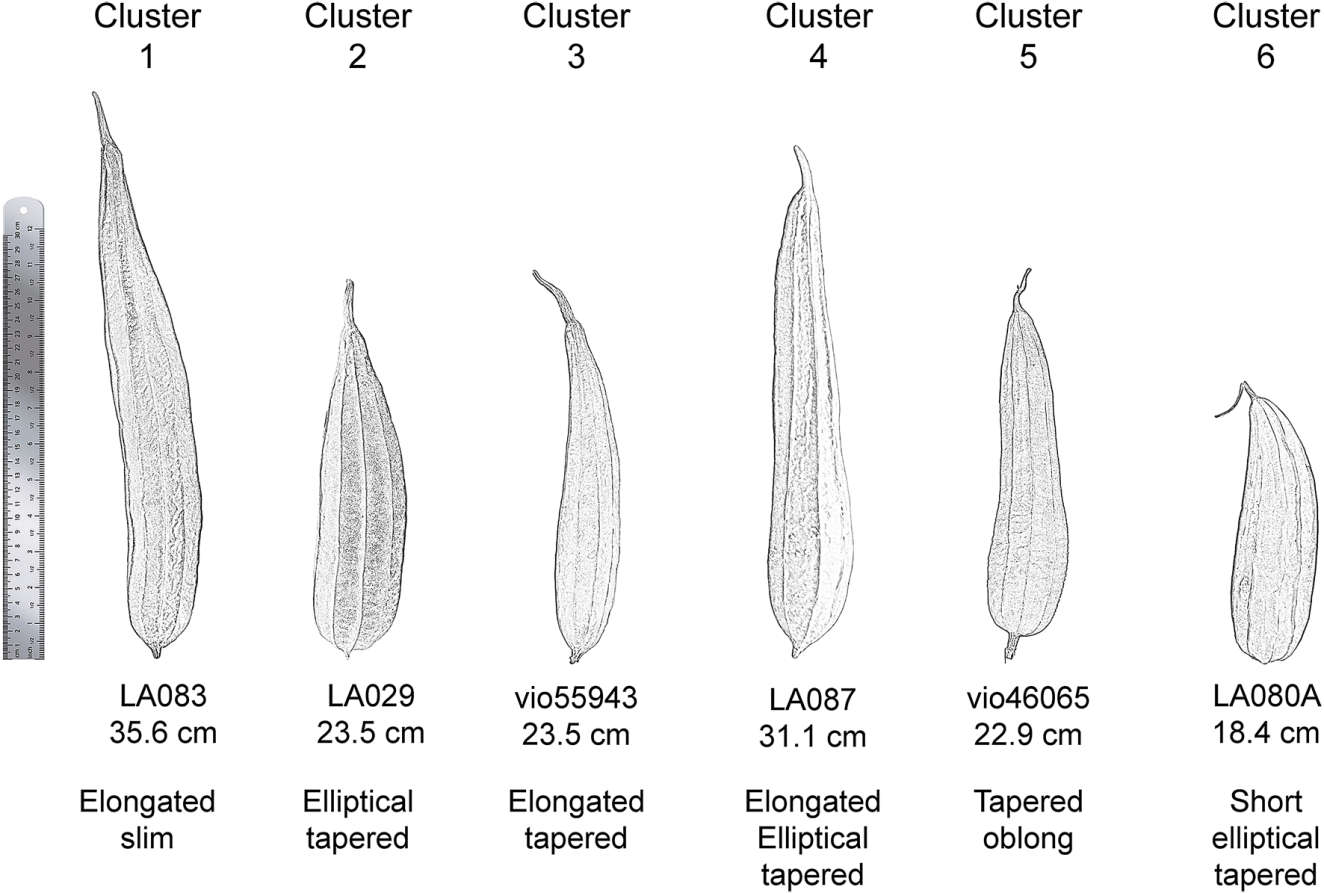
*Luffa acutangula* fruit shape. Each cluster shape is represented by an accession with the least or no admixture.

Correlations between fruit traits and subpopulations will help in selecting parental lines for breeding purposes, such as to solve the fruit breakage problem faced by vegetable exporters in Thailand. The 112 accessions of *L. acutangula* germplasm are diverse for fruit shape and length. Six different shapes represent each of the six subpopulations: elongated slim, elliptical tapered, elongated tapered, elongated elliptical tapered, tapered oblong, and short elliptical tapered (Fig. 7; Supplementary Table S2 and Supplementary Figure S1). Similar fruit shapes were observed on plants grown during a previous season (Supplementary Figure S2). One-way ANOVA test of fruit length shows a significant difference between STRUCTURE clusters (p-value = 5.1e−08) (Supplementary Table S3). Tukey HSD p-values after adjustment for the multiple comparisons for fruit length showed significant differences for Cluster 1 and Cluster 2, Cluster 1 and Cluster 3, Cluster 1 and 6, Cluster 2 and Cluster 6, and Cluster 4 and Cluster 6 (Supplementary Figure S4). Other cluster combinations do not show significant differences in fruit length, according to Tukey HSD.

Fruit shape for each subpopulation was defined using the least admixed individuals. Subpopulation 1 displays an elongated slim fruit shape and a long fruit type. The fruit shape of subpopulation 2 is elliptically tapered with a medium length, round blossom end and round stem end. Fruit shape of subpopulation 3 is elongated tapered, has medium to short length, pointed blossom end, and pointed stem end. The fruit shape of subpopulation 4 is elongated elliptical tapered with medium to long length with round blossom end and pointy stem end. Subpopulation 5 fruit shape is tapered oblong consisting of medium to short length, oblong shape, and a bit round at the bottom. The fruit shape of subpopulation 6 is short elliptical tapered, short, and quite similar to a pyriform shape (Fig. 7; Supplementary Figure S3). The fruit shapes of the accessions that display different genetic admixtures are affected by the admixture proportions of each subpopulation representative. The influence of each subpopulation representative on fruit shape was observed in admixed individual accessions containing substantial admixed proportions of subpopulation 1, causing a long fruit type or accessions containing admixed proportions of subpopulation 6, which causes a short fruit type.

## Discussion

### Population structure and diversity in L. acutangula

The whole-genome DArTseq based SNPs and fruit characteristics in this study enabled us to demonstrate the diversity of the 112 *L. acutangula* Thailand germplasm divided into six subpopulations based on STRUCTURE analysis. These findings are essential for *L. acutangula* breeding regarding parental selection to cross for desirable commercial fruit traits. Analyses using the STRUCTURE (Fig. 1) supported with AMOVA, weighted neighbor-joining method (Fig. 3) and PCoA (Fig. 4) statistics showed an almost consistent representation of a total of six subpopulations. However, the statistical power of PCoA was unable to differentiate two distinct subpopulations. This grouping consistency was more prominent at the level of individual accessions in both STRUCTURE (Fig. 1) and phylogenetic dendrogram (Fig. 2). It can be explained by the low *F*_ST_ values occurring between Cluster 2 and Cluster 1 (Table 2). The low differentiation observed between Cluster 2 and the other four Clusters (Cluster 1, 3, 5 and 6) may reflect the presence of genotype proportions of cluster 2 in those subpopulations, which indicates exchange among accessions collected from different regions. The highest pairwise genetic differentiation occurs between Cluster 1 and Cluster 4, and Cluster 3 and Cluster 4 may be because of a greater distance between the location of collected accessions resulting in a low admixture genotype.

### The geographical aspect of L. acutangula diversity

In the present study, population structure assignment corresponded to the geographic origin, which are similar observations in a *Capsicum* germplasm^30^. Geographically, Thailand's accessions make up two subpopulations collected from the central plain and scattered all over in Thailand. The scattering of the largest subpopulation emphasizes the wide range of acceptable genotypes throughout Thailand. A subpopulation made of accessions from northern Thailand and Laos shows the similarity in cuisine between the parts of the north of Thailand and Laos. The admixture proportions in the Vietnam accessions (Cluster 4) were found in accessions collected from Southern Thailand. Also, the main admixed proportion making up accessions from Vietnam is closely shared with accessions from the Philippines and Indonesia. This suggests a close relationship between people from these countries. In the past, this part of Southeast Asia may have shared a common ancestral history with the possibility of immigrations among mainland (Vietnam) and islands in southeast Asia (the Philippines and Indonesia) which probably lead to the exchange of goods between these countries in the past^31,32^. The observed distribution of a subpopulation (Cluster 4) to the southern part of Thailand point at the distribution of seeds along historic commerce ship routes from the Southern Thailand regions to countries such as India and China^32,33^. Peculiarly, the leading representative of a subpopulation (Cluster 5) is from the USA, perhaps collected somewhere from Asia, as Luffa is native to Asia and not the Americas^1,2^. The last subpopulation (Cluster 6) was made up equally of accessions from Thailand and Bangladesh with a genotype characteristic which might have been introduced from the Nepal region via Bangladesh^34^, but due to the lack of samples from Myanmar, confirmation of the trafficking of this material is difficult^32,35^ (Fig. 6).

The influx of genetic material (*H*_*O*_ > *H*_*E*_)^25^ occurs in four subpopulations (Cluster 1, Cluster 2, Cluster 5 and Cluster 6) (Table 1) which may be possible naturally or by the communication of the people in a close area. Two subpopulations (Cluster 3 and Cluster) are likely an inbreeding population (*H*_*O*_ < *H*_*E*_) caused by isolation due to barriers such as the mountainous area of Laos and Thailand and that of the island countries^25^. A closer look at the genotypes of ridge gourd in Thailand (Cluster 1) shows that the spreading of a subpopulation representative specific SNPs occurs throughout accessions that were collected nearby the area (Fig. 6). The same was true for the genotype of cluster 3, which was represented by accessions from Laos. Still, the other accessions in cluster 3 that were domesticated in Thailand were already admixed with the genotype of cluster 2, which is the primary governing genotype of ridge gourd in Thailand.

### Association of fruit traits of germplasm collection

Two subpopulations (Cluster 1 and 4) with accessions from Thailand, Vietnam, Indonesia, and the Philippines favor long fruits with a wide range of fruit shapes (Fig. 7; Supplementary Table S2 and Supplementary Figure S1). The long fruit type is found in the genotypes used by seed companies to improve yield for the benefit of the farmers, as seen in the commercial accession (LA-CM) in cluster 1. Medium length fruits with an elliptical tapered fruit shape occur in Cluster 2, which may have been the domesticated fruit type in Thailand before the seed company had bred and sold its commercial cultivars. Accessions from Thailand and Laos were grouped in Cluster 3. They had medium-length fruits with an elongated tapered fruit shape, indicating the similarity in culture and food shared across both countries. Medium length fruits characterized by elongated elliptical tapered were found in Cluster 5, which consisted of accessions from Thailand, China and the USA. The distinct shape is also part of the variation of ridge gourd fruit shapes found in Asia, such as Thailand and China. The fruits of Cluster 6 were short in length with a short elliptical tapered fruit shape. This observation in Cluster 6 may indicate that people in Bangladesh may prefer the short fruit shape, and the accessions found in Thailand may have been brought from there.

Each of the distinct clusters will prove a valuable source for selecting promising parental genotypes regarding fruit length and fruit shape characteristics. Choosing such material from different clusters will help maximize genetic diversity while efficiently meeting demands from consumers and producers. These subpopulations will also play a vital role in breeding programs to mitigate future environmental limitations such as resistance to plant pathogens and adjustment to a constantly changing climate.

## Conclusions

The study found significant genetic diversity in Thailand *L. acutangula* germplasm. Phenotypic information, such as the fruit length and fruit shape, displayed a corresponding variation according to the six populations inferred by the genotypic data. At the same time, geographical provenance was reflected in the clustering analysis, which also showed a relationship to the phenotypic information. This moderate diversity serves as a solid catalyst for utilizing such germplasm for breeding programs geared towards the farmer, consumer and vegetable exporter preference. The wide-ranging fruit lengths and shapes will help solve current ridge gourd exporter’s problems in Thailand. One main problem faced by ridge gourd exporters is fruit breakage during packaging caused by very long fruits. Farmers tend to grow long fruits to get a higher weight or yield.

## Materials and methods

### Plant materials

We used in this study an *L. acutangula* germplasm comprising of 112 accessions from Thailand (91), Vietnam (4), Philippines (2), Indonesia (1), China (3), USA (1), Laos (3) and Bangladesh (7), conserved by the Tropical Vegetable Research Center (TVRC), Kasetsart University, Kamphaeng Saen Campus, and the World Vegetable Center, Taiwan (Supplementary Table S1).

### Fruit trait evaluation

Current important fruit traits such as fruit shape and fruit length were evaluated. Accessions were grown from August to December 2019 under field conditions in which five plants per plot were planted in a single bed with a 0.5 m space between the plants and 2 m spacing between beds. Accessions were maintained by self-pollination. Fruits were harvested when they were young for consumption as a vegetable. All *L. acutangula* accessions were evaluated for fruit traits such as fruit length by measuring the stem-end to blossom-end of three fruits per accession, and fruit shape was adapted from the descriptors for sponge gourd^36^. Fruit shape was classified into either elongated slim, elliptical tapered, elongated tapered, elongated elliptical tapered, tapered oblong or short elliptical tapered. The fruit traits were further analyzed for correlations with subpopulations as a means to identify useful gene pools from which breeders can select potential candidates for trait introgression.

### DNA extraction

Genomic DNA samples were extracted from 100 mg of pooled young leaves tissue of 2-weeks-old seedlings from 20 plants per accession using a modified cetyltrimethylammonium bromide (CTAB) method^37^. Precipitated DNA was resuspended in TE buffer (10 mM Tris–HCl; 1 mM EDTA, pH 8.0) containing 2 μg/mL RNase. DNA quality was evaluated by electrophoresis on a 1% agarose gel and was quantified with a NanoDrop 2000c spectrophotometer V 1.6.0. The DNA concentration was adjusted to 50 ng/μL for DArTseq GBS analysis.

### Genotyping of accessions of L. acutangula using DArTseq

The genomic DNA samples were sent to Diversity Arrays Technology Pty. Ltd., Canberra, Australia, for DArTseq genotype-based sequencing^38^. To this end, DNA was digested using *Pst*I-*Mse*I restriction enzymes as described by Kilian^20^. The digested fragments were then ligated to adapters and amplified by PCR^21^, followed by sequencing on Illumina Hiseq2000. The single read sequencing was run for 77 cycles, and sequences generated were handled by DArT analytical pipelines (Diversity Arrays Technology, Australia). In the primary pipeline, poor-quality sequences were filtered from the FASTQ files by applying rigorous selection criteria to the barcode region^39^. Identified sequences per barcode/sample were used for marker calling. These files were then used in the secondary pipeline for DArT P/L’s proprietary SNP calling algorithms (DArTsoftseq).

### Population structure and data analysis

DArTseq based SNPs were filtered using a call rate of 80% with a co-dominant marker polymorphism information content (PIC) greater than 0.125. After filtering, 2834 SNPs were used for data analysis. The population structure of the 112 *L. acutangula* accessions was determined using STRUCTURE version 2.3.4^23^. Ten repeats were performed for each number of hypothetical subpopulations (*K*) which were set from 1 to 10. The parameters used consisted of an admixture model, a burning period of 50,000 steps, and 100,000 Markov Chain Monte Carlo (MCMC). The STRUCTURE results were further analyzed and visualized using the r package, pophelper version 2.3.0^40^. The optimum number of *K* was calculated using the Evanno method^24^.

We constructed the phylogenetic dendrograms with the weighted neighbor-joining method^41^ and visualized the data with DARwin software version 6.0.021^42^. Principal Coordinates Analysis (PCoA) graphs were created in dartr version 1.9.1^43^, expected heterozygosity (*H*_*E*_), observed heterozygosity (*H*_*O*_), and pairwise *F*_ST_ were calculated using adegenet version 2.1.1^44^ in the R statistical environment^45^. Differentiation among populations (*F*_ST_), total fixation index (*F*_IT_ ), and fixation index within populations (*F*_IS_ ) were calculated using the r^46^ package hierfstat version 0.5–7^47^. Analysis of Molecular Variance (AMOVA) and Shannon-Weiner Diversity index were calculated in poppr version 2.8.3 in r. Jaccard distance was calculated in r software by using ade4 version 1.7–13 package^48^. One-way ANOVA test, box plots and Tukey HSD were carried out using standard r statistics. Linkage disequilibrium (LD) for SNP markers was calculated using TASSEL v.5.0^49^.

### Permissions for ridge gourd collection

112 *Luffa acutangula* accessions were used according to the Standard Material Transfer Agreement (SMTA) from the World Vegetable Center (AVRDC) number SMTA-00AD43-00AV74-180920, and from the Tropical Vegetable Research Center (TVRC) number SMTA-62/033. Experimental research and field studies on plants did comply with relevant institutional, national, and international guidelines and legislation as well as SMTA guidelines.

## Supplementary Information

Supplementary Information.

## Data Availability

Data supporting the findings are available within the paper and the Supplementary Information file. Passport, characterization, and genotype data of the ridge gourd accessions from TVRC and AVRDC that were used in this study are available at http://breedserve.cab.kps.ku.ac.th/luffagermplasmdb/. Additional passport data for accessions from AVRDC is available at http://seed.worldveg.org/search/characterization/luffa. Datasets generated and analyzed during the current study are available from the corresponding author upon request.
