# Supplementary material for: Genetic diversity and population structure of ridge gourd (Luffa acutangula) accessions in a Thailand collection using SNP markers
Source: Sci Rep. 2021 Jul 28;11:15311. doi: 10.1038/s41598-021-94802-4 (PMC8319206; doi:10.1038/s41598-021-94802-4)
Supplement: Supplementary file 1 — Supplementary Information. [file 41598_2021_94802_MOESM1_ESM.pdf]

Supplementary Information

Genetic diversity and population structure of ridge gourd (*Luffa acutangula*) accessions in a Thailand collection using SNP markers

Grimar Abdiel Perez, Pumipat Tongyoo, Julapark Chunwongse, Hans de Jong, Anucha Wongpraneekul, Waraporn Sinsathapornpong, & Paweena Chuenwarin

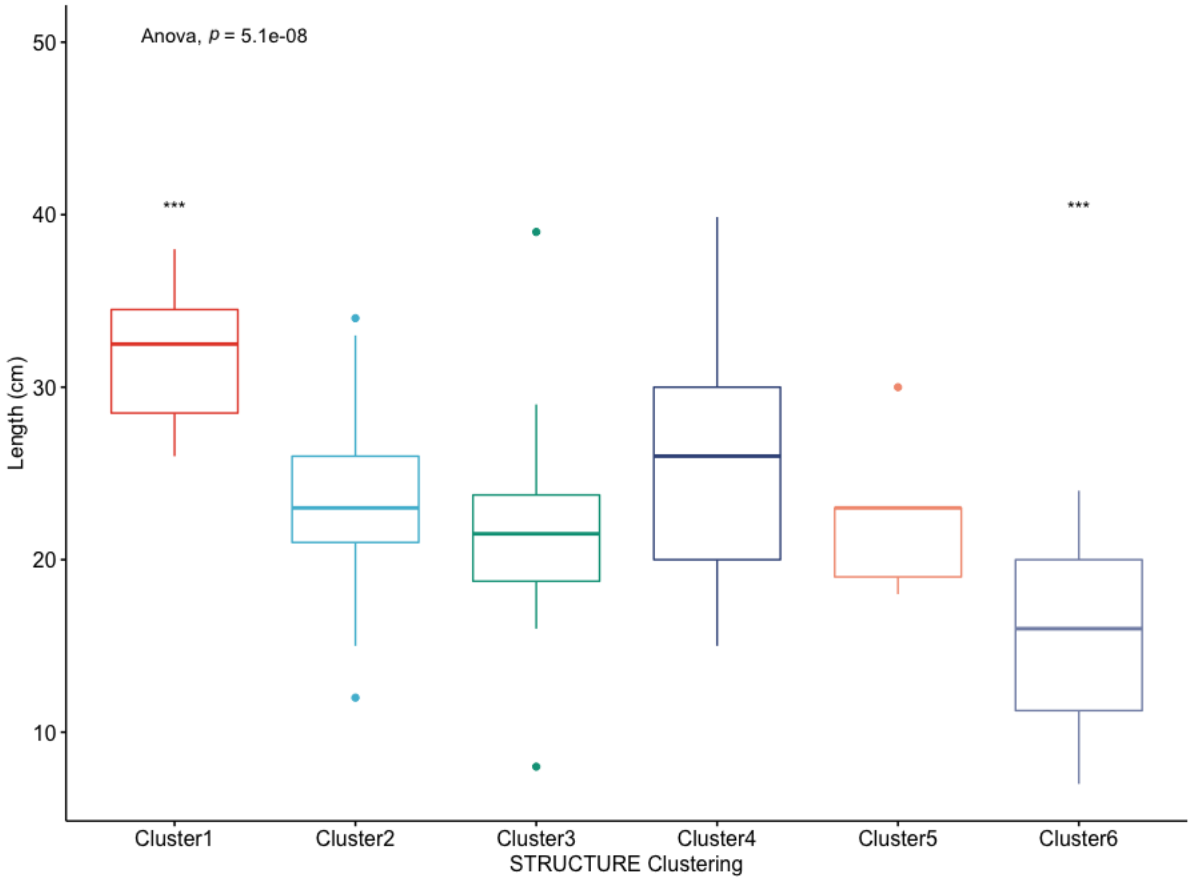

Supplementary Figure S1. Box plots of fruit length (cm) by STRUCTURE clustering.

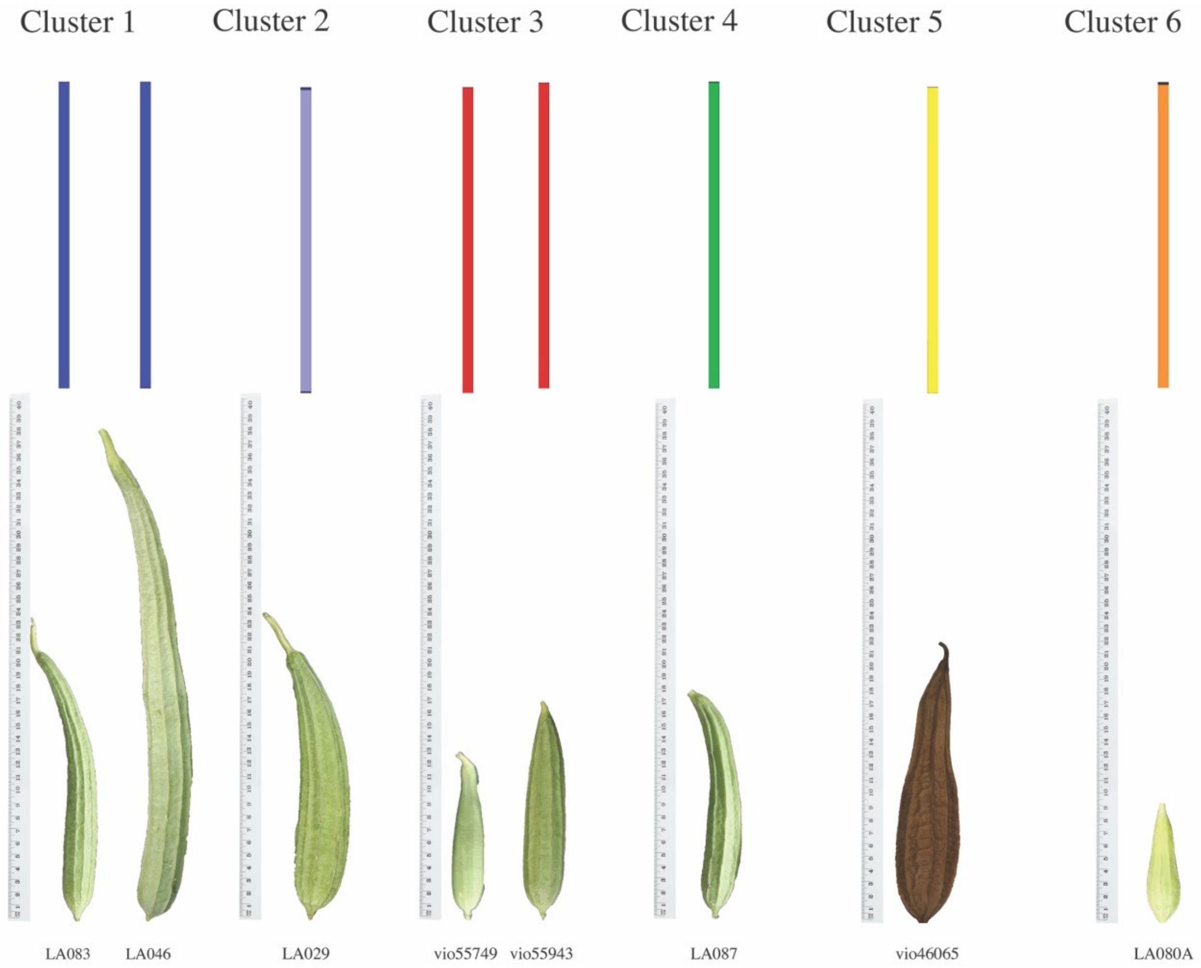

Supplementary Figure S2. Fruit shape of *Luffa acuntagula* representatives based on STRUCTURE grouping.

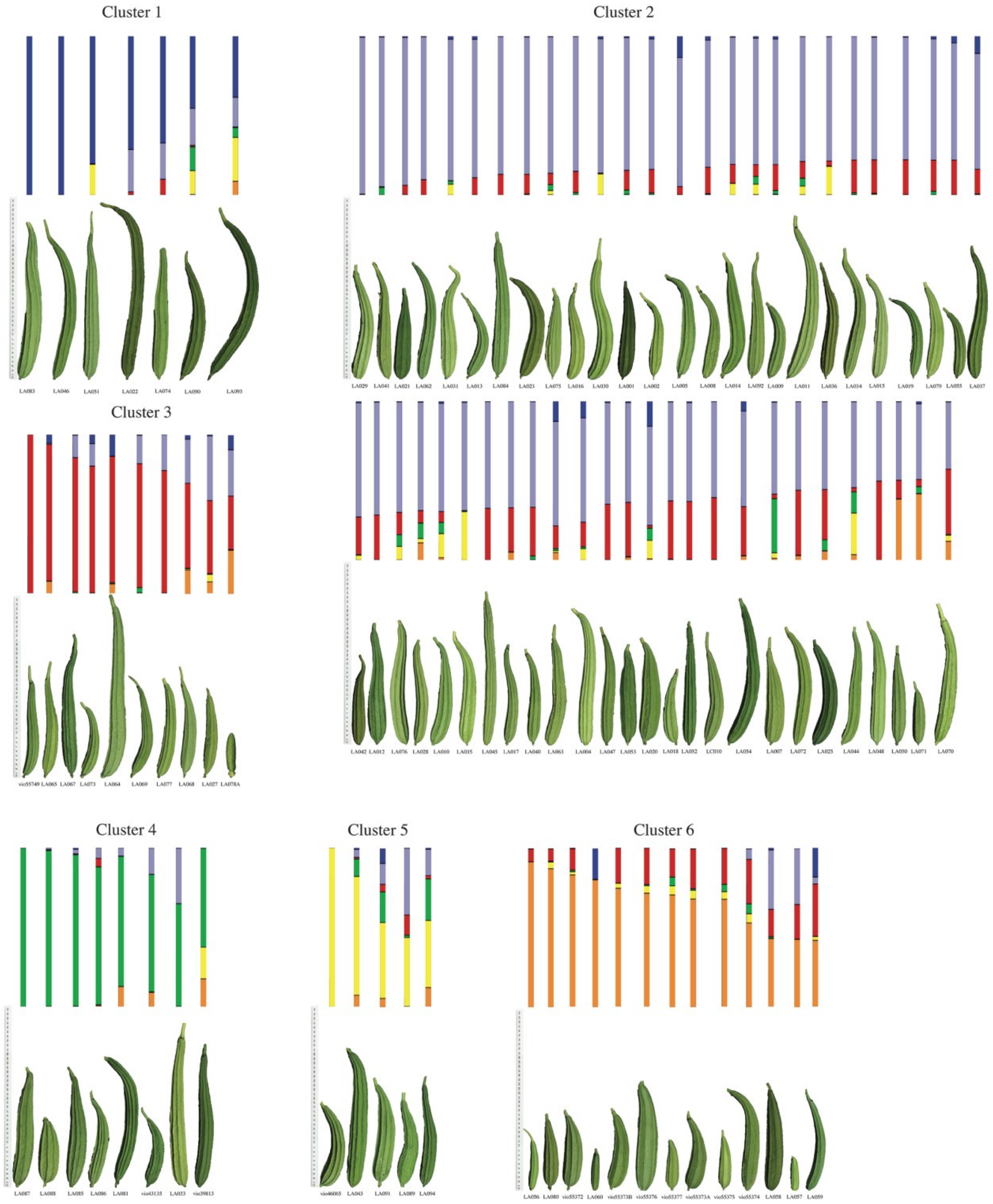

Supplementary Figure S3. Fruit shape of *Luffa acutangula* accessions based on STRUCTURE grouping.

Supplementary Table S1. Passport Data of *L. acutangula* accessions. *TVRC* Tropical Vegetable Research Center, *AVRDC* World Vegetable Center.

Accession number	Names	Country	Province/ State	Institute
1	LA001	Thailand	Chaiyaphum	TVRC
2	LA002	Thailand	Loei	TVRC
3	LA003	Thailand	Loei	TVRC
4	LA004	Thailand	Kalasin	TVRC
5	LA005	Thailand	Loei	TVRC
6	LA006	Thailand	Phetchabun	TVRC
7	LA007	Thailand	Pattani	TVRC
8	LA008	Thailand	Narathiwat	TVRC
9	LA009	Thailand	Ayutthaya	TVRC
10	LA010	Thailand	Ayutthaya	TVRC
11	LA011	Thailand	Lop Buri	TVRC
12	LA012	Thailand	Sing Buri	TVRC
13	LA013	Thailand	Sing Buri	TVRC
14	LA014	Thailand	Chai Nat	TVRC
15	LA015	Thailand	Chai Nat	TVRC
16	LA016	Thailand	Chai Nat	TVRC
17	LA017	Thailand	Uthai Thani	TVRC
18	LA018	Thailand	Uthai Thani	TVRC
19	LA019	Thailand	Chai Nat	TVRC
20	LA020	Thailand	Suphan Buri	TVRC
21	LA021	Thailand	Suphan Buri	TVRC
22	LA022	Thailand	Suphan Buri	TVRC
23	LA023	Thailand	Suphan Buri	TVRC
24	LA024	Thailand	Suphan Buri	TVRC
25	LA025	Thailand	Kanchanaburi	TVRC
26	LA026	Thailand	Kanchanaburi	TVRC
27	LA027	Thailand	Kanchanaburi	TVRC
28	LA028	Thailand	Kanchanaburi	TVRC
29	LA029	Thailand	Ratchaburi	TVRC
30	LA030	Thailand	Ratchaburi	TVRC
31	LA031	Thailand	Ratchaburi	TVRC
32	LA032	Thailand	Petchaburi	TVRC
33	LA033	Thailand	Prachuap Khiri Khan	TVRC
34	LA034	Thailand	Chanthaburi	TVRC
35	LA035	Thailand	Chanthaburi	TVRC
36	LA036	Thailand	Chanthaburi	TVRC

37	LA037	Thailand	Chanthaburi	TVRC
38	LA038	Thailand	Chanthaburi	TVRC
39	LA039	Thailand	Tak	TVRC
40	LA040	Thailand	Sukhothai	TVRC
41	LA041	Thailand	Nakhon Nayok	TVRC
42	LA042	Thailand	Prachin Buri	TVRC
43	LA043	Thailand	Chanthaburi	TVRC
44	LA044	Thailand	Chanthaburi	TVRC
45	LA045	Thailand	Chanthaburi	TVRC
46	LA046	Thailand	Nakhon Sawan	TVRC
47	LA047	Thailand	Kamphaeng Phet	TVRC
48	LA048	Thailand	Kamphaeng Phet	TVRC
49	LA049	Thailand	Kamphaeng Phet	TVRC
50	LA050	Thailand	Phitsanulok	TVRC
51	LA051	Thailand	Phitsanulok	TVRC
52	LA052	Thailand	Phitsanulok	TVRC
53	LA053	Thailand	Phitsanulok	TVRC
54	LA054	Thailand	Phitsanulok	TVRC
55	LA055	Thailand	Uttaradit	TVRC
56	LA056	Thailand	Uttaradit	TVRC
57	LA057	Thailand	Uttaradit	TVRC
58	LA058	Thailand	Uttaradit	TVRC
59	LA059	Thailand	Uttaradit	TVRC
60	LA060	Thailand	Uttaradit	TVRC
61	LA061	Thailand	Uttaradit	TVRC
62	LA062	Thailand	Phrae	TVRC
63	LA063	Thailand	Chiang Rai	TVRC
64	LA064	Thailand	Chiang Rai	TVRC
65	LA065	Thailand	Chiang Rai	TVRC
66	LA066	Thailand	Chiang Rai	TVRC
67	LA067	Thailand	Chiang Mai	TVRC
68	LA068	Thailand	Chiang Mai	TVRC
69	LA069	Thailand	Chiang Mai	TVRC
70	LA070	Thailand	Lampang	TVRC
71	LA071	Thailand	Lampang	TVRC
72	LA072	Thailand	Lampang	TVRC
73	LA073	Thailand	Lampang	TVRC
74	LA074	Thailand	Nakhon Sawan	TVRC
75	LA075	Thailand	Phetchabun	TVRC
76	LA076	Thailand	Phetchabun	TVRC
77	LA077	Thailand	Phetchabun	TVRC
78	LA078	Thailand	Loei	TVRC

79	LA078-A	Thailand	Loei	TVRC
80	LA079	Thailand	-	TVRC
81	LA080	Thailand	-	TVRC
82	LA080-A	Thailand	-	TVRC
83	LA081	Thailand	Narathiwat	TVRC
84	LA082	Thailand	Patthalung	TVRC
85	LA083	Thailand	Phichit	TVRC
86	LA084	Thailand	Phichit	TVRC
87	LA085	Vietnam	Ho Chi Minh City	TVRC
88	LA086	Vietnam	Ho Chi Minh City	TVRC
89	LA087	Vietnam	Tay Ninh	TVRC
90	LA088	Vietnam	Lam Dong	TVRC
91	LA089	Thailand		TVRC
92	LA090	China		TVRC
93	LA091	China		TVRC
94	LA092	Thailand	Nonthaburi	TVRC
95	LA093	Thailand	Kanchanaburi	TVRC
96	LA094	China	-	TVRC
97	LA-CM	Thailand	-	East-West Seed International Limited
98	LC010	Thailand	Loei	TVRC
99	VI039813	Philippines	La Union	AVRDC
100	VI039833	Philippines	Nueva Ecija	AVRDC
101	VI043135	Indonesia	-	AVRDC
102	VI046065	United States of America	-	AVRDC
103	VI055372	Bangladesh	Chittagong	AVRDC
104	VI055373A	Bangladesh	Chittagong	AVRDC
105	VI055373B	Bangladesh	Chittagong	AVRDC
106	VI055374	Bangladesh	Chittagong	AVRDC
107	VI055375	Bangladesh	Cox's Bazar	AVRDC
108	VI055376	Bangladesh	Kushtia	AVRDC
109	VI055377	Bangladesh	Nawabganj	AVRDC
110	VI055749	Laos	Savannakhet	AVRDC
111	VI055839	Laos	-	AVRDC
112	VI055943	Laos	Saravane	AVRDC

Supplementary Table S2. Descriptive statistics of *Luffa acutangula* fruit length (cm).

STRUCTURE grouping	Count	Mean	Median	Max	Min	Standard Deviation	Variance
Cluster 1	8	31.90	32.50	38.00	26.00	4.26	18.10
Cluster 2	62	23.20	23.00	34.00	12.00	4.44	19.70
Cluster 3	14	22.00	21.50	39.00	8.00	7.47	55.80
Cluster 4	9	26.00	26.00	40.00	15.00	8.40	70.50
Cluster 5	5	22.60	23.00	30.00	18.00	4.72	22.30
Cluster 6	14	15.60	16.00	24.00	7.00	5.60	31.30

Supplementary Table S3. One-way ANOVA based on STRUCTURE grouping. ***P-value < 0.001.

	**Df**	**Sum sq**	**Mean Sq**	**F value**	**P-value**
Structure clustering	5	1483	296.52	10.27	5.1e-08***
Residuals	104	3002	28.86		

Supplementary Table S4. Tukey HSD based on STRUCTURE grouping with a 95% family-wise confidence level. *Diff* mean difference between two groups, *Lwr* lower endpoint of the interval, *Upr* upper endpoint of the interval, *P adj* p-value after adjustment for the multiple comparisons.

Pairwise comparisons	Diff	Lwr	Upr	P adj
Cluster2–Cluster1	–8.7137097	–14.573774	–2.853645	0.001
Cluster3–Cluster1	–9.875	–16.994895	–2.7551046	0.001
Cluster4–Cluster1	–5.875	–13.454707	1.705	0.224
Cluster5–Cluster1	–9.275	–18.167746	–0.3822535	0.036
Cluster6–Cluster1	–16.2321429	–23.14562	–9.3186653	<0.001
Cluster3–Cluster2	–1.1612903	–6.08082	3.758	0.983
Cluster4–Cluster2	2.839	–2.725536	8.403	0.677
Cluster5–Cluster2	–0.5612903	–7.813173	6.691	1.000
Cluster6–Cluster2	–7.5184332	–12.134171	–2.902695	<0.001
Cluster4–Cluster3	4.000	–2.878472	10.878	0.542
Cluster5–Cluster3	0.600	–7.703153	8.903	1.000
Cluster6–Cluster3	–6.3571429	–12.493721	–0.2205648	0.038
Cluster5–Cluster4	–3.4	–12.100656	5.301	0.866
Cluster6–Cluster4	–10.3571429	–17.021724	–3.6925615	<0.001
Cluster6–Cluster5	–6.9571429	–15.083988	1.170	0.138

Supplementary Table S5. Raw DArTseq SNP sequence data used to generate the marker data for 112 accessions.

<table>
<tr><th colspan="2">Organism name: Luffa acutangula (eudicots)</th></tr>
<tr><th colspan="2">GenBank assembly accession: GCA_012295215.1</th></tr>
<tr><th>AlleleID</th><th>Sequence</th></tr>
<tr><td>39714580|F|0-32:G>A-32:G>A</td><td>TGCAGAGGCGGAGGGCTTTATGCGTCGTGTTGGCGAGATCTTAGTTCAGGTTGATAAGGTGAGGCCTCT</td></tr>
<tr><td>39715258|F|0-35:T>C-35:T>C</td><td>TGCAGCAAAGGTTGGATTGAATCACCACTGAAGGGTGCTGAGGGTAATACAGAATTTTTGGTTTGCTTT</td></tr>
<tr><td>39715420|F|0-48:A>G-48:A>G</td><td>TGCAGCACACTGTCCCTCCCGCATTCCCTTCGATACTCCAAAGTGATGAAAGAAAGTTCATGACTCTCT</td></tr>
<tr><td>39729507|F|0-48:G>A-48:G>A</td><td>TGCAGTATGAGAAGCATGCAATGTTATTATGAACAACTAATGAAAAGCGACTAAAGATGTATTAC</td></tr>
<tr><td>39718538|F|0-7:A>G-7:A>G</td><td>TGCAGTTACAACAGGCTCTCATGTTGATGCAATTCCATTCTCTGGGAAGTATGATGGAGTTGTTGGGGT</td></tr>
<tr><td>39722527|F|0-59:T>C-59:T>C</td><td>TGCAGCATCCACGGCTTCACATCTTCCAGCCTCGCCGCCGCCACGCCGCGGAAGGCGAATCGGACGTTC</td></tr>
<tr><td>39723188|F|0-16:T>C-16:T>C</td><td>TGCAGGACTTCGAGATTGATGAGCTTTCACAAGAGTATCTTGCCCTGCATCCAGTGGCTTCTACGAAGC</td></tr>
<tr><td>39715517|F|0-65:T>C-65:T>C</td><td>TGCAGCAGATCGAGAATCCTGTCATTCTTCTTCTCCATTGCCATAACCAATGCCTTCTTCACAACTTCA</td></tr>
<tr><td>39718010|F|0-43:A>T-43:A>T</td><td>TGCAGTCAACACCAAGGCATTTGTACAACCTCAAAGCCAAAATACGCCAAGGAAATCTATCAGGTTTTC</td></tr>
<tr><td>39726404|F|0-26:C>T-26:C>T</td><td>TGCAGGATCCGAAAATACTGAAACTTCTGGAGGGAGCTTTTGAATTTTCCAGCTCGTTTTTTTGCTTTG</td></tr>
</table>

<table>
<tr><td>39728037|F|0-27:G>A-27:G>A</td><td>TGCAGCTTTCTCACCCTGGAAGTGTAAGAATGTTATTACATTGATGACCCTAATTTAC</td></tr>
<tr><td>46776489|F|0-19:T>G-19:T>G</td><td>TGCAGAAGTAATGTAACTTTGTATCAATGGTTTAC</td></tr>
<tr><td>39716990|F|0-12:T>A-12:T>A</td><td>TGCAGGATGAGATGAAGCTTTTACAGATTGCCAAGGAAGTCGAAAGCGAAATTGTCGGTATGACATAAC</td></tr>
<tr><td>39718036|F|0-68:G>C-68:G>C</td><td>TGCAGTCACTAAGTTTTGAGTAGATTGATAAATTCTGCACCAAATATCTTGCTTTTCCTTGTCAAAGCG</td></tr>
<tr><td>39715910|F|0-40:A>C-40:A>C</td><td>TGCAGCCATTGCCGTCGCTGGTTTTGGTGGTGGCGATGGGAGGAAGAGGAGGACGATTGGGATTTGGGA</td></tr>
<tr><td>39723455|F|0-49:C>T-49:C>T</td><td>TGCAGGGAGTATCAGTAAACCAAGGATCGATGGTTATCTCGAATATATACTGTATGTTGTTTCTGTATT</td></tr>
<tr><td>39728405|F|0-30:T>C-30:T>C</td><td>TGCAGTTCAATTTCAGGTAGCAAGGAAGCATGCCATACTTTTTCAATTTGCTATTCATAAAATTTCTCC</td></tr>
<tr><td>39715824|F|0-16:T>C-16:T>C</td><td>TGCAGCCAAGGCTGGATTATTGCCCACCACCAGAGGATTTGCAGGTAATATAGTACTCGGGGACATCAT</td></tr>
<tr><td>39724599|F|0-22:C>A-22:C>A</td><td>TGCAGAAAGTGGATGATTGACGCATTAC</td></tr>
<tr><td>39714498|F|0-24:C>T-24:C>T</td><td>TGCAGAGCATCTCTGTCTTCCTTGCCGAAGCCGAATGGGGTTTGTTCGAGAATCGCGAAGGGATCGAGG</td></tr>
<tr><td>39719813|F|0-32:T>G-32:T>G</td><td>TGCAGATTTATCTCTGAAGTAAGTCTTTTAGCTAGAGTTGACTTATGGATCTTAC</td></tr>
<tr><td>39720743|F|0-18:C>A-18:C>A</td><td>TGCAGTGCTTGCCCTCCACGACTTGCAACTTAC</td></tr>
<tr><td>39717990|F|0-60:T>A-60:T>A</td><td>TGCAGTATTTGGTTGGATCACTGGCCTTTGTTCTGCTTCCCATGTTGTTGGAAATTTGCTTGCACGGTT</td></tr>
<tr><td>39715904|F|0-39:T>A-39:T>A</td><td>TGCAGCCATGTAAATAGAATCTTATTGTAATTCCTAAAATGAAAAATAATAGGAACAAAGCAATAAGCG</td></tr>
<tr><td>39716267|F|0-64:T>A-64:T>A</td><td>TGCAGCTAGAAGAAGAGGGCTATTTATAGATAACGAGAGGCATTCAAAGGACTAAAATACTCCTTGGGA</td></tr>
<tr><td>39717816|F|0-50:A>T-50:A>T</td><td>TGCAGTACAACCAAAGAGATACATGACTATTTCAATCAGCCAATTTTGCTATTCTCTTCTTCCCATAAA</td></tr>
<tr><td>39721535|F|0-30:A>G-30:A>G</td><td>TGCAGAATTTCTATCCTCGGCTGTAGCTTCAAAGCCCACTGAGAACAAAAGCTTTGGCTTTGACTTTGC</td></tr>
<tr><td>39720233|F|0-27:G>A-27:G>A</td><td>TGCAGCTTGAAAAGCCTATAACGGCGGGGCTTGTGCTGGTTAC</td></tr>
<tr><td>46753974|F|0-33:A>G-33:A>G</td><td>TGCAGAAGTCGCTTCTGTTGTAGTAGCAACTTCAACAGAGCAAGGCTCATTGTTTTCTTCACTCTCCGA</td></tr>
<tr><td>39720809|F|0-6:T>C-6:T>C</td><td>TGCAGTTATCAACTAAATGCAGCTACCTACACTAACCATAGCATCAACTCTATGCAATTAC</td></tr>
<tr><td>39714301|F|0-37:T>C-37:T>C</td><td>TGCAGACTGGGCATATTTGATTGCCATGTTTTACATTTGACACGACGCAGTGAAAATGGAAGGAATGTG</td></tr>
<tr><td>39715521|F|0-21:A>G-21:A>G</td><td>TGCAGCAGATGCAATTCTTCGAAACACGTTATTGAAATCGCCCAATTTTGTGCTGATAGGTATTGGAGC</td></tr>
<tr><td>39716415|F|0-25:T>C-25:T>C</td><td>TGCAGCTGCAAGCACATATTCTCTTTTCTCGCTCAAAATCCCACCTCCCCACTGAACCATTTCTCCTTT</td></tr>
<tr><td>39714868|F|0-43:T>C-43:T>C</td><td>TGCAGATCAGTCACCGTAGCCACAGCAACACCAACAGATACCATTACAAGTGCTAGGACCTTGGCGACA</td></tr>
<tr><td>39724040|F|0-52:G>A-52:G>A</td><td>TGCAGTGACAAACTAAGGAAAGAAAGTAAGAAAGGAGCACACTTGTATCTTTGGCAGATAGCTCAAAAT</td></tr>
<tr><td>39717341|F|0-25:T>C-25:T>C</td><td>TGCAGGGGATGTCGAAGCCAGTTTATTTGCTGAATATCACCGCCCTGTCGCAGCTGAGAATAGACGGCC</td></tr>
<tr><td>39717518|F|0-16:T>A-16:T>A</td><td>TGCAGGTGAAGCTTGGTTGGATTCATTCAAGTAATGTCAAATCCAAACATGTACTGTGTTAGCTTACTG</td></tr>
<tr><td>39725117|F|0-28:A>G-28:A>G</td><td>TGCAGGCGGTTCCCTTTCCCAAAATGTCATTAC</td></tr>
<tr><td>39718075|F|0-67:C>T-67:C>T</td><td>TGCAGTCCAAATCCAAGGTTGATAAGCTGCACTTCTTTCTACGAACCTTCCCCATGTGTCCCACCTGCT</td></tr>
</table>

39722225|F|0-32:T>C-32:T>C	TGCAGCAAAACATTGGATGTTCCAACTTCAAATTTACATGCCTATGTCAAGCCACCCTCACATGAAGGT
39723524|F|0-59:T>C-59:T>C	TGCAGGGTGGGTGATGGACCTATAGCAGGATCTTCAGCATATGCAGATAGTGATATCGGTGCGTGTGGA
39724941|F|0-35:A>T-35:A>T	TGCAGCCAAACGGGCGAGAACTGATGGTATGCACTATTAC
39717502|F|0-12:C>T-12:C>T	TGCAGGTCCCCGCTCATCAGACTCGCCACAGTTAGCAACAACAAGTGCTGAATTAGGCCCGAGAATGTT
39726307|F|0-68:G>A-68:G>A	TGCAGCTGGTCTAGGATTTCTCAAGTAGGTTGTCCTGACTATTGAATCTTGGGCTGCTTTCTTTTAGAG
39723642|F|0-62:C>T-62:C>T	TGCAGGTGTAACTGCGGATTGGTGGTCTGTTGGAGTAATTCTTTTTGAGATGCTTGTTGGAGCACCCCC
39725502|F|0-55:A>C-55:A>C	TGCAGAAAAGTTAGCACCACGGAATCTTTAGTCTAGTAGAAACAAACTCAAGATAAAACAGTCATTTAC
39724652|F|0-46:C>T-46:C>T	TGCAGAAGTCACTAAGAGATTCAAAGATTGCTGATAATATTTCAATCGATCAACTTAC
39720426|F|0-8:T>G-8:T>G	TGCAGGCTTTTGATCTTGGAGGGATTTTGAAAGTGACACTCTTAC
39722853|F|0-9:T>C-9:T>C	TGCAGCTACTGGGAGCAGTTAGTTGTAGCGTGCGCTATCGATGTACTATCAACATTTTACATATAAGTT
39728556|F|0-25:C>G-25:C>G	TGCAGCACGGCGTCACCGGGGGCTTCCCGATGGTAACGTCGACGAGGCCGCCGAGCAGCTTGGCGCAGA
39714405|F|0-51:A>G-51:A>G	TGCAGAGAGAAGAACAATGGAGACACCGTGCTACACCGCGCCCTCAGAGACAACCACTTTGGTGAGCTT
39723643|F|0-42:A>G-42:A>G	TGCAGGTGTCTCTGATGTACAAACTGAAACTGCTACATGTCTAGATCCTGACCAGTTCACAAATTTTTT
39716903|F|0-12:T>C-12:T>C	TGCAGGAGCAATTCCAATTGGTGGGAGACTTACTTTACTCCAATTTTATTTATATTTCTTTTGGTTTA
39717813|F|0-54:C>T-54:C>T	TGCAGTACAAACCTAAATTCGATGCGTTGCATGTAGAATTGAAGGAAGCTGAAGCGAAATCCCTAAAGG
39713403|F|0-54:T>C-54:T>C	TGCAGAAAAATCACAGATCACATTCTTGCAGCCTATGTAAGAAATAATAATAATTGTTCGATCTTTCAG
39722434|F|0-52:C>T-52:C>T	TGCAGCAGCATTTGTATTGGTAAACCCATTGACTAGATAATGAAATACAACACCATTAGGTGTGCACCT
39724838|F|0-42:C>T-42:C>T	TGCAGATTCAGCCCTAATACAATATCAGAACACCCCCACAATCGTTCTTCCCAAATTAC
39727666|F|0-26:A>G-26:A>G	TGCAGGAACCAGGTATCTTATCGACAAATTCTGCTGTTGAAGTAAACTCCGAAAATACACCACCAAAAG
39723197|F|0-44:C>T-44:C>T	TGCAGGAGAATCGCCGGTGCATCAACTGCAACAGCCTGGTACACCATTACTACCGTTCTCGCTGATATC
39718625|F|0-39:T>G-39:T>G	TGCAGTTCATCTACTCATACATGGGATGCTTTTCAGCTTTCACATGCTGCTTTTAGGCTTCATTGTGTG
39714155|F|0-11:G>T-11:G>T	TGCAGACAGAGGGATGTCCTCAACCTGCCCAAGGAACCAGTGTCAGAGGAGCTTTTTTACAAGTGTCAG
39715669|F|0-42:C>G-42:C>G	TGCAGCATATATAAAAGACTTCCAAAAAAGGTTTCTTACACACTCTACTTTTGGAAATGCTTTCATTGG
39718786|F|0-26:T>G-26:T>G	TGCAGTTGGCCTGTAGTCAGTATTTTTTTAGACAACCCAGCGGTTCAAATATCAACCTTTACCAACACT
39719954|F|0-6:A>C-6:A>C	TGCAGCAGCCGCCCCGCCGCCGATCATGGAGTAAAAATCCGTGTGATTAC
39717492|F|0-56:T>C-56:T>C	TGCAGGTCACTTATTTATCGGCTCAAAGAAGTTGATCTCAAAGCGATGAAACACGATGAAAAGCTCGCG
39713563|F|0-15:A>G-15:A>G	TGCAGAAAGGTGGGGAGATGTGGTGGCTGTAAGAGAGGAGATGAAGATGAAAGGGATTGAAAACAAAGC
39713274|F|0-28:C>T-28:C>T	TGCAGCAATAAGATATAATAAGAACGAACAACAAATGAATGAAATAAAAATATAAATGAATAGCTGATA
39724339|F|0-51:C>T-51:C>T	TGCAGTTGCTCTAACTATAGAATCAGAATTGTGAGTAGACTTCATCGTCAACGAGTTCTTGGAAGTGTT

39714965|F|0-10:A>C-10:A>C	TGCAGATGATACTTGCTGATGGGCACAAAATTCCAACAATCTCATTTCTTCTTTCCTCCCTTGGCAGCA
39721026|F|0-45:T>C-45:T>C	TGCAGTCATTCAGCAGCATCTGGTTCATCCAAATCCAGAGATTTGTGAGAAGAAGTGACTGTACGTACC
39718286|F|0-17:G>T-17:G>T	TGCAGTGAGCTTTTGACGGGTGTTGTTCCATATACTGATCTTCGTACTGAGGCACAGGTCAGATATGTT
39720444|F|0-33:C>T-33:C>T	TGCAGGGCAGATAAAATAAACATAATGATTGAACCTTAC
39717779|F|0-27:A>G-27:A>G	TGCAGTAAGTACCAACACATTCAGTCAATGTGGCTTATTATTTCTATCATTCATCTTTATTCTGTTGCT
39716801|F|0-56:C>A-56:C>A	TGCAGGAATACGTGGATCATTCATCAACTCTCTACAAATTTTACGTACTAGGTGAACAGATTTTTTATG
39716273|F|0-13:G>A-13:G>A	TGCAGCTAGCACCGTAAGGCCACCACCTGAAAAGATAGGCGGCATTTTTCTGAAAGCTTTCATAGTGAG
39720810|F|0-36:T>C-36:T>C	TGCAGTTATCATCTCCGCATGGTTTAGAGGCATTCGTGTTCGATCTGATTTGCAGTTTAC
39716222|F|0-5:C>T-5:C>T	TGCAGCGTTGTTTCTTCATATCAGTAGCTAATGATTCTCTCTTTTGTAGATCAAGTGTCACAATGTGAT
39716568|F|0-7:T>G-7:T>G	TGCAGCTTCCGCCGGGCTTCCGATTTCACCCCACCGACGAGGAGTTAGTGGTTCACTATCTCACAAGAA
39717344|F|0-9:A>T-9:A>T	TGCAGGGGCAATAGATTGTCATGTGCACTTCATATGCCCTCAACTAGCATATGAAGCAATATCAAGTGG
39728011|F|0-42:A>G-42:A>G	TGCAGCAGTTGGCCCAATATATGGACCTTTCACCAGCAAAATATTCTTTAC
39715790|F|0-34:T>C-34:T>C	TGCAGCATTTGGATCTGTCTTCGAATAGATTTTATGGGCCGATCCCGGAGCGGATCAATGATCTTTACA
39719730|F|0-8:C>T-8:C>T	TGCAGATCCCCCATGGGAGAATTAC
39721190|F|0-68:C>T-68:C>T	TGCAGAAACTGTCATTACATTATGGGACCCTGAGCAGAACATCCTCGTTGCTGTAATTGGAGAGACTCC
39719744|F|0-22:A>G-22:A>G	TGCAGATGAACTGCATGCACTGACTCTTGGGAGAAGCATTGTTAC
39719458|F|0-46:T>A-46:T>A	TGCAGAATCGTACAGTGGAATTTTCTCAACTGAATACCTAAAACCGTGCAAGACGGTTAC
39729134|F|0-31:C>T-31:C>T	TGCAGAAACTTGAAGAGGATCTACAGACGTTCGGAATGAAAATAAAACAGCACGAGGACAACATTACAG
39715215|F|0-26:C>T-26:C>T	TGCAGATTTTTGTCACACACCTGTCCCCAAGTTGAGATTTATTATAACATGATGGAGGAGATTCTTCTG
39729371|F|0-10:G>C-10:G>C	TGCAGCTCAAGAAAGAGTTTTATTGCTTCTTCACTCGATCCCTCATTTGAGTAACCTGTCATTAC
39717367|F|0-34:G>T-34:G>T	TGCAGGGTAAATCAGTTGAGGTTCGACAAGCTGCGGGCCTGCTTCTGAAAAACAACCTTAGAACTGCAT
39718697|F|0-27:A>C-27:A>C	TGCAGTTCTTTTAGTCATAGTCATTCTAGCTCTTCTCTTTTTCATCTCTGGTCTTCTTCACCTGCTTGT
39716551|F|0-32:T>C-32:T>C	TGCAGCTTCAGTAACTCTAACATGAGCCTGAATGTCCTCATCTGATATTTCAACTTCAGTAATAGGAGC
39721409|F|0-53:C>G-53:C>G	TGCAGAAGGGCATTGGAATTATTCCACTCCATGTGGATGAATGGTGAGGTCGACCCTAATGAGTTCACT
39718167|F|0-55:T>G-55:T>G	TGCAGTCTCCCAAAGGAGATGTAAGTTATTTGCAGAAGTTCTTTCTAATGTACGTTGGAGGTAGGCAAC
39717545|F|0-58:C>T-58:C>T	TGCAGGTGCTCGGAAATGCGAAGGCGGCGGTGGCCGCCGTCGTGTCGATTCTGATTTTCAGGAATCCGG
39724156|F|0-51:G>A-51:G>A	TGCAGTGTCCAAAGGATGCACATCAAAAGATGGCGAGTTCGCTCTTCCCTCGTCTGTTGGTGTCTGCTA
39728142|F|0-65:T>C-65:T>C	TGCAGAACACCTCCCGTCTTTTGCTCTGCAAGATGACAGTATTGTTGAGATACAGGGGGAATCTGTTGC
39720147|F|0-15:C>A-15:C>A	TGCAGCTCAAAATAGCATCTTGATTTAC

39727768|F|0-33:A>G-33:A>G	TGCAGGTTCAGTGTTACTCTTGCGAAGCTTTTTATCGCGCTTCTTCATCCGTCTATCCATCTCACTCTG
39721829|F|0-29:T>C-29:T>C	TGCAGAGCTTGCCAGTGTACAAATTCTTCTTGTGATCGCATTTATTTATAATTGGCCTTTTTAC
39724096|F|0-12:A>C-12:A>C	TGCAGTGCTTCTAAAAAAGTTTGCTGGTGAAGAAGGAGAGAGGGTTGACGTGCAAAAATTGTTTGGATA
39713430|F|0-19:A>T-19:A>T	TGCAGAAAAGATCCCCACCACCTTCATCTTCCCGATTATCGTCCCAGTCAAAAACTCTTGATTCTCCAA
39716313|F|0-51:G>T-51:G>T	TGCAGCTCAATTGTTCAGATTTTCGCACCAGATTCCTTCGTTTCCACTCACGGGTGACGAAAGAGATGA
39713334|F|0-67:T>C-67:T>C	TGCAGCTACAGCAAGCTGTTTCCTCACGATCTCAACCACCTTATCCACGGTCTCTGGTTTAGCCTGTTC
39725034|F|0-46:A>T-46:A>T	TGCAGCTTGAAAGGCTTCAAAGTCTTCCATCCACTGCATCCACAATACCTTTCTTAC
39717197|F|0-19:G>T-19:G>T	TGCAGGCTGCAACTCCTCCGCTTGTTTTCATTTGAATTGTTGTATCTATTGTTTCGTTTCGTGTCGATT
39719686|F|0-26:G>A-26:G>A	TGCAGATACCAGAATTTGGGTTGTCAGGATATTTTTCCTTACTACAGTAAGCTTAC
39729378|F|0-25:A>C-25:A>C	TGCAGAATAAAATATGCAATAGATTATTATCTATACCAAACTACAAGAAATACTAGATGATTAC
39726956|F|0-5:A>C-5:A>C	TGCAGAAGAAGAACGAACACAAAAACAACAAATTAC
39713969|F|0-38:T>C-38:T>C	TGCAGAATACCTAAAATTGAAATCGGAAGCAATTCAAGTAAGATCGAGTCGAATTGCTGCTGAGAAAAT
39725745|F|0-67:C>A-67:C>A	TGCAGAGCAGAACAGCATAGATATGCTCCAATCCATTGTCCAATGTGGTGGAGACGTAACACTGTCGCG
39718614|F|0-51:A>C-51:A>C	TGCAGTTCACATGGGCTAGATGGTTATTTCTATTTCCTACTTTGTCCCTATATGCTTGAATATAGTCAT
39713918|F|0-56:G>T-56:G>T	TGCAGAAGTACAATGAAAAAGAGGGTAAATATTATTACAATTACAAATTCTTTCAAGTACAATTTCAAA
39717412|F|0-60:T>C-60:T>C	TGCAGGGTTTGCAGGGTTTGCACGTGCTGGGTCTGAATGTTCTTATAACTACTAAATTGCTTGGATTTT
39722465|F|0-23:G>A-23:G>A	TGCAGCAGGCAACTCTGACATTGGTACAAGTTCAAATTCACAATTGGTTACTTCTGGAACTTGTTCTCC
39720542|F|0-24:T>A-24:T>A	TGCAGGTTGGTCTCCTTAGTGCATTCATAATTAC
46757789|F|0-68:A>G-68:A>G	TGCAGTTTTACTAGACTGATATATATAAATTATTAGAAATATAATTATAAGTTATGTTGTGTTGTATCA
39719703|F|0-10:C>T-10:C>T	TGCAGATATGCGTATGCATCTTGTGTGATAAATATGTTTTCCAGGATTAC
39714857|F|0-14:G>C-14:G>C	TGCAGATCAATGTCGGTCGTTCAATAGTAGTATATTAGCCTTTCTCTGATAATTCTGTCCAAGCAAACT
39715423|F|0-12:G>T-12:G>T	TGCAGCACAGGCGGCCAAACAACTCCTACCATCATGATAGCAACATGCATAACTCAATAAACTAGTATT
39726341|F|0-24:C>T-24:C>T	TGCAGCTTTTCATGAAGAAGCAATCAGATTCAAATCATAATGCTTACTCTAAGATTTCCCACTCCAGAA
39719856|F|0-9:A>G-9:A>G	TGCAGCAACAAACAGAAAAGAAAAGGCTTAC
39720903|F|0-18:G>A-18:G>A	TGCAGTTTGCAGTGAAAAGGAGAATGCCCATCAATTGCCAACTTTTTAC
39721507|F|0-47:C>T-47:C>T	TGCAGAATGGCTGTAGCCCATCACTGGGAAGATTGGATTTATGGAATCTACAGGTGTAGAACCTCCAGG
39714510|F|0-14:T>G-14:T>G	TGCAGAGCCCCATTTCACTTATGAAAGTTATTGGATCACAATTCATGTTTACAATGTCTGATTCCTTGC
39716283|F|0-65:C>T-65:C>T	TGCAGCTATAGTTGATTGGAAATAATTATGCAAATATGTCGTGAAAATATTATGTTCGTTTCAATCTTC
39714416|F|0-59:C>T-59:C>T	TGCAGAGAGAGATGAGAAGCCCATGCAATCTCTTGCTTTAGAGTTGTCTCAGAATCAATCCGTAGAATC

39727793|F|0-35:T>A-35:T>A	TGCAGTAGCTGGGTTTGCGAGGTCTCAGGGGAAGATTCCCGTCTGTTTCGGGGCTACGGATCTCGTGTG
39718761|F|0-64:C>T-64:C>T	TGCAGTTGCTGAAATATGTTCTGATTTTGATCCCACAATGAATATCGAGCCATCCCTTTTGAAGCTATT
39723184|F|0-66:A>G-66:A>G	TGCAGGACCTTCATCTCCTCAGGCTACCACCTTCTTGATTTCCGACATAAATGCTACCTTTACCAAATT
39725053|F|0-6:G>A-6:G>A	TGCAGGGAACTGGTTTAGCCTCTTTAC
39718143|F|0-15:C>G-15:C>G	TGCAGTCGTTCTTTTCTGGGTGGGTTCCTATTTCTTTTGTATCTATGCCTGCATAGCAGATTCTATGGC
39714364|F|0-5:A>G-5:A>G	TGCAGAGAAGTTGCAAAGGATCTCAATGCAACCTGCGTTATTGGTAATCAAGAGGATAAAAGAATTTGG
39713649|F|0-35:A>G-35:A>G	TGCAGAACAACATGTTGCCTAACAAAAATAGAACAAATGAATATGAATACACATACCTGCATATGCAGA
39716809|F|0-37:T>C-37:T>C	TGCAGGAATCATTCCATGCGTTTTTCTGATTGTTGGTTTGTGGTTCGTTCCGGAGTCTCCTCGATGGCT
39718311|F|0-39:G>A-39:G>A	TGCAGTGATGCCGGGACAATAGACCATGTGACAGGATTCGAGCATGGTGCATCTCTTCACTAAATGCAT
39726542|F|0-9:T>A-9:T>A	TGCAGGTGGTCATGCCAAAACAGTAATGTTTGCACATGTAAGTCCAGAAGAAGATTCTTTCAGTGAGAC
39716251|F|0-44:T>A-44:T>A	TGCAGCTACACCTCGGTCCCCAACATAGCAACCCTAGAAAATATTAGAGTTGAATAAGTACTTCTCAGA
39716988|F|0-6:T>A-6:T>A	TGCAGGTTGAAATCTAATAAATCACCATACAAGCAAATTCTACTAGGATATAAATCAAAAGGCATGCTC
39718947|F|0-16:A>G-16:A>G	TGCAGTTTTCATTCATACACTTGTAAAATGTTGTAGCAGGTTCATCAGCTGATCTCAGTTGTATTTGTA
46769502|F|0-64:A>G-64:A>G	TGCAGGGAATTGTTCCAGACAGCTTGGGAGAACTTGAAGATCTTCATCTATTGTAAGCGAGCTCATACC
39714973|F|0-58:T>C-58:T>C	TGCAGATGATGGCGGCGGAGGACAAGAAGAGGGTGGTGGTAGTAGGCGGAGGAATTGGTGGCGCTTTTG
39724308|F|0-30:T>A-30:T>A	TGCAGTTGACATGAAAAGCAACCGCTATAATACAAGAAAATTGGCATTCTAAGTAACATCCACAGAAAA
39719865|F|0-9:G>T-9:G>T	TGCAGCAACGGGTTCCTTTTTCACTCTGATGCTTCTAGGGCTTAC
39714359|F|0-63:C>T-63:C>T	TGCAGAGAAGCCTGATGTGATGGAAACTGAAACAAATGTTGGCGAGCCTGAAAGGAGTGATTCCGATGA
39716200|F|0-39:C>T-39:C>T	TGCAGCGTATGCCATTGAAAGGGTAGCAGATCCTGCTCCCGCCTTTGCTTCCACAACTTCGGTTCCCGC
39713741|F|0-37:C>T-37:C>T	TGCAGAACTTGCAACGTGAACTTGTGAGAGATTTACGCGAGGTATTTCAATTAGTTATATCTTTCTCTT
39722858|F|0-26:A>T-26:A>T	TGCAGCTACGGCGGCAAGATCCTCCCACGTTATCCCGACGGCAAACTCCGTTACCACGGTGGCGAAACC
39720106|F|0-23:T>C-23:T>C	TGCAGCGGGATTTGCAGGTCTGCTGAGAGGATTAC
39714631|F|0-32:C>T-32:C>T	TGCAGAGGTTTTGGTGGTATTGGAGGAATTCTCCGTTACCAGCTTGACATGAGGTCGTTTGATGAACTA
39718108|F|0-33:G>A-33:G>A	TGCAGTCCTACTCCAAGCGATTTTATGTCAGTTGCAATACTTGTGATGGTTTTTGAAGATGAGCAAGAA
39718685|F|0-23:G>A-23:G>A	TGCAGTTCTGTTTTGTGCAACATGAATAACAACATTTGGGTTTGAACTAGGTACAGTTTCTCCATGACT
39724582|F|0-53:T>G-53:T>G	TGCAGAAACACCTGCTTGTGAAGTCCTATCCATAGTTTCATGGATCTATTTTTTATTAC
46755244|F|0-42:T>C-42:T>C	TGCAGCAGGCAAGATGCAGTTTTTTTGTCAAGACTTCAAATCTTTTTTTAGGTACAGACAGAACATATG
39717314|F|0-58:C>A-58:C>A	TGCAGGGCGACTTTATGAAAATGCCGTTTGAAGATAATTCATTTGATGCAATATATGCCATTGAAGCCA
39724024|F|0-36:G>A-36:G>A	TGCAGTGAAAAGCCCATCAAGATGGAGCAGATCATAGGTTCTAGGATATGTTGAAAATGCCTCACACCT

<table>
<tr><td>39718876|F|0-67:G>A-67:G>A</td><td>TGCAGTTTCGATTCAGAAAGACCTTCCTACTTCATGTGTGGAGATGTTGGACAGGTTCGAATATTACGC</td></tr>
<tr><td>39718709|F|0-54:G>A-54:G>A</td><td>TGCAGTTGAAGAGTGTTTTTCTTGGCCAAGCTCCATAAGCAGGTTGGAAGCACTGCTTCTATCACCCAG</td></tr>
<tr><td>39713351|F|0-14:A>G-14:A>G</td><td>TGCAGGATGAGAGAATTGTTGCTTTTCAAGATATAAACCCCTCTGCTGTCAGGTATTTGAAACCTCTAA</td></tr>
<tr><td>39714266|F|0-17:T>A-17:T>A</td><td>TGCAGACTAGCTTCTCCTAACCCAAGGACTTACATAGGATCTGGGAAAGTTGCAGAAATCAAGAGTGCA</td></tr>
<tr><td>39717246|F|0-40:A>G-40:A>G</td><td>TGCAGGGAAATTATTATCCAATGCCTTCTTTAGCATTCATACAAGGATCCAATGGTCAAAGGTTCTCTG</td></tr>
<tr><td>39717992|F|0-27:A>G-27:A>G</td><td>TGCAGTATTTTGAAGGGTATGGTTATCATGGAACATCATTTGAGCAAAGATATCAATGCTTCTCTGCAT</td></tr>
<tr><td>39727037|F|0-30:G>A-30:G>A</td><td>TGCAGATGTCCTGACAATTGCTGTAAGTCAGTGAATAGTAGTACCTATTTTGTTAC</td></tr>
<tr><td>46757663|F|0-34:T>C-34:T>C</td><td>TGCAGTTGGGGAGGAGAAAGAGTATACAAACCCCTAAGTATACTTGCTTGACCATGTCCTCGTAGCTAC</td></tr>
<tr><td>39723839|F|0-50:C>T-50:C>T</td><td>TGCAGTAGTCCTATTTTCCTTTTGTTTTTTTGACTAATTATTCCTTTTGACGCGTGAAACAGGTCCCAG</td></tr>
<tr><td>39725634|F|0-18:G>A-18:G>A</td><td>TGCAGAATTCGTCATAAGGACCCTAGATAGCTTCACGCAACCAAGCATCCAAATTAGTTAGCCTGTCTC</td></tr>
<tr><td>39714169|F|0-5:T>A-5:T>A</td><td>TGCAGTCATATACTAAGGATGGGGGCCTGGGGCTAGCCGATCATCAACACTCCTCAAGATGGGGTTAGA</td></tr>
<tr><td>39716107|F|0-21:G>T-21:G>T</td><td>TGCAGCGACACCAAGAAACAAGGTTGGGATGTGTTATAAGAGTTCAGATATTTGATTTATAGTGTTTGT</td></tr>
<tr><td>46762664|F|0-38:A>T-38:A>T</td><td>TGCAGAATTGGACGTGGAAGCGAATCGAGCCGAATAGAAGACATCCTATTCTTTATGAGCACATCCATG</td></tr>
<tr><td>39717735|F|0-38:A>G-38:A>G</td><td>TGCAGTAACAAACGGTCAGATTTTCTTCTTATTTACATACAAGAGTAATTTCAATTAGAGCTTGTCCGT</td></tr>
<tr><td>39719415|F|0-15:A>G-15:A>G</td><td>TGCAGAAGGAGGTGAACATACTGGTGTTAC</td></tr>
<tr><td>39714355|F|0-47:G>A-47:G>A</td><td>TGCAGAGAAGAAACGGTATGAAGGACACAGGACAATCTAACCAAAAGGAATTTCATCAAACATGGATGT</td></tr>
<tr><td>39714794|F|0-56:T>C-56:T>C</td><td>TGCAGATAGGGTTGTGGCATAGCATATTTGGGGGGTCGAATTGTAGTATTCTATACTACCTTTTAGGTT</td></tr>
<tr><td>39725050|F|0-6:A>C-6:A>C</td><td>TGCAGGAAAGTAAGGAATTCGATGCCTTGAAAGTCTTTTAC</td></tr>
<tr><td>39720450|F|0-30:T>C-30:T>C</td><td>TGCAGGGGAATCTGGAGCCCTGACATCAAATCTTCCACTATTAC</td></tr>
<tr><td>39719472|F|0-29:G>A-29:G>A</td><td>TGCAGAATGTTTGAGCAAATTGATATGATGATTATGTCTTCGAAATTGAAATTCTTAC</td></tr>
<tr><td>39726075|F|0-67:G>A-67:G>A</td><td>TGCAGCATACTGTGTAGTTGCAACTGAATCAGCTGGAAGACAAGTGCCTATTGCATTTTTGGAGCGTGT</td></tr>
<tr><td>39716732|F|0-9:T>G-9:T>G</td><td>TGCAGGAAATGTTGTAAACCTTCCAACACTTGAGATTTGGAGCACATTCTGCATCAATCAAAATTCTAA</td></tr>
<tr><td>39717313|F|0-51:G>T-51:G>T</td><td>TGCAGGGCCTTTCCATTCCTAATGGGAAATTCTCTGTAGAACAAAGAACTCGCACCACATCAGCCATGA</td></tr>
<tr><td>39722735|F|0-8:G>T-8:G>T</td><td>TGCAGCCTGATTGATCACTTGGCCAAATAAAGTGGGTCCACCCAATGAAACATTGTGAAGAGCATTTGC</td></tr>
<tr><td>46764988|F|0-40:A>G-40:A>G</td><td>TGCAGTCATATACAGCAAAGCTATCGGACTTCGGTTTGGCAAAATTGGGTCCTTCAGAAAGTAAATCTC</td></tr>
<tr><td>39717846|F|0-38:C>T-38:C>T</td><td>TGCAGTACGTCTGGTGCTTGATTTCGAACGTATCCCCGCAGCTCCGGTTCGACTCTCTCAAATCAAAAA</td></tr>
<tr><td>39721036|F|0-57:C>T-57:C>T</td><td>TGCAGCTGCACCGCTACTCGTATTCCTTGGCGTAACTGGAATATCCTACTTATCATTCCCGTTGAGTAA</td></tr>
<tr><td>39721711|F|0-66:G>A-66:G>A</td><td>TGCAGAGAAGCCTGAATGCACTATGGTACCAAAGAGCTTGTACTCTGGACGTGGATCCTGCATCATGAT</td></tr>
<tr><td>39717891|F|0-46:T>A-46:T>A</td><td>TGCAGTAGCATCTGATAATAAAGTTACGACGGGCAAAAATGAACCTTCTCGAAGAGTTGAGGAGGATGT</td></tr>
</table>

39718091|F|0-15:A>G-15:A>G	TGCAGTCCATGCAGCATGGAGATCCATCTGGAATTGGTTACGTTTATGCTTTCACTATATTTGCAGGAG
39729451|F|0-47:G>T-47:G>T	TGCAGGTCATCAAACCAAAGTGCCAAACCTTTTATCTTATGTTAGGCGAAACATGAAATTTAC
46755107|F|0-20:C>T-20:C>T	TGCAGCACAATGTTTTTGCTCCTCTTACCACGTGAATTTATTTACTATACCAGGTTATTGCGCAAAATG
39721413|F|0-39:C>T-39:C>T	TGCAGAAGGTGAAGCCACTGTTACAACAGGCAATGAACGCGCTTGCTCGAGAATGGTTTGATCAACGTA
39721279|F|0-29:C>A-29:C>A	TGCAGAACCATCTCTATATGATAAAAGAGCAGGATGCTTTCTTGCAGCAGATATGGAGGTAGTACTAAA
39715519|F|0-13:A>G-13:A>G	TGCAGCAGATGACAGCGGCGGCAGAGTCATTCCCGGCGTTTATAGCGACGAATCCGCCAGATCTTTCGA
39714023|F|0-42:A>T-42:A>T	TGCAGAATCTGTTTGAGAAACTATGGATTAGAATCAATAGCCAGTTTCTGAACCTGTAAACATCACCCC
39727921|F|0-66:T>G-66:T>G	TGCAGTTTGTCAATCTTGATTTGCTTGAAGTCAAGGTACTACAAATTACAAATTTTTCCGTTGGTTTGG
39720654|F|0-11:T>G-11:T>G	TGCAGTCACCTTGGATGTGCAAATTCAATGAAAAATTAGGCACTCAAGTTAC
39717465|F|0-33:A>C-33:A>C	TGCAGGTATAAAGCCGTTTCACTCTACTCTAGTACATATTTTTCAGAAGCGATAGCTTTTAC
39718864|F|0-27:C>A-27:C>A	TGCAGTTTCAGTACTGAATTGATAAGCCGCCATATTTTCTCACCTGATAAATTACCTGATCCACCTCCA
39725398|F|0-26:T>C-26:T>C	TGCAGTTGTTCTCTTTCTGATATGAGTTTTTCCTTCTCCTGGACTGTAGTTCTTAC
39728782|F|0-37:A>G-37:A>G	TGCAGTGAGTTTTCTCATAAATTTCTTTTTCCTTCATACATGTATTTTTCATAAAACTTTAC
39717571|F|0-41:A>G-41:A>G	TGCAGGTGGTTTGCAACCTGACGCAGAGTCAAACATGGTTCATTCATCATCTTCAGTATAAGTTTTGGG
39727078|F|0-15:G>C-15:G>C	TGCAGCAGTTGTACAGAATGCCTGTCCCCTGAAAGAAAAATTATTAC
39726169|F|0-27:G>T-27:G>T	TGCAGCCTCTGCAAGCACTCCTCCTAGGAAAGAACCAACATGTGCTGTGATTTTGAGATACAGATTCTA
46757397|F|0-63:C>T-63:C>T	TGCAGTGGTGTTTGGCTAAAACCACGATCGAAAATTGTATTCCTTATTACTTTTATTTGTAGGCCGCTT
39714277|F|0-55:T>C-55:T>C	TGCAGACTCATGAGCTTTTATTTGGCTGGTTGTTTTGTCATTGGATTTTTGTGAATTGAGGGAAGATAG
39717884|F|0-6:A>G-6:A>G	TGCAGTAGCAATCAGAGCTCGTATGTTCTCCTGTCTGTCAATGAATCCCATTTCTTGCAGCTGTGTGAG
46760880|F|0-21:T>C-21:T>C	TGCAGCCTCTGCTGCATTGGCTGTATTAC
39725626|F|0-50:A>C-50:A>C	TGCAGAATGCACCAGTTGTTTTTGGTCATCAGGTACTATTTGATTTTGAAATTTATAGTGTAAATATTA
46769166|F|0-41:C>T-41:C>T	TGCAGCGTCTCCGTCCAAACGCCGCCAATGGCGCCTCTGGCCTCGAAGACGATCGGAAGGAATCCTTTG
39721789|F|0-50:A>T-50:A>T	TGCAGAGCAGAGTGTGAAACAGAAGGCTGCTAGAAACAGAGGGAGACAGAAAACACAGAGTAATTGTCT
39715300|F|0-65:G>A-65:G>A	TGCAGCAACCTCTCAGCTACATCTATGAGCCACTTCAGAGCTCTAGGTAGAAGCTTCTTAGGGGAGCCT
39727144|F|0-30:C>A-30:C>A	TGCAGGAGCAGACTTCTCTAAATTTTCTTGCCTGCGACTTAC
39726188|F|0-20:G>C-20:G>C	TGCAGCGACTGTTGAAGCACGGTCGACCATTTTTGTCGATCATCTTTCATGGTTCTGCAACCTTGGAGC
39722461|F|0-57:T>G-57:T>G	TGCAGCAGGAAGCAGTTTTCTTGGTATGATGATGTCATGTGAATTATTGGTTTATATTTTCAGAAACTT
39723617|F|0-31:A>C-31:A>C	TGCAGGTGAGATGTTATTTGAATAACTTCACAACAAATCTATAATTAGGATATAAATTATTGATGTGTT
39714855|F|0-13:C>T-13:C>T	TGCAGATCAATAACCATATTCTACTTCTTGCCTATTCTTTTCTTTCACAAACATGATGAATCTCATATT

39726270|F|0-56:A>G-56:A>G	TGCAGCTCGCTTTGATGGCCATGTAGATGAATGTATCATGGGATTGGTTCGAGGCAAATTAC
46753808|F|0-49:C>T-49:C>T	TGCAGAACCTGGAGTAGGTGGTGTTCCCAATCGCTTTCTTGGAATCACACCTGCCTTTTTATGGCAAAC
39720693|F|0-9:C>T-9:C>T	TGCAGTCTCCCGGAAGTTTGGAAATTACAGGCAGCCGAGATCTTAC
39717181|F|0-40:A>G-40:A>G	TGCAGGCTAATGTTCGAGAAAGTTTGTGGCGTTCATTGGAAGAGGACATGATTCCATCGTTATCCTCTG
39719301|F|0-26:G>C-26:G>C	TGCAGAAAGAAAAGGAAGTTGAAGCTGTGTGTATGTCATGGAACTAAAGAATGCTGGTTAC
39715970|F|0-18:C>T-18:C>T	TGCAGCCGACTCGAAAACCGATCGGATCATGCTTCTCACTCTCGAATGGATATCCAACCACTTAC
39720917|F|0-16:T>C-16:T>C	TGCAGTTTTGGTGAAATCCTGTTGTTAC
46769166|F|0-20:G>A-20:G>A	TGCAGCGTCTCCGTCCAAACGCCGCCAATGGCGCCTCTGGCCTCGAAGACGATCGGAAGGAATCCTTTG
39715499|F|0-8:G>A-8:G>A	TGCAGCAGGGAAGATAGAAACCAAATCAGCCACCACCACATTACATCTCAAACGCCAAGCTTGAGGTCC
46756254|F|0-17:A>G-17:A>G	TGCAGGAGGGAAGAGGGAGCCGAGGAAATCGGGAAATCGCTGAAATAATGGAATCTCTGGAGCCTGAAA
39724610|F|0-5:C>A-5:C>A	TGCAGCAATGGAGTTCCCAGTTATCAACTTGGAGAATCTTAC
46774172|F|0-20:G>A-20:G>A	TGCAGGCAATTTCTCTTTACGGTTTAC
39728910|F|0-10:G>A-10:G>A	TGCAGGGGTAGTGCTAGTAGTGAATCAGATTTACTTTTTAC
39713785|F|0-41:A>T-41:A>T	TGCAGAAGACAGCTACATGGAACCTGTTCATCAGAAGCACGACCAAAATTCTTTATAACATTCCTGAAG
39714305|F|0-25:A>T-25:A>T	TGCAGACTTATCTAGAAAGAGGAATATATGGAGGTCAGAACAATAATTTTATGTTGTGTAGTTCCTCTT
39717804|F|0-46:A>G-46:A>G	TGCAGTAATTGCAACTCGTGCCTTTTGGCTATAACAGATTCACGCAACTTTGAAATACCATTCCACGCA
39725161|F|0-14:A>G-14:A>G	TGCAGGTACTTATCAATCGCCTTTCTTTAC
46755456|F|0-24:C>T-24:C>T	TGCAGCCAGTAAAGTTTCTCGACCCCCGTTGGATATTGACTTGAATGTTCCTGACGAAAGGATTCTTGA
39723620|F|0-34:T>G-34:T>G	TGCAGGTGATGTAAGCACGACTCCTGATATGCAATAACTGATACTAGGGTCAAGCACCGCCTGAGCAAG
39724693|F|0-32:G>A-32:G>A	TGCAGACACACGATTCGAGGATTCAGAACCCTGAAGGAGTGGCTCGGTTTGCTTAC
39715295|F|0-50:G>A-50:G>A	TGCAGCAACCAGCCAGTCCTCAGGGTGCTTTTCTTGTAGATATTTCTCTCGTTATCACTGCATTCACAT
39723417|F|0-32:A>G-32:A>G	TGCAGGCTTGGGCGGAGAATTTCAGATCCGCCACTGTCATCAGAGATCATATTCCCGATCACGATCAAA
39714756|F|0-48:G>A-48:G>A	TGCAGATAATGATGATGGTGCTTTTGATTATTCGAGATTTGGCGAGTCGAATGAGGATCAAACCATGGA
46765518|F|0-52:A>T-52:A>T	TGCAGTTTGAGCACTTTGACATCGTTGTACCCAAGAATGCAAAGAATTTTGAAGCCAATCTGAATTTAT
39726063|F|0-29:T>C-29:T>C	TGCAGCAGCTGCTGCAAAACCACTTCCACTTTCCCCGTCTCCAGTGAAGATAGGAGTACTGAAATCAAA
39718358|F|0-55:T>G-55:T>G	TGCAGTGCCTTTTGCATACTGGCAAGCAAATTTGAATGGCTTCTGTAAGTTATATTCCTTTTTTGCGAA
39717501|F|0-29:A>G-29:A>G	TGCAGGTCCCAGTTATTTCCCCAGCAGAGACTTCATTATTGGCTTCAAAGTATTACAATCAAGGATTTG
39720269|F|0-17:A>T-17:A>T	TGCAGGAAACAGCATCAATAGAGGTTGATAAAATTATGTTAC
39713328|F|0-48:T>C-48:T>C	TGCAGCAGTAGCTGAAGAAACAAAATTGCCTTTTGTGACAGCAGAAAATAAGTTTGAAGCCTTGGTTAG

46754079|F|0-5:G>A-5:G>A	TGCAGGATTATCATTTTGTCATTATTAGATCTTTTTTTCTTATCTGATTCTTATGGGTGGTATTTTCTG
39718541|F|0-48:T>C-48:T>C	TGCAGTTACAAGTTGTTGGAGATATCCTTTGTATGAAGGTAAGAAATCTGCTGTAAAGAAGAACTTGAA
39714842|F|0-58:T>A-58:T>A	TGCAGATATTTGAAAGTAAGGGAGAGTTGGGGGTGTTCTGTAACGTGAAAAAACATGTTACTAAACCGC
39715310|F|0-6:A>T-6:A>T	TGCAGCAACTCTGTGTATTGTCTCATCCTCAGTGCTTCTGAGTAGCATCAGCAAAGATGAAAGGCCCCC
39725797|F|0-8:A>G-8:A>G	TGCAGAGTAAGGCCCTCGTTCAGTTGTTCATCAAAAGATGTTATAGAATTTATTGGTGAGGATGGTTCG
39727249|F|0-18:A>G-18:A>G	TGCAGTGTGGGGAGCATTACTTAGTGCTTGTTGCATCCACCATGAAGTTAC
39721859|F|0-57:C>T-57:C>T	TGCAGAGGATTGCAGGTCAGATGCCTCCCTAAGAGCCTGTTCGCAGAACGATACTAGCCTGTTTTCAGC
39720420|F|0-35:T>A-35:T>A	TGCAGGCTGACAAAAGGACAGGGACACATAAAAATTTTAC
39724211|F|0-18:T>C-18:T>C	TGCAGTTAGAGTCGAAACTGGGCTCTTGTCGTGATCCATGAGCTTCGCCAATCCAAAATCGCCCAATCT
39717888|F|0-25:T>C-25:T>C	TGCAGTAGCAGAGACAAATAGGCCTTCTTCAAGATCATCAAGGAGAGGGCCTGGGCAATTACAGTTGTG
39720082|F|0-5:G>C-5:G>C	TGCAGGCTTGAGATATACCACAACCAAGAGTTAC
39725269|F|0-36:C>A-36:C>A	TGCAGTCCACTTTATCCGCTATAAACAGAGAGCAAACCCTACAAATGCCTTACTTTTAC
39714557|F|0-23:A>G-23:A>G	TGCAGAGGACAGATGGCAGGTACAAACCAACAATAAAATCACTTGATTCTGGATTGCGCTTTTTTTCTA
39717214|F|0-19:T>A-19:T>A	TGCAGGCTGTTTCCCTCATTTCTTCATATCTAAAAGTTCCTTTACGATACCCTATACGCTTGGGTGGAT
39723687|F|0-48:T>A-48:T>A	TGCAGGTTGTGATGTTGGATGAGAATGGAAAGAGATATCCTCCGAGCTGATGGCTGAGCTGGTGGCAG
39724899|F|0-30:A>G-30:A>G	TGCAGCAGAAAATAAAAGGACAATCCCCACAGTTCATTTGAAGGCAAAGTTAC
39713331|F|0-40:A>T-40:A>T	TGCAGCCTAACATTGGTGAAATTTTGCAACAATGTTTCGGAGGCACATTTTAGAAACTGGGAATTCTTG
39718240|F|0-27:C>T-27:C>T	TGCAGTGAAATTCTTTTACACTGTTATCATCTCATTGATGCTTGGAACAATATGTTGGAGATTTGGTGC
39720523|F|0-9:T>A-9:T>A	TGCAGGTGTTGGCACATATCCTTGCTCGATCCAAGTACTTAC
46765438|F|0-54:G>A-54:G>A	TGCAGTTGGAAGATGACAAAAAAATGTAATAGATTACAGAAAGCGTATTATTGCGAGGAATGGGTTATC
39715528|F|0-52:C>T-52:C>T	TGCAGCAGCAACAAAAGCATTCACTCGATCTTGAACATTGTAGTCAAACAAGCCAATATTATCCTTCGT
39726055|F|0-61:G>A-61:G>A	TGCAGCAGCCTTCAAATTTGGAAAAGAAATAAAACAGTCAGAAAACTAAACAAAACTCGGAGTAGTTAT
39728482|F|0-27:A>T-27:A>T	TGCAGGTGTATTTGCTTATAGAGGAAAACTCTCCTGTTATATTAC
39728949|F|0-63:C>T-63:C>T	TGCAGGATAAGAAAAACGACGACAAAGCACCAAAGGAAGATGAGAAATGAGGCATTTTGGAGCCCGGTT
39721911|F|0-63:A>G-63:A>G	TGCAGAGTCTTCCACTTGTAAGAAATTTTCGAGATTGATGCTATTGTTCGGAGTTTTCCCTCCAACATT
39725418|F|0-51:A>G-51:A>G	TGCAGTTTTCTTTTACTTTCTTGTTCGGTTCTTTGGAGAAGAGAAGAAAGGAAATTTTTAC
39727188|F|0-31:A>G-31:A>G	TGCAGTAAACAGCATAACTGAGCTGATACATATTAC
39719559|F|0-21:T>C-21:T>C	TGCAGAGAAACCGTTGAGCGTTGCAAAATTGCGAGAGATGAGTGGTAGTGACATATTTAC
39720633|F|0-16:G>A-16:G>A	TGCAGTATTCTCATCAGGGCATAAAGCTTTAGCTAATTGAAAGAGTGTTAC

39726596|F|0-10:T>G-10:T>G	TGCAGTACGCTCTTTCGCATGCCGTCATTGATAACGACGAGGTCGTTCTCCTTCATGTCGATAACCCTA
39727799|F|0-54:T>A-54:T>A	TGCAGTATCTTATCATCCTGATGGACAGGTGTGTGCTTGTCTGACTCTTTCATTTCGTAGAAGTAGAAA
39718954|F|0-17:G>A-17:G>A	TGCAGTTTTCTCTGACCGTGCATCTTTCCTTCCATAGACTACCATTTACACTGCTAGTGTTGCAAAAGG
39727218|F|0-21:C>T-21:C>T	TGCAGTCAAAACATAAATCTACAAGAATTAGTTTTAGTTTAC
39717274|F|0-67:C>G-67:C>G	TGCAGGGAGATTCAAAAGTTTTGAACTTGAGCCAGAACCCGGCGAGGTTTTCACCGGAAAACATGTCCG
39725453|F|0-49:T>C-49:T>C	TGCAGCATATTTGCCCCGATTTTGTACTTATACAAGAAACCAAGAAGACTTCCTTTGATAGGAGATTTA
39713788|F|0-10:T>C-10:T>C	TGCAGAAGACTGCGTTCGGGTCTTTCTCGAACAGCAGCACAGGGAGCAGCCCAAGAACAGAGAACAACA
39718069|F|0-50:T>G-50:T>G	TGCAGTCATTGGTGCCTACCCGGAGTGCCTGATGTGTGGAATGAAATTTTTCTGTCCTACCTGCTTACT
46755075|F|0-49:C>T-49:C>T	TGCAGCAATATCCTCTGTGGCAATGCAAGCCATCGGTCATATAGGATTGCGTGTACCATTGCCACCACT
39729408|F|0-32:C>T-32:C>T	TGCAGATGATGAAGAGTAAATATTTTGAACGCCTTTTTGAGTTTTGATTGATATGGCTGCTTTAC
39713787|F|0-19:C>T-19:C>T	TGCAGAAGACCCAAGCAGTCTTATTTTTTGATGTGATAGTTCCAACTTGGTAGTCCCAAACAGAAATGG
39715755|F|0-13:T>C-13:T>C	TGCAGCATTATATTCTGGCACACGCAAGCAAAACATTTTCCAAGTAGTACAAAAACCTTCTTATATATG
39723619|F|0-31:A>G-31:A>G	TGCAGGTGATATGCTCAATGTGTTCATACTTATCTTTAGTATCTGTGTTCAGTGCATTGATTGAAAATG
39722834|F|0-28:C>G-28:C>G	TGCAGCTAAATTCGTAGTCAGCGGCGGCCCCGTGGTGGGGGTGGTGGCTCCGGCGAAAAGGGGCGAGGG
39716710|F|0-6:A>C-6:A>C	TGCAGGAAAAGGCATTGTTCACTCTGAAATGCCTGCATCCCATGGCACCCAACATGGCTTACAACTTTG
39715032|F|0-55:C>G-55:C>G	TGCAGATGGGTGGAAATTCACAATAGAGAAAATGGGACCCCGAGGGAAACGGGGACGTGGTGGTGGATG
39720139|F|0-18:T>G-18:T>G	TGCAGCTATATAACACGCTAAGAACGGTGTCGTTTAC
46756600|F|0-44:G>A-44:G>A	TGCAGGGGAGAAGGAGATGGAACCGAAAACTTGATTGTGAAGCCGAATTATCAAGTCAGGCTCGAAATC
39715683|F|0-36:T>C-36:T>C	TGCAGCATCAACAGCATGCCCTCCTCGCCTAAGCATTGACGCTCCAATTTCGGAGCAGCGGCCATCATC
39725587|F|0-57:T>C-57:T>C	TGCAGAAGCAACTACAAAAGATTTACCACCGTTGATACTTGAAGATGTTCCATCAAGTTTCAGCAATTC
39724574|F|0-7:T>A-7:T>A	TGCAGAATAATTGTCGATGTCACACTTTCTGTTTTGGATGCTTGTACTTTACTTAC
39720009|F|0-17:A>C-17:A>C	TGCAGCATTTGCGCCAAAATAGTTAC
39728482|F|0-35:C>G-35:C>G	TGCAGGTGTATTTGCTTATAGAGGAAAACTCTCCTCTTATATTAC
46756797|F|0-29:T>C-29:T>C	TGCAGGTGGGTGGATGAAGTGATTCCTGATGCACCCTGGGTGATCTCTCAAGAATTTATTGACAAGCAC
39718675|F|0-13:G>T-13:G>T	TGCAGTTCTCCTTGTACAGTTATGTCTTCCATGATGTAGTAATAATAACAAGTAATTTAGAAAGAAAGC
46768604|F|0-65:G>C-65:G>C	TGCAGAGGTTGAAAAAGCTAAAGAATTCACAAGAGTGAAGAATAAACGAGGTATGCTCTGAAATTGGGT
39714628|F|0-39:C>G-39:C>G	TGCAGAGGTTCACAGACGACTAAGTACTTTTGAATCTTTCTTTTGCTATGATAAGGCTATCCCAGAGGA
39724380|F|0-36:A>G-36:A>G	TGCAGTTGTTATGTTTCCTTTTTCAAAGGAGACAACATCATGCTTGTGTTGATCACTATCCTCCCCATT
39725506|F|0-33:G>T-33:G>T	TGCAGAAAATACAGAAAAATATGAAAAACAAAAGTATTAGTACAACGTAAAAAATAGTGTATGCGTTAC

39716315|F|0-55:C>G-55:C>G	TGCAGCTCACAGGCGATGGCAGCATCAAATATGTTCAGGTTTTTCTTCTCTCTATCATGCTTGTTTGGG
39715407|F|0-9:A>G-9:A>G	TGCAGCACAAACGGTTATCATCTATGTGAGTAGCCTGGAGACCAATCATCCATGATCCCACTGATACAT
39716178|F|0-19:C>T-19:C>T	TGCAGCGGCGGCGGCGAAGCAGAGAAGGAGAAGAAGATTGAGTAAACGAGGAGTCTCCATTGTCTTCGT
39728907|F|0-6:A>G-6:A>G	TGCAGAAATTGAGAAGTCTACCCAAATTGATAGGGACTTCACCTAATTTTAC
46753583|F|0-62:A>T-62:A>T	TGCAGAAAAGGAAAAGAGGCGAACGAGGTTGATGGCTAAAATGGAGGAAATTGGTCTAACGTACAACAG
39721096|F|0-29:A>T-29:A>T	TGCAGAAAAACAATGGTAATGTGGAGAAAACCCTTGCTACTGTGAAGACCAAGTTGGATTCTAGGTGTG
39714386|F|0-9:T>A-9:T>A	TGCAGAGACTCCTACTTCAATGACACTATTGGATTTCATGAACTGGGGTCCTGATATTTCTGATAATGA
39716316|F|0-24:A>G-24:A>G	TGCAGCTCACAGTTAGTCATAGTAATTTCAAGGAATACCTCTCTCGACATTGTTTCCCTATGCGACCTG
46757725|F|0-59:C>A-59:C>A	TGCAGTTTCCTCCACCAAAAGCTTCTTCTCAATGGTTATATTTCCTTACTGGCTCCTTCCACATAAACA
39718454|F|0-6:G>T-6:G>T	TGCAGTGGTCATCTCTGCAATACCAAGGGCAATGGCTTCACCCTTGGTGGTCATAAGTACAACTTCTTC
39724223|F|0-20:A>T-20:A>T	TGCAGTTATCATCTCAAATGATATGGAAAATGACCTCCTAATCACCATTAGTACAGTGCTAAATACAAC
39719935|F|0-11:A>G-11:A>G	TGCAGCAGAGAAAGGTCCTCGGGTTAC
39724432|F|0-63:A>T-63:A>T	TGCAGTTTCTTCAATTCAAGAGCCTGAGGCATTGAAATCAAACAGAACAATTTAGAACAAACAAGCTAA
39728653|F|0-18:C>T-18:C>T	TGCAGAAGTTACCAGTAGCAGCAGCAGTAATTTTATTTAC
39726538|F|0-42:C>T-42:C>T	TGCAGGTGCAGGAAATACCTGTTCCAATCAACTTGTGTTACACATATTGGAAATAGAACTACAAAACAA
39717065|F|0-16:T>G-16:T>G	TGCAGGCACTACTTCATTCAGTCCCATTTGGGAAAATGATTGTTCTTGATCTTTTTGCGGACGTCAAGC
39722352|F|0-7:T>C-7:T>C	TGCAGCATCATGAACAACCAGAGGTCATTCATAACAGATCTCACTCGTTTGTGGGTCCTTCTCCCCGGT
39714200|F|0-62:G>C-62:G>C	TGCAGACCCATTACAAGAGAAAGCATGGGGCAAAGCCCTTCAGTTGTAGGAAATGTGGGAAGGCCTTTG
39713810|F|0-62:G>C-62:G>C	TGCAGAAGATACCATACTTCTCGATAGAATACTGTCAAAAAAGGTTTGACAGAAAAATTCCAGCACCCT
39722611|F|0-18:T>G-18:T>G	TGCAGCCAGAAGTTGCCCTATTCTTGGCTGAAATCTCACCTGAGTCGAGCCAGAGCCACCCTGTTCACA
39722021|F|0-64:C>T-64:C>T	TGCAGATCCTGAAGCGCGGGATCTCTCCTTCACCAATGGCTGTGGTTCTGATAGGAAAAAGTATCGTAA
46765013|F|0-62:T>C-62:T>C	TGCAGTCCAGATACGAAGTCATGCACAGAAGTTCTTTTCAAAGGTCAATGGGTACCCTTCTCTACTTAT
39729413|F|0-26:A>T-26:A>T	TGCAGCCTGGTTTTGTGGGCTAAAATAACCGATGAATTATGTGGTAAAGTCAGGGATGCTGATTACAGA
39725011|F|0-17:A>G-17:A>G	TGCAGCTGAAACAAGTAATAGCAATGTGCTTGAAATAGGAATGTTAC
46762606|F|0-51:T>G-51:T>G	TGCAGAAGTTGGTATTCCTAGTGACAGACGTGAGTCTACTCATGATGTGCATGTAAATCAATCAGACAC
39718881|F|0-65:T>C-65:T>C	TGCAGTTTCTAACCAGCAACCATCCAAAAGCAGCCCATCCATGTAGTCCAAATCCATGGCAGTATTAGC
39717683|F|0-34:G>A-34:G>A	TGCAGGTTTTGAGTGGAGAGAAAGAACTGTCAGAGCTCGCAGAAATTTCAGGATTCGAGCCCGATGGGA
39728013|F|0-29:C>A-29:C>A	TGCAGCATTTCAAATCTAGGGATGTTCCCCGTGGATCATTTCTGTGCTATAATTAC
39727516|F|0-34:T>C-34:T>C	TGCAGCAACCATTCTTTGTGGGGCCTCCTTGATCTGAGGTTGGAATATTCAGGTGACGATTGAAAGAGA

<table>
<tr><td>39724843|F|0-31:A>G-31:A>G</td><td>TGCAGATTGATCTTTGAAGGAGTAGAGAGCAATTTGAATTAGTTAC</td></tr>
<tr><td>39714417|F|0-37:T>A-37:T>A</td><td>TGCAGAGAGAGCAACGGTTTCTTTCTTCTTTAGCTTCTCCGTATGTAGTTTCTTATAGAGGCTGTGAGG</td></tr>
<tr><td>39722081|F|0-27:C>T-27:C>T</td><td>TGCAGATGCCAAGTCCTTGCCAAAAGGCTTCTCCACAACAATCCGGGTCCATCCACCAAGATCAGCTAA</td></tr>
<tr><td>46774192|F|0-24:A>G-24:A>G</td><td>TGCAGGTGACCAATTAGGATGCAGAGATTTAC</td></tr>
<tr><td>39723279|F|0-33:T>C-33:T>C</td><td>TGCAGGCAAACCTTCTGGAATTGCAGCTACAGCTAAAGCCACCGCAATCTCAAAGTAATATGTACATTT</td></tr>
<tr><td>39719288|F|0-19:G>A-19:G>A</td><td>TGCAGAAACAATTGGACCTGGGATATTAC</td></tr>
<tr><td>39727097|F|0-6:T>G-6:T>G</td><td>TGCAGCTATGAATCTGTCATCTCTTGGTGAAGCTAGCTTAC</td></tr>
<tr><td>39725054|F|0-15:T>C-15:T>C</td><td>TGCAGGAACAAGTAATAAAACTGTCAGACTCCCCAAAAACTTCACTCTTAC</td></tr>
<tr><td>39713859|F|0-7:G>T-7:G>T</td><td>TGCAGAAGCTTTATTTTTCCTCCTCCTTGTTATATGTTACCTAAATGGAGTCACTAAACCTATCCTTGT</td></tr>
<tr><td>39721275|F|0-58:A>C-58:A>C</td><td>TGCAGAACCACATGCTGGAAACTTGGCTGCTAATTGAAAGTTTTAGGGAATGTCCTGGAAAATATTTGG</td></tr>
<tr><td>39728470|F|0-26:T>A-26:T>A</td><td>TGCAGGCAGTTGAAAGTTGAAAAGTATGTTTTAC</td></tr>
<tr><td>39723757|F|0-62:C>A-62:C>A</td><td>TGCAGTAAGTGCAACCATATAATATCATGAAAATGTAATATATTGTCTACACAACACATTAGCAGGCAA</td></tr>
<tr><td>39716056|F|0-54:G>A-54:G>A</td><td>TGCAGCCTGATCTTGTTGCTCTCTAGTGACACCAAAACGATGGGCAACATTTTCGGAGGTTATACCCAT</td></tr>
<tr><td>39716848|F|0-43:C>T-43:C>T</td><td>TGCAGGACCTTCTGAGGCAGGTGTTTACGCCTATACCAGGGTACGCAACTTTAGCCTTCTCCTTTGTTC</td></tr>
<tr><td>39715659|F|0-5:G>C-5:G>C</td><td>TGCAGGATACCTGATTTTGTTCAAAATAGTTAGGCAATACGTTGTGCTCCCCAAGCAGAACTTCACCTC</td></tr>
<tr><td>39723478|F|0-60:C>A-60:C>A</td><td>TGCAGGGCTGGGAAACTGATTGCCATCCACATTCTGGCTATGAACAAGCTGTTCCCAGCTCAATCCCAC</td></tr>
<tr><td>39727845|F|0-26:C>T-26:C>T</td><td>TGCAGTGAGTCTGTGTTCAATCGAATCCGCGCTTCTGTGCTAAAGCTAAACCCACATAGAAATGACGGA</td></tr>
<tr><td>39715223|F|0-11:G>A-11:G>A</td><td>TGCAGCAAAACGAGAATCTCGGGTCCTCTGATCCAGGTCACCAGCATTGTCCATCTGAGAGCAGACAAC</td></tr>
<tr><td>39719290|F|0-22:T>G-22:T>G</td><td>TGCAGAAACAGTTACAATTTGGTTGGTTCTGCCAGGATGAGCTTTTAC</td></tr>
<tr><td>39720847|F|0-12:T>C-12:T>C</td><td>TGCAGTTGAATGTACTTCAACGAATAATCCCTTTTAC</td></tr>
<tr><td>39725530|F|0-44:A>G-44:A>G</td><td>TGCAGAAAGCTGTGGCTCGGCACCATCCCGTATTTGGCCAATCCATGCTTCTTCGTCACGTGGGTTTCC</td></tr>
<tr><td>39715335|F|0-40:A>G-40:A>G</td><td>TGCAGCAAGATTTTCAGCATTTTGATGAATCAGTAACCATATGAGAAATGAGATTGTCAAACGAAATGA</td></tr>
<tr><td>39726096|F|0-24:G>A-24:G>A</td><td>TGCAGCATTTTCTTCCTCCTCACCGGACATGTTTGAGAAGTTGAAGTCTTGGACAACCTAAAGAATCTG</td></tr>
<tr><td>46757321|F|0-36:C>G-36:C>G</td><td>TGCAGTGCCTCTGAACGAGAGCTAATCTGCCTCCTTCCAGGCCGAGCACTTCCAAAATCCATCAATATA</td></tr>
<tr><td>39723599|F|0-22:C>T-22:C>T</td><td>TGCAGGTCCTTTATATCAACCCCAGCAGCATCGGCCCAACAACTCATGCTTGCTATTCTTCCGGATTCC</td></tr>
<tr><td>39721039|F|0-36:A>G-36:A>G</td><td>TGCAGAAATGGCATCCTGATAAGCAGAAAGATCAGGATGTTGCCACTTCCAGATTTCAGGAAATAAACG</td></tr>
<tr><td>39717165|F|0-6:T>C-6:T>C</td><td>TGCAGGTGGAATAACTCCAGAGAGTGTCATATACAGCTTTGGAACTCTTTTGCTTGACCTTCTAAGTGG</td></tr>
<tr><td>39719639|F|0-6:G>A-6:G>A</td><td>TGCAGAGGTTTTTTCTTACCTGTCAGACCAATTTCCCTTTCAAGATCAGGGGGAGTTAC</td></tr>
<tr><td>46755282|F|0-12:A>C-12:A>C</td><td>TGCAGCAGTTGCAAATTGTTGAGAGCCACCAGATTGCTGCCATTGGTGGCCTTCACTCAAACACAGTGA</td></tr>
</table>

<table>
<tr><td>46760713|F|0-6:A>T-6:A>T</td><td>TGCAGAACATATACGAGCTCCTGGTTAC</td></tr>
<tr><td>39715946|F|0-8:T>G-8:T>G</td><td>TGCAGCCCTTACAGTGCAGTTGTTCTTGTCTCACATCCTCAACAAGGAAAGTTGACGATTAC</td></tr>
<tr><td>39716354|F|0-63:A>C-63:A>C</td><td>TGCAGCTCGGAAGACACCCTTTGCACCGAACGGGAAAAAGGGCACCAAATTTGCAGAATTCCCACGCAC</td></tr>
<tr><td>39718849|F|0-25:G>A-25:G>A</td><td>TGCAGTTTATGGCAGTCAACGCTATGTACGGTTTTCACCAGAAGTGCTTTCTTCTGCTCAGGTCTCTTC</td></tr>
<tr><td>39726385|F|0-32:C>T-32:C>T</td><td>TGCAGGAGATTCTAGGCAACAATATTCTTCCCCTGTGTCGAGGTGATAATTACAAGTTCCATGCTGCTG</td></tr>
<tr><td>39725920|F|0-13:G>A-13:G>A</td><td>TGCAGATTGCTCGGGGGGCTGCGGGGGGCCTGGCTTATTTGCATCAATCCTGTGAACCCCATATCCTCC</td></tr>
<tr><td>39717572|F|0-45:T>A-45:T>A</td><td>TGCAGGTGTAACCTTGTCGATCTAATGGTGGAAATGGACCGCATCTTACGTCCTGAAGGAACAATAGTG</td></tr>
<tr><td>39725296|F|0-16:T>A-16:T>A</td><td>TGCAGTGACAGGCCAATCGACATGAACTTTTGTTTAC</td></tr>
<tr><td>46762815|F|0-64:T>C-64:T>C</td><td>TGCAGACTTTCTTGTTCAGCTCGAAAACAGAAGATCCATCAAGGTTTGTAATGCCACTGTACTATTGAA</td></tr>
<tr><td>39728826|F|0-45:C>G-45:C>G</td><td>TGCAGGAAAACAAAATGCAGACAAATCCTTCGTACGCATTGGCAGCCTCTTCCCAGGTGGATACTTACA</td></tr>
<tr><td>39729095|F|0-20:A>G-20:A>G</td><td>TGCAGCTTGATTGCAGTCTCAGGGCCATGGCCAAACACATAGACCTCATGGGAGTAAAACATCATTACA</td></tr>
<tr><td>39725354|F|0-17:T>A-17:T>A</td><td>TGCAGTTAGATTTTGATTGTGCTTCACCACCTGAACAAGCCTGGAGAAGGAAATTAC</td></tr>
<tr><td>39719316|F|0-16:C>T-16:C>T</td><td>TGCAGAAAGGAGAAGTCGATGCCATTAC</td></tr>
<tr><td>39718056|F|0-33:C>T-33:C>T</td><td>TGCAGTCATCAAGCATATGTCCCCTGATATAGCCAAGATCCGCAAGCTTGTCAGGCAGTCTAAATGTTT</td></tr>
<tr><td>39726637|F|0-68:T>G-68:T>G</td><td>TGCAGTCAAATCAAGTAGTTTTATATAAAATGAATCTGGCTGCCAGTAGAGAGAGAGAGAGAGAGAGAT</td></tr>
<tr><td>39716662|F|0-39:A>G-39:A>G</td><td>TGCAGCTTTCTCATCAGATGCATCTGTTTTCTGTAACCTATCTTGAGTGGATGATGCAACAGAAGTTTC</td></tr>
<tr><td>39718838|F|0-24:G>A-24:G>A</td><td>TGCAGTTTAGATACAAGAAACACAGATGGGCTATTGCTCCTAAATCAAAAAATGAAGTCTACCTTTCTG</td></tr>
<tr><td>39726238|F|0-29:C>G-29:C>G</td><td>TGCAGCTACTATTCTATGGCGTTTATGGCCTCAGCTGTAGTTTTATTGATCTGGGCTTTGTTTCAGACA</td></tr>
<tr><td>39728106|F|0-9:A>G-9:A>G</td><td>TGCAGTTGAATTCAAAACCAGTAATTTTGATTAC</td></tr>
<tr><td>39719539|F|0-23:T>G-23:T>G</td><td>TGCAGACTATTCAATCACACGTATAACATAACCCAAGGGTTTAC</td></tr>
<tr><td>39728284|F|0-33:A>G-33:A>G</td><td>TGCAGCTATTCGTCCCGAGCTCCGGCCGCCACCAATCTTCCCGAAAGATGTCATCGACGACAACCCTTT</td></tr>
<tr><td>39717791|F|0-63:A>G-63:A>G</td><td>TGCAGTAATCCTATAGATTTCATGTCCGAGACTAGCATTCCAGCATCGTATAAACCTGAAAGTAGTAGA</td></tr>
<tr><td>39726965|F|0-33:G>A-33:G>A</td><td>TGCAGAATACTCTGAAGAATGAAGATTTCCGTAGACCCGAACACTTAC</td></tr>
<tr><td>39720107|F|0-13:C>T-13:C>T</td><td>TGCAGCGGTGATGCTGTCGTTAC</td></tr>
<tr><td>39718308|F|0-17:G>T-17:G>T</td><td>TGCAGTGATGACACCAGGAGGTCCTCTGGATCTCTCTTCTGTGTTATTTAGGAATCGTATCATATTTAT</td></tr>
<tr><td>39721231|F|0-35:T>C-35:T>C</td><td>TGCAGAAATGAAATCAACATTTTCTAATATAAACCTTGTGAGCATCTACTTCATTGTCAGTATAATGAT</td></tr>
<tr><td>39717202|F|0-32:C>T-32:C>T</td><td>TGCAGGCTGCTGCCTTGGCTCTTAGGAAGGTCCCTCAATGCAACAGTTCATGGACGGATAATTATATTA</td></tr>
<tr><td>39721389|F|0-11:A>G-11:A>G</td><td>TGCAGAAGCTGAAGGAAGAGAATGGGAAGATGATGATGACTTTTCCAGTAATTTTTTAGCAGAAATCAT</td></tr>
<tr><td>39714886|F|0-15:A>T-15:A>T</td><td>TGCAGATCCCATAAAACCACAGCTAATAAATGGTATTATGAGACAACCAAAAGGCCAGCAAAAGAGTCA</td></tr>
</table>

39728122|F|0-16:G>C-16:G>C	TGCAGAAAATCATCGAGTGTATCGAAGCTTGAAAGCTTATACAGAGTTACCAAGGTTTTGGCAGGGCAA
46755320|F|0-62:G>A-62:G>A	TGCAGCATCACGAGTGAGCAGCAGTCCAGTCAACCAAGTAGTGTGGAGAGTGAGAGACGCCTGTCTGAT
39714267|F|0-8:G>A-8:G>A	TGCAGACTGGTGGAATCTTACATTAGAATAGCCTTGCAAATTGCAAGTATATCAATTACCATGATCATA
39726805|F|0-45:C>T-45:C>T	TGCAGTTCTTTCTTGGTGACTATCGCCCGCTTATTCAGGATCCTTCCGGCAATGTAGTTGACCAGGTAG
39715628|F|0-39:C>T-39:C>T	TGCAGCAGTCGTATCTTTTGTTCTTGCGTGGTATGATGGCGAGGAAGGAGGTGAGATGGAGATTACAGC
39725679|F|0-44:G>A-44:G>A	TGCAGACTCGTAGCCAACTAGCCACAAAGTCTTTATTGATATTCGAGAGTAAGCGGATACAGAAGATCT
39727181|F|0-19:A>G-19:A>G	TGCAGGTTCGCCGTGCATGAATATTTAC
39713454|F|0-43:T>A-43:T>A	TGCAGAAAATATACCAAATTAGTGAATAAACCAAGCAAACAACTAACTAATAGTGTGTTTAC
39723003|F|0-48:G>A-48:G>A	TGCAGCTTATAATATAATCCATTTTTGAAGAGTTCAATCTCATCCTCTGTGGTGAATATACATGTCCAT
39727515|F|0-56:T>C-56:T>C	TGCAGCAACCATTCTTTGTGGGGCCTCCTTGATCCGAGGTTGGAATATTCAGGTGATGATTGAAAGAGA
39715412|F|0-11:G>A-11:G>A	TGCAGCACAACGCTTTGCTATTTTAGTTGAGATTCGAAGCTCTGTTGGCCTGGCCTCGCAAACAGGACG
39720384|F|0-22:G>A-22:G>A	TGCAGGCAGCCTCAATTGAAAAGCATCTTGGTGTTATCAAGCGGATCTCTTAC
39728059|F|0-10:A>G-10:A>G	TGCAGGCTGTACATGCTCAAGTTCTGTTGGATTCTTTAC
39720437|F|0-18:C>T-18:C>T	TGCAGGGATACTTCAAGACTACAATTATTGTTCTATATTTAGATTAC
39713795|F|0-30:C>A-30:C>A	TGCAGAAGAGATCATTTACGGGCGAGACACCTCAAAGGCATCGGTTAGCTATCTTGCCGATGCTTCATG
39718518|F|0-68:T>A-68:T>A	TGCAGTGTTGCAGTCAGTCAGTTGAGCAAGAATTGATTCAGATATCAGGGATAACAGCCAGGGATACTT
39723122|F|0-19:C>T-19:C>T	TGCAGGAAAGTCAAAACACCACCACACCATGCAACCTACATTTTGAAGCATGTGTTAGCAGGGAAGTTT
39717249|F|0-41:C>A-41:C>A	TGCAGGGAACTGGAGGTGTCAGTATCTATGGTTCCAGTTTCCGAGACGAGAATTTTTCATGTATGTCAT
39719560|F|0-12:T>C-12:T>C	TGCAGAGAAAGTTCACTGACATGCTCCTTATCCCCTCTGTTTGCAGTTTATGTTTAC
39727175|F|0-31:A>T-31:A>T	TGCAGGTCATAATGTAGGCTACTGATGTTGCAGAAGCAAACCCATTACAGAATATTAC
46773627|F|0-52:C>T-52:C>T	TGCAGCTGCTCATTTCTTCCCCACTCAAAGGAATTTCAACTTCTTTTGGTGACTGATTATCACTTTTAC
39727471|F|0-10:T>A-10:T>A	TGCAGATATCTTCTCTTCATCTGATCCTTTTACTGCTACCTCAAATATTTTCTCCAGTTCTGCTAAGCT
39724063|F|0-7:A>T-7:A>T	TGCAGTGATTGCTGGAAGTGAGTACGTGAGATTAGATCGTGAACATGAGCAGAGGCAGCCTTTTCCAAA
39725022|F|0-15:G>C-15:G>C	TGCAGCTGGCGGTGCGCCTGCCGGATACATTGATCCGGTTAC
46757112|F|0-16:C>T-16:C>T	TGCAGTCACCACCTCTCTCTTCGACAATCTAGGGTTTCATCGAGACGCCTCGTTTAGCTTCAAATTGTT
39721490|F|0-57:C>G-57:C>G	TGCAGAATCTCATCATCATTTGGATGGAAGTGTATCTTCCCACTTCTATAGATACCACAAACAACTGCC
46757326|F|0-16:T>A-16:T>A	TGCAGTGCGAGCTGAATTGAGGCATGGCAACAAGAAAGAATCTGATATTTTGATGGCCAAGGCACTGCA
39715574|F|0-35:C>A-35:C>A	TGCAGCAGCTGAGATAATCATGATAGGGTTTTTGGCTATGGAAATGGAATTGTAATGAATTATAACCCA
39722458|F|0-58:T>G-58:T>G	TGCAGCAGCTTGCCCGACCCATGTACAGCAATCCACCTGTTCATGGTGCACTGATAGTTTCAACCATCC

<table>
<tr><td>39729565|F|0-19:G>A-19:G>A</td><td>TGCAGAATAGAATTTCAATGCTATCTTAGAATTTCCAGCACCTAATCCATTGGGTAATCTAATTTACAG</td></tr>
<tr><td>46762396|F|0-24:C>T-24:C>T</td><td>TGCAGAAAGAGTGATAATTTTATGCATAAATGAACCAGCAAAACAAATTATTCCAAGCACAAAAAGTAA</td></tr>
<tr><td>39716126|F|0-48:C>G-48:C>G</td><td>TGCAGCGATCTGGATCTGATATCAATCAAACATCTGACGAACAAAGCTCGCGGTATTGTCGGTATCGCA</td></tr>
<tr><td>39722520|F|0-24:C>T-24:C>T</td><td>TGCAGCATCATAATATGTATGAAACGCCTTTCTGAAGAATTTCTTTGGTCGGCAGTAATATTGAGGCT</td></tr>
<tr><td>39725246|F|0-8:A>G-8:A>G</td><td>TGCAGTAGATTTGCAAAGTTGAACCTTAC</td></tr>
<tr><td>39717662|F|0-55:G>A-55:G>A</td><td>TGCAGGTTTACGAACAATTGAAAATTGAGCCAGGAATGAAAAATGAAAGAAAACTGCTGGTCTTACATT</td></tr>
<tr><td>39714404|F|0-64:C>T-64:C>T</td><td>TGCAGAGAGAAATGCAATGGGAGGATTGTCGATTGATGATTTTTATCACTGGTATGTTTGTAACCTGA</td></tr>
<tr><td>39716391|F|0-23:A>G-23:A>G</td><td>TGCAGCTGAAGAATCCCCCTGCTACATCCAGAAAATTTGACAATATTATGGAAAGGAAACCATAATATA</td></tr>
<tr><td>46754083|F|0-42:C>T-42:C>T</td><td>TGCAGAATTCAGAATGCCATAGCACCATTTACAAAGAAGAAACTTTTTGGGTCTTCCAAAAGAATGTTG</td></tr>
<tr><td>39720512|F|0-7:T>G-7:T>G</td><td>TGCAGGTTATGCTCTTTGATGAAAACAGGAAACTATTGGATAGCAGGTTTATTAC</td></tr>
<tr><td>39726807|F|0-19:G>C-19:G>C</td><td>TGCAGTTCTTTTATTCCACGTTCCAGTTCTTCAACTTTCTGTTCCAATTTCAACAATACCACAAAAGGC</td></tr>
<tr><td>39723053|F|0-54:G>C-54:G>C</td><td>TGCAGCTTGGAAATATAGACCGATGCCGTAAGTTGTATGAAAAGTATTTGGTGTGGTCACCTGAAAACT</td></tr>
<tr><td>39714854|F|0-23:G>C-23:G>C</td><td>TGCAGATCAAGTATTTGTGAACAGTGTCAAACTTCCCAGTGACTCCGTTGTGGAGTTCTTCACTGCTCT</td></tr>
<tr><td>39722007|F|0-43:G>A-43:G>A</td><td>TGCAGATCATCCCATATGTGTACAGACGAATGATGCTTCTGGTGATGACGTGAATAACCCATTGTTTTG</td></tr>
<tr><td>39718978|F|0-24:A>G-24:A>G</td><td>TGCAGTTTTTCACTACCAAGCCATATGCTTGTGAGCCTTCCAGCCTTCCCAAGTATCCTCCAAGCAAGG</td></tr>
<tr><td>39728567|F|0-24:A>G-24:A>G</td><td>TGCAGCCATCATCCCAAAAGCATGACCAGCATTGAAAGTTGGAGCACCCCACTGAAGAGGGTATGGAAT</td></tr>
<tr><td>46775928|F|0-14:T>C-14:T>C</td><td>TGCAGTTGCTCTAATGAAACATAGGGAACCAAATTTTTAC</td></tr>
<tr><td>39717415|F|0-49:T>C-49:T>C</td><td>TGCAGGGTTTTATATTTACAAATACATTTCTAAATTCCTTTTCATATGGTTTGATCTATTATTGTCTGT</td></tr>
<tr><td>39715713|F|0-12:G>A-12:G>A</td><td>TGCAGCATCTCCGGCCATCTTCTTCCGTTCCTGTTCACGATTCTTCGTTGTCGGTATGATTGACTCTTA</td></tr>
<tr><td>39727033|F|0-22:A>G-22:A>G</td><td>TGCAGATCGGGGACCACGTCCAACTCGCCTACACTCACGAAAGTGAATCCGCCTTAC</td></tr>
<tr><td>39720534|F|0-14:C>A-14:C>A</td><td>TGCAGGTTCTGTCACCGATCTCTCCCTAACAGAATCATATTTAC</td></tr>
<tr><td>39726602|F|0-68:T>C-68:T>C</td><td>TGCAGTAGAAACAAAGACATCTATTCCTGGAAGATCAGATTTTCCAGTAGGATTACTAGGGCTAGGTGT</td></tr>
<tr><td>39715614|F|0-6:G>A-6:G>A</td><td>TGCAGCGGTAAATTTTTCATCAAAGAAAGGGGATGCCTACGTATCATCTAGTAGTGACATTGCATCTTG</td></tr>
<tr><td>39716826|F|0-33:G>A-33:G>A</td><td>TGCAGGAATTATCTTCATACCACCGCTACCTCCGATTACCGCAATCAACCGATCATCCTATGAAGGTTC</td></tr>
<tr><td>39715306|F|0-50:G>C-50:G>C</td><td>TGCAGCAACGTGGACTTTTGGGATGCCTGTATTGCTCAAAGCAGCCTTGGGAAGAACAGTTTGAGAGAT</td></tr>
<tr><td>39717537|F|0-34:C>T-34:C>T</td><td>TGCAGGTGCAGCCCAAGGCAGTGAAGAGGCTGTGCAGAGTTCACAACATTATAACAGTGAATGGGCAGT</td></tr>
<tr><td>39727306|F|0-14:T>G-14:T>G</td><td>TGCAGAAGCATTGTTGGGGAGCTAGATGAGGAACTTGATTCTAATTTGGACTTGTCGAAATTGAGAGCT</td></tr>
<tr><td>46756835|F|0-53:A>C-53:A>C</td><td>TGCAGGTTCAGGAGTTTCTTCTCGATGGGCAACTTTGACTGAACTTTTTTGTAATTTACCCACTTCCTG</td></tr>
<tr><td>39720323|F|0-5:G>T-5:G>T</td><td>TGCAGGAGATAGGGGAGCAATTATTAC</td></tr>
</table>

39715439|F|0-54:C>T-54:C>T	TGCAGCACCAGCTACCTCCATAAATCAAAAATTAGCAAGACACAGGGATATCCTCCATGAATTTACACA
39717533|F|0-66:C>T-66:C>T	TGCAGGTGATGGTGAAGTGAACGTTGATGAGTTCTTTAGAATGATGAGGAGAACAACATATGGATTCTA
39726181|F|0-32:T>A-32:T>A	TGCAGCCTTGGAGATGCCGTACGCTGGAAGCATTGTTGTCCATCCATTTGCTTCTAATCTGTCTTCCTT
39716678|F|0-57:T>C-57:T>C	TGCAGCTTTGGTTTGGATCTTTTGAGCATTCCCTCACTTTGTCTGCTGTTGACATGCTATGAAACTCCT
39718355|F|0-46:A>G-46:A>G	TGCAGTGCCTTCTCAACCACACCACACTTACAATACATATCAATCAAAGAGTTTCCTACAAAAACGTCT
39727061|F|0-28:A>G-28:A>G	TGCAGCAACCCCTCTTCAAATGGGTTCCATTGCTTGGAGGCTTAC
39728663|F|0-32:T>C-32:T>C	TGCAGTCAAATTACTCCTTATTTTTAGGATCTTCATCCATATTATTAC
39726814|F|0-29:T>C-29:T>C	TGCAGTTGACTATCTAACCTACGGCTCAGTGGATTTCGCCTTTGCTCCCTATGGACCCTTTTGGAAGTT
39720419|F|0-13:G>A-13:G>A	TGCAGGCTCTCTTGCAGAAAATGCACGCACTGTAAGGATTTGCAATTGTATTATGATTAC
39713713|F|0-64:A>T-64:A>T	TGCAGAACCTCCTAATATTGCTGCAAGGACCCTTGACATCTCATGTGGGTTCTTCCCTTCACCGATGCT
39722862|F|0-42:C>T-42:C>T	TGCAGCTAGAATTTTTTGAAGAGCCTGGTCTTGTAACTCGATCGAGACTCCCTCTCCGATAATGGTGGA
39722759|F|0-67:T>C-67:T>C	TGCAGCGAAGAGTGTGAACGACATTCCAAGGTATCAATACAATTACAATATAATCTCTCGCTGCTTGTT
39729173|F|0-62:T>A-62:T>A	TGCAGTTGGGATCTTGCTAAGATGCATTCAACAGGATGGGAAGTGCAGAAATATCATAGCTGTTACAGA
46776198|F|0-31:C>T-31:C>T	TGCAGAGATGCAACCATTATACGAAGAGTTTCAGAAGGATTAC
39726050|F|0-27:C>T-27:C>T	TGCAGCAGCATCTTCACTATTCTTGTTCTTTCCGAGATTGACACCAAGAATTCCAGGACCAGCTTTACC
46754511|F|0-9:C>T-9:C>T	TGCAGAGTGCTTGCTCACTTTTGCACGTGAATAAGATATTTGAAGTTGGCATATCAGAGAATCTTGGAG
46767068|F|0-33:G>A-33:G>A	TGCAGCAAGCTGGCCAAGGAAGAATCTGTGCTCGTTCTCCCTGGTAATCAACATTAC
39716419|F|0-62:C>T-62:C>T	TGCAGCTGCACCATTCTCTCCCTTGGTTTTATTATTGCATAAGGAGGAAATGGGATGGTTGTCTATGTC
46775546|F|0-23:T>A-23:T>A	TGCAGAAGGTATCTTGTTGGCTGTGTGAGTAATTTGTTCAGTTCAACTATTTAC
39724747|F|0-7:T>C-7:T>C	TGCAGAGTGCTAAAACAGCAACTCCGTTTTAC
46760930|F|0-24:C>T-24:C>T	TGCAGCTTCTTTGCTGATCGTTGACAAAAGTTTTGCAGCAGTGTTAC
39725508|F|0-43:G>A-43:G>A	TGCAGAAAATGGAGTCAAGAGAAAGTGGCCATTTTCAAGCAGTGGCAGGTTCAACTTCCATTGGCAGTG
39728023|F|0-6:C>T-6:C>T	TGCAGCCTACAGAAAAACATTCATAAGACAACTGAATAAGGAACAATAGATTAC
39714898|F|0-63:G>A-63:G>A	TGCAGATCCTTGTGCGGGTTACATTTCATGTTTACTTTGCTCGATGCTGAATGTCAATATGGTGAATTG
39720373|F|0-29:A>G-29:A>G	TGCAGGCAACTTCCAGTCGGTTACCAACAAATGGAGCTCAGGGGACAAATTAC
39727693|F|0-47:C>A-47:C>A	TGCAGGATATCAACTTGATATCGTCATTCTAATTCACTAAATTCATACAACAATTATAATCTTCTCTCA
39717768|F|0-59:T>C-59:T>C	TGCAGTAAGCACAGAGCATTGTGTTCAATATTACAATGTCATATCTGTCATATGTGGCTTCAAAATCTT
46754517|F|0-40:T>C-40:T>C	TGCAGAGTGTTCTTCCTGGCATTCTTCAAGGGGACAAATCTGACTCTCTTCTGTATGGTTCTGTTGACA
39717598|F|0-68:C>T-68:C>T	TGCAGGTTATAAATTTAGGCCGTAGATTCATAGCATCTACAAAATACATCTTCTAACTTTTCTCCCCCC

39727379|F|0-64:T>G-64:T>G	TGCAGAATCCAAAACTCTGTACTACAAATGGGAAGTCAAGTCCGAATTGAAGTCCCCCGACTGCTTTAC
39728023|F|0-11:G>A-11:G>A	TGCAGCCTACAGAAAAACATTCATAAGACAACTGAATAAGGAACAATAGATTAC
39715852|F|0-24:G>C-24:G>C	TGCAGCCACCTTTTTCAAATCCTCGGCAGTGCTCTTTCCATCCCAAAATGATTCAAGAGCAAACTTGAG
46762283|F|0-55:T>C-55:T>C	TGCAGGTTTTCAGCATGAATCTTTCTGCTCCATTCATTTGTGAGTTCTTCAGAGATGTGCAGGAGAAAG
46775149|F|0-30:A>G-30:A>G	TGCAGTAGAACCTAAATCAAAAGAAAAGGAACTTATCAACATCATAATCAAGCCAAAGAATTCTAATCT
39721247|F|0-16:T>C-16:T>C	TGCAGAAATTGCAGGCTCGAGATTCTGGAACTTTCGCAGCGCTAGTATCAGAAATGTTCGATCAAGACG
39729130|F|0-43:C>T-43:C>T	TGCAGCATGAAGGGCTGTCCGACCCATACTGTCCACCATATTACAGCAATCAGGACGACAATTTACAAG
46763984|F|0-13:C>T-13:C>T	TGCAGCTAGAGGCCTCTTGATACGTCAGCCAGGCGTTCCGGTTCAAGCTATGCCACCACTTACCCAGCT
46772327|F|0-12:T>C-12:T>C	TGCAGTCAAGGCTCTTGCATGGTGTCCATTCCAAGGCAACTTGCTCGCAACAGGTGGAGGTGCTGGAGA
39725937|F|0-25:T>C-25:T>C	TGCAGATTTCAGGATAAGCGTTACTTTTGACTACAAAATTCAAAACAAAAAATGTAACCAACTAAATCA
46760877|F|0-26:C>T-26:C>T	TGCAGCCTAAGAAGCCTAAAACAAACCTGATCTTTTATGATTAC
46775699|F|0-42:G>A-42:G>A	TGCAGGGGAGGTGATACCAGCAAACTTGGCAGCCACAATGACGGAGAGTGGAGGGCTATCGGACGGAAA
46775909|F|0-21:C>T-21:C>T	TGCAGATCCTGTAAAGCCAATCATCCTACACAGTGGACCATATTAC
46775920|F|0-18:C>T-18:C>T	TGCAGGTGTATTTGCTTACAGAGGAAATTTGTCCTGTTATATTAC
46772963|F|0-9:C>T-9:C>T	TGCAGCTCTCGTTTTAGCACTCATCTTAC
46774415|F|0-33:T>C-33:T>C	TGCAGAAGATGCAAGCTAAGCTGGATAAGTGCATCAAAGAAAAGAAATTTCTAGATGACGTTAGTGACC
46775310|F|0-12:C>T-12:C>T	TGCAGGAATCATCTCCACTCTTCGATTAC
46776067|F|0-57:G>A-57:G>A	TGCAGCAGAGCTGGCAAGGGATCGGATTCTGATTGGAATTTGTGATGGTCCTATGCTGAGTAAAAAAAA
39729616|F|0-39:A>G-39:A>G	TGCAGTTCGGTGGAAGATTCTTGTTATTCCACGCTGAATATCTGGTGCCCTTCCCAAAGTTGTTACAGA
39728455|F|0-19:G>T-19:G>T	TGCAGATCGTGAATTGTTGGGTCAGAAGTTCTGGATGTCTTAC
39719401|F|0-25:T>A-25:T>A	TGCAGAAGCAGGGGAGAGTAAGGTATTATTGAAATTAC
39713697|F|0-23:C>A-23:C>A	TGCAGAACCACTAATTAGAGGAACAATTTGAAATGGATGACAATCTAACTGAGAATCTGTCTAAAATCT
39719491|F|0-17:A>G-17:A>G	TGCAGACAAACCAAAAGATTTTCATTGTCAATATCTCTGTAATTTAC
39719480|F|0-6:A>C-6:A>C	TGCAGAATTCTACCAGTAAGACACCTTAC
39724772|F|0-18:T>C-18:T>C	TGCAGAGTTCAGTTCAGCTATCCCACATTAC
46774612|F|0-47:A>G-47:A>G	TGCAGTATACAAGGCGCAGAACAAAGTGAGATATTATATCCTTGTTCACAACCTCGGATGCCTGTGGAT
46775568|F|0-10:T>A-10:T>A	TGCAGCATCATGAGTAAGAAGGTGGAACCATTAC
46775579|F|0-18:T>A-18:T>A	TGCAGCTGAAGCACTGAATCTTTTCAATGAAATGATTAC
39724313|F|0-35:T>A-35:T>A	TGCAGTTGATCATCCTCTAATGTAGCATACCATGCTGGAATTCTGTGACCCCACCAAAGTTGCCTTGAG

<table>
<tr><td>39724338|F|0-21:G>A-21:G>A</td><td>TGCAGTTGCTCCACCTGTTTCGAATGTCGAGTGGGCCCCTGAGACAGGTTGAACCCGATTTTCCTTATA</td></tr>
<tr><td>39727013|F|0-28:T>C-28:T>C</td><td>TGCAGAGTGATCATCTCCTTTGGTACCTTACTCTTAC</td></tr>
<tr><td>39728281|F|0-18:G>T-18:G>T</td><td>TGCAGCTAGTGTCCAGTAGTCTAGTGAAAAGTTCATGTCCTTGGCCATGTTATCGCGTGTAAAAGGAAA</td></tr>
<tr><td>39729066|F|0-39:T>C-39:T>C</td><td>TGCAGAAGTGTGCTCTTCAAGTCTGTGAAGCCACTGGGTTGCTGAGTTCGAAGATGCCATTGATCTATC</td></tr>
<tr><td>46767095|F|0-20:A>T-20:A>T</td><td>TGCAGCAGTAGGTTTGATCTAACAGTTAC</td></tr>
<tr><td>46770746|F|0-25:C>A-25:C>A</td><td>TGCAGAAGGTAGCTGTTATTCGGGGCAAACTTAC</td></tr>
<tr><td>46770870|F|0-23:C>G-23:C>G</td><td>TGCAGCAGGCTTTCTCTTCTCAACCTTAC</td></tr>
<tr><td>46770879|F|0-15:C>G-15:C>G</td><td>TGCAGCATCCTTGGTCAAGTGTCTCAGGTATATTTTTGGGATTTAC</td></tr>
<tr><td>46772412|F|0-65:G>A-65:G>A</td><td>TGCAGTTACCATGGATGAGCAGGGAAATGAGACGTTTGATACAAGTGGCGCTCTTGATAAACTACGCAA</td></tr>
<tr><td>46773536|F|0-10:C>T-10:C>T</td><td>TGCAGCAGATCACTGGTTTGGCCAAACAGGCAAGTTTGACACATTCACCATGTCAACAATGCATTATTT</td></tr>
<tr><td>39719342|F|0-19:A>C-19:A>C</td><td>TGCAGAAATTTATGGGCTCAATATTCTTGCAGAAAATGTTCAGGTAAGACTTCTTTTAC</td></tr>
<tr><td>39715534|F|0-35:T>A-35:T>A</td><td>TGCAGCAGCAACTACCACTGTTGTAGCCAAGCGCTTTGCTTTTCTATTAGCTTCACTTATAGAATTATT</td></tr>
<tr><td>39722627|F|0-23:C>A-23:C>A</td><td>TGCAGCCATCGGGAAGGAGTTCACAAGAAGCACACTACTACTTCAAACCAAAAGAGAAATGGATATAGC</td></tr>
<tr><td>39724861|F|0-8:A>C-8:A>C</td><td>TGCAGCAGAAGTATGATCTGGAAACAAAGAGATTTAC</td></tr>
<tr><td>39725471|F|0-35:G>T-35:G>T</td><td>TGCAGGAATGATTCTAAGTTAGCAATTAGAACTGCGCACCGGTGTTTTTGAACTGTAATTTCGTGAGCA</td></tr>
<tr><td>39715760|F|0-27:G>T-27:G>T</td><td>TGCAGCATTCAGATATTCGCAAGCTTCGTTCGGAGCAGCAGAGTCCCAAATCATAGATTGATCAGCCTC</td></tr>
<tr><td>39717749|F|0-46:A>T-46:A>T</td><td>TGCAGTAACTAACAACTGATACATATATGATAATATGATCAACATAAAATAAATGAAATGCTCATGATC</td></tr>
<tr><td>46755567|F|0-45:T>C-45:T>C</td><td>TGCAGCCGTAGTAATCCCCCAAGGATAATTTCATTGTGAGTGCATTATTTTAGCATAGCAAAGTCGTCC</td></tr>
<tr><td>39714482|F|0-62:A>T-62:A>T</td><td>TGCAGAGCAAATTGCTTTTGAAATCTTGACATGCTTTTTCTGAGTTCCATCATTATTATTATAGGTGAA</td></tr>
<tr><td>39726423|F|0-50:T>A-50:T>A</td><td>TGCAGGCACATAGAAAGCTAATGGTGAGAAGCATAAAGAAAATCCAACCATTGGAAGCAACTCATCTAA</td></tr>
<tr><td>39720706|F|0-32:A>T-32:A>T</td><td>TGCAGTGAAAGGTGGAATTTGGGTGAATGATCAATTAC</td></tr>
<tr><td>39716529|F|0-21:C>T-21:C>T</td><td>TGCAGCTTAGTTTTTCGACTTCGAATGCTTTACGAGCCAGTCAATAACAGTGTCGATATTGGTGGAGTT</td></tr>
<tr><td>39726257|F|0-34:A>G-34:A>G</td><td>TGCAGCTCCAGTCGGTGGGAATGGCTGTTTGATCATAAAAAACCAAAAAGGAATTTAGAAATAACTTAC</td></tr>
<tr><td>39726994|F|0-25:C>G-25:C>G</td><td>TGCAGAGACTGGTGAATCTGTGCTCCTTCTTAC</td></tr>
<tr><td>46755438|F|0-30:C>A-30:C>A</td><td>TGCAGCCACCTCTGCTTGCATCGCAGTGCGCAAGATCCAACTTTGGTGTCCGACCACAGCCAAAGGTGG</td></tr>
<tr><td>46755747|F|0-8:A>G-8:A>G</td><td>TGCAGCTAATAGGCAATACTCCTATGGTGTATCTCAACAAAATTGTAGATGGCTGCTATGCCAAGATTG</td></tr>
<tr><td>46768538|F|0-10:A>G-10:A>G</td><td>TGCAGAGATCACGGTGCCATGTAGGAACATTTTTGTATGTCAATCTGGCAGTAACATCAAACATGATGA</td></tr>
<tr><td>46755807|F|0-63:A>G-63:A>G</td><td>TGCAGCTCAGCCATAAAAACTTGTTGCAAATTAGATTTGCAAAGTTCATACTGTTTTTCATCAAACTTA</td></tr>
<tr><td>39725706|F|0-32:A>G-32:A>G</td><td>TGCAGAGAAGTGCAAGAAAGGAACATCAAACAACCCAGCTGCCAAAGAAACGAGTAGCTCATGTCGCTC</td></tr>
</table>

39726298|F|0-25:A>G-25:A>G	TGCAGCTGCTGTAGAATCGATTGGAAATTCTCTTCTAAAGGATCTCTCAATGCCTTACCCAATCATCTC
46764364|F|0-38:T>C-38:T>C	TGCAGGAGTAAGCTGCAACAGTTGGCTGAAGATGGCCATAGACAGCAAGAGGAGCTTCTTCAACGACAG
39726437|F|0-11:A>G-11:A>G	TGCAGGCGAGGAAACTCATGGCCATCCATCAATTTCATATCCACCACTTCTTCCAAAACCTTCATCCAA
39728526|F|0-22:A>T-22:A>T	TGCAGACGTTTCAGATACATTGACTGTCTTCTACGAAGGTCCATAGAAAGATTCTGAAGCTCTGTAGCA
46757776|F|0-66:C>A-66:C>A	TGCAGTTTGGGGACATCACTTTCCAGAGGGAAGCCATTTTGAAGAAACCTACTAATAATCCTGAGGCAT
39716073|F|0-51:C>A-51:C>A	TGCAGCCTTCAACCGAGGGCTAGGCTCTGGCTCACCCTTCTCCAAAATAGGCGACAATTTGCCAGCATC
39726526|F|0-50:C>T-50:C>T	TGCAGGTCCTGAGGAAAATCTGTTGGTACCTAGTTCTAGCACCACATGATCCCATGCAGTCAAGCCTTC
46773772|F|0-61:A>G-61:A>G	TGCAGTCTTCCAACTTCAAAGCTTCTTATCTATGATTTTCTAGCAATGGGCAGCTTAGATGATTTCTTG
39719807|F|0-10:A>T-10:A>T	TGCAGATTGGAATGAATAAAAAGGCTGGAGAGCAGTTAC
39716065|F|0-16:C>T-16:C>T	TGCAGCCTGTTCCCCACATAATAACTCCTACTTTGATGATCAATCGTGCAAGTAAGATATTCTTACCAA
39722513|F|0-9:G>A-9:G>A	TGCAGCATCGACTTCTCTCGGTGATGTTTCTGGAATGGAAGATTGTAATCAATATCCCAAGTTTGTATA
39728287|F|0-23:G>A-23:G>A	TGCAGCTCGCAGCGATCACTTCGGAGGAGCTTGTTGAAGATCCTTTGAAATCGGCAGAGCAGTCCAAGC
46776614|F|0-35:C>A-35:C>A	TGCAGAAAAAAACTGAAGCTGAATTTTGTAGCAAGCACCAAAATCCAGAACAAGCTGTACTTAC
39720470|F|0-44:A>C-44:A>C	TGCAGGTAAGGACGTAAGGTGTACTTTCTCACCAACTGATGACCAATTAC
39719995|F|0-27:A>G-27:A>G	TGCAGCATGCAAGAGAGCAAAACTTGCAAATTAC
46756224|F|0-60:C>T-60:C>T	TGCAGGAGAGGCACACTCATTGAAGGTGGAGAAAATTTTATTTCGAGGGAAGTCCGACTACCAGGATGT
39727797|F|0-15:A>G-15:A>G	TGCAGTATACAAGGCACAGAACAAAGTGAGATATTATATCCTTGTTCGCAACCTCGGATGCCTGTGGAT
46761052|F|0-46:C>T-46:C>T	TGCAGTCATGGCACCATCACCTATAACTGCAACCACATTGTTCTTTCTTCCCTTAC
46773536|F|0-17:T>A-17:T>A	TGCAGCAGATCACTGGTTTGGCCAAACAGGCAAGTTTGACACATTCACCATGTCAACAATGCATTATTT
39713294|F|0-28:T>C-28:T>C	TGCAGTAGTAAATCTCCAAATGGAAGCTTGAGTTGGCATGCAATACATTCTGAAAGCACTTCAATGTCT
39727054|F|0-15:C>T-15:C>T	TGCAGTAAAACAAGTCCCGGAAGGTTTATGAAATTAC
39718976|F|0-56:A>T-56:A>T	TGCAGTTTTTATTTGTGGACTCAACATTCTTGCAATCAGTCGTTTTGAAACTGAGAAAACTGCTTGGTT
46775797|F|0-35:G>A-35:G>A	TGCAGCTGGACACTTTGAATGAGAAGATAGTTTCTGGTGGTTTAC
46757148|F|0-36:G>T-36:G>T	TGCAGTCCACGTATACAGAGGTGGGACACGTGGCATGATCTCAGCAGCATAAAAGCTAAGCCGCCTTCT
46773722|F|0-49:C>T-49:C>T	TGCAGGTATTTCATCTTCAAAGACATTGAATCTCCCACTTTCACTATCTCCTTTTCAATCTCAATGGCT
39727780|F|0-47:G>A-47:G>A	TGCAGGTTTTGCAGTCGAAGGATCTTCTGGTGGCAATCCAAGCCTCCGTCTTCTTTCTGCCTGTGAAGA
39713451|F|0-12:A>G-12:A>G	TGCAGAAAATAAACGACCTATAATTCCAAATTTGGAATTCGACTACCCTGATCAGGATATTCATGAAGA
39717994|F|0-65:G>A-65:G>A	TGCAGTATTTTGTAAAAGCTACACCTTGCAGGCCAAAGACGAAGATCGAAAAATCTATAGCGCCCGAAG
39721858|F|0-10:G>T-10:G>T	TGCAGAGGATGGACGCATAGGCAAAAAGACAGGCATATACACATCTGTCATATACGTACACTCAACCAA

46755855|F|0-60:T>C-60:T>C	TGCAGCTCTGATTCTTTCAGCATAGGCCCTCAGTTTCTTGATCGTTGGAACAGTCTCCAATGAGTCCCT
46756762|F|0-65:T>C-65:T>C	TGCAGGTCTTTGAAAGATGTCAAAGGCTACATATTCACTGATGTCTCATCAAAGAAGCTTTTGGATGCT
46772914|F|0-7:G>A-7:G>A	TGCAGCAGCAGTATGATCTGGAAAGAAAGAGATTTAC
39721828|F|0-21:A>G-21:A>G	TGCAGAGCTTCCATCACCATCAGGCTCCTTCTCAAAGTGTTGGTTTGCAGTAGCAAAAGCTATATCTTC
39718395|F|0-48:C>T-48:C>T	TGCAGTGGAGCTCAATGGCGTGCTTTATGCAACAGGGGGGTTTGATGGCAGTGACTATTTGAAGTAAGA
39727816|F|0-67:T>G-67:T>G	TGCAGTCATAAAAGCTAAAACAAAATAACGAGAGGTAAATCAGGATTACAAGGACAGACAATCATAATC
39728884|F|0-64:C>T-64:C>T	TGCAGGACATGTGCTGTTCGTTGTAATCCTGAATATGATGGTCTAATATTGGTTCGTTCTCCTTCCCTC
39720539|F|0-17:C>T-17:C>T	TGCAGGTTGCTGAATTTCTTGGTTTTAC
39726288|F|0-40:C>T-40:C>T	TGCAGCTGATCCCAAGTTACTTGTTTATCTAAAATCGTATCGCAACACAGTTCCTGTGCCAAGGCATTG
46755273|F|0-43:A>C-43:A>C	TGCAGCAGTCTGCCAATTCCAGAAGGTCAAAGAACAAATAGTCATTGCCATACAACTTCAGAAGCTCAC
39713452|F|0-55:A>C-55:A>C	TGCAGAAAATACCATGTCTACTTCAATCTATGCATTTCATTCATATAACTTTTATAGGCTGGTTGTAAG
39716966|F|0-61:C>T-61:C>T	TGCAGGATATGGATCGAGTTTTAGCTGGCCAGTATGTTTCTTGTCCATTTCTCTATTGCTGCTTTTTTA
39717046|F|0-41:T>G-41:T>G	TGCAGGCAAGAATTCAAGTCAGTTTTCTTTGAATGAAAATCTGAAGATTGATCATCAGCTTGAACCAGA
39723251|F|0-25:T>C-25:T>C	TGCAGGATCTATTGCCCTTCATGGATGGAGGTCCAAAGGATCATCATGTCACCTTATCTCATAAACGTC
39729344|F|0-52:A>G-52:A>G	TGCAGATCAGAAACTTCAACACCAACTTAGATTTCCAGCCCATAAAGAAATGAGCATTTTGATTTTACA
39727682|F|0-50:A>T-50:A>T	TGCAGGACTCAAGAGCTGTTCGATTTTTTGATCGTCGGGTCAGTTATTCAATCTTTCCTTCATGTGTTT
39717153|F|0-55:C>A-55:C>A	TGCAGGCCTTTGCTCGTATATTTGGGTCTTCATGTGAAAGGAATTCCTTTAGGAACTCCAAAATAGATG
39715342|F|0-29:T>C-29:T>C	TGCAGCAAGCTGATGAAAAGGAAAATGGATTTACAATCAGGTAGTAGGATCGAAGGAAAAGGGAAATAG
39717643|F|0-34:G>T-34:G>T	TGCAGGTTGAAGCAGCTGTTTTGTACCCGGGCGCGCGTCCTACTGTGCTTAGAATCCGCATGAGGTTTG
39724602|F|0-46:G>C-46:G>C	TGCAGAAATAGGATGGTGGTGTTTCGCTATCCGCCATTTCTAAGCAGCTGCAATTAC
39716555|F|0-9:G>A-9:G>A	TGCAGCTTCGTTTGGCATAAGCACAATATTGCTGGAAAAATATATCACCCAGCCAAGGCACATAGAAGT
39718636|F|0-29:G>T-29:G>T	TGCAGTTCCAGTTCAGAGGGCTGTCTGGAGCCATCACCTGCTAAAGTTCCAGCACCGTAGGGGCTCCCG
39720858|F|0-17:C>T-17:C>T	TGCAGTTGCTGCCGTAACATTGCTGCTTCCCTTTGCCAGTACTAATGATTAC
39719465|F|0-19:G>A-19:G>A	TGCAGAATGATGCACGCACGAGCATGTCATTTAC
39722303|F|0-44:T>G-44:T>G	TGCAGCAAGTTTATTCTGGGCATTCACTCTCAAAGAGAACTTTCTCAGCACATGGTACTGATGGCTTCC
39714198|F|0-68:C>T-68:C>T	TGCAGACCCAGCAAACATCCCATTCACTCCAAAAGGAAAGTATCTACAAGAACATGAGAAAGCAAAAAC
39713903|F|0-12:A>G-12:A>G	TGCAGAAGGTCCATGTAGAAACACTCTGGCAACCTTGCAGCAGGATTACAAACAATTTTTCCAATATGA
39714431|F|0-59:A>C-59:A>C	TGCAGAGAGTAGCACAAGTCAGCAGGAGTACACTCATTACTTTTTAGCTTCTCCTCAGCAGTTGTTTCA
39713308|F|0-56:C>A-56:C>A	TGCAGATCTCCATCCCCAAACCATGACTGGACGACAAGAACAAAGCCGAGATAGAGCAAGAGTGAAAAA

39716503|F|0-5:T>C-5:T>C	TGCAGTTGTGAATCAAATTCTACTTTCCATTCAAGAGGAAGCTGCTTTCAGCTGACACTTTGAAAAAAA
39728526|F|0-9:T>C-9:T>C	TGCAGACGTTTCAGATACATTGACTGTCTTCTACGAAGGTCCATAGAAAGATTCTGAAGCTCTGTAGCA
39713498|F|0-15:A>G-15:A>G	TGCAGAAACATGCTCAATGAGCCTGCTGGTACCTCCTCAGTCGTTCCACTTATAGGGACTTGTGGTAAG
39714730|F|0-19:G>T-19:G>T	TGCAGATAAAGGGTAGAAGGGGATCAGGTGCAGATCCATGTTTATTTCCCCTGCTCTTTGTTGTCAGCT
39725094|F|0-18:A>C-18:A>C	TGCAGGCAAAGGTCTAGCAAAACATTCCAAAATCTGTTGCCTTAC
39724334|F|0-45:A>G-45:A>G	TGCAGTTGCCAACTCTACCCCAGCACCCAATTCCATAAGGAGATCACCTGCAAAACGTAGCTGCAACCA
46760941|F|0-10:C>A-10:C>A	TGCAGGAAATCAACTTATGCGGTTGCTGTTAC
39715828|F|0-58:G>A-58:G>A	TGCAGCCAATGCTAACTGCATGTGATCGATAAGGTTGAATTAGATAGTACATAATAAAGTTATCAAATT
39728072|F|0-41:T>C-41:T>C	TGCAGGTATCGTGACCCAAAAACAGGGTTACCTTATGCAACTAAAGAAGCTTTTAC
39716122|F|0-6:G>T-6:G>T	TGCAGCGATAATCTGCTCGTGGAGAATATTACTTTAGATATCTTGGTGCCGTCCAATAGAATTAGGTGA
39714199|F|0-26:G>T-26:G>T	TGCAGACCCAGTGGCATCAGAGAACAGCATACCTTTGTCCCCTCAATGGCTATATGCCAAGCCAAGTGA
39724760|F|0-39:A>G-39:A>G	TGCAGAGGGCACAATCTGTTTCAGGACACTTCCACCAAGACATATTTTAC
46755252|F|0-52:C>G-52:C>G	TGCAGCAGGGGAAGGATTTGGATTTCAAAATTGGAGGCAAAATCGGAGAAGCCAACGAGGTAATGGAAG
46774035|F|0-29:T>G-29:T>G	TGCAGAAGGAAATGTTGGAGAAGGTGGGGTTTTTAC
46775267|F|0-7:G>A-7:G>A	TGCAGAGGAACTCTTCTTGCTCCATTAC
39721173|F|0-44:A>T-44:A>T	TGCAGAAACCATTGAAACTATTTCCACAGTTGTGTACCCAGCTAATTATGGGACCAATAAGTTGCGGCT
39723486|F|0-40:A>G-40:A>G	TGCAGGGGACAAGATCACATGAGGAACAGGAGATAGAGAAATAGATGAGGAAATGATAAAGAGAGTACA
46754306|F|0-31:G>A-31:G>A	TGCAGAGACGGGATCTACCTTCACTAATCCAGGGCCAATAGCAGCTGAAGTACCTAAATTTCCTCCAGA
39719672|F|0-38:T>C-38:T>C	TGCAGATAACAATTACTTATTCACAAATCAGTTTACAGTCATCAGATTTCAAATTAC
39719938|F|0-27:C>T-27:C>T	TGCAGCAGATATCTGGAGCGTATCCATCCCTTTCATTTTAC
46771749|F|0-37:G>A-37:G>A	TGCAGAGAAGTGCAAGAAAGGAACATCAAACAGCCCAGCCGCCAAAGAAACGAGTAGCTCCTGTCGCTC
39714006|F|0-36:G>C-36:G>C	TGCAGAATCCTTCAACTCCCCTTCAGACCTTTGACAGCGGAAAACATATATTGATATGATAGCCAGTAA
39727224|F|0-6:C>T-6:C>T	TGCAGTCTCGATGATGCTTCGTTCAACAGATTTGTGCGTGGTTTAC
39715231|F|0-28:T>A-28:T>A	TGCAGCAAAAGTCACAACTCAGTAAGACTTTACAGATTAGAAATTCTTGTTTCAAAATAGTCTTTTCTT
39723551|F|0-48:T>A-48:T>A	TGCAGGTAATGTTCTTATTCCCTTGTTGAAATGGTATAGATAATAAGCTATGTGGCTTGAAAGGGCAAA
39713710|F|0-33:T>A-33:T>A	TGCAGAACCGATTCCATATGCTAATGTAAAAACTGTTGTCATAGTATTGAAGCTGTTCATAAAAACCAA
39725926|F|0-20:T>C-20:T>C	TGCAGATTGTCAAAGTAAACTATTCACGATGGTTACTCTAAATAATAAAAATGAATGGAATAACAATTA
39714124|F|0-31:C>T-31:C>T	TGCAGACAAGGTGACAGATCCTAAGATTAGTCGTTTTCTATACGAGAAGGCGTCGAGCAAGGACAAGAC
39715049|F|0-63:T>G-63:T>G	TGCAGATGTGAAGAAGAAAACCTTGATGTTATTTGATATAATATTTCTGAAAGATCAAGCTTCTGCTCA

<table>
<tr><td>39720600|F|0-42:A>G-42:A>G</td><td>TGCAGTAGAACAACTGCTGCTTGGCCAGTTTTTCAAAGAAAGAAACCCTTAC</td></tr>
<tr><td>46757083|F|0-34:T>C-34:T>C</td><td>TGCAGTATTATATGAGGACAGGAATGTGCAAATTTGGTGCTTCCTGTAAGTATCACCATCCTCAGCAGG</td></tr>
<tr><td>39720230|F|0-10:T>C-10:T>C</td><td>TGCAGCTTCTTCCAAGGGAGAAGAGAAAGCAGGAATATCAGGAGGCTGTTAC</td></tr>
<tr><td>39715670|F|0-51:A>T-51:A>T</td><td>TGCAGCATATATGTTTTGAGCTGCAATAAAAAGTCAAGCATAGTCAATATCAACTAGCAGAGAAATAAA</td></tr>
<tr><td>39728122|F|0-9:T>A-9:T>A</td><td>TGCAGAAAATCATCGAGTGTATCGAAGCTTGAAAGCTTATACAGAGTTACCAAGGTTTTGGCAGGGCAA</td></tr>
<tr><td>39727604|F|0-50:C>T-50:C>T</td><td>TGCAGCCTGAAGCCTGTTATGATTTTTTTCCTTCTTCTTTGGTTCACCTTCCTTCTATTATGCTCTTGC</td></tr>
<tr><td>39723942|F|0-17:C>T-17:C>T</td><td>TGCAGTCCCAACTTCTGCATTTCGTGAGGAACAATCGGCATTCTCCGGGTCTTTTGATCCAACTATACA</td></tr>
<tr><td>39713818|F|0-12:A>G-12:A>G</td><td>TGCAGAAGATCTAAGACAGAGAATAATCGAACATGTAAGTATCTGTATATCCCTGGACTAACTCTAGTT</td></tr>
<tr><td>39720346|F|0-26:C>G-26:C>G</td><td>TGCAGGATATTTCTTTCTATTTATTTCATCAATTTCTTTTAC</td></tr>
<tr><td>39724657|F|0-18:T>G-18:T>G</td><td>TGCAGAAGTTGAACTTAGTGCAGAAGTGTTCTTAC</td></tr>
<tr><td>39714411|F|0-37:T>C-37:T>C</td><td>TGCAGAGAGAATGCCAGACAGAGATATGGTTCAGGATTTGCTCATAAGTTACATGGATGGTACATGCTA</td></tr>
<tr><td>39716258|F|0-41:G>C-41:G>C</td><td>TGCAGCTACCTGGTGTTCTGGATGATTTGTTCTCATATGAAGGTCCTCTAGGTGCAGTTTACTCAAAAG</td></tr>
<tr><td>39721816|F|0-68:C>T-68:C>T</td><td>TGCAGAGCTATTGTCTTAGAACCTGGGAATCCATCTATGAAGAATCAAATAAAGGCATTAGCTGCTGCC</td></tr>
<tr><td>39715680|F|0-8:G>A-8:G>A</td><td>TGCAGCATGTTTTGCTAACTCAACATTGCCAAAAACTCTACATGCACTAAGCAAGCTCCTCCATACTAA</td></tr>
<tr><td>39722945|F|0-14:C>T-14:C>T</td><td>TGCAGCTGAGGATGCAGCAGAAAGGGAAAGTGACACTACCAGTGATTCTGAGGCTAAGACATTGAAGCA</td></tr>
<tr><td>39727054|F|0-5:C>T-5:C>T</td><td>TGCAGCAAAACAAGTCCCGGAAGGTTTATGAAATTAC</td></tr>
<tr><td>39716421|F|0-54:C>T-54:C>T</td><td>TGCAGCTGCACGAGCTTTCTGGGCAGCCGCAGGATCATCAAACCTGATATAACCCGACTCCTCTCCGAT</td></tr>
<tr><td>39720479|F|0-23:G>C-23:G>C</td><td>TGCAGGTAGAGTTTCACGGACTCGAGCTTAC</td></tr>
<tr><td>39724891|F|0-18:T>C-18:T>C</td><td>TGCAGCACATTCTTCATGTCTCCTGCGTTCATAATTGTCCTCATGTATTAC</td></tr>
<tr><td>39716110|F|0-9:T>C-9:T>C</td><td>TGCAGCGACTAGCAGTTATAATGATTTGACGGTCGTTTCGAGTAGTGATCGGGGGAATGTAGACCAGTT</td></tr>
<tr><td>39719817|F|0-44:T>A-44:T>A</td><td>TGCAGATTTCAGGTCCGACTCTTCTTGTGTGGTTTTTCATTCTTTTATTTTTAC</td></tr>
<tr><td>39721049|F|0-40:G>A-40:G>A</td><td>TGCAGCACATCAGGCCATACTTGCGGATCAATCCATGGGGGTTACCGCAGACTCGGCTGGAAAATAACA</td></tr>
<tr><td>39715719|F|0-33:C>T-33:C>T</td><td>TGCAGCATCTGGGGGCACTTCAGTTCTCAGAGACGCTCGACAGCCTAGCCCATGGAATTAGAGGTCGTC</td></tr>
<tr><td>39714745|F|0-23:G>T-23:G>T</td><td>TGCAGATAAGGAGAAACCCAACTGTGATCTTCAATCACCAAGGAAAAGGCAAATGGAGCAAGGAATAAG</td></tr>
<tr><td>39714956|F|0-38:C>T-38:C>T</td><td>TGCAGATGACTAAGAGGGATCTTCAATAATCTATCTCACACCCTGGCGAGCAGTGGCACGTCCAAGTCC</td></tr>
<tr><td>39722855|F|0-14:G>C-14:G>C</td><td>TGCAGCTACCTCTGGCTTTCTCATATCTGCAAGACCAAGCAAAATAATCAACAAAATATTTGTTATTTT</td></tr>
<tr><td>39722080|F|0-39:T>C-39:T>C</td><td>TGCAGATGCATGTGCAAGGAATAGTCATTTTATTGAGGTTGGATTTCCATACAAGAGAAGATTTTCTGA</td></tr>
<tr><td>39718465|F|0-24:A>T-24:A>T</td><td>TGCAGTGTAAAATCTAATACTAGTACCATAACCAAATACGATATAGATCTTTCACAGAACAAAATTCTT</td></tr>
<tr><td>39716763|F|0-62:G>C-62:G>C</td><td>TGCAGGAACTACAATATGTTTCATGGTGGCTTCGTCTGGCAAGACAACATCAAAGCCCTGAAGAAAGAA</td></tr>
</table>

39721368|F|0-13:T>A-13:T>A	TGCAGAAGATGCATTTACTATACAACCAAGAACTCACGACTGAAGCGTAGATAGACAACACAACAGCTG
39715220|F|0-44:G>A-44:G>A	TGCAGCAAAAACGTGTATAGTTTATGTCTGAAAGATGTTTCAATGATAATGTCATATCACTGCAAAATT
46756843|F|0-38:G>A-38:G>A	TGCAGGTTCGCCAGCGAGCAGCCGTGGTGGTTCTCTACGCAGAATTTGTTGACCAGATCTCTCTGCCGG
39716813|F|0-54:A>G-54:A>G	TGCAGGAATCTCAAACTCAGCAGGGACATTAGCAGCAATTGTGAGCACAATTGGAACTGGTTATTTTGT
39724376|F|0-54:T>C-54:T>C	TGCAGTTGTCCGGCTTATGGGGCGTGCTGCTTCACACATCACTCTAGAGTGTGCTTTGCAGACTCATCC
39727305|F|0-39:T>C-39:T>C	TGCAGAAAGTTTAGTATAAGAAGATATCGAGCATCATAATTCCATGGCTCTTGACGTAAGCATCTGTAA
39714179|F|0-48:C>T-48:C>T	TGCAGACATTTATGTTATAAATTCCCAGGTGATCTATTTTTGAAGAACCGCTCATGTAAAAGCAGTAGT
39725412|F|0-47:C>T-47:C>T	TGCAGTTTGGTTTGTTAGTCAGTGCCTAACAGTACAGATCTGAATGTCTTAC
39721922|F|0-17:C>T-17:C>T	TGCAGAGTGGCTCCTTGCTAGTTACAGATTTTGAAATAACAAAACATAAATCACCAAATCACTACTTTC
39727081|F|0-30:T>C-30:T>C	TGCAGCATTCAAATACTCAAAATAATTCCATGGCTTTGTTCATTTAC
39720328|F|0-8:G>C-8:G>C	TGCAGGAGGCTCCATCTTGGGCTCCTCCTCGTTCTTAC
39718408|F|0-47:A>C-47:A>C	TGCAGTGGATTTCTTGGAACTGTGGAAGGTACTGCCAAACTTATCAGAGAACTCTATGTATATCTTCTT
39727146|F|0-6:G>A-6:G>A	TGCAGGGTTGGAAAAAGGCACTGGTTCAGTTAC
39721598|F|0-65:G>A-65:G>A	TGCAGACATTGTCAGTGCCAATACTGATAGTATGTCTGCCAAGTATTTCTGCAATTGAGATGCAAGTAG
39725202|F|0-27:T>A-27:T>A	TGCAGGTTGTAATCTCTTTCCGTTTCCTTAGCAATAATTTATATAAGAAATTAC
39715552|F|0-33:G>A-33:G>A	TGCAGCAGCCATCGGAGTCATCGGTCGAGGTGGGAACTCGCATATGCATTGGATAAAAGTCTGTGGTTT
39717476|F|0-65:A>G-65:A>G	TGCAGGTATGATGTCTGCATTTTTTGCCCCCACAAAAGCTGAAATTTACAATAACATTTTGGTAAATGG
39719973|F|0-5:T>C-5:T>C	TGCAGTAGTCTCCAACCAGGTAAAATGTTTTTCTGCATCAAGAAAATCTTAC
39719948|F|0-7:G>A-7:G>A	TGCAGCAGCATTATAAATGAAACAACTTTGATAAAGTTTAGGTCTAGTGCGTTAC
39714933|F|0-18:G>C-18:G>C	TGCAGATCTTCAACGCTTGCAGAATGTCCGACAAATGGAGCATTATCAACATTCCATGAAGTGCCAGAT
39728494|F|0-18:A>C-18:A>C	TGCAGTGATGTCGTTGTTATTGGTATTTTCGTTGCTTGTTTGTGTTTTAC
39719431|F|0-32:C>T-32:C>T	TGCAGAAGTGGACCATAGTCAAATTAGATTCACCTAATGGAATAAATTTAC
39715064|F|0-12:G>A-12:G>A	TGCAGATGTTTCGAAAATTCGGGTTGGAAAGCTAACTCCATATGGGATAGAAACTCTCAGAAATATAGA
39721224|F|0-7:A>G-7:A>G	TGCAGAAATATGAGATATATTGTTATTCCATTCAACTGAGACTATAAGCTGTTTTCATCTTTTTCATTA
39729510|F|0-9:A>G-9:A>G	TGCAGTTCTAGTTGATTCATATATGAATTGCCCAGATAATTTCTATCAAGAGAAGGATCATTTAC
39725149|F|0-21:T>A-21:T>A	TGCAGGGTGCGGTTCGGAGGCTTGTTGTATATATAGAGATTAC
39727471|F|0-33:T>A-33:T>A	TGCAGATATCTTCTCTTCATCTGATCCTTTTACTGCTACCTCAAATATTTTCTCCAGTTCTGCTAAGCT
46756740|F|0-52:A>T-52:A>T	TGCAGGTCATTGAGCCTCTTGCAAGTGTGTCTTCAGCCAACTTTACCTGAAAATCAAAAGAAATTCACA
39725167|F|0-13:C>A-13:C>A	TGCAGGTATAATGCTTATGTACAAACTTGTTGTGATTAC

39718451|F|0-45:C>T-45:C>T	TGCAGTGGTAGAAGACATATACAGACGAAGGCAGCCACTGCCATCCATGGATGCTATCTATTTCCTTCA
39720445|F|0-15:C>T-15:C>T	TGCAGGGCCCAAATACCAGATATATTTCTAGGAAAAATGTTTGTTAC
39726516|F|0-11:A>T-11:A>T	TGCAGGTAGTTATGGACAATGGTATATTGCAAGTGACACTGTCCAAGCCAGATGGAAATGTTACTGGGA
39717203|F|0-49:A>G-49:A>G	TGCAGGCTGCTTTATGTGGTCCTCTTGGTTCCTTATGCAATCAAGGGTTAGCAAGGTTTATCCATGTCA
39728506|F|0-8:G>A-8:G>A	TGCAGAAAGTAATTGTGAAAGTGATGAGAACATTATAAAACCACACTTGACTTTGAAGAAGATTTTTAC
39724959|F|0-10:A>G-10:A>G	TGCAGCCATTACTGGTACAAGAAGACCAATCTTTAC
39718894|F|0-20:G>C-20:G>C	TGCAGTTTCTTCTGCTTCCTGATCAATATAATCTTCACCTACAACCTTTTGGCTAAGTTGCAACTTCCT
46764323|F|0-32:T>A-32:T>A	TGCAGGACTCACCAAACTATACATAGATCACATGGGAGCAAGCTGATTCTTGTAAAATAAGCTGCTGAT
39718262|F|0-67:A>T-67:A>T	TGCAGTGACAAGTCCAGATGTTACCACTTAGACCAAACTAATAGACATGATAGATAAATGAGTTCCAAC
39728044|F|0-50:A>G-50:A>G	TGCAGGACGAGCAACTAATAATTACACCACAACACAATGCTATGAGCTGAAAGGCTTAC
39726938|F|0-12:C>A-12:C>A	TGCAGAAACGTTCTCGCCAGCACCTGTCTTTCTAAAATCTTAC
39728526|F|0-25:G>A-25:G>A	TGCAGACGTTTCAGATACATTGACTGTCTTCTACGAAGGTCCATAGAAAGATTCTGAAGCTCTGTAGCA
39714463|F|0-13:G>T-13:G>T	TGCAGAGATGGGGGTTCATACATTCTTTCCATGCAAGACTTCATGTCTAAATAAGTGCGTTTTTGATGT
39717346|F|0-17:G>A-17:G>A	TGCAGGGGCCAATCAATGTAGTATTCAAGAGCAACACCATCCCAGCCATCAAGAGACATATCTGAAGAC
39719483|F|0-11:G>A-11:G>A	TGCAGAATTGGGAAAAAATGGGCAATTTTTAC
39718573|F|0-15:G>A-15:G>A	TGCAGTTATAATCACGTTGAAATCGCCATCAATGCCGATGGTTCCCATGGCCGACTCCAGAGCTTCTCT
39717211|F|0-47:T>A-47:T>A	TGCAGGCTGTTCAAGGTCAATGGGCAGCCTATATTTATTCGTGGTGGTAATTGGATATTGTCTGATGGG
39715658|F|0-10:A>C-10:A>C	TGCAGCATACAGAAGTTCCCATTCCTAAACCCAAGATAGATGAGGTTTTGGTAAAAGTGGAAGCAGTTA
39722845|F|0-33:C>T-33:C>T	TGCAGCTACAGTGGTGTTCAACAATATGCATTACACTCTGCCTCCTTGGTCAATAAGCATCCTTCCCGA
39727360|F|0-58:T>C-58:T>C	TGCAGAAGAGGCATAAAAGCCATGCTGTTCTCTAATTGTTTGACATTATTTTGCTGCCTTTCTTGCACA
39724105|F|0-64:A>T-64:A>T	TGCAGTGGACTTCTTCATCGGAATCAAATGCACAAGGACATTCAAGTGTTTCAGCTGAAAAGATAGATG
39715678|F|0-67:G>A-67:G>A	TGCAGCATATTCATTTGTACATGTTTAGAACAAGATCCTAAATAATGATGAAAATGGTGGGAGTACTGT
39713268|F|0-63:C>T-63:C>T	TGCAGAGACCCTTCTGAGGTACGTTCTTATTTGGATTTGGGTAAATTTGAAAACGATTACTGACCCTAT
39715385|F|0-21:C>T-21:C>T	TGCAGCAATGGATCCAAGAAACAAAATTGGTAGCTCAGGCTTGTTCAGGGAAGCTAATCGAGTAATTGA
39720061|F|0-20:A>G-20:A>G	TGCAGCCTAAGCTAGCTAACAGCAGCTTAC
39720874|F|0-27:C>G-27:C>G	TGCAGTTGTTGCGGACTCTCAAGGTGTCAAGGTAATTAC
39720003|F|0-41:T>C-41:T>C	TGCAGCATTGGCAGAAAAATATGCCTGGAAGAAGTGACAGTTATAAGCTCAAATTTTAC
39714181|F|0-13:C>T-13:C>T	TGCAGACATTTGTCGAGGTGGAACTTGATGAGCTAAGTAGGAGGACTACTGTAAGATCAGGCTCCAGCC
39717619|F|0-43:G>C-43:G>C	TGCAGGTTCCAAGATGCTCCTCTTCTCCTCAAACATATCCTACGATATGAGAACAAATATCAAAAACCT

39713333|F|0-30:A>G-30:A>G	TGCAGCCTTCTCACCTAATTCTGTGTCACCATGAATTCGGCTTGCACCAAGTAAAGCACCCCAAGTTGC
39717951|F|0-65:G>A-65:G>A	TGCAGTATGATTTCCCGAAGCAGGAGACCGTTATACAATTTGTCATTGATGCCATTCAGGCTGAAGCTT
39719849|F|0-26:A>G-26:A>G	TGCAGCAAATAAGAAAAATCATTGAAAGAAGTGGCTAGTTAC
39723635|F|0-33:G>T-33:G>T	TGCAGGTGGAATCAACTATTGAGTATATTTGTCGTCATGAAGGTCACGGTGCTATTCTTGTATTCCTTA
39722185|F|0-45:T>C-45:T>C	TGCAGATTGGCATGGAAAACTTGAGTCAAGTGGTTTCTCTAAGAATCTGAAAAGAAAAATGTAATTATA
39726612|F|0-54:T>A-54:T>A	TGCAGTAGCATTCAAAGGCTCTTCTTCTCTTCATCAAGAGTTCGGAAATTGGAATAATCCGGAACGGAA
39728994|F|0-18:T>C-18:T>C	TGCAGATCTCATTGCCGGTTAGACCTGCTTCAAAGGCAGAGCAAATGAGAGAATGCTTGCGATCTTTAC
46761047|F|0-14:T>G-14:T>G	TGCAGTCAATTAACTTTTGACCAAAAACATTAC
39715273|F|0-20:C>T-20:C>T	TGCAGCAAATTACTCACTCGCTCTGAGAGATTATGGACCTCAGCAGCACGAGATCTTTTTGTAGAGGTT
39716532|F|0-49:A>T-49:A>T	TGCAGCTTATAATTTTGTGTCCATTGCAACTGATCTTTACCGAAGTTACAGCTGCTGAATGATCAACAA
39717022|F|0-30:G>C-30:G>C	TGCAGGATTCTCTTTTTCTTTTTTTGCTTTGCAAGGGCAGGAATACAATATTGCGGATAAGTATAAATC
39720394|F|0-25:G>A-25:G>A	TGCAGGCATTGAGGAGCAAGCTGAGGGAGATGGGGATGTTAC
39714672|F|0-59:T>C-59:T>C	TGCAGAGTCTCGTGCTTCAAGACAGCATTTATCCGAAATTTGGTGAAGATCCATGTAGATCGTCCATTT
39714642|F|0-51:T>C-51:T>C	TGCAGAGTACAGATTGTACGACCCGAAACACGAAACGGCAGAGACATCTAATAAGCTTTTCACAACAAC
39722086|F|0-36:T>C-36:T>C	TGCAGATGCCCTTTCTGCCCTTGTAAAATATGATCCTGAATATTTTGCAAGCTATGCTGTAGAGAAGTT
39721114|F|0-37:A>G-37:A>G	TGCAGAAAAATTTACCTTTTCAAATTAGCTTTCAGTGAATATCCACAGTATCAGCTATTTGCTCTTAGT
39714137|F|0-37:G>A-37:G>A	TGCAGACAATTTACTCTGCTCGGAGGCAGTGGGTTCCGGTGTATTTGCGGGACACTTTTTTTGCTGAAA
39718729|F|0-39:T>C-39:T>C	TGCAGTTGAGTGCTGTATTGTCAAGAACAACTTATTTTCTTGACCCTCTCTCTTTGTTTATTTGATTCT
39721928|F|0-8:C>A-8:C>A	TGCAGATACAAACCAACAAAACTACATTCAGTAATTGCCAAGAAACCCATAACGCAAACACTACCAGCA
39727759|F|0-8:T>A-8:T>A	TGCAGGTCTTATGACTGCGAAAGTTGTTTGTCTCATTGAATCACTACTTGAATACAGGTATCTGTTTCG
39717650|F|0-58:T>G-58:T>G	TGCAGGTTGCTGACTCATGGGACGCGCTCGATGGATGGCTCGACGCAATAAGATTAGTTTACACAATCT
39719356|F|0-35:C>T-35:C>T	TGCAGAACAGTCCATGGGGCGTGAAGCTTGAGGACCCAGATGTTAC
39717626|F|0-58:A>G-58:A>G	TGCAGGTTCTCAAACAACATTCTAGAAAAATCTGTTGATTATCAAATCATTTCTCCACAGGAGCTCGCA
39724997|F|0-25:G>A-25:G>A	TGCAGCTAGATTTTCCCCTTTTGTTGTTTTAC
39724921|F|0-18:C>T-18:C>T	TGCAGCAGTTCCACATCACCCTAGCTCTTTCTGTTTAC
46754114|F|0-27:A>G-27:A>G	TGCAGACAAACTAATAGAATAGATAGTATTGCCATCATCTGATACAATAAACTCCATCAACACTAAGAA
39720330|F|0-15:G>A-15:G>A	TGCAGGAGGTCGACCGGTTTGATGATTTAC
39721584|F|0-46:A>C-46:A>C	TGCAGACAGTTATACCCCTCTACATTTCGGTTATTTACTTTTATGTACTCGAATCGTCGCCGTGTAGTA
39718530|F|0-32:G>T-32:G>T	TGCAGTGTTTTGCTCAGGAAGAAAACAAACATGCATTTGGTGGCTCAGGATTTTCAGAGATTGGTGAGA

39715073|F|0-35:G>T-35:G>T	TGCAGATTAGAACTTCTCATGATGACCCCTCTGAAGTGGAGGATCAAAGATTCTCTACTAGTGGCAATC
46764836|F|0-12:C>T-12:C>T	TGCAGTAACGGTCCTAGACGGCAAGCAGCTACGAGACATCCACCTCTCTCCGCCGTTCTCCGATGCTGC
39717284|F|0-50:G>T-50:G>T	TGCAGGGATGCCATATCTTCCAATTCGATTCCAGCATTCCGTAGAGGTATGTCCTAGTGGGTTTCATAT
39719502|F|0-5:A>G-5:A>G	TGCAGACAGCTCAAATTCATCATGTTAC
39715388|F|0-62:T>C-62:T>C	TGCAGCAATGTATATAGGAGTATTTGATGGGTATCCAAGAGCGTTTAGAAAAATTCCGACCTTTTTTGG
39719923|F|0-5:C>T-5:C>T	TGCAGCACCTTATTGTTGGCTGGACCGATGCATCGAGGCGTTTTAC
39727148|F|0-30:A>G-30:A>G	TGCAGGCAACAAGGGCCCCAAAATTGTCTCACAAAGAAGTTCATCATCGGAATTAC
39714795|F|0-67:T>A-67:T>A	TGCAGATAGTAAAGTGCCTATTATCATGGTTGGTCCTGGAACTGGATTGGCTCCTTTCAGAGGTTTCTT
39723613|F|0-10:G>A-10:G>A	TGCAGGTGAAGGATGCCATTTTCAGGACGCTCATTCGGAATGGAATGTTTGATAATGCACACATCCGAT
39725072|F|0-29:A>G-29:A>G	TGCAGGACCGGAGGTATTGCTTCTTTCTGAGCTAGCACCTGAACTTAC
39719404|F|0-39:G>T-39:G>T	TGCAGAAGCCTGATATGAAAGACATTCATCTATAATAATGGTTAC
39717204|F|0-54:G>T-54:G>T	TGCAGGCTGGCGTCTTCATAGTTCCATGAACTGCATCTGTATGATGTGCTGCTGGCCATTTCTTGGGTG
46755145|F|0-7:C>T-7:C>T	TGCAGCACTGCAAAGCTGTGTGCCATTCGAGACCCATTCAGAAGCCCTGCCACACACAAAGTCATTCAC
39726181|F|0-60:G>A-60:G>A	TGCAGCCTTGGAGATGCCGTACGCTGGAAGCATTGTTGTCCATCCATTTGCTTCTAATCTGTCTTCCTT
39717752|F|0-50:G>C-50:G>C	TGCAGTAACTCAACATACCCTGTTTTTCTTTTATCATTTCTAATAAAAGGAACATTATTCACTCTCCT
39715029|F|0-66:T>G-66:T>G	TGCAGATGGGCTTCTAGCAGATATCATGAGTAAAAGTAAGTGAAGTGATTGCCTTACTTCATGTGCTAG
39722463|F|0-55:A>G-55:A>G	TGCAGCAGGACCTGGTCTATCAGAAGAATGTGGAACACTTGTACGCCTGTGATTGATTTTTCTTCAGTT
39722155|F|0-52:C>T-52:C>T	TGCAGATTCATCAACCTCACGCCCCAGAAGTCGCCCATCTCTCTCACCGGATCGTCCCTCGCCCATTCA
39722578|F|0-54:G>A-54:G>A	TGCAGCCAAAGAAGCCTCTGTTAGCAGACAAACCAGAAGGATGCGCTGCTTTGAGTATGTGATGCCTTT
39726814|F|0-39:C>A-39:C>A	TGCAGTTGACTATCTAACCTACGGCTCAGTGGATTTCGCCTTTGCTCCCTATGGACCCTTTTGGAAGTT
39716410|F|0-9:T>C-9:T>C	TGCAGCTGATTTCTTGATCATTCCAAGTAGATTCGAACCCTGTGGTCTCATCCAGTTGCAAGCCATGCC
39716523|F|0-12:A>G-12:A>G	TGCAGCTTACAGAACTGAAATGGAACTAAAGTTGTCTGCTCTGCTGACATAATTTATGATACCAAAAAA
39720396|F|0-6:C>T-6:C>T	TGCAGGCCAGCCTGATAAAGCTGTTAC
46754973|F|0-38:C>T-38:C>T	TGCAGCAAAATCAACTTGAGGGTCCGATTCCGGGTGAGCTTGGAAACTGTTCTAGCCTTCTTGTCTTCA
39718497|F|0-21:C>T-21:C>T	TGCAGTGTGCCTTGGTTTGTGCAACTAAATAGGCGAGAGGGTCGGTGTCTTTCCGGTAAAGCATCAGGC
39715254|F|0-34:C>A-34:C>A	TGCAGCAAAGAGGAGATTCGCGGGAGAACAGCCGCTCGAGAACGAGGATTTGTGAAGTAAAGACGAAGA
39714564|F|0-21:T>G-21:T>G	TGCAGAGGAGCCAGGAGGAACTAGAGAAAAAACGGCAGGAAAGGGAGCTGGCATTTGAAGAGGAGAGAG
39717969|F|0-61:A>G-61:A>G	TGCAGTATTCCACATTTTGATGACAGCTGATGACCAGAGTCTAACGGTGGCTCATTACCCTATTCAAAC
39716449|F|0-49:A>G-49:A>G	TGCAGCTGCTGTTCTTCTCCTTGCCTTTTTCTGCACTGCCATTGTCTCAATTCTCTCTTCATTGGCCCT

<table>
<tr><td>39714040|F|0-46:A>G-46:A>G</td><td>TGCAGAATGCTAGATGTGCAGCGGAGCAGACCCAGACTGTTACGAGAGAGGCTCCTACAATCACTGTTC</td></tr>
<tr><td>39717371|F|0-58:G>A-58:G>A</td><td>TGCAGGGTAATTACAGAAGTTGGTGTGTATTCTATTCTATATATAGAGCCAAAAGGAAGACAGGAATAA</td></tr>
<tr><td>39727306|F|0-54:T>A-54:T>A</td><td>TGCAGAAGCATTGTTGGGGAGCTAGATGAGGAACTTGATTCTAATTTGGACTTGTCGAAATTGAGAGCT</td></tr>
<tr><td>39713893|F|0-28:T>G-28:T>G</td><td>TGCAGAAGGGGTAAAAATATGCTTTTGGTATTCTCTTATAATTTACTAATTTTGCAGAGGTTAGTGAAA</td></tr>
<tr><td>39726050|F|0-32:C>T-32:C>T</td><td>TGCAGCAGCATCTTCACTATTCTTGTTCTTTCCGAGATTGACACCAAGAATTCCAGGACCAGCTTTACC</td></tr>
<tr><td>39724600|F|0-44:G>A-44:G>A</td><td>TGCAGAAAGTTTGTCTCAGCTATAGATGTGCAGACATGGATAGGGAGATCCCTGCTTTAC</td></tr>
<tr><td>39729038|F|0-47:G>A-47:G>A</td><td>TGCAGTTGGCATTGGCGCTTCGGCTTTCCTCAGAGGCCACTTGTGCTGATGATCCTAATTTCTTACAGA</td></tr>
<tr><td>39724288|F|0-51:C>T-51:C>T</td><td>TGCAGTTCTCAGATTAGCAGTCAGTGGTGCTCCAGTATAGTTGAATGGAGTCGGAGGTCTGTCTGGAAA</td></tr>
<tr><td>46766917|F|0-12:G>T-12:G>T</td><td>TGCAGAAATGCAGATCTAGTAGACATTAC</td></tr>
<tr><td>39714962|F|0-33:G>A-33:G>A</td><td>TGCAGATGATAATGCTTCTCTACCTTCTAAGGAGTCAGCAGCATCTTTGTCAAAATAAATGCCAGTAAA</td></tr>
<tr><td>39729265|F|0-62:A>G-62:A>G</td><td>TGCAGGGATTATCTACTGAGTGTACGAGGATTGGGTTTGAAAAGTGTGGAGTGTGTTCGTCTATTACAG</td></tr>
<tr><td>39717233|F|0-53:G>T-53:G>T</td><td>TGCAGGCTTTGATGTCGCAGCAGATTTTAGATCCTGCAAAGCAGAGTTTTCTTGCATTGCCCGTCTTTG</td></tr>
<tr><td>39717382|F|0-12:C>T-12:C>T</td><td>TGCAGGGTCATACAAATACATTGCCCCCTTCTCTCTTGAATGGGTTGGCTACAAGCGGCATCCTATAGT</td></tr>
<tr><td>39717611|F|0-35:T>A-35:T>A</td><td>TGCAGGTTCAGGGTTCAGAGGCTGGGCTTCAGCTATAGCACACTCTGCAAATAAAGAAGCTAGTTGATC</td></tr>
<tr><td>39728325|F|0-33:T>C-33:T>C</td><td>TGCAGGATTGTGTACACATATTCTTGAAGGTCATAGTGACGCTATATCTTCGGTCTGTATTGTGAATCC</td></tr>
<tr><td>39724337|F|0-60:A>G-60:A>G</td><td>TGCAGTTGCTCAAGCCGCCCTTGCTTACCAGCTCTACCAGAAGAAGTTCTCAGGCCCAAGATGGGAGGC</td></tr>
<tr><td>39719565|F|0-22:G>A-22:G>A</td><td>TGCAGAGAAGAATAGCTTGTTGGAGTTTCAGTCAAATTAGCCATCTTAC</td></tr>
<tr><td>39728866|F|0-52:T>C-52:T>C</td><td>TGCAGCCACGAATGCAATTGAATAAGTCCAAGATTCAGATAAAACAGGCCGATATTGATAATAATCATT</td></tr>
<tr><td>39717192|F|0-49:G>A-49:G>A</td><td>TGCAGGCTCGGTCACAGCGGTTGGGCGGAGTATTGGCGGTCGAACCCGGGCTGGAACTGGGCGCGGTTG</td></tr>
<tr><td>46756484|F|0-14:G>A-14:G>A</td><td>TGCAGGCTGCATCAGCCTATGTACTTTAGTGGAAAAATTATGGAGAAGACTTCTGTTCCAATTAGGAAA</td></tr>
<tr><td>39713586|F|0-23:A>T-23:A>T</td><td>TGCAGAAATAGAGGGTCAATCTGATTGTGATGATTCGTCTCCTATTGTCATAGTGAATGAGAATGATCT</td></tr>
<tr><td>39717983|F|0-9:G>T-9:G>T</td><td>TGCAGTATTGGTGTAAGAAACAAGCTGTGTTGAAGTATCTGTAGTTCAACCGTGGGGCTTGTGGCATAT</td></tr>
<tr><td>39721982|F|0-64:A>G-64:A>G</td><td>TGCAGATATTCCGAACTCTAGGCACACCAAATGAAACGATTTGGCCTGGATTTTCCAAGCTTCCAGGAG</td></tr>
<tr><td>39717669|F|0-40:C>T-40:C>T</td><td>TGCAGGTTTCAGCCTCTTCAGAGATGTCAGTTTCCATAGGCACTATATATAATGAAAATTCTGCAATCC</td></tr>
<tr><td>39718618|F|0-30:A>T-30:A>T</td><td>TGCAGTTCACTTCAAGTGTCTAGTTTTTCAAGGGCAAAAATTTCTCTCTCCGCAGCTCTTGGTTGCTTC</td></tr>
<tr><td>39716675|F|0-43:C>T-43:C>T</td><td>TGCAGCTTTGGAGGAGGAACTTGAATTGCAACGTAGGGAAATGCAGAGAATTATTTCAGATAACCGCAT</td></tr>
<tr><td>39721433|F|0-24:T>G-24:T>G</td><td>TGCAGAAGTGCCTTGTTCTGCATCTCCTTCGACATTATCCACCTTCTCACCGCTAACTTTCTTCTGATA</td></tr>
<tr><td>39725415|F|0-30:T>C-30:T>C</td><td>TGCAGTTTGTTTGAGATCAATGAATCAGCTTCACAAGAGTTTTTCCTTTTTCTTTAC</td></tr>
<tr><td>39723364|F|0-22:G>T-22:G>T</td><td>TGCAGGCGAATCTAGCGGCGGCGCGAGTGGCGGTGGCGAGCGGCTGGGTTGAAGAGGACGTGCATCGGA</td></tr>
</table>

39719829|F|0-26:T>C-26:T>C	TGCAGATTTTTATTTTGTTTTCGTTTTGGCAGAACAAGATCCGAATCTGTTAC
39722740|F|0-62:G>T-62:G>T	TGCAGCCTGCTCATAGAAGCAGTCTACTTTTAGTGGCACAGAAATTGTCCTCAAATGCTTTCGAGGCAA
39727018|F|0-20:G>A-20:G>A	TGCAGATAACCAAACTGGCTGTCATTGCTACTAAATTTAC
39715728|F|0-14:A>T-14:A>T	TGCAGCATGACAAAACAAAAACTGATCATAACCAACAAAACCAACCAAAACTGAACTGACTGAAATGGC
39723133|F|0-48:C>T-48:C>T	TGCAGGAACAAGGTGCAATCTCAAATTGACTGTTATACAGGATAGGTTCTAGATAGAGTCGTTATTAGA
39716811|F|0-7:G>A-7:G>A	TGCAGGAGTCGAATTGGAAGGTACAACTTACTCCCAGATGAAAAGGTGTGACATAGTTCAAAATATAGA
39718809|F|0-57:C>T-57:C>T	TGCAGTTGTCAGCATATGAATAGTGGACCTGACGACTTAGCCCTCTCTGTTTCATGCCAAATCTTGTCT
39719804|F|0-9:A>G-9:A>G	TGCAGATTGACAATGAATATACAGAGTTAC
39722026|F|0-56:T>A-56:T>A	TGCAGATCGCCGTTTTTTGATCCTTCCGTCGCTTGTGTAAGATATTGAAGTTTTCTTTGATTCTCAAAA
39727751|F|0-15:C>T-15:C>T	TGCAGGTATTATCCTCCATCTCTTTTGCTTTATGTTTGTTTTTCCATTATCAGTTTTATATGTTGAAAG
39716810|F|0-12:G>C-12:G>C	TGCAGGAATCCTGCCTTGGTCCAACGTATCCAAACAAATGTTTATATAAAATATTGATTTTATATAAAA
39721075|F|0-16:A>C-16:A>C	TGCAGTAAATAGTATAATTGTGAAATGCTACACTAGGACTACTACGCTACATTATTCTGAGCATAGACC
39715162|F|0-50:G>A-50:G>A	TGCAGATTTACTGCGAACTCTGAGTTGTAAGTACCACTTATGTTTCTGATGTTGATGTCACTCAGCTTC
39716624|F|0-6:T>G-6:T>G	TGCAGCTTGGTACAAGTGTAGTCCCTCTTTTGTAATGACTCTGTATGTGATATATGTGCTAATTGGATG
39726288|F|0-39:T>A-39:T>A	TGCAGCTGATCCCAAGTTACTTGTTTATCTAAAATCGTATCGCAACACAGTTCCTGTGCCAAGGCATTG
39717680|F|0-37:T>A-37:T>A	TGCAGGTTTGTGGGTTTGATCTTCTACGATGTGAGGGTCGCTCATATGTTTGTGATGTAAACGGATGGA
39717706|F|0-28:T>C-28:T>C	TGCAGTAAAAGATGATGAAGCATGACAATATTTGGGGAGCCTAAGCCTTTTGAGCAAAATACATGCTTT
39714750|F|0-65:T>A-65:T>A	TGCAGATAATCAAGATAATATTTCAAAAAACAGCTAATTTCCTAGTACAACAGCAACACCACTAATAAA
39715606|F|0-51:C>A-51:C>A	TGCAGCAGGGCTTGAAGGCCTTCTAAAGAAGGTTGAGGAGATAATGAAAGTCACACCCAATAGCTATTT
39727786|F|0-57:A>G-57:A>G	TGCAGTAACTTCTTCTCGTCAAAGTGCTGATGTCGGAACAGTCATCTCCAATTTGAAATCTGTTTTGAA
39714047|F|0-50:A>C-50:A>C	TGCAGAATGTAATATGGAAGTAAGGCTTTTTTCACCTTTTCATAGTTGTAACCTTTGAAGTTTTACGCA
39728023|F|0-38:A>T-38:A>T	TGCAGCCTACAGAAAAACATTCATAAGACAACTGAATAAGGAACAATAGATTAC
39724254|F|0-56:A>T-56:A>T	TGCAGTTCATTGCAGCTTGCAAGAAACCTCCAGTGTTTTGTATCATCACGGAGTACATGGCAGGGGGTT
39724576|F|0-18:G>A-18:G>A	TGCAGAAAAGAAATTATCGAACGAAGAATTAC
39714992|F|0-50:C>G-50:C>G	TGCAGATGCCATGTCAGAACTCAGAAGATAAGTTCATAAATTTTCCACCCCATAAAAAGATGGGAAAAA
39720154|F|0-16:C>A-16:C>A	TGCAGCTCAGAAGCAACTATGGACGCTTAC
39713345|F|0-46:T>C-46:T>C	TGCAGCTTAGTTGGGAAAAAGAAAAACCTGAAGATCTCAATGATTGTGGTATCTTATCTAGAGTCTGAA
39715317|F|0-15:A>G-15:A>G	TGCAGCAACTTCCTCAGTGATGTTATTATGTCTGTTTCATCCTCAATGGCTCGGCGGGAGCGAAGTTCT
39728224|F|0-12:C>T-12:C>T	TGCAGATGGGACCCGAGCGCGCTATTCTCGACTATACAGAACTTTGGCCATGAAAGTCCCATCGTTACA

<table>
<tr><td>39715277|F|0-64:C>T-64:C>T</td><td>TGCAGCAACAAAAGATACTTGCATGGCAGCTTGCACGCCCCTCAGGGACGTCCGAAGAATGCGTCGATT</td></tr>
<tr><td>39720795|F|0-21:G>T-21:G>T</td><td>TGCAGTTACAGCCTGTCAGATGTCAAGACCATTATTAC</td></tr>
<tr><td>39717055|F|0-6:C>T-6:C>T</td><td>TGCAGGCAATCTTGCGATCTGACCTTCCACTCTATATGGTACCATTTACAACCAGAGATCTTTTATTCA</td></tr>
<tr><td>39719336|F|0-8:T>C-8:T>C</td><td>TGCAGAAATTACATATAGACTACTAAATGAACAAATTTAC</td></tr>
<tr><td>39720625|F|0-22:T>C-22:T>C</td><td>TGCAGTATGCAAATGACAATGCTTTGCTTCTTGCCAAGGTATTTCAACCATTAC</td></tr>
<tr><td>39728091|F|0-29:C>G-29:C>G</td><td>TGCAGTATTCAAGTTCTTATAAACGGTAGCTAACAAAAGAATTAC</td></tr>
<tr><td>39716242|F|0-25:C>A-25:C>A</td><td>TGCAGCTAAGTTTCTTTTATCCTATCAAATAAAATACTAGAATAACATTATGAAAATTTGTATTATATA</td></tr>
<tr><td>39724383|F|0-18:A>G-18:A>G</td><td>TGCAGTTGTTGCGTAGACATCGTCGAGTAAATGTGAACCAGGGTTCCCTAGGTCATGAAGAGGTATTGA</td></tr>
<tr><td>39729494|F|0-48:T>C-48:T>C</td><td>TGCAGAGACATTACCCAAACATTTGGAAAATCAGAACACAATCTTCTCTGGAAGCAAGGATTTTACAGA</td></tr>
<tr><td>39721142|F|0-58:G>T-58:G>T</td><td>TGCAGAAAAGTTTCATGCTACTATTCATTTTCTGACGCAATCCTGCAACCACTACTAGGAAATAAAATT</td></tr>
<tr><td>39714993|F|0-16:C>T-16:C>T</td><td>TGCAGATGCCTTATGCCGAAGCCTACGAATTGGTCCAATGGAACCAGTTGCCTCATCTAAAGGATAGTT</td></tr>
<tr><td>39715691|F|0-15:G>A-15:G>A</td><td>TGCAGCATCAGACAGGTAAGGAAGGTTGGATAGATCTAATTCTTGCAGTTCTATTTTAGATAAGCCATC</td></tr>
<tr><td>39718379|F|0-30:G>A-30:G>A</td><td>TGCAGTGGAAAGAAGAGTAAGAGAAGGAAGGCTCCTCGTCTAAAGAGTATGCAATACATTATTCAGTTA</td></tr>
<tr><td>39718155|F|0-23:C>T-23:C>T</td><td>TGCAGTCTATCCTCATTCTTCATCTTTGTTGGACGCAGTGCATTTACTTTTGCCTTCATGCTTTAGAGT</td></tr>
<tr><td>39720504|F|0-30:A>G-30:A>G</td><td>TGCAGGTGAAATTGTAATGTAATCTTATCAACATTATTGTCTCCTTTTCATTAC</td></tr>
<tr><td>39716447|F|0-54:C>T-54:C>T</td><td>TGCAGCTGCTGGGGCTTGTACAGTCGTATTGACGCAGGTGTAAATAACTAACAACACTTTTTACTTATA</td></tr>
<tr><td>39724202|F|0-32:C>T-32:C>T</td><td>TGCAGTTACGGAAAAACTGAACGTGGTGAGATCGCTGCTAAATCTAGCAAATGAGGTCTAATGTGCAAG</td></tr>
<tr><td>39713706|F|0-43:A>G-43:A>G</td><td>TGCAGAACCCATTTCGTGGCTTCTTGCAGGATATGATATCAGAAAAGGAGTCAACTAACTGTCCAGCCA</td></tr>
<tr><td>46775562|F|0-6:T>C-6:T>C</td><td>TGCAGATTTCCCACTCTGCAACCATACAGAAACGTTTTAC</td></tr>
<tr><td>39718990|F|0-52:T>C-52:T>C</td><td>TGCAGTTTTTTATTCGAAGCATTGTGAATAATTTTATTAGAGGTTGCTATGTTCGTCTCTTGATCGATG</td></tr>
<tr><td>39720481|F|0-11:T>C-11:T>C</td><td>TGCAGGTATCCTATCCGCCAATTTGTTTCACTTTTTCTTCAGGAACCTTTTTAC</td></tr>
<tr><td>39717543|F|0-27:C>T-27:C>T</td><td>TGCAGGTGCGTCCCATCTAAGAAATGTCTTTTACAGGATGGGACTAACTGACAAGGATATTGTGGCATT</td></tr>
<tr><td>39715661|F|0-12:C>G-12:C>G</td><td>TGCAGCATACTTCTGTTTACCATAAGTAATGCCAGAAAGTGTTGAAGGTGGATCAGGTTGCTCCGGACA</td></tr>
<tr><td>39720067|F|0-51:T>C-51:T>C</td><td>TGCAGCCTCAACTGCTGTTTATGTGTACTTGTACTCAGTATACTACTACTATGTTAC</td></tr>
<tr><td>39713732|F|0-34:T>G-34:T>G</td><td>TGCAGAACTCTCTTGGCCTCGTTCATGCGGCCTTTGCTGACAAGCCAACGAGGAGATTCAGGGAGAAAC</td></tr>
<tr><td>39717164|F|0-55:G>T-55:G>T</td><td>TGCAGGCGCTCTGAAGAAATTCGGTGCAGATGTTGAGTGTGCAGATAGCGGGAAAGCTGCCCTGAAGTT</td></tr>
<tr><td>39727220|F|0-8:C>T-8:C>T</td><td>TGCAGTCACTTCCTAAGAGAAGAAGTAAAAAAACACATTAC</td></tr>
<tr><td>39727694|F|0-66:T>A-66:T>A</td><td>TGCAGGATATCAACTTGATATCGTCATTCTAATTCACTAAATTCATAAGATAATTATAATCTTCTCTCA</td></tr>
<tr><td>39715589|F|0-6:A>G-6:A>G</td><td>TGCAGCAGCTTGTGCTGGCAGATATGGGATTCAGGCTTGGCAGGCATTCAAGGCAAGGCCACCAACGGC</td></tr>
</table>

<table>
<tr><td>39723577|F|0-36:A>G-36:A>G</td><td>TGCAGGTATTCTACTATCTTAGTGAGCTTACCTTCAATTTATAAGATCAACATTTCACTACTTGTTGGC</td></tr>
<tr><td>46753686|F|0-11:C>T-11:C>T</td><td>TGCAGAAAGTACGATTGGCTTCTGCTGCATACCTGCAACTCCCATGACTGCTGAAGAGTGCAGGTTTCT</td></tr>
<tr><td>39726462|F|0-65:T>C-65:T>C</td><td>TGCAGGGATGTATTCTGCATTACCTTATGCCCTTGCTCAGGTATGTTGATGGCTTACAAATTCATTTAC</td></tr>
<tr><td>39715734|F|0-38:T>C-38:T>C</td><td>TGCAGCATGCCATAATAACTCAAAGACACATGCCTGAATCCCGCGAGATCGAACCGTTGACTCCACTTC</td></tr>
<tr><td>39720775|F|0-27:A>C-27:A>C</td><td>TGCAGTGTCCATATCTGTAACACCGTCAACCGCCTTAC</td></tr>
<tr><td>39714507|F|0-25:A>G-25:A>G</td><td>TGCAGAGCCAGCGTTTGAGGTGTGGAAATTTGCTTATTTTTACACATTTTGTTCATTCATGTGTTTTAG</td></tr>
<tr><td>39717609|F|0-17:C>T-17:C>T</td><td>TGCAGGTTCAGGAAAAACTCTGCCTAGCTGCTTCTGCCGCAGAACATGAAGATCTCCTCCAGGACAATA</td></tr>
<tr><td>39717902|F|0-47:T>A-47:T>A</td><td>TGCAGTAGCTCTTCAAATATTGTTTCAAGTCCAGGAAGTGGTATAATTTCACTGTCACTTCTAGGTCCG</td></tr>
<tr><td>39715726|F|0-6:A>G-6:A>G</td><td>TGCAGCATGAAATGACTTAGACACCACATATAGAATCGTCCTTGGCAAAAATCAGTTCACCAGACTATT</td></tr>
<tr><td>39728073|F|0-23:A>T-23:A>T</td><td>TGCAGGTCGCTTACAAACTTGACAACTGACATACATCAAAATAATTAC</td></tr>
<tr><td>39716650|F|0-30:T>C-30:T>C</td><td>TGCAGCTTTCAATACAGGCTTGGGGGCAGGTTTGAGAAGAAAATCTGTGTTATCATCGTCTTTACTTCT</td></tr>
<tr><td>39715468|F|0-58:C>A-58:C>A</td><td>TGCAGCACTTCTAAGGTCGCTTTTCTCGCCTTTTCCTTGTCTTCTTCACCTTCCGCCACAAAGACTCCC</td></tr>
<tr><td>39714025|F|0-39:G>C-39:G>C</td><td>TGCAGAATCTTCTTCAGTTGCCGGAAGCAATCGTATACCGAGGCCCATCTTCCGAGAAACATTTTCTTC</td></tr>
<tr><td>39716663|F|0-14:C>T-14:C>T</td><td>TGCAGCTTTCTCATCTGCCTTCTGCTGCTGTGACCTATCTCCAGTTGATGAAGCACGGGAAATCTGTAG</td></tr>
<tr><td>39715094|F|0-20:C>T-20:C>T</td><td>TGCAGATTCAATCTTCATGTCAGAAGAGAAAATTGAACAAATAATGAATCATTATAACCAAATCCAAAA</td></tr>
<tr><td>39728718|F|0-50:T>C-50:T>C</td><td>TGCAGATATATAGTTCCATTGGCATGAGTAAAGATTCATTCTTAGTGTCATACAAATATCATGGTTACA</td></tr>
<tr><td>39718961|F|0-55:T>C-55:T>C</td><td>TGCAGTTTTCTTTTTCTATGACCTTTCTCCTATCAAGGTTGGTTTTTCTTACATGTTCAATATGTGATG</td></tr>
<tr><td>39719781|F|0-32:A>G-32:A>G</td><td>TGCAGATGTTCTAAATGAGAAGAATCGAAGAAAAATTGCGGGATGGGCCATCTTGTTAC</td></tr>
<tr><td>39727143|F|0-9:A>C-9:A>C</td><td>TGCAGGAGAAGTCCCAGTCTTTAGTTGAAATTTCAGGTAGAAGAAAATTATGTTTAC</td></tr>
<tr><td>39713332|F|0-46:T>G-46:T>G</td><td>TGCAGCCTCGTTCAATCCCTTTGAACAATGGGAATACGGAGGCTTCTGGTGCGGCAGTAAAGCCAAAGA</td></tr>
<tr><td>39713906|F|0-51:G>A-51:G>A</td><td>TGCAGAAGGTCTAGAGGAAGCGGTTTCATGTGCCTAAGCTTTCTCGTTTCAGCATCATATCTGCATGGG</td></tr>
<tr><td>39723609|F|0-60:T>C-60:T>C</td><td>TGCAGGTCTTCTGTTTCCCTTACCACTTCCGTCCTTTCGGATTTCACTTTCCTTGTATATTAGGCAATT</td></tr>
<tr><td>39722397|F|0-66:C>T-66:C>T</td><td>TGCAGCAGAATTAGCAGAAGAGGAAAACCGAGCTAGAGAATTGGAAGAGAAGAATTTGATTATCGACCG</td></tr>
<tr><td>39723260|F|0-67:T>G-67:T>G</td><td>TGCAGGATGCTCAGAAAGAATCTGAGCTTACTTCTGAAGCAACCGAGAGGGAACTTGAGTTAGAAGCTC</td></tr>
<tr><td>46767178|F|0-14:G>A-14:G>A</td><td>TGCAGCTTTCTTTCGTGTTTGTCTTTAGGGGCTCTTAC</td></tr>
<tr><td>39717106|F|0-63:T>G-63:T>G</td><td>TGCAGGCATTTTCTATAGCACTGCTTGCCAAGGAGGTGGACAGGAGACTACTGCGTGAGCCTCTTTTGA</td></tr>
<tr><td>39713998|F|0-35:C>A-35:C>A</td><td>TGCAGAATCATCAGGCTGATCTAGCTCAGATTTAGCAACACCCATGCTTATAATTTGAGATTCTCCTAT</td></tr>
<tr><td>39722780|F|0-9:C>T-9:C>T</td><td>TGCAGCGATCTTCTGATTCAGCTCTTCAATCTCAAACAGAAATTTAGATTTTTCAACACCCCAGGTTTC</td></tr>
<tr><td>39713863|F|0-54:G>T-54:G>T</td><td>TGCAGAAGGAACTGAAATCGTCAAAACCATCGAGTTAGAGTTGCACAAGTATGGGAACGGAAACGGGAA</td></tr>
</table>

39716961|F|0-7:T>A-7:T>A	TGCAGGATAGGCAGAGCCAACGTAACTGGCTGGAGGCATGCCATAGTTTCTATTGGCCATACCCTGGCC
39715158|F|0-45:A>T-45:A>T	TGCAGATTGTTGGAAGTGTTGTTGAAGGAACAATTCTTAGTGTTGACAAAAGTTACTGGGGTGGCGATG
39715381|F|0-51:T>C-51:T>C	TGCAGCAATGCTCTCACCAGGGAAAGATGGAAAACTTGTAGAGATTTCAGGTCCCATTGACAGCAACTA
39715818|F|0-66:G>A-66:G>A	TGCAGCCAACCAACCATCGACCAGCTCAGCAAACTGTGAATCAGTCACTGTAAAGAACTGGGCCGCGTG
39716696|F|0-68:A>C-68:A>C	TGCAGCTTTTTATGGCCTCTCTTCTTCTCTTCATGTGCCCCCACATTCCCTAAAATGACTTTGAGAGAA
39715455|F|0-63:A>T-63:A>T	TGCAGCACTAAAGAACCAGCTTGGAACTCTGTGAGTACTCAAACCTCTATTGTTTTACAGCAAATTGAG
39716417|F|0-57:G>A-57:G>A	TGCAGCTGCAAGTGTGAGAGACAGGGGAAAGTGAGGGAATATTATGAGAAAATTGGGGAGATGGGTGGT
39719562|F|0-17:C>A-17:C>A	TGCAGAGAAATTCGCGGCGCTTTAC
39725062|F|0-17:T>C-17:T>C	TGCAGGAAGGAAAAGAGTGAAGAAGATTTGTTAC
39728582|F|0-9:T>C-9:T>C	TGCAGCTGCTCTTGCTTCTGCTTCATTGGTCTTCAGAAGATCTGATCCCACCTACTCCAAACTCTTACA
39715495|F|0-39:T>C-39:T>C	TGCAGCAGACAATGGAAGTATGGTTTCCTGTGAAGCAACTTCATTTTGAATAAAAGATATATATACACA
39720092|F|0-9:A>C-9:A>C	TGCAGCGAAAGGGAGAAATGTGTCGGTGTCGGTGCAGTGTTTAC
39725312|F|0-43:C>T-43:C>T	TGCAGTGCAACTGATTCATTTCCTGAAACCCCAGACTGATCTTCGGGAGCATTAC
39715201|F|0-63:C>T-63:C>T	TGCAGATTTTCAAAGACTTCAAGAATTCAAAGTTGGTGATTGGATCCGTGTGCGTAATACTGTCCCCAC
39724727|F|0-25:C>G-25:C>G	TGCAGAGACATCACAACCAATTGAACATCAGGAACTATGAGAAAAGCATTAC
39723569|F|0-17:A>C-17:A>C	TGCAGGTATAAGAATATAATGGCAAGCAAGACTACAATTTGTTGATTTCATTCTATATGCATTGCAATC
39715384|F|0-10:G>C-10:G>C	TGCAGCAATGGATACTTCAGCAACTGTAATTGGATGGGCAATGGCAGAGCTTATTAGGCATCCACATGT
39717057|F|0-40:G>A-40:G>A	TGCAGGCACAACTGGAGAAGATATTGTCTGCCAATGGACTGTCTGAAAATGTGTTTGAAATTGCATCAA
39716622|F|0-6:T>C-6:T>C	TGCAGCTTGGCAAAATAGCCGATCAAGCCGTAGATTTCGCTCTAAGCAAGTCTCTCCTAGAGTACCTGC
39715759|F|0-43:G>T-43:G>T	TGCAGCATTCAAAGGGGGAGCTGGTTCAAGGGATATTTATGAGGTTGTGAGAGAAATTGATGTTCTGTT
39714309|F|0-11:T>A-11:T>A	TGCAGACTTCGTTCCCTGATCAAAACTACCACTTTAGATACGGTAGTCGGTAGGGAATTGTTTCATACT
39716856|F|0-38:C>A-38:C>A	TGCAGGACGGCTAGAAAAATAGCTGGAATCTTTTTTTTCTAGACACTTTACTGTTAGAGAACTGGATGA
39722991|F|0-58:T>C-58:T>C	TGCAGCTGTCTCAATTAGGCGAACCATCTCATTTGTGATGACACGCAATTCATCCATGTGTTGTGTCTT
39717675|F|0-13:G>A-13:G>A	TGCAGGTTTGCAGGGGGACTGCTGTAATGCTTGTGTCTCCAGTTGATGGTACAGACGAGATTGCTAACC
39716203|F|0-8:C>T-8:C>T	TGCAGCGTCGAACCCACGGGAACCCACGCGTTTGACACCATAGCTACGCGAGCTTTCAGGGCGGGTTTA
39720315|F|0-18:G>A-18:G>A	TGCAGGAGAAGATGAAAAGAGGTCAACTATGGTTAC
39725165|F|0-36:T>C-36:T>C	TGCAGGTAGTTGTGGATATGATATGAAAAAGGTATATAAAATTCCAGTTTTAC
39716538|F|0-58:C>A-58:C>A	TGCAGCTTATGAACGCCGCCGCGTCCCCCGATATTCTGAACGCCACCGCCTGCTTCCCCAGCGCCCCCG
39719937|F|0-14:C>G-14:C>G	TGCAGCAGAGTGAACAAAGCGATTAC

39721082|F|0-25:G>A-25:G>A	TGCAGTGGTGGCGGCTGCGGCGGTGGTGACGTGGATCACGATGGTGGTTCTGCTGGCGTTCATTGGGAG
39713743|F|0-59:G>A-59:G>A	TGCAGAACTTTCACCACCATGAACGCACTAGTCGAATCGAGTCCCGATGTCGGCTCGTCGAGGAATAGG
39720095|F|0-11:G>A-11:G>A	TGCAGCGACCCGGGCAGCTAAAGAGGAAGAAGAGTCAAGACCTGATGTCTTTAC
39724648|F|0-25:C>A-25:C>A	TGCAGAAGGAAGCTCTCACAGATGACATTCTGACCTTTCTTCCTTAC
39725329|F|0-14:C>G-14:C>G	TGCAGTGGAAAGATCAGCTAAGTTTTAC
39725776|F|0-60:T>A-60:T>A	TGCAGAGGATTACCCAATACTTCTCGGTAGTTTGTAACAGCTTATGGTTTTTCCCCCTCATTTTATCTT
46765286|F|0-7:A>G-7:A>G	TGCAGTTACACTAAAAATCATCGTACGGGTGCTAAGAATCGGCTTCTTTATCTCACTTATCTCACTTTG
39727414|F|0-54:C>T-54:C>T	TGCAGACTGAAACTATTATCCAATTGCTGATGAGCTCAGGTGGGAAAATTTCTACGGAATTGGATCCAT
39720227|F|0-35:T>A-35:T>A	TGCAGCTTCTCCTGGAGCTCTTGGTCAAACCATTCTGTATATATTTATCCTCCTATTTAC
39727045|F|0-24:C>G-24:C>G	TGCAGATTCTGCACTGATCGGAAACTTCTGCCACGTTACCGTTTAC
46765363|F|0-27:G>A-27:G>A	TGCAGTTCCGTTCATATGATTTTATATGCATTATGTTGTTTCTTGTCCAAAAGTTCTAATGTCACTTTG
39722688|F|0-50:G>C-50:G>C	TGCAGCCGCGGCGGCCTGGTGGTAGTCTTGTTGCGACATAGAATTAGAATGAGTCTTTGAGTTGCAGAA
39713259|F|0-45:C>T-45:C>T	TGCAGAAGATCACATTCAAGGAGACCCAATGGACGAGTGTCAGTCCGATCCACAGATCTCAGAGACAAA
39723457|F|0-51:T>C-51:T>C	TGCAGGGAGTGCTTGTTCAGTAAAAACCCAGTCCTTATATAGAACAGCTGATATCTCCATGGCATATTT
39713827|F|0-41:G>A-41:G>A	TGCAGAAGCAATTGCTGGGTTACTATCTTCTGCAAGTCTCTGCAAAACCAACAAACAAGCATCAGAGAC
39713456|F|0-12:A>T-12:A>T	TGCAGAAAATATAGCAGTGGAGGCATTAGCTGCATTTCAAAGTGCCTTGAAACACCACATGCCTTGCAC
39728010|F|0-21:G>A-21:G>A	TGCAGCAGATGAAACCCAGAAGTAGACTATACAATTGAGGCAGAAAAATTAGATATTTAC
39729454|F|0-42:A>T-42:A>T	TGCAGAATTAGGTACTGTCTTTACAACTCCATAGATATGCTGACCACTGTTATGTTTATCATTAC
39727973|F|0-43:A>G-43:A>G	TGCAGAAATTGTGACCTGAACTTGCTGTGCTGTATGCTTTTGAAACCCAAAAATTAC
39716773|F|0-33:T>C-33:T>C	TGCAGGAAGAACTGATACTTGGAGACATCTTTATGCAGGATTCACGGTCTGGCTTTCTCAAACTGAGGC
39717283|F|0-50:T>C-50:T>C	TGCAGGGATCTCATTTGTCTCTGCCACACCTCTTCCATTGACTTCATTTTTAGTTCATACTCTGACCAA
46763530|F|0-24:G>A-24:G>A	TGCAGCAGCGGCAAAGCAAGCGAAGAATTCCAAAAAAGAAACTCCAACATTGTCGGCTACTAATCAGCG
39717036|F|0-58:C>A-58:C>A	TGCAGGCAAAAAATCTAACCTTCTTGGGCTTCGAACTTCCCAAAGCAAGGAGAACAACCTATATTTTTA
39724979|F|0-11:C>T-11:C>T	TGCAGCCTCATCTCTCCATTCCAATTGCTTAC
39725864|F|0-38:G>T-38:G>T	TGCAGATCTGATGCTTGGACTGCAAGTTCAATTCCACAGAGAAAAAAGCCAAGCCATTCAGAACATCAA
39723737|F|0-62:A>C-62:A>C	TGCAGTAAATATTGTACATTGCTCTTGAAATCCACATCTGAGGTTGATCCAGGATTGGTAAGAATGAAC
39719448|F|0-33:C>T-33:C>T	TGCAGAATATCGCCCGAGTCGATTGCTGATTACCTCTGTACTTAC
39717201|F|0-62:C>T-62:C>T	TGCAGGCTGCGCCATAGAGGTCCTGACTGGAGTGGTTTGCATTGTTTTGAAAACTGTTATCTCGCTCAC
46753523|F|0-41:C>A-41:C>A	TGCAGAGGCTGTTTGGCGGCCGAGAAAGTGAGGGAATCCGGCGGAAAATTCAGGCTGAGGCTGAGCTGG

39728250|F|0-35:A>G-35:A>G	TGCAGCAGGGGAATGTGCGTGGCGTGTCCGAGCCCAAAGGGGCTTCTGGGGTGGAGCGAGAGCTGTGCG
39717248|F|0-27:A>G-27:A>G	TGCAGGGAACTCAAATTAGTGACTTTCAATATCAGCAACCAGAAAACAAAAGAATATAGGATACATCAA
39716969|F|0-16:G>C-16:G>C	TGCAGGATCAGAGTACGGATAACGATGCTGACTTATCATCTTCGAATATGATTAGTTTCGAATTATCCA
39723159|F|0-57:T>C-57:T>C	TGCAGGAATGCGTAAACACTGTGCTACATGCCAAGCATTCATTTGGAAAAGAGCATGTTTCCCCGAAAC
39716109|F|0-17:T>C-17:T>C	TGCAGCGACCGGTACTGTATTTTGTTGCCGGTTTATAATCAGACGGACGCTGTGAAGGCCATGGTGGCG
39724435|F|0-43:C>G-43:C>G	TGCAGTTTGAAATGTGATTGAAGTTTCCTTTTGATGGATTTGACGTTGAAACCCTAAAACGGGTTCGAA
39715898|F|0-60:T>A-60:T>A	TGCAGCCATGGAAAAAATTTCTGGTTGGAGGAAAGATGAAGTTATAGGAAAAATGCTAGCTGGGGAGAT
39721937|F|0-55:C>G-55:C>G	TGCAGATAACCCAAAAGTCATTCAATAAATCTTAGTAAAAAATTCTCCCACTATTCGATTCAATGACTT
39714971|F|0-46:G>T-46:G>T	TGCAGATGATCTTCTTGCATCTGTTCTAAAGGTTTGATTCATTCATGCATTTATGTTTCTTTTCTGCAC
39722261|F|0-65:G>A-65:G>A	TGCAGCAACAACAATTTTGGCTGCTGTTTCTGCTGTTTATGTTAGCAAAGGGCGCTGAGGCTGAGGCTG
46757706|F|0-43:C>G-43:C>G	TGCAGTTTATTCTCATTTCTGTGATTGTTGATCGAGCCGATTCCAAGGGCGTCCCCAATTCGACGATTC
39722655|F|0-18:T>G-18:T>G	TGCAGCCCGAATCACTCATATCTTTGGAGAGACCATCAATATTTCATTCCAAGTTATTATAGGCTTACA
39725234|F|0-17:A>T-17:A>T	TGCAGTACATAATTACAATTACACGGTTAC
46754334|F|0-35:A>G-35:A>G	TGCAGAGAGGTACTCTGTTTTACAACCAACCAGATAATAATGTAAAAGCTAGTAGTTCTAAATCAAAAG
39724797|F|0-32:G>C-32:G>C	TGCAGATATCAACAACATCATAAGAGGTACACGATAGGAAGCACTCTAAAAATTTAC
46757530|F|0-36:C>T-36:C>T	TGCAGTTCCAAACAAAGAATCCATCTATGAAGTGAACGGCAGTCCAATTACAAAGCACAAGGGATCTTT
39720505|F|0-11:A>C-11:A>C	TGCAGGTGAAGAACAGATTGCGGCATTAC
39725369|F|0-11:G>A-11:G>A	TGCAGTTCCCCGAGTTGGATGTTAC
46764972|F|0-51:C>T-51:C>T	TGCAGTCAAAGCTTAGACCACTTGATCCAGTGCGTGAGTAAGATGGTATGACTCGAAAGATATTGATGT
39713849|F|0-10:C>G-10:C>G	TGCAGAAGCTCCAATGACGTTTGCTGAAAAGCATGGCTGGCAATACGAGGTTTCACTGAAATTTTGTTA
39716201|F|0-47:C>T-47:C>T	TGCAGCGTCCAACTGTCTTCCTGTCACCACATTCTTCTTTTCTCTTACTCTAAGGTTCTACTCTACATT
39725292|F|0-12:C>T-12:C>T	TGCAGTCTTCAACCAACTTGCTTTGTGATGATTGTTAC
39715875|F|0-38:T>A-38:T>A	TGCAGCCAGGGACATACCTATACCATGCTCATTACGGTTTGCAAAGAGAAGATGGTCTCTACGGATCCA
46761021|F|0-31:G>T-31:G>T	TGCAGTAATGAAAAGGTTATTTACTTTTTTTGTTTTTAC
46776428|F|0-52:C>A-52:C>A	TGCAGAAAGTTATAGTGAAAGTTGTACTTTTTCCCATAAGACTTCACCTTCACCATAATATGCACAAGC
39723852|F|0-66:A>C-66:A>C	TGCAGTATATATATTTAGTTCGTGTTTCTTGCATTCCTTCTAGTATTTTACCTCCCGAGACTTTATACC
39724364|F|0-55:C>T-55:C>T	TGCAGTTGGTTGTGATTCTTGTGGGGTAATTTCCTTATATTTCCATCATTCCTCCCGTACTGCTTTCGA
39725315|F|0-14:A>G-14:A>G	TGCAGTGCCGATGAAAGAGTCAATTAC
39726083|F|0-61:T>G-61:T>G	TGCAGCATCATCTTCTCCTTCTCCACCTCCATGTTTTGCAACCAGAACCTGTTGAAAACAATAAAAAGT

<table>
<tr><td>39718246|F|0-18:A>T-18:A>T</td><td>TGCAGTGAACTTATGGGTAGCCGAAAAGCCTTACTATAGTTATAACAACAACGCCTGCGTCGGGGGCGA</td></tr>
<tr><td>39717299|F|0-55:G>T-55:G>T</td><td>TGCAGGGCATCATATTGATGGCCGCTACAAACTTGCCAGATATTCTTGATCCAGCGTTGACAAGGCCCG</td></tr>
<tr><td>39720148|F|0-11:T>C-11:T>C</td><td>TGCAGCTCAAATCATAAATAATACCATAATTTGTTTTTAC</td></tr>
<tr><td>39718512|F|0-35:G>A-35:G>A</td><td>TGCAGTGTTGAAGAGGATATGGTCGATGGCTTCCCGTACGAAGTACCTGAGGAATACAAAAACATGCCC</td></tr>
<tr><td>39727917|F|0-55:C>T-55:C>T</td><td>TGCAGTTTGCCGTTGAGCATCGAGGGTCGTATAGTGATTCGCTTGGATCTGCTGTCTGTCCCTTTTATT</td></tr>
<tr><td>39721378|F|0-9:A>G-9:A>G</td><td>TGCAGAAGCAGGCACAAATGACTGCGACTGTAATGAAAGATTTCAAGCCGGCCATGTTCAAAGTTTCTT</td></tr>
<tr><td>46756353|F|0-36:C>G-36:C>G</td><td>TGCAGGCAACCAGAACATGTTTGCACATGACACTATCTCAGGGTAAGCCAGAGCCCATGATCCAGTATG</td></tr>
<tr><td>46763304|F|0-44:G>T-44:G>T</td><td>TGCAGATTGCTGAAGAACGATATTTGAGGTCATGTGGTGTCTCTGCTTTGACCTCACAGCTACCTAAAT</td></tr>
<tr><td>39726053|F|0-17:C>T-17:C>T</td><td>TGCAGCAGCCGGGCATTCTCAATTGATCTTTCTCCACTAACTTTTGTTTTCCCAAATCTCCATGGTTCT</td></tr>
<tr><td>39718757|F|0-53:A>T-53:A>T</td><td>TGCAGTTGCGCTGCAATCAACAGAGATGGAAACCTTTCCTTTGTCAGGCTGCAATGCATTAGGCCTTCC</td></tr>
<tr><td>39715414|F|0-27:G>A-27:G>A</td><td>TGCAGCACAAGTTGCCACAACCACAGCGAAGGTCAGGACAGAAATTGCAGCAACAGAAGCCATGAATTA</td></tr>
<tr><td>39724641|F|0-20:A>G-20:A>G</td><td>TGCAGAAGAGAAACAAAATCAGATGTCGAAATTAC</td></tr>
<tr><td>46765261|F|0-68:G>T-68:G>T</td><td>TGCAGTGTGTGGCTTCTCACCCGGAAACAAGGCTGCCATTTATGAAAGGTAAAATCTCGGTAAAGTTCG</td></tr>
<tr><td>39720768|F|0-14:T>G-14:T>G</td><td>TGCAGTGTACAACCTGACGACTGAAATTAC</td></tr>
<tr><td>39725714|F|0-7:G>A-7:G>A</td><td>TGCAGAGGCACACTTCTTGAAGTACTCCACAATGAGCCGCACAATAGAAGCAGCATGAGGTAACAATTG</td></tr>
<tr><td>46756865|F|0-37:T>C-37:T>C</td><td>TGCAGGTTGCCCTGCTTCTCCAGCCCTCTACAGCCAATTCCAAATGGTATCATAGTACGGGTGATGTGC</td></tr>
<tr><td>39719495|F|0-55:A>G-55:A>G</td><td>TGCAGACAAGACCAATGAAGCTATGCAGTGTAGTTTTCTGGAAATAGTAGGATACATTAC</td></tr>
<tr><td>39713726|F|0-47:G>A-47:G>A</td><td>TGCAGAACTACATTGAACACAGGTATGCATACCAGATATTATTGGTCGTAAATACATACACTCGGCAGA</td></tr>
<tr><td>39715444|F|0-45:T>C-45:T>C</td><td>TGCAGCACCCTGTAATATTCCTGAAATTCACAGGGAGGTAAAGCCTCAAGACATTGAAAACGGGCACTT</td></tr>
<tr><td>46776530|F|0-61:A>T-61:A>T</td><td>TGCAGAGAAAAGACTTTCAAGTTCTGACATGTCAATCTCTGGTGCCCTACAACAAAAAAAAATTACAGA</td></tr>
<tr><td>39726465|F|0-54:T>C-54:T>C</td><td>TGCAGGGATTTCGAAACTACCGAGAATGTGTCTGTTCATAGTCTTGGCTTGGATTGTTCCAAATGAATT</td></tr>
<tr><td>39725332|F|0-39:T>G-39:T>G</td><td>TGCAGTGGAGATGTATGTACAGCTAACTGTTCATATTTTTCCCATTTTAGTTTATTAC</td></tr>
<tr><td>39725074|F|0-18:A>G-18:A>G</td><td>TGCAGGACTTCCCCTCCTAATGAAGTTTCCTTCTTTAC</td></tr>
<tr><td>39726006|F|0-57:C>T-57:C>T</td><td>TGCAGCAATTTGCCAGATGGAGACGCCAATTGATTCTAAAACAGTCTGTGAAAATAACAACCATAAATC</td></tr>
<tr><td>46776531|F|0-62:T>A-62:T>A</td><td>TGCAGAGAAAAGACTTTCAAGTTCTGACATGTCAATCTCTGGTGCCCTACAACAAAAAAAATTAC</td></tr>
<tr><td>39715488|F|0-25:G>T-25:G>T</td><td>TGCAGCAGAATACACATCTTTTTCTGTGTTTGAATAATAATTCCCCGATAGCCTTCCACTGCCCTACAA</td></tr>
<tr><td>39715947|F|0-30:C>T-30:C>T</td><td>TGCAGCCCGTTTCTCTCCTGATGACAAATACTCGAGGCAGAGAGTTCTTCTCAAGAAGCGATTTGGCTT</td></tr>
<tr><td>39722231|F|0-66:A>G-66:A>G</td><td>TGCAGCAAAATACCAAGAACAAGAAGATAGTATTTCAACAATATTCCATTGGCAACAATAAAAAAAAGG</td></tr>
<tr><td>39717445|F|0-40:T>G-40:T>G</td><td>TGCAGGTACAGCCTATGTTTATCATGGTACAGTCGGTGGCTCTCGTTCACTTCATCTGAGGTTATTTTT</td></tr>
</table>

39727739|F|0-46:G>A-46:G>A	TGCAGGGTGAAGTTGAAATTGTCTGTCAGTGTGTTAGGCTTAGTTTGGGAGCTAAGGCTTGAGGAAATC
39723282|F|0-16:C>T-16:C>T	TGCAGGCAAATGGTTTCTGCTGTTTACGTTTCAATGGGTTCTACCAATTTGGGCATTGTCACTCCTTGT
39722323|F|0-7:A>G-7:A>G	TGCAGCAATTGGAACATAGAGTTTTAGTCAAACCTTAGAGAATGAAGAAATTACTGAAATCTAATATGT
39722716|F|0-60:C>G-60:C>G	TGCAGCCTCAATGTACACTCTTCCTAATCAACCAAATTGTGTTGTCAAGTTCAACTTCGGCCCCGATTT
39726146|F|0-47:C>T-47:C>T	TGCAGCCGATGATCGTTCAAGTGCAAACCAGACCGATAAAACCACCCCCGATGATTCCAATTCAGATGG
46765044|F|0-54:T>C-54:T>C	TGCAGTCGGGCCGGCACGTGCCCACCACATTCTTCTGTTTGAAATTGTTGGGTTTTTGGAAATTGGTTT
39724371|F|0-22:T>G-22:T>G	TGCAGTTGTATATACTTACTATTACTTTGTCATAGGTTGGTGATCCAGAGCAACTTCCAGCCACTGTAA
39718765|F|0-12:C>T-12:C>T	TGCAGTTGCTGTCACCATTGGTCCCGGTTTGAGCCTCTGTCTCCGCGGTAATTTTGCAGGATTCCTCCC
39728758|F|0-67:C>T-67:C>T	TGCAGGCTGGGTATGTAGGGAGATGAAGAAGTGACTCGAATTATCTTGGTCTCTTTCTAGTTCACTTCA
39714492|F|0-7:T>C-7:T>C	TGCAGAGTAGATGTGTCTGGTATTTTGGCCACAGCATCAATGATTCCTCGGACAATTTTTGCTGTTTTT
39717480|F|0-28:T>A-28:T>A	TGCAGGTATTCTTGTCTTGTGCCGATCTTCGGAATTATTTTCGTTCAATTTTGATAATGTACCAAAATT
46755806|F|0-15:T>G-15:T>G	TGCAGCTCAGAGTGCTGATGTTATCATGAGAATGATGGCTAGGAACTCCATGTCAGAAAAACTTGCTGA
39723815|F|0-55:A>C-55:A>C	TGCAGTAGCAGTCCCATCTGGTATAGCTTGAATCCGATCCGGCAATGCAAGCACCAAATCATTTCGAGT
39725582|F|0-57:G>A-57:G>A	TGCAGAAGATAAGAGCCGGTGAGATCAAGGTGGTGACCGGAATAAAAAAATTCCGGCGAGGTGCAGTGG
39726073|F|0-18:C>A-18:C>A	TGCAGCATAAGAACATCCCAGAGTAAAGCTAGTTGCTAGTTCATAATTCTTCATGTGTATGTCTAACTT
39715938|F|0-66:A>G-66:A>G	TGCAGCCCCGACATTCGTGACTTGACGAGTGTATCGAACAACCCTCTTGCTCCCAAACACAACTGAAAA
39721306|F|0-15:A>G-15:A>G	TGCAGAACTGCTCTAAAGACTTGGTGTCATTGGTATGCTTAGGAATGAGATTGACATTGGCCTTCACAG
46757512|F|0-36:T>C-36:T>C	TGCAGTTCACCCAAAAACCCCATACCAAAACCAACTTTATACATTTTCAAATAAATCATCTGAATCTTT
39714998|F|0-55:A>G-55:A>G	TGCAGATGCTATTGAATAACACGACACTACAGGCATTATATTTATTCGTTGTTTTAGTCGGCTACAAAA
39725018|F|0-34:T>C-34:T>C	TGCAGCTGCCTCTTGCTTGCCTTCCAAGATCTCCTTGCTTCCCAATCAATTCTTAC
39714746|F|0-18:A>G-18:A>G	TGCAGATAAGGATAATAGACCTTGCAAAACTAACAGGCAACGACAATCCAGACGAGAATGTAAGTGGCA
39724041|F|0-63:A>C-63:A>C	TGCAGTGACAATGAACAATTTTCAGATGTACCGCCATATCAATGCACCAGGATGGACTCTAGGATGGTC
39723592|F|0-60:C>G-60:C>G	TGCAGGTCATTGAGCTCTAGTACAAATATGGTAGATTTGACTGACAAACTTCCATATCACCTGTTGTAA
39726977|F|0-6:G>C-6:G>C	TGCAGAGAAGTAGAACAACATTTGTTAC
39715781|F|0-12:C>T-12:C>T	TGCAGCATTGTTCTTGTTCTATGTGTATCTTAGTGCAGAAATTTTCCTCTGGCAAGCTATTGGGTTTGT
39719580|F|0-14:C>T-14:C>T	TGCAGAGACGTATCCCCCTTCGAGTGGTAAGTTTAC
39717240|F|0-52:C>G-52:C>G	TGCAGGCTTTTCCTTTCAATACTTTGTGGCATTATGATCTAGAAGCGCCAGTCTTTGGGTCCCTTGCAA
39715450|F|0-7:C>G-7:C>G	TGCAGCACGCACATACTTTGGTGTTTGGAGTGACCCATGTAATAAAAGCCGACATATTCAGCATGAAGG
46771788|F|0-18:G>A-18:G>A	TGCAGAGGTAATGATACAGATGCAATTCAAAAACTACTGAAATTGGAACACTCGTCTACATTCAAAGTA

39728384|F|0-11:G>A-11:G>A	TGCAGTCTCTTGTTCTGTTCTGTCAATAGCCCGAACCATCTCTTTAGATACTCGCATTCCATCTCTGTT
39721133|F|0-16:G>A-16:G>A	TGCAGAAAACGTGCCCGCATTTTGTCGTTGTTTCTTCGACCAATGGTTCTATGCAGATCGCACACCGCA
39714211|F|0-28:G>T-28:G>T	TGCAGACCTCCATTTACAGAGGTTTCGCGACGGCATTCATGAAGGAAGCGGCTGCGCTGAACTTCACGC
46768538|F|0-46:G>A-46:G>A	TGCAGAGATCACGGTGCCATGTAGGAACATTTTTGTATGTCAATCTGGCAGTAACATCAAACATGATGA
46764609|F|0-59:C>T-59:C>T	TGCAGGGTCGAGGGTTGTGTATTGTAATGTGCAAAGGGCTGGGAGTAGAGTGAATTCATCATGCCCTGC
39717386|F|0-35:A>T-35:A>T	TGCAGGGTCCATATGTTAGTAATATTTTATTGGTGACTGAACAGGGTGACTTGGAATGAATCGTTTGCC
39718514|F|0-20:T>A-20:T>A	TGCAGTGTTGATAACTAGAATTTTCTGCTCTGGTTGTTGGAGCTTGAAAGCCACATTGCCATGGTGGTT
39722143|F|0-65:G>A-65:G>A	TGCAGATTATAAAGCAAAACAATAGATTTTGGGAGCTCTCTTATTCGAGAGTGTGAAATGTCAAGGTAC
46754505|F|0-54:A>T-54:A>T	TGCAGAGTGAGAAATTACTAATACTTCATATAATGGTTACAATTTATCACAGGGAATAAGGAAGCTTTT
39723998|F|0-60:T>C-60:T>C	TGCAGTCTATATTCCTCCATGCTCTTTCTGCAACAACCCCTTTTCTTCTAGCCCCATTACTCCAACAGA
39717193|F|0-54:G>A-54:G>A	TGCAGGCTCTAATCTGTATTTCTGGATAGCAAGCATTCCGATTACTGTGAGTGCGCTCTCTCCTTCTCT
39718314|F|0-5:T>C-5:T>C	TGCAGTGATGGATAATAAGCCTCAAAGTTGCTTGCCAACGAAAATTTTCAAGTGCTCCTTTCAAGATAC
39714561|F|0-19:C>T-19:C>T	TGCAGAGGAGAGAGAAACGCCGCTGGATTTTCAGAAAGCCAACCAATCACCATCAACAACCACCGCCCA
39715873|F|0-12:C>T-12:C>T	TGCAGCCAGCTTCGGCGCCTCTGCCACGGCAAAGATTAGTGTTGATGCTTCTCTTGCTGGACCCATCAT
39715955|F|0-56:T>A-56:T>A	TGCAGCCCTCTAGCAATTACGCTAGAAAGCCAAGGTGAGAGTTTACTTGGTAGACGTATAACTGCTTGC
39726791|F|0-29:T>C-29:T>C	TGCAGTTCCAGTCAATGCAAATCCTGAATTTGTAGCAGCTACTAGATTCGCCCAATCTTCTTCACATGG
39713825|F|0-41:A>C-41:A>C	TGCAGAAGCAACTCGCGAGTACCGAAAGGGGGCTTTTCACCAGTGATCCAATCTTTCTCAAGAACACCA
39723377|F|0-62:G>A-62:G>A	TGCAGGCGGTGGGCCAGCTCCGCCGCCTGAGCTCTGAGAATGGAATTCTCAGACTCGACGGCGGCGTAG
39723501|F|0-24:C>T-24:C>T	TGCAGGGGTCCCCTTCTCTTGCATCGCGGAGAGCAGTCGTTTGGCTACGAGACCCTTGCTGACGCCCTG
46756200|F|0-48:G>T-48:G>T	TGCAGGACGGATCCTTAGATCTCTATGCCTGTGATGATGAGGGAAAGTGTATGCGGCTTTTCATTTCTA
39725015|F|0-20:A>G-20:A>G	TGCAGCTGCAAGAATCTGAGACGATTTCCTCGAGCCCCGCCCACCTCTTAC
46769895|F|0-36:T>C-36:T>C	TGCAGTGTGCTCATTTTTTGTGCAGAGATATATGCATAGTGTTCGAGTACTCATAAACTTTTCCTTCCC
39725064|F|0-9:C>T-9:C>T	TGCAGGAATCGGAGCCTGCCTACTATGGAGATCCATTAC
39714470|F|0-62:T>C-62:T>C	TGCAGAGATTGAGTGCCCACTCTATAATTCAAAATTGACCCATCGTTTTTACTCTCTCTCTCTCTGTAC
39714991|F|0-9:C>G-9:C>G	TGCAGATGCCAACTTGAATCTTGGGCTTGGCAGTGGAATTCTGATTGCTGTTCCAATTCCAAAAGAGCA
39719481|F|0-7:T>C-7:T>C	TGCAGAATTCTGAGGCGCTCATTGATTCTCTCTCTTCTCTTCTGCATTGGTTAC
39722523|F|0-48:C>T-48:C>T	TGCAGCATCATTAGGACCTCTTCTAGCTTGGGGATTAGTGGCTTGAGCCATCTGTTGGTTCCTATTTAC
39716426|F|0-7:T>G-7:T>G	TGCAGCTTCATTTTAGTTTCTTCAGATAGAAACTTTTGTAACTACTCTATTCTCCAAATTAC
39718126|F|0-34:G>A-34:G>A	TGCAGTCGAGCTTGATGCTCCCGAAGACCCCGAAGGGGCCATCATCATCTTACCATCTTCCATCTCCGA

<table>
<tr><td>39717295|F|0-5:T>G-5:T>G</td><td>TGCAGTGCACAATCACCAGAGAATCCTCGCCAAGAAACTTCAAATTGATAGGCAGTAGTTGGGGCTATA</td></tr>
<tr><td>39725304|F|0-9:T>C-9:T>C</td><td>TGCAGTGAGTTTATTGTATATTTTTCTAAAAAAGATTAGTCTCTATTAC</td></tr>
<tr><td>39720684|F|0-7:G>A-7:G>A</td><td>TGCAGTCGCTTGACGGCCTTACCCCGTCTCTCTTATATTAC</td></tr>
<tr><td>39729483|F|0-18:C>G-18:C>G</td><td>TGCAGGCCCTTCAACGTCCACTCTCATATGATGGTACCCCAATCTTTACTATCGAAGTACGTTAC</td></tr>
<tr><td>39719452|F|0-42:T>G-42:T>G</td><td>TGCAGAATCAACCAAAAGAGAAGTCCATTTTCAGCACAAAACTTGGACGCTTAC</td></tr>
<tr><td>39719352|F|0-5:T>A-5:T>A</td><td>TGCAGTACACAAAATTCAAAGAAACCACCCTATTAC</td></tr>
<tr><td>39720872|F|0-46:A>T-46:A>T</td><td>TGCAGTTGTTCGACAGGTATGGTACTTTGAGCATAAGAAATGAAATATGTCTCTTAC</td></tr>
<tr><td>39729487|F|0-57:C>T-57:C>T</td><td>TGCAGTTCTCGACAACTACAGTGAAATTTTGAAATCTTGTAATGCTCTGGACTACCACGACTTAC</td></tr>
<tr><td>39713960|F|0-6:A>C-6:A>C</td><td>TGCAGAATAAAGCTGGCAAGCGAAAGATGGTAACTCATTTTCCTTGACCTCTCTAGCAAGAACCACAGA</td></tr>
<tr><td>39715118|F|0-51:G>A-51:G>A</td><td>TGCAGATTCTGTATATAGAGCATGCTTTGAAAAGGTTATCATTCAAAATGAGAAGTACTACAAGAGGTA</td></tr>
<tr><td>39728383|F|0-17:C>T-17:C>T</td><td>TGCAGTTTCTAAATGTTCCATATTTATTTTTGAAGCCTCAAGGTTTTCCTGAAAATAAAAGTAAGATAT</td></tr>
<tr><td>39722854|F|0-61:T>A-61:T>A</td><td>TGCAGCTACCGTCTAAACAAAAATGCGCATTCAGAACCAAAATCTCAGTATGCCTCTGTCCTCCTCTCC</td></tr>
<tr><td>39728448|F|0-26:C>T-26:C>T</td><td>TGCAGAGAACCCGTTATCGGCAACAACCTCCCTCTAAGAATAAAAGCTTAC</td></tr>
<tr><td>39726593|F|0-30:C>T-30:C>T</td><td>TGCAGTACCTGAAGGGGTGGGCAAAGAAGCCTATGCCTCAGTTCAACTAAGTTACAAGTATGTAGAAGG</td></tr>
<tr><td>39721699|F|0-29:T>A-29:T>A</td><td>TGCAGAGAAATCTCTCTGTTCTGACACTATAGCTTCTTCAACAGTACTGGGAAAGCATCAAAGAGACAA</td></tr>
<tr><td>39713392|F|0-20:T>A-20:T>A</td><td>TGCAGAAAAAGAACATTTATTGTTGTGATGCATGATGTGGAAATTGTCTCTTGGCAATGAATTTTAGTG</td></tr>
<tr><td>39718885|F|0-36:T>C-36:T>C</td><td>TGCAGTTTCTCAGGTGCGTTCGATGGTTTCAGATGGTGCTGATATGATTGACATTGGTGCTCAGTCGAC</td></tr>
<tr><td>39717688|F|0-55:C>T-55:C>T</td><td>TGCAGGTTTTTATCCCACAGGCTTTGTCAATGAGGGTATGTAAAATTTTTGAATGCGTTAGAAGGGGCA</td></tr>
<tr><td>39718393|F|0-28:G>C-28:G>C</td><td>TGCAGTGGAGCCAAAATCTAGCGAATCTGACAGATCGACTGGTCCGATTCCAACGCAACCAAAAGGGGT</td></tr>
<tr><td>39716341|F|0-8:C>T-8:C>T</td><td>TGCAGCTCCTCCGACTATTTTTCTGCCATCCGCTTGATTCTGCCTAGCCTCGATCGAGAGCGCGGCACC</td></tr>
<tr><td>39717581|F|0-28:T>C-28:T>C</td><td>TGCAGGTGTGGCTTTATGATGTTGGATTTGGAAGTTTTGTTTATCAAGTTGAAAAGGTTTGTGATAATT</td></tr>
<tr><td>39728228|F|0-35:G>A-35:G>A</td><td>TGCAGATTCACTCCTACATCTTCGTCGTACTCAAGGCCAAGTTCTTCCTGCAAGAATACAAAGAATTTA</td></tr>
<tr><td>39717429|F|0-59:T>A-59:T>A</td><td>TGCAGGTAAATTTTGTAAGTGTAACCTTGTTTCTAGGGTAAGTAACTTCTACAACTAAATTGGAGCATT</td></tr>
<tr><td>39717276|F|0-35:G>C-35:G>C</td><td>TGCAGGGAGCGGGCCTTGTTCTTCCAGATATCAGTGCGGTCGATGAAATTCACAGCCATAGCATTTGAC</td></tr>
<tr><td>39726234|F|0-49:A>G-49:A>G</td><td>TGCAGCTACCCGAGAATATGCACCACTGGTTGAAGAGTTGTGGAATGATACTGCTATTCAGGCTACCTA</td></tr>
<tr><td>39713768|F|0-45:C>G-45:C>G</td><td>TGCAGAAGAAGATTGAGAAACATAGTAAATATTGATGATGCTCATCAGGCTTCTCAATAAGTAAGAAGA</td></tr>
<tr><td>39727920|F|0-52:A>T-52:A>T</td><td>TGCAGTTTGTCAATCTTGATTTGCTTGAAGTCAAGGTACTACAAATTACAAAATTTTCCGTTGGTTGGA</td></tr>
<tr><td>39721958|F|0-13:T>G-13:T>G</td><td>TGCAGATACTGTCTGGAGAATATATGCAGTTCACCTGATATGCTAATCTCTGTCTTGGCTGGTTTGTCA</td></tr>
<tr><td>46757615|F|0-22:T>A-22:T>A</td><td>TGCAGTTGATAAGCTCGTTCGCTTGGGCTTTGATAGAACAAGAGGTATCTATGTATGATCTAGATGTTA</td></tr>
</table>

<table>
<tr><td>39725522|F|0-48:C>T-48:C>T</td><td>TGCAGAAACTGAACCAACTTCCAATTTGTATCTTACTGACTGGTAATCCGTAATGCAGTTGTACTGGAG</td></tr>
<tr><td>46755056|F|0-30:G>T-30:G>T</td><td>TGCAGCAAGGGACAATTTGGAGTTCTTGGAGAAATGGTTTGTCAGATTCCCACATTACAGAAACAGAAG</td></tr>
<tr><td>46755574|F|0-24:C>T-24:C>T</td><td>TGCAGCCGTGGTAACAGATTGAGACGGTCCTCCAACATTTCTCAGCTCCCCAGCAGATATCCTAAGCCC</td></tr>
<tr><td>39721271|F|0-39:T>A-39:T>A</td><td>TGCAGAACCAAAAAAATGTATCCAGAGAATCCAAAACACTATTAGCAGCCTAACAAAATTAGCATGCCT</td></tr>
<tr><td>46754390|F|0-39:T>C-39:T>C</td><td>TGCAGAGCCATGGATTCATAACACCACTATCTGGGAAGATGAAGAAACTGACCTCAAATGTACTGTGGA</td></tr>
<tr><td>46762309|F|0-23:G>A-23:G>A</td><td>TGCAGAAAAAGCTGGTGCTAGAGGATTTCAATATAGTGGATGCAGCAGGTGGCGTTGGTAAAGCAGTCA</td></tr>
<tr><td>46765077|F|0-19:A>G-19:A>G</td><td>TGCAGTCTGTAATTACCTCATAAATCATCTCCTTCATACCTTTTCCTCATGAGGATGGTTGTTGTTTAG</td></tr>
<tr><td>39713273|F|0-47:T>A-47:T>A</td><td>TGCAGATGTTAGGAAACCACCGTGGTTATTGTCTTGAATACATGCATTAGAAAAATAGTGGAAAGCAAA</td></tr>
<tr><td>39723161|F|0-13:T>G-13:T>G</td><td>TGCAGGAATGGATTCAATCTGCTCCTGATGTAAAATTGCATCTACCAGCTCCGAACATTTTCATCCCCA</td></tr>
<tr><td>39725065|F|0-12:A>G-12:A>G</td><td>TGCAGGAATGTTAGTTTAGTCATCTTGTACACAATTAC</td></tr>
<tr><td>39729043|F|0-60:A>G-60:A>G</td><td>TGCAGTTTCTGTTTGTCCAAACAAATAAAAATTGCACCATCAAACATTTTCAAATGCTTTAGAATCAGT</td></tr>
<tr><td>39729517|F|0-19:T>C-19:T>C</td><td>TGCAGATCAGAACATTGAATGCAATTAC</td></tr>
<tr><td>39718751|F|0-33:A>C-33:A>C</td><td>TGCAGTTGCATGGTCATATCTTTCAGCTAAGGCACGGTAAGCTCGGTAGAATTCCTCAACTAGTTTCAT</td></tr>
<tr><td>39727204|F|0-42:A>T-42:A>T</td><td>TGCAGTAGTAAACAAATATTGTGAATGTTTTGCATTTTGCTCAATCAGTTCTGAATATTAC</td></tr>
<tr><td>46755817|F|0-20:C>T-20:C>T</td><td>TGCAGCTCCAAGAGAAGTGGCTGGTAAAACATGAGGGTCGGCAAAAAGCTCCTCCCCTTCGAGTTGTGT</td></tr>
<tr><td>39721343|F|0-32:C>G-32:C>G</td><td>TGCAGAAGACAGTGACCATGCCGAGAGTTTTACAACTAATGTTCGTAGACTTGGTCAAGCAAATTACCA</td></tr>
<tr><td>39727451|F|0-20:T>C-20:T>C</td><td>TGCAGAGGATGCTGATAGTGTTGAATCTGAAGGAGGTACAAGAAAAAAAGAAGGGGCCTTCTATGTGTG</td></tr>
<tr><td>39725013|F|0-12:C>T-12:C>T</td><td>TGCAGCTGATTTCCATACATATTCAATAATGTGGTCTCATTGGCAAATCATGTAAGTTAC</td></tr>
<tr><td>39721920|F|0-31:T>C-31:T>C</td><td>TGCAGAGTGCCTTCATCAACCAAGATAGACATATGTATCATGAGGAAACTGATAAGCCAGCTCATGCCC</td></tr>
<tr><td>39717868|F|0-55:G>T-55:G>T</td><td>TGCAGTAGACACCACAAGAGAACTATCCCATTCATCTCATTTTGGACAATCATGTGCATCATCAAAATC</td></tr>
<tr><td>39721118|F|0-46:C>T-46:C>T</td><td>TGCAGAAAACAAAAGAGCATGTGTTGGCTTCCAAAATTATAAACAGCGCCCACTTACTTTTGCTCCTCT</td></tr>
<tr><td>46760835|F|0-23:A>C-23:A>C</td><td>TGCAGCAAGTATAAACACTATTCATGTCATTACCTTCCTTTTCGCTCTTCTATTAC</td></tr>
<tr><td>39720047|F|0-8:C>T-8:C>T</td><td>TGCAGCCCCTTCTGCTTTCTGGATTTACTTTTTTGACTTTTAC</td></tr>
<tr><td>46774452|F|0-30:A>G-30:A>G</td><td>TGCAGAGGATGCTGATAGTGCTGAATCTGAAGGAGCTACAAGAAAAAAAGAAGGGGCCTTCTATGTGTG</td></tr>
<tr><td>39724671|F|0-5:A>G-5:A>G</td><td>TGCAGAATGCTGGCTTTTTTATTAGAGGAAAGAGAGATAGATTGAGATTAC</td></tr>
<tr><td>46755216|F|0-57:G>T-57:G>T</td><td>TGCAGCAGCCCTGATGATCAAACCGACATATCATTGCCTTGAAATAAAATGCAATGCGTTCTATCGGGA</td></tr>
<tr><td>39720617|F|0-23:C>A-23:C>A</td><td>TGCAGTATACAGATGTGTACAGCCACAAAAGAAGCGATACGTTAC</td></tr>
<tr><td>39721657|F|0-53:C>G-53:C>G</td><td>TGCAGACTCAGTTGCTTGGCACCGGCTAGAGCTGCAATTCCTGAATCTGTGATCGAGCATTTTGAGACA</td></tr>
<tr><td>39717049|F|0-7:A>T-7:A>T</td><td>TGCAGGCAAGCCCTTCAGATCCGGAAAATTTTCCCTTTGTTGTTTTAGGAAATAAAGTTGATGTTGATG</td></tr>
</table>

<table>
<tr><td>39725456|F|0-14:C>T-14:C>T</td><td>TGCAGTGGACAGAACACAGAGCATGGAATGTCAAACATACACACTCTATTTGTTCTGTCCTTTACTGCA</td></tr>
<tr><td>39718486|F|0-49:T>C-49:T>C</td><td>TGCAGTGTATTGATCCACATACGCGTGAGTATGTTGAAAGACTCAGAGATGAGCCAATGTTTATGGTTC</td></tr>
<tr><td>39717691|F|0-13:G>A-13:G>A</td><td>TGCAGGTTTTTGAGCAAAAATCAAAGTGGAGAACTTGTAGACTTGCACTGTATTTTGTATTGTCCTTCT</td></tr>
<tr><td>39725956|F|0-63:C>T-63:C>T</td><td>TGCAGCAAAAATCAGCAATATACAAATCCGTCGTGGGTGATCCTAAATTTCTTCAAATTTTAGCGGGCA</td></tr>
<tr><td>46756457|F|0-12:C>G-12:C>G</td><td>TGCAGGCGGCGGCGCCCAAGGCGAACTTGGTGGTGGCGCAGGATGGGTCGGGGAATTTTAGGACGGTTC</td></tr>
<tr><td>39715644|F|0-42:T>A-42:T>A</td><td>TGCAGCATAAACATGATAAACATGTATAATTACAACGTATATTGCAACACATCAATCAAAATCCAAATT</td></tr>
<tr><td>39719598|F|0-55:A>T-55:A>T</td><td>TGCAGAGATTGCAGATAGTAGAAAGGCATATAAGTAGATGCGGGGATGCCCGTCGAGTTAC</td></tr>
<tr><td>39721042|F|0-56:C>G-56:C>G</td><td>TGCAGAAGTCTCCGGCGGCTGAATCTGTCGCACAACATCCTCGACGACAAGTTGAACTTGTCGGGGTTG</td></tr>
<tr><td>39715392|F|0-25:A>G-25:A>G</td><td>TGCAGCAATTCACAAGTCAATTTGAAGTTTCAAACTTCTTTCCTTTGTGGAAACTGCAACTCCTCTAGA</td></tr>
<tr><td>39714906|F|0-11:G>A-11:G>A</td><td>TGCAGATCGGTGGCCGGAACGGTGACGAGCGAGGGGCAGGGGCCTTGGGTGATGAAGACGTAGCTGGTG</td></tr>
<tr><td>39725200|F|0-43:T>A-43:T>A</td><td>TGCAGGTTGATCTATTTACAAGATGGATAAAATATCATTTTACTCTTAC</td></tr>
<tr><td>39718243|F|0-54:G>A-54:G>A</td><td>TGCAGTGAACATCGTTGCCACAGTGAATACATCAGTCGGGTATTGTTCACCGAGGAAGAAATGGAACGA</td></tr>
<tr><td>39724272|F|0-40:T>C-40:T>C</td><td>TGCAGTTCGAAATTTGGTATTCTTCGATTATATTTTCATATGCCTGGTGTTTTCACTGATAGGAGCTTC</td></tr>
<tr><td>39716135|F|0-57:G>T-57:G>T</td><td>TGCAGCGCAAGAAGGAACAGTGTACTAAGCCCTGTCAAAAGAACGCCACACCAAACAGGAATGCTGAAG</td></tr>
<tr><td>39717016|F|0-68:A>T-68:A>T</td><td>TGCAGGATTCCAATCATAAAGCTTGAACGATATTCGGCCTGTTGGACACTATAACAAAGAGATTTGGAA</td></tr>
<tr><td>39728087|F|0-33:A>T-33:A>T</td><td>TGCAGTAGAATGCTTAGGAAAATAAGTATCTATACGCATTTTAC</td></tr>
<tr><td>39723627|F|0-27:T>C-27:T>C</td><td>TGCAGGTGCATAACACTTTTATTATGTTTACAAAGTGAATGAATGAAAATAATTTACTCATTACACACA</td></tr>
<tr><td>39714401|F|0-5:A>G-5:A>G</td><td>TGCAGAGAGAAAAAAATAGCGAAGGCTGAGAGCATAAGAATGGCTAAATCTCGCTTCCTCATATTGTTC</td></tr>
<tr><td>39723778|F|0-35:C>T-35:C>T</td><td>TGCAGTACAACCTTCTTCTTCTTCTTCTTCTTAGTCGGATCAAAGGGGGCAATCTGCACAGAATAAACC</td></tr>
<tr><td>39714899|F|0-13:T>G-13:T>G</td><td>TGCAGATCGACGATGAACACCATCATTGAAAGAGGCATTATCTAGTGCCATTTTGTTTTCTTTCGATAG</td></tr>
<tr><td>39718032|F|0-30:A>G-30:A>G</td><td>TGCAGTCACCGCTGGCTTCAAATCATCGTCACCAATTGATCGAGTCACCTGTCCAAGCTCAAAAGAGGC</td></tr>
<tr><td>39716989|F|0-43:T>C-43:T>C</td><td>TGCAGGATGAATCGGAGTTAGGCTTCCGATTGGAACTGGAGACTGGAGTTAGGGGTTTGGAGGGCTTGA</td></tr>
<tr><td>46756753|F|0-58:G>C-58:G>C</td><td>TGCAGGTCTCACAAGCGACCTTGCTGTCAGGGTCCTGAGCTAGGCAGGCGTCGAGCACGGCATCAGAGA</td></tr>
<tr><td>39715952|F|0-33:T>G-33:T>G</td><td>TGCAGCCCTCATGCGTGCATTCAATTGTCTTTGTTGGACCTTCACTTTCATGCTTCTGTCGTAGATGTT</td></tr>
<tr><td>39715582|F|0-57:G>T-57:G>T</td><td>TGCAGCAGCTGGATTACTTGAGAAATTGAGTGTTGAAGAAAAGAAAGCTGATGAAGGGAAAGCTGGGGA</td></tr>
<tr><td>39728792|F|0-61:G>T-61:G>T</td><td>TGCAGTTGGTTCATATAATCATTGGAACAAGTTCCATGGGCTGTCCACTCGTGGCTCCACAGTGTTGGA</td></tr>
<tr><td>46754269|F|0-52:T>C-52:T>C</td><td>TGCAGAGAAAGATTGCTTTGCCCTGCAAGGCTTGTAGAGGAAAAGGGTTCTATATATGCAAATTATGCC</td></tr>
<tr><td>39723326|F|0-26:T>A-26:T>A</td><td>TGCAGGCATGCTTTTGCCACACTTTCTTTTGTTGCTGAGATCTTTATCTTTCTTTACGTTGGCATGGAT</td></tr>
<tr><td>39723688|F|0-22:T>C-22:T>C</td><td>TGCAGGTTGTGATGTTGGCCCTTATTTTCATTTTTGCCATTGTCCACAGTGGTTTGGCAAGTCTCCGAG</td></tr>
</table>

<table>
<tr><td>39727272|F|0-21:C>T-21:C>T</td><td>TGCAGTTGTCAACGATGATGACGAAGATGACGACGACGAAGTGCTCGAGCTTAC</td></tr>
<tr><td>39718430|F|0-29:A>G-29:A>G</td><td>TGCAGTGGCTTCTCACTCACTGATTTAGGACACCCTAGGGTAACTTCAACATAGCAGTTTTGCCCCTTC</td></tr>
<tr><td>39726746|F|0-23:T>C-23:T>C</td><td>TGCAGTGGCTATCATCTTGAGATTCTTGTTGCCAGTCTTTGCAAATGAAAATGCATAAACCAATGGAGG</td></tr>
<tr><td>39725474|F|0-48:C>A-48:C>A</td><td>TGCAGTAACTAGCAGCACAAAAGCTAAAGGCAAAATGGAGACTGTCCGCCCAAAAACGGCAAGCTGGGG</td></tr>
<tr><td>39715753|F|0-5:G>C-5:G>C</td><td>TGCAGGATTAGTTTTACACAGAGGGTTTCAAATTCCAATTACCATCTCATCTTCAATCACTAAACTTTT</td></tr>
<tr><td>39722226|F|0-15:A>T-15:A>T</td><td>TGCAGCAAAACCTCCATGATGAGCCTCCTTTTACTTACACCCCAATCCCTAAATTTCTAAGAGGAATCT</td></tr>
<tr><td>46756041|F|0-5:C>T-5:C>T</td><td>TGCAGCTTGGTGAGTTTTCTTCCGAGTTACCAGAACGACATGTCGTCGTCGGTGTCGTCGGAGGAGGAG</td></tr>
<tr><td>39724635|F|0-18:T>G-18:T>G</td><td>TGCAGAAGAAACTTCTACTGCTTGTGATGACAATCCCGATAGGTTGGAATTAC</td></tr>
<tr><td>39721884|F|0-66:T>A-66:T>A</td><td>TGCAGAGGTACTAATGCAAACATCGGCCAATCTTGGATTGTTCAACTCATTCCATTTTGCCTGAATTTC</td></tr>
<tr><td>39714069|F|0-20:T>A-20:T>A</td><td>TGCAGAATTATATACATATATAAAGAAATCAGTTTTCTACAATGCTAACAGCAACATTCATCTGGTTGC</td></tr>
<tr><td>46776397|F|0-30:C>T-30:C>T</td><td>TGCAGCGCCGCCTAAGTGTTCGATTGAGTTCGAATCACTTAC</td></tr>
<tr><td>39726875|F|0-44:G>C-44:G>C</td><td>TGCAGTTTTCTATTTTATTCTTGCATATTTTTGTGGTAACTCCAGCTTCCGTAAAAGTGATATAGCATG</td></tr>
<tr><td>39715140|F|0-45:T>A-45:T>A</td><td>TGCAGATTGCTCCGAACAGATTCTGTATGAAGCGTCTTTCAAGGATTTTGGGAGATACAGTGTTCAATA</td></tr>
<tr><td>39721273|F|0-25:T>C-25:T>C</td><td>TGCAGAACCAAATCCTCGACTCGGCTGAGTCGTCCGAGTTCTGGAGGTATCAAACCGCTAAGACCACAC</td></tr>
<tr><td>39715080|F|0-67:C>A-67:C>A</td><td>TGCAGATTATATCACAAATAAACAGCTCAGAGTGGAAGCACTAAATATTTATTCAGATACCTTGGGGCA</td></tr>
<tr><td>39724895|F|0-19:T>G-19:T>G</td><td>TGCAGCACTTGCTCGACTCTTACTTTAC</td></tr>
<tr><td>39722701|F|0-7:G>T-7:G>T</td><td>TGCAGCCGTCATTCGTCACCGTGTCGGTCACACAAATTGTATTGAATAAGCCGATTTCTTTTCTTTGAA</td></tr>
<tr><td>39728663|F|0-5:T>C-5:T>C</td><td>TGCAGTCAAATTACTCCTTATTTTTTAGGATCCTCATCCATATTATTAC</td></tr>
<tr><td>39726930|F|0-16:C>A-16:C>A</td><td>TGCAGAAAACTTGCATCAAAATTATCTTAC</td></tr>
<tr><td>39721138|F|0-53:G>T-53:G>T</td><td>TGCAGAAAAGAGACACTTCAATCAGCATATCATTTCAGAAGTTTTATGCTCGAGAAAGAAATAAAGGGA</td></tr>
<tr><td>39720270|F|0-12:G>A-12:G>A</td><td>TGCAGGAAACGAGTCTAAGAGATTAC</td></tr>
<tr><td>39727527|F|0-57:A>T-57:A>T</td><td>TGCAGCAATATGGTTTGCACTTTTCATCTCCTTGAATTTGACCTAAAAGAACAAAGCATTCATCTTTTT</td></tr>
<tr><td>39724157|F|0-26:C>T-26:C>T</td><td>TGCAGTGTCCACCGTTTGGCTCTCCGCTGTGGCGCAGCTGCTATTGATCTGATCGGCAGTGACTTCCAA</td></tr>
<tr><td>39727819|F|0-52:T>G-52:T>G</td><td>TGCAGTCTGAAGATTCCTTTCAAACAGATGATTATGAACTTGTTATTGTTGATATTTCAGAGCTGCCCA</td></tr>
<tr><td>39723477|F|0-12:G>A-12:G>A</td><td>TGCAGGGCTGGAGTATTTGCACAATTCTTGCAAGCCACCTATAATTCACAGAGATTTGAAGACTTCAAA</td></tr>
<tr><td>39714088|F|0-21:A>G-21:A>G</td><td>TGCAGAATTGGTGTCTTTCAGAAAGGGAAAACTTGTACCAAGAGATCATAACTTTCTGTCATTCAATTC</td></tr>
<tr><td>39720189|F|0-19:G>A-19:G>A</td><td>TGCAGCTGAGTCAAATGAAGTGGCTCTTCCCTTAC</td></tr>
<tr><td>46756767|F|0-26:T>C-26:T>C</td><td>TGCAGGTGAACCGGGAGACCGGGCGGTTGGAGCTGGTTTGTAACAATGCCGGCGTTCTGTTTGTGAATG</td></tr>
<tr><td>39713565|F|0-8:G>T-8:G>T</td><td>TGCAGAAAGGTGTTTTTTGCAATTGCAATTGCAACTATGCTTCTGAAAAATCTATCGTCACACGTACTC</td></tr>
</table>

39715077|F|0-42:C>G-42:C>G	TGCAGATTAGTTCGTCTACGGTATATCCTTTCTTGATGTACGCAGTGACTTCGTCCGGGGTCGCTTACT
39729487|F|0-45:T>C-45:T>C	TGCAGTTCTCGACAACTACAGTGAAATTTTGAAATCTTGTAATGCTCTGGACTACCACGACTTAC
39717037|F|0-41:G>A-41:G>A	TGCAGGCAAACATCACTCTTTGAGATGCTACATCCATGTGGGATCTTTACAAAGTCCAAACTAAGATCA
39722143|F|0-25:A>C-25:A>C	TGCAGATTATAAAGCAAAACAATAGATTTTGGGAGCTCTCTTATTCGAGAGTGTGAAATGTCAAGGTAC
39723735|F|0-45:A>C-45:A>C	TGCAGTAAAGTAAATGAGAAAAAAACTATTTAGTAGTGCCTCAAGAGAACATGGATTTTCATAAAAGGG
39727176|F|0-9:G>T-9:G>T	TGCAGGTCTGGCTAGGACATAAATGACTTTAC
39721654|F|0-29:A>G-29:A>G	TGCAGACTATTTCCACGTTCACTTGAAAGATAATGAAGAAATATAGATACAGATTGAGCCTCACTTTGG
46753791|F|0-19:A>G-19:A>G	TGCAGAACAGCGGCAGCCCATTTGGGTACTTTCCAATGGGTTCGATCCGGGTCCACTTTCCGGTGACCG
39718959|F|0-22:C>G-22:C>G	TGCAGTTTTCTTTACTTATCTCCGTCGCATTTTCAAAAATATTTTTAGCCATGCGGAGCAGATTTCTCA
39721750|F|0-41:C>T-41:C>T	TGCAGAGAGTTAGATATGATCGAGTCTTCCCTCCTTTGATGCACTTTTGCCTTCAAAAAATTGAAGATT
39727490|F|0-18:A>C-18:A>C	TGCAGATGCTGACAGCAGATAAACAGAACACGTAACTGAGGTTATACAATAATTTCTAGAGTTGCTACA
39714127|F|0-48:A>C-48:A>C	TGCAGACAAGTATCCGAGCCACGTTCATCCGAAGCTTCCCGATTACGAAGAAATCGCAGACAAATTCAT
46755528|F|0-43:A>T-43:A>T	TGCAGCCCGCTTAAGAAACGAAGGTTAGTTGAAAATGCCCAAAAGGAAAACGGGAGAAGAAATTCCATC
39716127|F|0-48:C>G-48:C>G	TGCAGCGATCTTTAGATCTCTTCTCTATGAGGCTTGTGGCCGGATTGTCAATCCGATTTAC
39714961|F|0-18:T>C-18:T>C	TGCAGATGAGTATTCTTTTCCCTCATATCTTCTGTATAAACTTTTCTTTGTATCGATTAGAAAGGAAAA
39724594|F|0-46:T>A-46:T>A	TGCAGAAAGAGTATTAGATGATTGGCTTCACTTGTCAATGGGGCAATGCTGATTAC
39728625|F|0-34:G>C-34:G>C	TGCAGTTCAACAGTAGAATAAAAGAAATGCTTATGAAATAATTCTTTTTCTTTTGAAATGAGCAAAGTA
39725070|F|0-14:A>G-14:A>G	TGCAGGACCAAAGAACCCGTTAC
46755683|F|0-14:C>A-14:C>A	TGCAGCGCAGACATCGCCGCCCCTCGCAGCGGCAGTCGGCCGGAGGAAGTAACGACTCGATGGTTTCGG
39729519|F|0-14:C>T-14:C>T	TGCAGATCAGAAAACCAAACACCATTAC
39722376|F|0-7:C>T-7:C>T	TGCAGCACTCACAAGAACTGAGAGTACCATCGACAGCAAAGAGTTGCTTACAATTATCAGTGCCTGAAA
39723730|F|0-35:G>A-35:G>A	TGCAGTAAAGAAAATTCACGAGCACTATCAAAGGCGAGCTTTCTTTCTAGGCTTCAGTCAGTCTCCGGC
39716232|F|0-65:T>A-65:T>A	TGCAGCTAAATTTCACACAATATGTTTAGGGGTTGATGGAGAGGTAATTTTTAGGTTTGTTGAACTCGT
39722399|F|0-21:C>G-21:C>G	TGCAGCAGACATAAGCCCCATCAGAAGAAAAAATGAACTCATGCTTCGATGCATTTTTACGGTCTTTGA
39722777|F|0-9:G>A-9:G>A	TGCAGCGATGTTATCTTGCAGCTCCTTCACCTTTTTGTCTTTCTCCTCGACCAAATTCTGCATGTCACG
39718424|F|0-45:A>T-45:A>T	TGCAGTGGCTCTCCAACTATGGTTGGTTCAATTTGGACTTGTGTTATGCACGTAATTGTTGAGTTCAAA
39722126|F|0-20:C>T-20:C>T	TGCAGATGTGCCTGAGATTGCTGAACTTGGTCTTCGAGTTGAGGTGGATCCAAAAACTCGACAGGCTAA
39723157|F|0-7:A>G-7:A>G	TGCAGGAATGAACTTGTGGGTCATCATATTCATCTTGCTAATTGCCATTGCTGCATATTTCATCAAGAA
39723477|F|0-6:G>A-6:G>A	TGCAGGGCTGGAATATTTGCACAATTCTTGCAAGCCACCTATAATTCACAGAGATTTGAAGACTTCAAA

<table>
<tr><td>39718674|F|0-63:A>G-63:A>G</td><td>TGCAGTTCTCCTGGTTTGTGGAGTTCTCATGGGAATTGAGTTGGTACCAGCTAAATTGGCTTCATGTTT</td></tr>
<tr><td>39716821|F|0-51:T>G-51:T>G</td><td>TGCAGGAATGGCTCTCCAATAATTGCCATATAGTTTACCATTCTAAACTTTTTATATGTTTTGTGACAT</td></tr>
<tr><td>46754021|F|0-36:C>A-36:C>A</td><td>TGCAGAATATGTAAATGATCAGTACCAAAAAGTTGGCTTCAGTAATATTTTATTGATAATTTCTATAGT</td></tr>
<tr><td>39726766|F|0-27:C>G-27:C>G</td><td>TGCAGTGTTGGACATGCCTGAAAGAGACCATTGAGAATTTGGTGTCAATTCTGGGAGTATCCAGCCAGT</td></tr>
<tr><td>39717497|F|0-57:T>C-57:T>C</td><td>TGCAGGTCATTTTCTTTGAAATACTTTATTTATTGATAATGACAGTCCATGTATTGTTCAATTGCGATG</td></tr>
<tr><td>39727338|F|0-32:C>T-32:C>T</td><td>TGCAGAAACGCTAGTCTTAGACGAGCTGTCTGCCTCTCACTTGCATACATCCAACACCCAATTTCTATA</td></tr>
<tr><td>39713592|F|0-20:A>G-20:A>G</td><td>TGCAGAAATCAAATTGTACTAACTGTTGTGTTTCCAAATATTGACATGTAAGATGATTTTTTTCCATTA</td></tr>
<tr><td>39713705|F|0-61:C>G-61:C>G</td><td>TGCAGAACCCAACTCTTCTTCCCCCATTTACTCCGCTGCTTCAACTTCCTCGACATCAACACCACAGTT</td></tr>
<tr><td>39716591|F|0-14:C>G-14:C>G</td><td>TGCAGCTTCTCGACCACGACCTTGATGCCGTTCTTTTTCTTCATGGTTCTAGATTTAGCCATATGACCA</td></tr>
<tr><td>39714237|F|0-7:G>C-7:G>C</td><td>TGCAGACGATGCGTCTTCTAGATCACCCAAACGTCGTGTCCCTGAAGCACTGTTTCTTCTCAACGACTG</td></tr>
<tr><td>39715579|F|0-9:T>G-9:T>G</td><td>TGCAGCAGCTGCTCCACCAGCCAACATTGGCCCCATGCCGTGGTGTCCGTGTCCATGGCCTGTAACGAA</td></tr>
<tr><td>39714514|F|0-59:C>T-59:C>T</td><td>TGCAGAGCCTAGATTTATTGAGCAGCAGTATCCTTCTCAATTCTGAAGTTTCAGAGAAACCGGTGACTT</td></tr>
<tr><td>39726049|F|0-67:G>A-67:G>A</td><td>TGCAGCAGCATCATTACGGGACTTGTATGTACAAGGAATGTTACGAACTTCATCTCTCAACCTACACGA</td></tr>
<tr><td>39725542|F|0-5:A>T-5:A>T</td><td>TGCAGAAATCAACAGACGGTGGACCACCCACAAGTTCCACTTTGTCCGATGCGTACTATTGTCACTTAC</td></tr>
<tr><td>39725690|F|0-41:A>G-41:A>G</td><td>TGCAGAGAAATTGCCAGAGAACTGTCCACGTGAAGCACTTCACTTTTTTGAGGCCTCCCCCACCCAAAA</td></tr>
<tr><td>39727301|F|0-38:T>A-38:T>A</td><td>TGCAGTCATTCTCCTCTCAATCCAAGAATTTCATATATTGCATGTCTAGGATAGTTTCTCATTATCCTT</td></tr>
<tr><td>39728541|F|0-28:A>G-28:A>G</td><td>TGCAGATCATCTCCAAAACAGAAAGCACAAGTAAGCATACCTCCTTCAACCATCTAAATCCAAGTCGAG</td></tr>
<tr><td>39725751|F|0-45:C>T-45:C>T</td><td>TGCAGAGCCGAAGCTTCGAGCCGGGTTGATACCGGCGCCGGTGACCGGAATGGTGGCCAAATTCACCAC</td></tr>
<tr><td>39715675|F|0-22:A>T-22:A>T</td><td>TGCAGCATATTATGAATTTGTCACAGATTCAAAGATTCACTCGTTTGTTGAAATTTTTGATATGCAATG</td></tr>
<tr><td>39726161|F|0-18:C>A-18:C>A</td><td>TGCAGCCTAAAATATTCACCATTCCGTATCATCAGTCAGCGAACCATACAGTAAGTTTCTACATGTGAA</td></tr>
<tr><td>39723709|F|0-24:G>A-24:G>A</td><td>TGCAGGTTTTTTTGGATTGTCCAGTGAAAACAAATAGAGGATACCTTTTGGTATGGCTTTGGTTGCTT</td></tr>
<tr><td>39720109|F|0-6:G>T-6:G>T</td><td>TGCAGCGGTGGCGGCGGCGACCGCGATGGCGTGGTTTCTGTCGCCGGCCGTTAC</td></tr>
<tr><td>46757525|F|0-42:T>A-42:T>A</td><td>TGCAGTTCATGAAAGACTTCTTCGGACAAGTGAAGACTTTGTTTGGCCTGTAGATTCAACCTTCCATGG</td></tr>
<tr><td>39722176|F|0-21:C>T-21:C>T</td><td>TGCAGATTGAACGCATATCATCTCGATATCAGCCGTCCCTTGTTTTATATAGTCTTGGGAACAACTTTT</td></tr>
<tr><td>39719505|F|0-18:G>T-18:G>T</td><td>TGCAGACAGTCAATTCATGTTGGAAGGCTGACTTAC</td></tr>
<tr><td>39726161|F|0-11:A>T-11:A>T</td><td>TGCAGCCTAAAATATTCACCATTCCGTATCATCAGTCAGCGAACCATACAGTAAGTTTCTACATGTGAA</td></tr>
<tr><td>39723157|F|0-43:G>T-43:G>T</td><td>TGCAGGAATGAACTTGTGGGTCATCATATTCATCTTGCTAATTGCCATTGCTGCATATTTCATCAAGAA</td></tr>
<tr><td>39715382|F|0-18:T>C-18:T>C</td><td>TGCAGCAATGGACATCGATCACTATTCCTTTTCTGCTGTGCTCAGATCATGCTCAGATTTGGCTACCTT</td></tr>
<tr><td>46754535|F|0-50:T>C-50:T>C</td><td>TGCAGAGTTTAGATACCACATATTCTCCAAAGAATTGCCTCTTGTAAGTTTTTCTTTTCCTATGTCTCA</td></tr>
</table>

<table>
<tr><td>39716636|F|0-67:T>C-67:T>C</td><td>TGCAGCTTGTTTCCTTTCCAACTCTTTTATTCTCTGAGATTTAGATTTCAAATGGATAAACCAATTCTT</td></tr>
<tr><td>39714269|F|0-58:A>G-58:A>G</td><td>TGCAGACTATGGTACAGCGTCTACAGGGGATGGAGCAGCGACAGCAGCAGATGATGTCATTCCTTGCAA</td></tr>
<tr><td>39724160|F|0-15:A>G-15:A>G</td><td>TGCAGTGTCGTTGAGAGGAGAGTAGCTCCAGCTTTCTGATATCTGTCAATCGAGAGATCAATCTGTCAG</td></tr>
<tr><td>39714983|F|0-26:A>T-26:A>T</td><td>TGCAGATGCAACAACATAATTAGAACACTGATGCAAGAACGCAAACAAGATTGACCAAAATAGAGCCAA</td></tr>
<tr><td>39723998|F|0-48:T>C-48:T>C</td><td>TGCAGTCTATATTCCTCCATGCTCTTTCTGCAACAACCCCTTTTCTTCTAGCCCCATTACTCCAACAGA</td></tr>
<tr><td>39727916|F|0-45:A>G-45:A>G</td><td>TGCAGTTTGAAGGTCGAACTTTGGGGTATAAAGGATGGTCTCCCCATAATCATTCTCAAGCTTTTTATA</td></tr>
<tr><td>39721623|F|0-49:A>G-49:A>G</td><td>TGCAGACCTGTCACTTGGTCCAAAGTTGTACCATCTAGAAATTGCATTGAGTCACTATAAGAACTGGTC</td></tr>
<tr><td>39716284|F|0-19:T>C-19:T>C</td><td>TGCAGCTATATGATTTGATTGAGCTGAAAGTGCAAGGAGATGGTAACTGCCAGGTTAGTTCTTCTTACT</td></tr>
<tr><td>39716049|F|0-14:T>A-14:T>A</td><td>TGCAGCCTCTCTGCTCAGGCCCTTTGTAGCTTGCAGAGATGGTTGGATAAAAGTGGATGCCAATGAGTG</td></tr>
<tr><td>39724043|F|0-50:G>A-50:G>A</td><td>TGCAGTGACAGACAAATCAACATCTCTTCTCCACATGGAGAATTGGGCAGGTTCAATTACAAAGTAAAT</td></tr>
<tr><td>39715084|F|0-34:A>T-34:A>T</td><td>TGCAGATTATTGACATCTCTCGAAACAACTTTACAGGGAATATATCTGGGAGAAGCCTAGCAACATGGA</td></tr>
<tr><td>39719944|F|0-25:T>C-25:T>C</td><td>TGCAGCAGATTTTTTCCCGGATAAATTCCTAGTTACCCTACATTAC</td></tr>
<tr><td>39714081|F|0-26:C>A-26:C>A</td><td>TGCAGAATTCTGATGGGATTCAGTGTCTTAGTGATGGTATGATCACACCCGTTTCAACAATATCTATCT</td></tr>
<tr><td>39721119|F|0-32:A>G-32:A>G</td><td>TGCAGAAAACAACTAGTCCATGCAAAAGATACAAGAAGGGTCGACATACTTTGTTATCGTGTCTCTTTG</td></tr>
<tr><td>39724695|F|0-17:C>T-17:C>T</td><td>TGCAGACAGACTTGTATCGTAACTTGGCTTGAATGTGTTCCTTCTCAAGCTATACTCTTAC</td></tr>
<tr><td>46768976|F|0-9:G>A-9:G>A</td><td>TGCAGCATCGACTCTCTCATTATCATCATCAGATTCACTTGCACTTGCATCTGTAGCCAAATAGAGAAG</td></tr>
<tr><td>39724664|F|0-38:G>A-38:G>A</td><td>TGCAGAATATTTTATTATTCTCATTTATGAGAACAACAGGTTTAC</td></tr>
<tr><td>39714194|F|0-7:T>C-7:T>C</td><td>TGCAGACTATTTTCTCCCATGCAAAGTTATGTATTATTATACATGAATAAACTGTAGGAGTTTCTTGTA</td></tr>
<tr><td>39723190|F|0-33:A>G-33:A>G</td><td>TGCAGGACTTGGAGGTTTGGGCAGTGGACCAGAACTTTGTGTATGGTCATTACTTTCCCTTAGGTAAAG</td></tr>
<tr><td>39728908|F|0-42:A>C-42:A>C</td><td>TGCAGAGATTGAGAAGTCTACCCAAATTGATAGGGACTTCACATAATTTTAC</td></tr>
<tr><td>39724021|F|0-23:T>C-23:T>C</td><td>TGCAGTCTTTCTTTGTCAACACATGAAGCTAAACGGAGGGCTTTACACCATGCTTCCTTCTCCCAGGAA</td></tr>
<tr><td>39725622|F|0-64:A>T-64:A>T</td><td>TGCAGAATCCGAGAGCTTTCTTCCCTAGCTTTGCTGATTCCATGTTGAAGATGGCAGCCATTGAAGTCC</td></tr>
<tr><td>46762338|F|0-45:T>C-45:T>C</td><td>TGCAGAAAATATATGACATTCATATGGCAAACTTTGTTCTTGTTTTAGAAGTTGTTACCCACCTGGTTC</td></tr>
<tr><td>39726094|F|0-9:T>A-9:T>A</td><td>TGCAGCATTTCTCAGCTCAAGTCCTTGCATCAGCTACACACCCATAGAATCTCTAGCATTTCAAATTAC</td></tr>
<tr><td>39721554|F|0-38:G>A-38:G>A</td><td>TGCAGACAACGACACTGAAGATGGTAGAGTATTTTCCTGTGAGAGTTCCCAATGTAGGCTTGAAATTTC</td></tr>
<tr><td>39721315|F|0-16:A>C-16:A>C</td><td>TGCAGAACTTTAACACAATCTGTGAAGCTGACGACTCTTCTTCAACCTGTTATGTACGTACCTAGTGCA</td></tr>
<tr><td>39728071|F|0-18:C>T-18:C>T</td><td>TGCAGGTAGAGATGCAAACATCATAACGGATCATTCTGGGAATTTAC</td></tr>
<tr><td>39729518|F|0-13:C>A-13:C>A</td><td>TGCAGATCAGAAACTCGAACAACATTAC</td></tr>
<tr><td>39717950|F|0-21:C>T-21:C>T</td><td>TGCAGTATGATGTACAGTGACCGGAGGCAGTATATGATGGCCAATAACGGCCAATGCTTTGTCCGTTTA</td></tr>
</table>

39726531|F|0-38:C>G-38:C>G	TGCAGGTCTGCTCTTTTCACCGTTATATTTAGGTGTTTCCATTTAGAGGCTACTACTCCAGGTGTTTGA
39726206|F|0-22:C>T-22:C>T	TGCAGCGGAGAGGTTGAGATTGCCGTACTTGAGTGCTTATCTGAACTCGCTCGGTGCTAATTTCCGGCA
39723484|F|0-21:T>A-21:T>A	TGCAGGGGAATCTCCACTCACTGCTTCCCCTAATATCCTCCCTTTACCACATCATGGGGGTTTACAGGA
39714074|F|0-38:A>G-38:A>G	TGCAGAATTCATGACCGCGGGAGCTGGATTGAAGAACAATGGGTCATTGACATTTATTGGGTTGGGTGT
39717235|F|0-29:G>T-29:G>T	TGCAGGCTTTGGCGGCCGAGTTCAATATCGCTGAGACGTGTTATTTGATTCGCATGAACGACGAAGACG
39726728|F|0-6:G>A-6:G>A	TGCAGTGCATAGTTTTCAAGTTGTTCATACAGGCCCGGAGCCAATGCTAGAGAAAATAATCTGTGAACA
39722311|F|0-23:C>T-23:C>T	TGCAGCAATCTTGCATTTGAAGACATCGAGATAAGATCTGCAAATTCTGATAGAATCACTCTAGCCTCA
39718161|F|0-18:G>T-18:G>T	TGCAGTCTCACCTCTTCCGGCGGATAATACTAACAAGCAAGCAAAGAAGGACAATACAAACCATCCACG
39721040|F|0-20:A>G-20:A>G	TGCAGAAGGCTTCCTTCTGGATGTGTTGTTCAAGATATGCCCAATGGATATTCCAAGGTAAAAACATAA
46756765|F|0-40:A>G-40:A>G	TGCAGGTGAAACACAACTCATGCCTCTCCGAAACGCCTCCAAAGGAGACAACAGGATTCTTCTTAGGGA
39725092|F|0-17:G>A-17:G>A	TGCAGGATTCGAGATTGGGGGATACAGGATGAGGATGAGTCGAAGTTAC
39713799|F|0-43:C>T-43:C>T	TGCAGAAGAGCCAGAATGTCACCGAGAGCATGGTCTCCATTCTCGGCTCCTTCGACTCCCGTCTCTCCG
39724415|F|0-17:A>T-17:A>T	TGCAGTTTCCGAAGCTAATCTCAAGCGAGCTTCCCAAGAAAGAGAATCGCGATTGGTTTTTTCATGGAG
39718633|F|0-65:T>G-65:T>G	TGCAGTTCCAGATCATTTTGGAGCCATTTTTGTGGAAAAAGTATAGTTCATCAAAGGTGTCAGAATATA
39721365|F|0-12:A>G-12:A>G	TGCAGAAGATCTATCCACAATCGAAGGAATTGCGACAGTGTCGCTTCTACATTCCTTCATTCCCACTCA
39723786|F|0-44:T>C-44:T>C	TGCAGTACCGATTGCTTTACAAAAGGTATGTTGCTTGTGCTAATTAGTAGACTCTGTTATACTTCCTCG
39723925|F|0-31:A>G-31:A>G	TGCAGTCATCCATTGCAAGTCACCAGCTCCAATTGTTCCTTTATGTCCTTTGAAATCTTCATGTGTCAT
39723266|F|0-24:A>G-24:A>G	TGCAGGATGTAATTCTCTTATTTCACTTCTTTTCTATTCCCTGATAAGCAATAATATTAGCAGCAGCTC
39713681|F|0-60:G>A-60:G>A	TGCAGAACATGCACCAGAAACGTTGTGTAGGATGGATTCTCTTGTCATTTGGGAAATACTGCAAACTTT
39716167|F|0-68:A>T-68:A>T	TGCAGCGGCAGCAGCAGTAGGAACAACACCTGTGGATATTCCCGGTTTTGACAAATGCAGCAGCCTGTA
39721657|F|0-20:A>G-20:A>G	TGCAGACTCAGTTGCTTGGCACCGGCTAGAGCTGCAATTCCTGAATCTGTGATCGAGCATTTTGAGACA
39714974|F|0-63:A>G-63:A>G	TGCAGATGATGGGAAGAGATGGGATGCAGAACCGGACCATCTGAGAGAGGAAGTAAAAGAGTTAGGTAG
39721910|F|0-44:A>T-44:A>T	TGCAGAGTCTAGACAGCTACACGTGTACAGGTTTTTACTGGAGAAAAAAGATGAGATAAAAAGCCTGTT
39721215|F|0-14:A>G-14:A>G	TGCAGAAAGTAAGCAACCTCGAGGATCGGTGGCTTCAAAGGAGATGCATAGGCAGCTTCTCCATGTAAG
39727848|F|0-51:T>C-51:T>C	TGCAGTGATTGGCATTGATCATGAGGTCACAAACTATTTCAATACTAATTTTTCACATTACTTCATATT
46776598|F|0-8:T>A-8:T>A	TGCAGATCTAGGAGAAAAAAAAATTCTTCTAGTGGAGATAATTTGGAATCTACAACGCCAGGTGCTTTT
46760832|F|0-37:A>C-37:A>C	TGCAGCAAATCTTAAAGAAACCCTAACATTCTTGTTGAGTATATCTTAC
39721733|F|0-45:A>T-45:A>T	TGCAGAGACGGTGGTGAACATTTCGCCTTGGATTTTGAGTGTGGCAGCGAGTTCTATGGATAGAAGTTT
39713776|F|0-21:G>A-21:G>A	TGCAGAAGAATGAGAAGGCTCGGACTGCATTGTATTACTTGCTTCTTTTTTCATTCTCTACTACCTATG

<table>
<tr><td>39720223|F|0-43:G>C-43:G>C</td><td>TGCAGCTTCCCCAGGCGCTGTGCAACCTACTAAGAAATCCTACGGAGATCTTAC</td></tr>
<tr><td>39720865|F|0-21:G>A-21:G>A</td><td>TGCAGTTGGGTGTGAAACACAGGTTAC</td></tr>
<tr><td>39723697|F|0-66:T>A-66:T>A</td><td>TGCAGGTTTGCGCCATTCATCCCTCTCTCTCACCTTCTCTTCTTTTCCTGTTCATCGTTTCGGCTATAT</td></tr>
<tr><td>46762726|F|0-21:G>T-21:G>T</td><td>TGCAGACATTGTGGCAAAAAAGAGAGCTTTCAACTAACACATAATTTACTTTCCTAGTAATGTCAGCCA</td></tr>
<tr><td>39722001|F|0-19:T>C-19:T>C</td><td>TGCAGATCATAAAGCTCAGTAGTCATAAAGTTTGGTGCTGTTCTTTCAATTTCTTTTGGTGTGGCCTTA</td></tr>
<tr><td>46776599|F|0-27:G>T-27:G>T</td><td>TGCAGATCTAGGAGAAAAAATAATTCTGCTAGTGGAGATAATTTGAAATCTACAACGCCAGGTGCTTTT</td></tr>
<tr><td>39725462|F|0-7:C>T-7:C>T</td><td>TGCAGAACAGAGAGGCCGTGTCGACGACGATGTTCCCGACGACGACTATGGAGCCATCGATGAAGTAAT</td></tr>
<tr><td>39725751|F|0-24:G>A-24:G>A</td><td>TGCAGAGCCGAAGCTTCGAGCCGGGTTGATACCGGCGCCGGTGACCGGAATGGTGGCCAAATTCACCAC</td></tr>
<tr><td>39722695|F|0-31:G>A-31:G>A</td><td>TGCAGCCGGCGGGAAGGCCGGAGATGACGTGGATCTCCCTTAGGGTTCCGACGTTGCCGTCGCCGACGA</td></tr>
<tr><td>39724388|F|0-64:T>G-64:T>G</td><td>TGCAGTTTAACCTTATTCATACAAATGGATAAACCAGAAACATGAAGTATTGTGCTATGTTGTTTTTTT</td></tr>
<tr><td>39725516|F|0-23:T>A-23:T>A</td><td>TGCAGAAACATTACAAAAATGAGTATGTGAACTTTTCGCAACTTCATCTCCGTCCTCTAAGAAAACCAT</td></tr>
<tr><td>46753609|F|0-8:A>T-8:A>T</td><td>TGCAGAAAATTTTCGGAGAAGATGACTCAGAAGGCGAACCTATTCAAAGGGCAACAGAAGAGGAAAACC</td></tr>
<tr><td>39718292|F|0-36:C>T-36:C>T</td><td>TGCAGTGAGTGATTCCAAGCTCTCATTTTTCCGGATCGCTTCCTCTATGCGAGTGGCCAAAACATCATC</td></tr>
<tr><td>46764483|F|0-8:C>T-8:C>T</td><td>TGCAGGCGCGGCAAGAGGCTGTGGTTGGCAGCACTTGGCTACTTACATAAGCCTCCCGACGTTCTATAT</td></tr>
<tr><td>39718593|F|0-14:A>G-14:A>G</td><td>TGCAGTTATTGGAGACATTGTATTACAAGTCTTGTTTACTTGAAGTTTTTAGCTATCACTTTCAATGAA</td></tr>
<tr><td>39722812|F|0-26:G>A-26:G>A</td><td>TGCAGCGGCGGAGAGGAGGAGGACCGGCGGCGAGATTCAGTTGAGGAGCGGTGGTTTCAGGCGAGATTG</td></tr>
<tr><td>39715211|F|0-65:C>A-65:C>A</td><td>TGCAGATTTTGGCGGGTTTTAGAGTAGGAAGCGATGAAGGAAAGTATCGAGCCGAGTGCGAGGGACAGT</td></tr>
<tr><td>39726728|F|0-8:G>A-8:G>A</td><td>TGCAGTGCGTAGTTTTCAAGTTGTTCATACAGGCCCGGAGCCAATGCTAGAGAAAATAATCTGTGAACA</td></tr>
<tr><td>39717946|F|0-22:G>A-22:G>A</td><td>TGCAGTATCTTAGAGACTACCAGACTTTTGCTGGCACAGCTCCAAGACGTCATCAGAAAAGGTTAGAGT</td></tr>
<tr><td>39728670|F|0-28:G>C-28:G>C</td><td>TGCAGGAGTCCTTCCAGTGCAAGTGGCTGAATAAGAAGATTGATTAC</td></tr>
<tr><td>39725897|F|0-35:G>A-35:G>A</td><td>TGCAGATGTGCGGCAATGGAAGTGAGATAAAGAAAGAGAAGATAGATTTTCATTTTTCTTAC</td></tr>
<tr><td>39715635|F|0-23:T>C-23:T>C</td><td>TGCAGCAGTGGGGAGATCATGAATTACATGAGTGTTGATATCCAAAGAATCACAGACTTCACTTGGTTC</td></tr>
<tr><td>39728901|F|0-8:C>G-8:C>G</td><td>TGCAGTTTCGATTTGTTGCTAGCAAATCCCTTTCTTCAACAATTTACTGACCCATATTTATGATTTTTG</td></tr>
<tr><td>39724357|F|0-25:T>A-25:T>A</td><td>TGCAGTTGGCTGCACAAGGGGTCATTGGTAAAAGGGTTGATGAGATGGAGTCAGGCTTTATGATGGCCT</td></tr>
<tr><td>39722593|F|0-15:G>A-15:G>A</td><td>TGCAGTCAAGGTTTGGTTCCTAGCTAGCAATGTTTGATCTAGGGCAACAATGGCATGCTCCATGGTGGC</td></tr>
<tr><td>39725739|F|0-68:A>C-68:A>C</td><td>TGCAGAGATTTCTTTCCTTGATGCTAGGTTGTATCAGAATCAGAATTCCCCCAGGGATCCTATCTTGTA</td></tr>
<tr><td>39714590|F|0-63:G>T-63:G>T</td><td>TGCAGAGGGACAATGATCTTGAGTTGAGGGAGTAATCAGTTGTACAAAAAGCAAATGGTGAGGGCTGTA</td></tr>
<tr><td>39722399|F|0-39:C>G-39:C>G</td><td>TGCAGCAGACATAAGCCCCATCAGAAGAAAAAATGAACTCATGCTTCGATGCATTTTTACGGTCTTTGA</td></tr>
<tr><td>39715249|F|0-44:T>C-44:T>C</td><td>TGCAGCAAACTTCAGAGTGATGCGTTGAGGGAAGCTATTTCATCTATCTTCAGTGATAGCAGCGAAAAG</td></tr>
</table>

46767014|F|0-32:A>G-32:A>G	TGCAGATAGTGTAAATATCAGTACAAAAAATCATCGTTAC
39724835|F|0-12:C>T-12:C>T	TGCAGATTAAGGCGTGTTTCTCGGCAAATGGGTTTTTAC
39726930|F|0-25:C>T-25:C>T	TGCAGAAAACTTGCATCAAAATTATCTTAC
46755473|F|0-18:G>A-18:G>A	TGCAGCCATGAAAGCTCTGGGTCTTGCTCACTTGAAAAGAATTTAGTCAAGCCTTGATGGAGCCTAGCT
39716067|F|0-37:T>A-37:T>A	TGCAGCCTGTTTCTTTTTCTAACTTGTAAATTTTGGATCCCTTGCTGTCATTGTTTACTAAATTCATGT
39723114|F|0-57:C>A-57:C>A	TGCAGGAAACTCGAGGAAGATTGTTGATGAGAAAGTTTCTTGTTCGAGGGCTGAATGCTGGGGCTTTGT
46756987|F|0-40:C>T-40:C>T	TGCAGTACGATGCATCCCAAAATCAATCTCATCTGCCCTGCTGTAGTCCCTATGATATCTATTACGAGA
39713493|F|0-32:A>G-32:A>G	TGCAGAAACATATGGGTACTGGATCCGTTGGGAGAGTTGGATAAAAAGAAACATAGGAATTCAGTGTAG
39716831|F|0-56:A>T-56:A>T	TGCAGGACAAAACGGACAAACCTTTCCATGAAATTAGCATAAAATAGACAAGGGAAAGGGAGTCTCAAA
39727089|F|0-5:T>C-5:T>C	TGCAGTCGTTGTAAGAAACCTAAGAGAACAGTTAGCATTTTTTCTTCAGCTATTATTTTAC
46763290|F|0-21:G>T-21:G>T	TGCAGATTCTTGCTCAGTTTCGCTTTCAACACGCCGATGTCGGAAAATTGCGGCTCCGGTGACCGGCCG
39727123|F|0-10:G>C-10:G>C	TGCAGCTGCTGAAGGAGGCATATTATTTCATATGCTTTTCAACTTGTTTCTTTTAC
39725342|F|0-18:A>G-18:A>G	TGCAGTGTCGGTAGCATCAATTGTTAC
39715555|F|0-67:T>G-67:T>G	TGCAGCAGCCGCTAAGGTAGCCATGGATTCAGATTTTCTTCCCTACAGAGAGACTGAGAGAGAGAGATC
46757642|F|0-59:T>G-59:T>G	TGCAGTTGCTGCTGCTGTCCAAATCCTAATTTCAAAGAAGGTAAAGTTAGAGCAAAATATGATAGAAAT
39716443|F|0-16:T>A-16:T>A	TGCAGCTGCTCTGCCTTCAGGTCATGTTTCTGTTGCTGGTTCTACACAGGTTCAAGCACAACTACCAAG
39725622|F|0-65:G>A-65:G>A	TGCAGAATCCGAGAGCTTTCTTCCCTAGCTTTGCTGATTCCATGTTGAAGATGGCAGCCATTGAAGTCC
39714677|F|0-36:A>T-36:A>T	TGCAGAGTGCATGTAAGATATAGCTCCTGCAACTTCAGAAGCGATTTTGAGACGAGTTTCCCATGGAAG
39717107|F|0-8:A>G-8:A>G	TGCAGGCCAAAACTATAATATTTTCACCAGCGCATAACACATACATAAGTGTCTTTTCAAGATAACTAT
39715175|F|0-7:T>A-7:T>A	TGCAGATTTCACAAACCTCAAAACGATTGAAAGAATCACCTTGGTGTGATACCATTTGTACTAATCAGG
39725720|F|0-38:C>T-38:C>T	TGCAGAGAGAGCAAGTGATCGATCAACGTGTTCCTTCCCTCTTCCTTGTTGCTCCGATGTTCGTCGATC
39717099|F|0-30:C>T-30:C>T	TGCAGGCATGTACATGGAATCACCAACCAACGGTGCTCCACAATCAGCTAGTTGTGCTCGAATCTGCAT
39726043|F|0-32:A>T-32:A>T	TGCAGCAGATCAAGAAACTGCGCTCGACGACTACACCAAAGATGGAAGCGTGGATCTCAAAGGCAACCC
39721134|F|0-65:T>G-65:T>G	TGCAGAAAGCTAGGAAGCAGAGGAGCATTGAAGACGGCCATGAAAAGGTAAAGGTGCAGCTAATTTTTC
39722189|F|0-37:G>A-37:G>A	TGCAGATTGTACCCCAGCTGCAATCTGGAAAGAACGTGATGATAGCAGCACATGGAAATTCATTGAGGT
46763722|F|0-8:G>T-8:G>T	TGCAGCCAGAATTGATATTCTGGATATGGAGATAGAGGAAGGGAAGGAGCTTGCACCGAGACGAGCCAG
39723598|F|0-45:C>T-45:C>T	TGCAGGTCCTGCTCCAATGATAACAACATCATATTCTATAGACTCCCGTTCCGCCTCACTACTGAATCT
39721846|F|0-39:A>G-39:A>G	TGCAGAGGAGAATGCAAAGCAAGAAAAAGGAGCTGAGCAACCATCCGGCTCACAGGATGCAACTATGTC
39725462|F|0-15:C>T-15:C>T	TGCAGAACAGAGAGGCCGTGTCGACGACGATGTTCCCGACGACGACTATGGAGCCATCGATGAAGTAAT

39728541|F|0-11:T>C-11:T>C	TGCAGATCATCTCCAAAACAGAAAGCACAAGTAAGCATACCTCCTTCAACCATCTAAATCCAAGTCGAG
39717744|F|0-29:G>A-29:G>A	TGCAGTAACCATCGGCGTCTATAATTTCCGACATGTCGAACAACGTCGGTGCTCGACACCGGTAATCCG
39721740|F|0-33:C>T-33:C>T	TGCAGAGAGAATGTTCAGAATTTAGTTCGTTGCCTTATTCATGCAAGCAAAAAAGAAAGAATCATCATT
39722511|F|0-10:A>T-10:A>T	TGCAGCATCAAAAATGCAAGTTTATGTCAAAAAAAACACAGACCATATTGCATCAAGAAATAGAAAAAG
46764552|F|0-14:G>C-14:G>C	TGCAGGGCAATGGTGAGGGGAAGAGGCTGAAGGTGGCAGCTTGTTGCAAGCATTACACAGCCTATGATC
39724575|F|0-27:A>C-27:A>C	TGCAGAAAACTCACCATATCAATGACAACTTCACTTCTTTAC
39719966|F|0-38:T>A-38:T>A	TGCAGCAGGGGTCGGATATTCACACCAAACATCAATCTTCTGACATATTCTTAC
39722798|F|0-52:T>C-52:T>C	TGCAGCGCTGTTGATTGATTGGGTCTGAATCACTATAAGCTCGAATACCCACTGCCCAAGGATCGTTCT
39726935|F|0-13:A>G-13:A>G	TGCAGATAATTGAAAATAAAACTTTTACACGTGTTGCAATATTTAC
46753848|F|0-21:A>G-21:A>G	TGCAGAAGAAACGCTAAAATCACTTATTGCAAATGGTGAAGCTTGCAGGTAAGCTTTGAAAAAACGATG
39722375|F|0-53:G>T-53:G>T	TGCAGCATTCACAAGAACTGAGAGTACCATCGACAGCAAAGAGTTGCTTACAAGTATCAGTGCCTGAAA
39714216|F|0-31:C>G-31:C>G	TGCAGACCTCTCATTCACATGTATGAAGTTGCAAAATTCCTTTTGGAGAGCACATATTTGAAACATGGA
46768934|F|0-35:T>C-35:T>C	TGCAGCAGCCAATGAAATGGCCACTTTTCTGTTTCTCTAGAACGGAAAATTCTGTCACAAGTGGAAGGT
39721343|F|0-39:T>C-39:T>C	TGCAGAAGACAGTGACCATGCCGAGAGTTTTACAACTAATGTTCGTAGACTTGGTCAAGCAAATTACCA
39719698|F|0-23:T>C-23:T>C	TGCAGATATAGAAATAATCAAAATATCTCTTCAAAGTACAAAGTTAC
39722020|F|0-68:G>C-68:G>C	TGCAGATCCTAAATGACTAGCCAATTTTCAAATAGTTTGGAAACCAGAACATACACAAATGGATAAGGG
39724377|F|0-64:T>A-64:T>A	TGCAGTTGTGCTCACTTCTTGCATACCCTGATATCAAAGTAGTCCATGCCACCACATCCCGCGCTGGAA
39727834|F|0-36:G>C-36:G>C	TGCAGTCTGTGGCTGGCGTTGGCAGCCAAACCAGCAGAGCACCGAGAGATTCTGTGGAAGCAAATTGAA
39718939|F|0-57:G>A-57:G>A	TGCAGTTTTACATTCAAAAACTTTAGCAAAAGAAAAGCTTCCCTGACCACAAACTAAGAAGAAAAAAAG
39726300|F|0-52:C>T-52:C>T	TGCAGCTGCTTTTGGCATGGCCAAGGCTATACAAAGATGAACTAAACCCCCCCATGTCAATCTCTAATC
39718404|F|0-30:A>G-30:A>G	TGCAGTGGATGTGCAGTAGCATCAAGATTTACAACAACTGACATTGCAAAGGAAGACAATGTTCACAAG
39715456|F|0-48:C>T-48:C>T	TGCAGCACTAATTCTCCCAATTTGATGCAGAGAATTGGCAAGGGCCGACTTACCAACATTTGGAATTCC
39714752|F|0-27:G>T-27:G>T	TGCAGATAATCATGGAAGAGTTCAAGTGATTTTCATCTCGCAACCATCCGAAACCGGAGTGAATTTGAA
39720421|F|0-23:T>C-23:T>C	TGCAGGCTGCAATTTTATAATTATTGGAGTTGCTGTTTAC
39722176|F|0-63:A>G-63:A>G	TGCAGATTGAACGCATATCATCTCGATATCAGCCGTCCCTTGTTTTATATAGTCTTGGGAACAACTTTT
39725039|F|0-57:C>A-57:C>A	TGCAGCTTGTTTTTCTTGGACTTGTGTGCTTCACACTCTCCCATATCCTCCTTCTCTCGAGATCGGAAG
39727511|F|0-13:A>T-13:A>T	TGCAGCAGAATGCAGATCGGAGAGAAAAAGGCAAAACCAAATAACTTCTCACAAGCATGTTCTCACCAT
39723401|F|0-50:G>A-50:G>A	TGCAGGCTGAGGACAATCACCAGGACAATGAGCAGCAAAATGATGATGCCGATAATCGTCCATTTCCGG
39725365|F|0-24:A>T-24:A>T	TGCAGTTCATCAACTGTATATATAAACTGAATAATTCAAGCATCATTAC

39715610|F|0-12:T>C-12:T>C	TGCAGCAGGTGGTTGGGAACCTAGGATGACTGATTGATAGAAGATACCTTTATTGCCTATTGGTTCATC
39725703|F|0-31:G>T-31:G>T	TGCAGAGAAGATGGTTATTTCCACAATTTTTGCATCTTGCTTTACTTTTCTTGAATACTAAAATGTAAG
46768554|F|0-27:C>T-27:C>T	TGCAGAGCAGCTAAGATCATCAATCTTCGACGAGATCACTAAACTGTCCTCTGAATCGTCGCTTTGTGG
46772371|F|0-60:A>G-60:A>G	TGCAGTGAGCTCATTTCAGCTCAGTGTTGGTGCAGCCGGAACCTCGAATAAAACTGTCAGACTTCCAAA
39713930|F|0-55:G>T-55:G>T	TGCAGAAGTCAGTGACCTCATGAGTGTCTGGACTCTCAACCAAGAAACTGTCTACGACATTCTCCTTGG
46756177|F|0-16:T>C-16:T>C	TGCAGGAATGTTTTTGTTTGCATTTCATGTGCGTTCATACATCTCTTTTGTGAAATACTGAAGTTGTTT
39721708|F|0-24:C>T-24:C>T	TGCAGAGAAGATCTCCTATAATTGCTTGATTCTGTTATGGATGTATTAGTTGTGGAGTTGGCCATGATG
39725943|F|0-56:A>G-56:A>G	TGCAGATTTTATTTCGATTTCTCTGCAACATATGTTGGGGTTGGAATGATTTGTCCATACATCATCAAC
39721987|F|0-13:T>A-13:T>A	TGCAGATATTTGCTTCTGATAGCTTTCTTGAGTTGACAGAATATACACGTGAAGAAATTTTGGGCCGAA
39722440|F|0-49:C>T-49:C>T	TGCAGCAGCCATTGAGACAGCAAAAGCTGACATTTGAAGAAAATGGTAACGGAAATGGGGGAATCCCAA
39725365|F|0-20:T>G-20:T>G	TGCAGTTCATCAACTGTATATATAAACTGAATAATTCAAGCATCATTAC
39724240|F|0-19:T>A-19:T>A	TGCAGTTCACAAACAAGCATATGATATACAGAATCCTTTGTTATAATTCAATCATCTTGTAATTCAAAT
39725687|F|0-36:G>A-36:G>A	TGCAGACTTTAACCCCATGATGTAGAGCTGAATGCTGGGATGCTGGTACTGGGAGTCCATCTGCAACAA
39723697|F|0-41:C>A-41:C>A	TGCAGGTTTGCGCCATTCATCCCTCTCTCTCACCTTCTCTTCTTTTCCTGTTCATCGTTTCGGCTATAT
39728787|F|0-30:T>C-30:T>C	TGCAGTTCCTCATGACTTACGAGGGGCTGCTCTCCATTCTGGCTAGTGGAAAATTTCTTCTCTATTACA
39718827|F|0-14:C>T-14:C>T	TGCAGTTGTTGGTGCGGATGATCTTGACTTGGGGGAACAAATTCACACACTTGTTATGAAATCAGGTTT
39721273|F|0-19:C>G-19:C>G	TGCAGAACCAAATCCTCGACTCGGCTGAGTCGTCCGAGTTCTGGAGGTATCAAACCGCTAAGACCACAC
39727212|F|0-22:C>A-22:C>A	TGCAGTATCCGATGCAGGAGAACTGGAGTTCAATTCTTGAGGGGACAAAAACCAAATTAC
39715603|F|0-5:C>T-5:C>T	TGCAGCAGGCTCTGGAACTGCGGATGAGCCAGCCACGATATCGGGACGATGTATCGGCTTCGGTTTTCG
46754303|F|0-7:A>G-7:A>G	TGCAGAGACACAATCTCTCACCTTGGTATCAAAAGAATGCAGCTTCACCATTTGTGGATTAGCAATCAG
39719766|F|0-5:A>T-5:A>T	TGCAGATGCCAGTCGTAGGGAATGTATTCTCTGTCCATTTTTTTAC
46768450|F|0-13:C>T-13:C>T	TGCAGACCGGACCCACGTGAACCGGCCGCCCCATCCCAAAGTCAACCTTCTCTAACCCCAGCCTTGACC
39718212|F|0-35:C>A-35:C>A	TGCAGTCTTCTGCCTTTTTGTCTTTGTTTGGGACCCAATTCGTGTCTCAATCATATACTATTTTGTCTT
39721466|F|0-62:C>T-62:C>T	TGCAGAATATCTGGTAATTCAATAGACAGCTTTCACATTGTATCACTATAATTTTCTATCTTCGACATT
39725248|F|0-23:A>G-23:A>G	TGCAGTAGTATGCACTAAACATTATATGCGACTTTTCTTTAC
46758077|F|0-16:G>A-16:G>A	TGCAGAAATTAAGCGCGAAGATGCTCTGTTCGGCGATATTTTAGGGATTTTGATTGCTGTTGCCGTTGG
39723708|F|0-59:C>T-59:C>T	TGCAGGTTTTTTTTGGATTGTCCAGTGAAAACAAATAGAGGTTACCTTTTGGTATGGCTCTGGTTGCTT
46774011|F|0-45:C>A-45:C>A	TGCAGTTAAGCTATGCTCCAAGCCCTTGCTTTATGGAAAAAAATCCAACTTCTCACTGGGCCAATCACA
39720312|F|0-11:G>A-11:G>A	TGCAGGACTTCGTATGCTTGATTAC

39724850|F|0-32:A>G-32:A>G	TGCAGATTGTTTCAACATAGTTGCAGTTTCAAAAAGTGCTGAACTTAC
39728414|F|0-22:T>C-22:T>C	TGCAGTTTCCCCATCTGATCTTTGGTTTTAGTTTCACTATTTGAATCGAATATATCCTCTATTCCTGTT
46765580|F|0-53:C>T-53:C>T	TGCAGCAGTTTGACGACTCTTCTCATTGAGGGGCGGTTCATGGGGAGTGAGCTCGTGCAGAGAAGTCCC
39714241|F|0-6:C>T-6:C>T	TGCAGACGCAATAGCTCCGATGAGGGTGGTCTGGAGGGCACTTTTGAAGGGCTTATTACCTGTGAAGTA
39723055|F|0-32:G>A-32:G>A	TGCAGCTTGGTCCTTCTAGCTTCCAAGGATGGGACAGATTGTGAGGGTAACTGATAGCTCTGGGGAGCC
39725505|F|0-48:G>C-48:G>C	TGCAGAAAATAAGCAACATATGGATATGGATTACTCCAAAGAACGTGAGGGGTTTCATGAGTAATGACT
46771683|F|0-13:G>C-13:G>C	TGCAGAAGTTGTGGATCATAAATGCAGTGACATTGTGTATCAGTATTATTTTCTGATGCATCAAATGAA
39727979|F|0-24:T>C-24:T>C	TGCAGAAGGGATGGCCAGTTTGAGTGTCGTGAGAAAGTAGTTTAC
39715313|F|0-57:T>C-57:T>C	TGCAGCAACTGCTCAGCCCAATACAGGAAACTGGGTCTACTGGCCTTCCCCTGCCGGTGCCGGGGCCTC
39726534|F|0-40:G>T-40:G>T	TGCAGGTGAGGCTACAGCATGTTTTATAGGGAGTCATTTGGTATGCAATATCTAATATCGTTGACTTCA
46768934|F|0-5:C>T-5:C>T	TGCAGCAGCCAATGAAATGGCCACTTTTCTGTTTCTCTAGAACGGAAAATTCTGTCACAAGTGGAAGGT
39728933|F|0-22:T>G-22:T>G	TGCAGCAAATGTAAGTTCTTATTGCCTCTTTATACTCAAAATCAGATACCCATTTGATCGATTAC
39714010|F|0-17:C>T-17:C>T	TGCAGAATCGCCACGAACGAAGGTTATTGTCCTCGCTCGTTCTTGGGCACTAGCTGCAATCCATGGTGG
39718724|F|0-36:A>T-36:A>T	TGCAGTTGAGACCTACGCAAAAGGCATTTCTTCCATAACGCAAAGAAAGGAGAATAGCAAGAACTGTAG
39721311|F|0-35:A>T-35:A>T	TGCAGAACTTGATGAGAGTAATGCCACAGCATCTGAGCGTACTGAAATTTCAGCAGAAAATGATATGCA
39722442|F|0-42:A>T-42:A>T	TGCAGCAGCCCAGATACCTATCCAAAGATACCAGAGTCTTCAAATTCATTCTGCTGCAAAAAAGTTAGA
46764813|F|0-49:C>T-49:C>T	TGCAGTAAAATTTCTGGCGAATACAGAGTAGAGCTACGGTACTGGAGTACGTTGTTTCTGGAAGGCTCG
39726039|F|0-18:T>C-18:T>C	TGCAGCAGAGCAGGCTCCTCCAGGAGTGGAACAACTGGGAGTGAGAGTAGAGAGATGAGAGCAGAGAAA
39721466|F|0-36:A>G-36:A>G	TGCAGAATATCTGGTAATTCAATAGACAGCTTTCACATTGTATCACTATAATTTTCTATCTTTGACATT
39717172|F|0-54:C>T-54:C>T	TGCAGGCGTATTTCTCCCGCCGGGCCAATGGGGTCTACCGGACTGACTTTCCGGCGAGGCCGCTGTCCC
39716325|F|0-41:A>G-41:A>G	TGCAGCTCATCTTGTCCAGCTTCTGGAAGCATGGCAGAATCATAAGCAACAATATTTCCATAAAAGATT
39714066|F|0-35:A>G-35:A>G	TGCAGAATTACTTCAGGAGCCATCCAATATGGAGTACCCTTCATAGATTTTGCACCTGAAATTGTAGCC
46757450|F|0-37:A>G-37:A>G	TGCAGTTAAGTTGCCTGACGAGGATGGCGATGCACGTACCGCAAGAAGCTTTCGAAGATGATGACAACT
39715551|F|0-67:T>C-67:T>C	TGCAGCAGCATTGTCGTAACCAATTTTCTGGAGACAATATGCAGGGACATATTATCAAACAAAGTACTA
39717168|F|0-53:A>G-53:A>G	TGCAGGCGGCGGCGGATTGCCGGAGATTTGAACGGAATTAGGCGGTGATGGCGACGACGGACGAGGATA
39726934|F|0-6:A>T-6:A>T	TGCAGAAAATTGAGAATAAAACTTTTACACGTGTTGCAATATTTAC
39725342|F|0-10:G>A-10:G>A	TGCAGTGTCGGTAGCATCGATTGTTAC
39726721|F|0-35:T>A-35:T>A	TGCAGTGATTCTAATGATGTGCACTTCCTCTATATTCATTTATGACATAGGAATTTGGGGCCTCTTATG
46758741|F|0-25:T>G-25:T>G	TGCAGATTAATGATATGTCAGCCCCTGTACAGCCTTGAGTAAGAGAAGCCAACTTCCTCGTACTGACAT

<table>
<tr><td>39724594|F|0-27:T>C-27:T>C</td><td>TGCAGAAAGAGTATTAGATGATTGGCTTCACTTGTCAATGGGGCAAAGCTGATTAC</td></tr>
<tr><td>39715161|F|0-30:C>T-30:C>T</td><td>TGCAGATTTACTCCTGGCGAGTCACAATAGCAAATGCCACCAGCAATGGCTTCTTCGGCTCCCATTGCC</td></tr>
<tr><td>39726805|F|0-46:C>T-46:C>T</td><td>TGCAGTTCTTTCTTGGTGACTATCGCCCGCTTATTCAGGATCCTTCCGGCAATGTAGTTGACCAGGTAG</td></tr>
<tr><td>39723490|F|0-46:A>G-46:A>G</td><td>TGCAGGGGATTATGTTCTTGATGATTGAAGTGGCAATCATTAGACCATGTACTTGCTTCAACTCCTTCA</td></tr>
<tr><td>39728610|F|0-42:G>A-42:G>A</td><td>TGCAGTATTTGCTCATATAGTAATATTCCAAAAAAAGGTGGTGTATAGTTTGAACGACACAAAAAAAAT</td></tr>
<tr><td>39716875|F|0-28:C>G-28:C>G</td><td>TGCAGGAGAAACCATATGTATTTAGCCACATTCTTATTTTTCTTATGAAAAAGAAAATAGAAAAGTAT</td></tr>
<tr><td>39727429|F|0-32:C>A-32:C>A</td><td>TGCAGAGACGACGGCTGAATCTAATGATCAAACTAAAGAATCAGGTTTCATATTCGGATGTTGATTTTT</td></tr>
<tr><td>39716750|F|0-42:T>A-42:T>A</td><td>TGCAGGAACAATAGAACAAAATTTTGCTTATTCCATGAAAAATTTGTGTTAGTTATTTCTCAACTATAG</td></tr>
<tr><td>39717618|F|0-58:C>T-58:C>T</td><td>TGCAGGTTCCAAGATGCTCCTATCCTCCTCAAACATATCCTACAAATATCATAAACCTCTAGCTCAAAC</td></tr>
<tr><td>39713964|F|0-24:A>G-24:A>G</td><td>TGCAGAATAACTTTACAATTATTTATGCCACATAAGAATAAATATATAAACAAAAAGCAGTTTTGAATT</td></tr>
<tr><td>39724021|F|0-20:A>G-20:A>G</td><td>TGCAGTCTTTCTTTGTCAACACATGAAGCTAAACGGAGGGCTTTACACCATGCTTCCTTCTCCCAGGAA</td></tr>
<tr><td>39725065|F|0-31:C>A-31:C>A</td><td>TGCAGGAATGTTGGTTTAGTCATCTTGTACACAATTAC</td></tr>
<tr><td>39720550|F|0-22:C>A-22:C>A</td><td>TGCAGGTTTGAATAGTTACAATCGTTCTCTGCTAATTAC</td></tr>
<tr><td>39717636|F|0-39:T>A-39:T>A</td><td>TGCAGGTTCTTGGAGTAAGTTGGCAAAAATAAGGACTCTTATGAGAGATTCTGGTGTGGCAAAAGCTCC</td></tr>
<tr><td>39728411|F|0-17:T>C-17:T>C</td><td>TGCAGTTGAATTTACAATAGTCATTTTGATTTTGCTTACAGAAAAAGGACAAAATGATACGAAGAATTT</td></tr>
<tr><td>39726721|F|0-66:A>C-66:A>C</td><td>TGCAGTGATTCTAATGATGTGCACTTCCTCTATATTCATTTATGACATAGGAATTTGGGGCCTCTTATG</td></tr>
<tr><td>39715803|F|0-24:T>A-24:T>A</td><td>TGCAGCCAAAACCCTCCAACCTCTTGGCAACTTCAGAGCCTATTTTCCCCAATCCAACAATCCCAATTC</td></tr>
<tr><td>39723214|F|0-55:A>G-55:A>G</td><td>TGCAGGAGGAGCAATTACACCCAGCCCTCCATCAAACGCAAGCCCGACGATAACAATGCCAGCTACAAC</td></tr>
<tr><td>39713611|F|0-20:C>T-20:C>T</td><td>TGCAGAAATGATTTTGGAGACTACGCGGAGCTTTGCTTTGAGGAGTTTGGAGATAGAGTGAAGTATTGG</td></tr>
<tr><td>39723116|F|0-36:T>C-36:T>C</td><td>TGCAGGAAAGAAAATCAGCACATCAGAGGATAAAACTGTCCATCTCATAAGTAGTTTTCTGCAAAGAAG</td></tr>
<tr><td>39717931|F|0-29:G>C-29:G>C</td><td>TGCAGTATATTCCAAGTTTAGTCCAGATTGATCAGCAAGATATTTTAGGTGCCGAATAGTTTTTTGAGA</td></tr>
<tr><td>39713407|F|0-57:T>G-57:T>G</td><td>TGCAGAAAAATGATTTTGGTTGGCCATGTGGGTTGGTCTGAATATTGTTGGAAGAGGTGAAGTTGTTTA</td></tr>
<tr><td>46756053|F|0-22:G>T-22:G>T</td><td>TGCAGCTTTAATTGCTTGAGAAGTCCTATCCTCACCCTTGGCAAAATAAGGGGATTTTTTTCCTGCACG</td></tr>
<tr><td>39724635|F|0-36:C>T-36:C>T</td><td>TGCAGAAGAAACTTCTACTGCTTGTGATGACAATCCCGATAGGTTGGAATTAC</td></tr>
<tr><td>39721884|F|0-15:G>T-15:G>T</td><td>TGCAGAGGTACTAATGCAAACATCGGCCAATCTTGGATTGTTCAACTCATTCCATTTTGCCTGAATATC</td></tr>
<tr><td>46755222|F|0-32:G>C-32:G>C</td><td>TGCAGCAGCGGACGCGTAGTTGACTCCATGGAGAGACTGTGCTCCGGAAACTTGAGAGAAGGCAGGGAC</td></tr>
<tr><td>39722676|F|0-20:G>C-20:G>C</td><td>TGCAGCCGAGGAGGCTGCATGGTGGAGGTTTTTCGTCGAGCTGATCAGTTGAAGTGCCTTGGCGACTTC</td></tr>
<tr><td>39719280|F|0-28:T>A-28:T>A</td><td>TGCAGAAAATGAATAATCTCTCTTTCCCTAAACCCATAAAATAATCGACACAAGAGATTAC</td></tr>
<tr><td>39722150|F|0-13:G>A-13:G>A</td><td>TGCAGATTCAAATGCTCAAGAAATCAAAGAAGCCTACAGAAAGTTGCAGAAGAAGTACCATCCGGACAT</td></tr>
</table>

39722183|F|0-18:C>T-18:C>T	TGCAGATTGGATACATGTCGATACATACATCACATATGTATGTATGGATCGCATCTGACTAAAGAGAGA
39717966|F|0-28:T>A-28:T>A	TGCAGTATTCAAATTTTATTTCACCTTCTTACAAATCTTCCGAATTTCTAGTCGAATTTTAC
46755385|F|0-23:T>C-23:T>C	TGCAGCATTGGTTTCTTTTTCCTTTATTTCATCTAAGTAGTCCTCTCGAGCATTTCCAGCCAGCATCAA
39717776|F|0-8:A>G-8:A>G	TGCAGTAAAGAGAAAAATGTCAAAATTGGTTCTTGCTTGCTGCAAGGTTTACATTTCTGAAAGCAGAAA
39722593|F|0-5:T>C-5:T>C	TGCAGTCAAGGTTTGGTTCCTAGCTAGCAATGTTTGATCTAGGGCAACAATGGCATGCTCCATGGTGGC
39714955|F|0-41:T>C-41:T>C	TGCAGATGACCTCGAACAAGTCCTCTGATGCGTCGTCTTCCTGGTCCCTGATTTGTTGAAACTCGGACC
39721706|F|0-30:T>C-30:T>C	TGCAGAGAAGACAGTATCAGGTGCAGATGCTGAGTATGAAAAGTTCATGGCAGATATGAAATGATTCTA
39719112|F|0-49:T>A-49:T>A	TGCAGCAGAAGTTAATATTTCATCAAGAAGCTCCATCATATGACTAGTTTTCTAAAGTCTCCAAACATT
39723249|F|0-68:C>T-68:C>T	TGCAGGATCCGACATGAAAGGCGGTAAATCGAAGTCGGAGTCGAAGAAAGCAGACGCGAAGTAAGTTTC
39721088|F|0-7:G>C-7:G>C	TGCAGTGGTAACATAACAGTAAACAGATGCCTCCTGAAGGGGCATGGAGACAAGAATGAAAGCTAAAAA
39722676|F|0-63:G>A-63:G>A	TGCAGCCGAGGAGGCTGCATCGTGGAGGTTTTCGTCGAGCTGATCAGTTGAAGTGCCTTGGCGACTTC
46756925|F|0-6:A>G-6:A>G	TGCAGTAACAAATACTCTTGCTCCTTGGTATTTAGCTATCTGAATGGCAAAAGTACCAATTCCACTTGA
39727818|F|0-64:C>T-64:C>T	TGCAGTCCATTGCAGTGTCGAGTCATTTGTGTATCGTTTGACTTTTCCAACTACAGAACTGAACCGAAA
39726738|F|0-48:T>A-48:T>A	TGCAGTGGAAAACAAAGCGTTTGAGAAAGCTTTAGCAGTGTTTGATGATGACACACCTGAGCGATGGCA
39718559|F|0-48:A>G-48:A>G	TGCAGTTAGACACGTGAAGCCATCTGAAACTGAGCCTTATCAAGAATTATCATCTAGGTTTCAAAGGCT
39727626|F|0-57:C>A-57:C>A	TGCAGCTAGCAGTTCATCAACGATGAGCCACGAAGAAAGCAGCTCCAACAACAACAACAAAAACAACAG
39726026|F|0-16:A>T-16:A>T	TGCAGCACTTCAGCTGAGTAATTTTTTTCATATGTAAGAAACCCACCATCATTCTTAGCCTTCCCATCA
39721050|F|0-59:A>G-59:A>G	TGCAGCACTCGCTCCACCGTCTGCTCCGCCGGATGTGGGCCCCCCGGATACGCCGGTCCACCCAACGGC
39722421|F|0-5:T>C-5:T>C	TGCAGTTGCAACTGAAGAGAAAAAAGCAGAGGAAAGCAAGGAGGAGCAGCAGTAGAGAATCAGGAGAGG
39722183|F|0-28:A>G-28:A>G	TGCAGATTGGATACATGTCGATACATACATCACATATGTATGTATGGATCGCATCTGACTAAAGAGAGA
39728912|F|0-22:G>T-22:G>T	TGCAGGTGGCGGTGGAAAGAGTGGATAGAGGCGCATTGGACGCCATTAGAGAGATTAC
39716573|F|0-24:C>T-24:C>T	TGCAGCTTCCTGCAAGTTACTAAACCCCCATCATGAACTATATAGGGAGTAATCTATCAGCCGGTAAAA
39721737|F|0-51:A>G-51:A>G	TGCAGAGACTTCAGCTTCAACAAAATTACCGGACCAATACTTGGTCGCTTTAGGGAGTTGACAAAATTG
46768532|F|0-43:A>G-43:A>G	TGCAGAGAGGGGAAGTGCGAGTGAAAGAGCGAAATGAAAAAGGATATTATATAAATGGAATTTGTTGGC
39716799|F|0-58:G>A-58:G>A	TGCAGGAATAAGAAAATGTGAATATAGTTTCAAAAGAGATCGTTATTATCACCTGGGTGGATCTCAGGA
46764263|F|0-56:G>A-56:G>A	TGCAGGAACTGAAATATTGATGAAATTATATTACTGCCTTTATAATATAAAAAAGGGAAAAAAAATTGT
39727685|F|0-57:A>G-57:A>G	TGCAGGAGAAGTTGTCTCGCCTCGAGCACGCGAACCAGGAAGCAGAATCTAGGTTGAAGATTGCGCTTG
39721104|F|0-17:T>C-17:T>C	TGCAGAAAAAGAACAGATACTGAAACTTATGGAGAATCACAAGGTAATAAACCAAAAGTTGGGTCAATC
39723032|F|0-50:A>G-50:A>G	TGCAGCTTCGTCTTCTTCTTCGATCTTAGGAGCCAAGTAGTATCTGATATAACCCATATCTTCAATTCT

39727729|F|0-7:G>C-7:G>C	TGCAGGGGGAAAAATCTCCATTATTAGCCATAGAAACAATTGGGAAACTTGATGTCTCTTCATTGAAAT
39719827|F|0-19:A>T-19:A>T	TGCAGATTTTGATTAGAAAATTATTCCCATGGAAAGGCCAAGCCATGAAGTTTCATTAC
39719530|F|0-11:C>A-11:C>A	TGCAGACGATGCGCTGGAAGAAGACACCATTAC
46771773|F|0-39:T>C-39:T>C	TGCAGAGGAAAAGGAAGCAAGAGAGCACAAGGTTTACATGAAAGCAGTGATATGAAGACAGAGTTAGA
39720105|F|0-7:G>T-7:G>T	TGCAGCGGGAGGTTTGAATTTTTAGTTTAC
46755030|F|0-30:C>T-30:C>T	TGCAGCAACGACTTGAACATTATGGACTCGCTTATCCAAACTCTTCTATGTAGAGTTAGGCAGATCTAA
39725551|F|0-29:T>C-29:T>C	TGCAGAAATTGACCCAAAGTTTTGTGGTGTTGTTTGTTTCATTTGACCCTTTGACTAAATCTCCAAATT
46772913|F|0-32:A>T-32:A>T	TGCAGATTTTTGATAAAAAGAAAAAAGATCATACATATATATTTATTAC
39724679|F|0-15:T>C-15:T>C	TGCAGAATTTACGATTGAATTTGCTCGTTAC
39721074|F|0-46:T>A-46:T>A	TGCAGGTGTACCCAAGGAATCTGTCAAAGACAGTGTCCATGCAGTATAAAGTTGGTAAAACGGAAAGTG
46755133|F|0-68:A>C-68:A>C	TGCAGCACGACACAAGTTTATACTATGTGCATAACCAAGAGTTCTATGAACGATGAATTCAGTGAAAAA
39727341|F|0-68:C>T-68:C>T	TGCAGAAAGCTCCAACAATGGCGAGAGAGAAGAAGAGAGTCTTATAGAGAAGCTGACGTCATAAAGCGC
46767191|F|0-17:G>A-17:G>A	TGCAGGAGAAAATTGTCGGGTGTTATCGGTTAC
39725450|F|0-6:C>T-6:C>T	TGCAGACAAGTGATGGTTATGCGTGGAAATGTTGCTTTTGAGGAGGGATTAGTCTCATTCAGGAGGTTT
39721526|F|0-29:A>T-29:A>T	TGCAGAATTGAAGAGAAAAGTGAGAGGAAATTGCAAAATGGCGAAGACCCAGATAAGTAAATTGCGGTT
39723517|F|0-33:G>A-33:G>A	TGCAGGGTCAGTAGCTTGAATCTCATAGTGGATGCCATCGAGCTCGTGCCGAAAGTTGTCTTTAGATGT
39726448|F|0-47:G>A-47:G>A	TGCAGGCTCGCATTGCAGCCGCCGTGATATCTTGGCTGTGGCAAAAGGGAATCCACTCGTTAC
39723438|F|0-68:T>C-68:T>C	TGCAGGGAATCAAACCACAAAGCACCAAGAAAACTCAAAAGGCAGTGATGGAGCTCAATCAACCCACCT
39723615|F|0-38:A>G-38:A>G	TGCAGGTGACAATATCCCAGATGAATGAAAAGGATTACACATCGCATTCTGTTCATGTATTTCATGTTG
46763247|F|0-37:G>A-37:G>A	TGCAGATGTTTCAACAACGACGACCTCGCGAATTTAGGGTTTCTATGAATAATGTCTCTGTTCTCAGCC
39721818|F|0-67:A>T-67:A>T	TGCAGAGCTCTCAGCAGAGATACAGAGAAAGAGCGGGAGAAGCTGAGCTTTGGACAGAGAGAGAGAGAG
39724733|F|0-19:G>T-19:G>T	TGCAGAGAGGCGGCACCAAGGGGCCAGATTGGTTGTAATTAC
39726040|F|0-15:A>G-15:A>G	TGCAGCAGAGCGCCAAAAATACAAATACAATGTTGACAAGGAGATGACACTATTGAAATTTGTGGATCT
39722706|F|0-6:T>C-6:T>C	TGCAGCTGTGTTTGAAAGAGGTGACTCTGTTGATGGACTTATCAATGCAAGGCCAACCTTCTAGTAACC
39717323|F|0-29:C>T-29:C>T	TGCAGGGCTTATGGCATAATATAGTCCGGCAACCTGAGCACTATAAGCTGCAAGACAAGAAAAGTTTAG
39722985|F|0-34:G>C-34:G>C	TGCAGCTGGTGAGCTCCGGCGGCCATATTGTGGCGTCGAGGACTCTCTACGGCGGCACTCATGCGTTGC
39716753|F|0-15:A>G-15:A>G	TGCAGGAACAGTTGGAAGACAAGACGAAGATAGTGTTTCCGATGACGACGAGCAACGACACGGCGGTGC
39719801|F|0-5:A>G-5:A>G	TGCAGATTCTTCAGAGCCACCGATTTAC
39725286|F|0-18:C>G-18:C>G	TGCAGTCTCCCAGGTCTTCACAAATCAACCGCTTCTCTTTTAC

<table>
<tr><td>39727507|F|0-7:T>C-7:T>C</td><td>TGCAGATTTGTTTTGGGCAATGTAGTTCGAATGGGGCTACCAAAATAAACCAACCAATCAGTAATCTTA</td></tr>
<tr><td>39729148|F|0-30:C>A-30:C>A</td><td>TGCAGCAGCTGATTCACGTCTTCAGCAGATCAAAAACTCCATTCGCTCCATGTAAGTAATGAATTACAG</td></tr>
<tr><td>39729485|F|0-49:T>C-49:T>C</td><td>TGCAGGTCAGAAGTACAAAAGCTGAACTTTTTAGAGCTTGTGTTATTTATCTGTATCTGATTAC</td></tr>
<tr><td>39721918|F|0-53:A>C-53:A>C</td><td>TGCAGAGTGAGGTCATTGCCTCTGCTTCTTTGGTGTGGAGTGATGATGATGGTATGCACGAAGTGAGAA</td></tr>
<tr><td>39727056|F|0-6:A>T-6:A>T</td><td>TGCAGCAAACACGTATATTCAGGTTTGTCTTTTAGCTTTTTTAC</td></tr>
<tr><td>39724431|F|0-60:T>C-60:T>C</td><td>TGCAGTTTCTTATTTTTGATTGAATAATTGATTCTTGAACATGGGCAAATGAGAGTCAAATTGGAAACT</td></tr>
<tr><td>39719314|F|0-20:C>T-20:C>T</td><td>TGCAGAAAGCAATAAAAAAACGAGTTAC</td></tr>
<tr><td>46760929|F|0-24:C>T-24:C>T</td><td>TGCAGCTTCTTTGCTGATCGTTGACAAAAGTTTTGCAGCAGTGTTAATTAC</td></tr>
<tr><td>39719603|F|0-27:C>T-27:C>T</td><td>TGCAGAGCAGTTTGAACACAAGAATGACAGCAGGGTTAC</td></tr>
<tr><td>39719525|F|0-15:G>A-15:G>A</td><td>TGCAGACCTACTACAGCCACCTAAACTTAC</td></tr>
<tr><td>39717421|F|0-45:G>T-45:G>T</td><td>TGCAGGTAAAATCGCCGGTCGCCATTGAATTTCCGCCAAATCATCGTCCTCACCTTCCTAAATCCCAAC</td></tr>
<tr><td>39715732|F|0-35:G>T-35:G>T</td><td>TGCAGCATGCAAATACAGCCGGCAGAACTTCTCCAGAATTCCATTACTATTATATCCTTGATCACCTGA</td></tr>
<tr><td>39727400|F|0-62:C>A-62:C>A</td><td>TGCAGACCAGGCACCCCCTCGAGTTTCACATTATTATTATATATCATATGCTCCAAGATGAACGACTTT</td></tr>
<tr><td>46754847|F|0-10:A>G-10:A>G</td><td>TGCAGATTATATACACCTGGAGGTCGATGTCCCACTAAGTGACAGCAGGATTTCCTGCCACAAATAATG</td></tr>
<tr><td>39720050|F|0-43:A>G-43:A>G</td><td>TGCAGCCCTATGAACTAACCTGCCTGATCTGGCTGGATTGCGCATAACAGGTCCTTTTAC</td></tr>
<tr><td>39725600|F|0-59:A>G-59:A>G</td><td>TGCAGAAGGAGGACTGTTCGGGTAGCGTACATCTTCGTCGACGTCGAGTTTATTCTACTAATGTATGGC</td></tr>
<tr><td>46757447|F|0-66:G>A-66:G>A</td><td>TGCAGTTAACTTTTCCTTTCCTGGATGGTCCTGCATATATATGTTCTGAATTGTTCATGTTTGACTGGT</td></tr>
<tr><td>46755617|F|0-30:A>T-30:A>T</td><td>TGCAGCCTGCTTCTATAACGCAAACGAGTCAGAAATCACAACCCAACTCCAAATCACAGCTCCAATGAA</td></tr>
<tr><td>39714280|F|0-34:T>G-34:T>G</td><td>TGCAGACTCCTCAAAAGGACAATAATAAAAAGGATTGATGGTTGTTTGTGCGTAACTAAAGGGGAAGAA</td></tr>
<tr><td>39721702|F|0-37:T>G-37:T>G</td><td>TGCAGAGAACATGGGAGCGAGCAAAAATGGCTACTACTTTGGATTGTGTTGGTAAGCATATGTGCTTTG</td></tr>
<tr><td>39723641|F|0-29:C>G-29:C>G</td><td>TGCAGGTGGTGTGGAAAATCAATCCATAGCTTAGCCTTCTTTGTCTGTCATGTTGTTGCTGCTGAGGAA</td></tr>
<tr><td>39714164|F|0-45:A>G-45:A>G</td><td>TGCAGACAGTTCAAGATGTTATCAGACCTCAGGTAGCTACACCAAAACATCGGACTAATTGTATGGCAT</td></tr>
<tr><td>39718623|F|0-61:T>G-61:T>G</td><td>TGCAGTTCAGTTGTGGGTGGATGAGAAGCAATACTACAACTATGGCACCAATACTTGTGCTTCTGGTAA</td></tr>
<tr><td>39715299|F|0-55:C>A-55:C>A</td><td>TGCAGCAACCGGTAATTCTATCTTCCTCTCTGATATATTGTTGCCAATGATCTGACACTGTTCGTTTTT</td></tr>
<tr><td>39719742|F|0-22:A>G-22:A>G</td><td>TGCAGATGAACCCACCTCTCAAACTTGACTGGCTCACTATTAC</td></tr>
<tr><td>39722951|F|0-67:C>T-67:C>T</td><td>TGCAGCTGCAAATATTTCATTAGCTAGTGTACAACCTTTGGTAGCCATTGCTCAAGCTGTCAACAACCA</td></tr>
<tr><td>39717705|F|0-25:T>A-25:T>A</td><td>TGCAGTAAAACTGAAAAAAAAAAAATAGTAATGAACAGGCAAATTTTCATGCCAAACAACTCCAAGTAT</td></tr>
<tr><td>39718341|F|0-28:C>G-28:C>G</td><td>TGCAGTGCATCTCCGGTGATGCGCCAAACCATAGACTTTGAGGCGGCGGCTGCGAGATGCAAGGCGCCG</td></tr>
<tr><td>46755871|F|0-67:T>A-67:T>A</td><td>TGCAGCTGAAAACATCACATTTGGTTTCCATCAAGGTTCAAACAATAATATTTTATTTTATTTTTTTA</td></tr>
</table>

<table>
<tr><td>39718664|F|0-49:T>C-49:T>C</td><td>TGCAGTTCTAAAATCATCTGCATGCTGCAATTTTCATAAAATTTTGCGCTCTTCTTTAGCTGAATAAGA</td></tr>
<tr><td>39713496|F|0-19:T>A-19:T>A</td><td>TGCAGAAACATGAGAGGGGTGAGTTTTTGGGATCAAACATATATGGGGTTGTTGTTCTGATCTGTTTGG</td></tr>
<tr><td>46761060|F|0-29:G>A-29:G>A</td><td>TGCAGTCTATCATATAAATAGTTGTGCATGCATATAAGCTGGTTAATTAC</td></tr>
<tr><td>39718400|F|0-57:G>A-57:G>A</td><td>TGCAGTGGATATTGAGGAGATCTACTTTGATTTTAGAAAACAACGGTTTATTTATTCGAAGGAGAAGGA</td></tr>
<tr><td>39720112|F|0-24:G>T-24:G>T</td><td>TGCAGCGTACAACCCAGTAAACTAGTGATCATTGATTAC</td></tr>
<tr><td>39727227|F|0-5:T>C-5:T>C</td><td>TGCAGTGAAACGGTTCGTTTTATATTTAC</td></tr>
<tr><td>39718770|F|0-54:C>T-54:C>T</td><td>TGCAGTTGCTTGAACTTTCTGACCGACCTGGATTCTGGTATTTTTCGATCCAAACGCACCGTTTCCGAC</td></tr>
<tr><td>39715679|F|0-26:G>A-26:G>A</td><td>TGCAGCATATTGCATATAATCCTCACGATGTATATATTTTCCTTTTGCCCCTCTTTGACTTCCAAGTTG</td></tr>
<tr><td>39714385|F|0-42:A>G-42:A>G</td><td>TGCAGAGACACATAAAGCCTGTAGAAAGTAGTAGCTAGCATCAAATTACAAATGGAAGCCCTATTATTT</td></tr>
<tr><td>39723139|F|0-19:T>C-19:T>C</td><td>TGCAGGAACTCCAATACCATCAGGATGCTGACCTCCAAATTGCATAACTTTACATATGTTCAAGTTCAA</td></tr>
<tr><td>39717862|F|0-26:C>T-26:C>T</td><td>TGCAGTAGAACCATGCCCGGTTCCCTCTACCACGAGGAACAAGACGCCAAAACCTTCGCCGAATGGGTA</td></tr>
<tr><td>39714579|F|0-66:G>A-66:G>A</td><td>TGCAGAGGCGCGTTCCCCATATCCAGCATGAAGCTGCAAGAAAGACAGAGTTCCATCACATCCTACGAA</td></tr>
<tr><td>39714027|F|0-26:G>A-26:G>A</td><td>TGCAGAATGAAAGTGCAAAAGATTGCGAATGACTTCAAACAATGACAGAAGCAAGTCCAGCTCGAGAAA</td></tr>
<tr><td>39727227|F|0-21:A>G-21:A>G</td><td>TGCAGTGAAACGGTTCGTTTTATATTTAC</td></tr>
<tr><td>39715874|F|0-26:G>A-26:G>A</td><td>TGCAGCCAGGAAGTTTGAAAAGGAAGGAGGAATTTTAGAGGAAATTCTTGAGCGTGCAAAAAGTAATCA</td></tr>
<tr><td>39716236|F|0-36:T>A-36:T>A</td><td>TGCAGCTAACTTAGATATATGGGATCATGGAGCTAATAGATCATAGGAATCTGTAACCATGGCCCTGAA</td></tr>
<tr><td>39717523|F|0-55:A>G-55:A>G</td><td>TGCAGGTGACAATATACACTTAGGTCATTTGGGTATATGTGTGTGTAAAGGTGACAATATTAC</td></tr>
<tr><td>39719956|F|0-11:T>A-11:T>A</td><td>TGCAGCAGCCTTGTCATTATTCGGAGGTGGAGCGTTTAC</td></tr>
<tr><td>39713881|F|0-35:T>C-35:T>C</td><td>TGCAGAAGGCCAATTTTCACTGATGATTGGAGTGGTAGAAAATTCCCTTATCCATTTGGACAAGAGTGG</td></tr>
<tr><td>39725848|F|0-51:T>G-51:T>G</td><td>TGCAGATCCCTCTGGACATGAACTTCCCCTTGACAGAGAAGAGGCACAGGATAGTTTGGAAATTACTGC</td></tr>
<tr><td>39716534|F|0-11:A>C-11:A>C</td><td>TGCAGCTTATCAGCCGTATTCAGACTCTTATGGGTTACAGACCATAAGAAGAATTTGCACTTCTTAGGG</td></tr>
<tr><td>39726804|F|0-13:C>T-13:C>T</td><td>TGCAGTTCTTCCTCCGGTCTCCCACTTGGAACCAAACCCACCTCCATCATTCCACCCGTAGGATCGCCA</td></tr>
<tr><td>39724064|F|0-15:T>C-15:T>C</td><td>TGCAGTGATTTCCCCTATGTGTATTTGGTTGTCTGGTTGGTGAATGAATCGATTCATAATCAATTATGA</td></tr>
<tr><td>39721788|F|0-33:C>A-33:C>A</td><td>TGCAGAGCAGAGTCGCAAGAGAATCCTGGAGAGCACATGGCGCTTCCATCCAACCGGACCAGATCGGCG</td></tr>
<tr><td>39718369|F|0-62:T>C-62:T>C</td><td>TGCAGTGCTCCACAGAAAGCAGAGCAGCCAGAATCCTTGAAGAAGAAGAAGAAGAAGAAGAGTAACATT</td></tr>
<tr><td>39717247|F|0-15:G>A-15:G>A</td><td>TGCAGGGAACCAATAGTGTTTACTGAGTCAAGCTTTTGATTCTTTTTGAAATTCAGGTTCTTGATTGG</td></tr>
<tr><td>39725882|F|0-30:G>A-30:G>A</td><td>TGCAGATGGGGTATATTCACTCTGTTTTATGCACTATTTTTTGTCCTTCCAAAATAAAACATATTTATT</td></tr>
<tr><td>39721309|F|0-16:C>T-16:C>T</td><td>TGCAGAACTGTTCACTCTGGTTTGAACAGTAAAGTTCAAGGAGAAATATCACATAAATCAGATGTGCCG</td></tr>
<tr><td>39719367|F|0-19:T>C-19:T>C</td><td>TGCAGAACCTTTTTCATCCTTTGTTTAC</td></tr>
</table>

39726804|F|0-44:C>T-44:C>T	TGCAGTTCTTCCTCCGGTCTCCCACTTGGAACCAAACCCACCTCCATCATTCCACCCGTAGGATCGCCA
39720006|F|0-24:T>C-24:T>C	TGCAGCATTTATCCTTTGATTGGTTTCATCTAAATGGGAAGTCTTAC
39725985|F|0-12:C>T-12:C>T	TGCAGCAACTCTCTTCTTTATTCATCTCTTTTGTACTTGTAAATGATAAATCTTCACAAGATTCCTTCC
39721105|F|0-21:T>C-21:T>C	TGCAGAAAAAGCAGTCAAAGCTGCATATTGAGTTTACTTATCAATTCTTTTTCATGGTTGATCACTTGT
39713899|F|0-38:T>C-38:T>C	TGCAGAAGGTAAGTGTGTTTGTTGGTATGGGGGATAAGTTGGATTCCCAACACCAACTAAATTTGGAAC
39719582|F|0-29:T>C-29:T>C	TGCAGAGACTGTTGATGAAATGCTCAAGGTGTTTAC
39720620|F|0-18:T>A-18:T>A	TGCAGTATACTCCTTCCATAAAACTTAC
39716714|F|0-24:A>G-24:A>G	TGCAGGAAAATATCTTCGTTATCAAAAATGGAAATAAAGGAACTTACATCAAGCAATTTACCTTGAACA
39722103|F|0-68:G>T-68:G>T	TGCAGATGGAAGTTCAGAAGCGACTGCATGAACAATTAGAGGCACGTTCTAGTTATTCTCCCTTTTTTG
46760996|F|0-18:T>C-18:T>C	TGCAGGTAGACTTAAAATTGCCTCGTTAC
39726557|F|0-48:G>A-48:G>A	TGCAGGTTGGCTTGCTACAGGCTTCACAGGTAAGCTGGAAGCACTAACGAAAGTCCGAGGCCCTTCCTG
39719258|F|0-7:A>T-7:A>T	TGCAGAAAAAAAGCAATAGTTATTGAAACTCATTAC
39714888|F|0-24:A>G-24:A>G	TGCAGATCCCTTCTTTTTTTTGATACAACAGGGTAAGTTAGAAGGAAGTTTGGTCTACAATATGGTGAC
39713968|F|0-42:A>T-42:A>T	TGCAGAATACATCTCTGCTCTGTTGACATAAAACCTTAGATGAACAATGTATAGGATGTTGCTGACTGA
39728528|F|0-14:T>C-14:T>C	TGCAGACTATATATTCCAAACTCCAAATGCTCCTTCTGCACCATTTTCCTTCTAACTCCATTTTTGTGT
39714727|F|0-17:C>G-17:C>G	TGCAGATAAAAGGAAATCTAACAATGCAACTCCGAAACAAGTTGCTGACAAATATGTAATGGAGTCTGC
39724565|F|0-29:C>T-29:C>T	TGCAGAAAAACGAGGTGGAAATTAGTGCACTTTAC
39725002|F|0-16:C>T-16:C>T	TGCAGCTATTTTCTTTCTATTTCTCTAAATATTTTCTAACTTGTTTATTAC
39716476|F|0-61:T>G-61:T>G	TGCAGCTGGGGTTGAATATCACCAACCCCAATAAATTTCACATAAAAAAAATTACCAACTTTATGTACC
39725330|F|0-7:G>A-7:G>A	TGCAGTGGAATTGCATGCTTTCAAGTTTGTACATTGATTTAC
39720463|F|0-16:G>A-16:G>A	TGCAGGGTTCAAGTAGGTGAATTCGTTAC
39718012|F|0-29:T>C-29:T>C	TGCAGTCAACCTTGCAATGCCAACCTCTTTGAAGTCATAGGTTACCCTTCAACTTCTTGTAATTACTCT
39718357|F|0-27:A>G-27:A>G	TGCAGTGCCTTTGCATACTGGCAAGCAAATTTGAATGGCTTCTGTAAGTTATATGCCTTTTTTGCGAAA
39714367|F|0-34:T>A-34:T>A	TGCAGAGAATAGGAAACAAAAGATTCGAAGAATTTCGCGTGATTGTATGTAACTACTTCCATGACAAAA
39725126|F|0-44:A>G-44:A>G	TGCAGGGAAAAGGAAGAAGAGCATTTGAAGGGCAAATTGGACCAAATTAC
39721022|F|0-64:G>A-64:G>A	TGCAGTAAGGGGCGAAGCATCCATTTTAGCGAACGAAGGGGAGCAGTCTTGCACTAACGGCGACGACAA
39713637|F|0-53:G>A-53:G>A	TGCAGAAATTTATTCCAATATTGAGATCTCAGAATTAGAATCAGATCAAGGAGGTGGTATGCTGATTTA
39715756|F|0-38:A>G-38:A>G	TGCAGCATTATCGATAAGGAAGAAGAGAGAAAGAGAGAACTCACCTCCTTGAAGATTTCACTGTAAGAA
39719523|F|0-10:C>A-10:C>A	TGCAGACCTACACCCTAAAATCAAATCAACCAATTTTAC

<table>
<tr><td>39721499|F|0-42:C>T-42:C>T</td><td>TGCAGAATGATTATCAAGTGTCACAACTTGAATTGAGGAACACCAATAGCAAATGCTGATGATACAAAC</td></tr>
<tr><td>39719711|F|0-19:A>C-19:A>C</td><td>TGCAGATCAAGAGACCAAGAGAAATTGAAGAAATTTTAC</td></tr>
<tr><td>39719726|F|0-21:A>T-21:A>T</td><td>TGCAGATCCAAAACTTACGCAACCCGGCTCACAGTACACATGGAAGTCTTAC</td></tr>
<tr><td>39728232|F|0-9:G>T-9:G>T</td><td>TGCAGATTCGATTCTGGGCATGAATGTGCAACCTAAAGACTCGCAAGTTTTTTCCTCTACTTCCCACC</td></tr>
<tr><td>39717178|F|0-52:A>C-52:A>C</td><td>TGCAGGCTAAGAGCATCTGTGTCCCATCTTCTCTCGGCATTTCACTACCAATAGGATCACCATTTACAG</td></tr>
<tr><td>39718324|F|0-22:T>C-22:T>C</td><td>TGCAGTGATTGTGTATGAGTCCTGAGGCCATTCTTCAAATATGTTTCTGTCTTTGTTCTGAACTAATGC</td></tr>
<tr><td>39721771|F|0-5:A>C-5:A>C</td><td>TGCAGAGATTTAGTTATGAGTGAATAATGAGAGTTCCATCCAGTGATTGTTCTTCCATTGATTCAAAAT</td></tr>
<tr><td>46754353|F|0-34:T>C-34:T>C</td><td>TGCAGAGATGCGCAAAGCTGAAATCTAAGGAAGCTGTGAATCTGATTGAGAATCATAGTTGTGCATTGA</td></tr>
<tr><td>39714333|F|0-41:G>T-41:G>T</td><td>TGCAGAGAAAGAACAATACAATATCACATATGTGTAAGCAAGAGATTATGAAAAATAGGGAAAACTTCC</td></tr>
<tr><td>39716974|F|0-39:G>A-39:G>A</td><td>TGCAGGATCATGTTGGATACCTCCACCAAAATAGTATTCGTTGCCATACACAACAATTCCTGTGTGCCT</td></tr>
<tr><td>39717942|F|0-66:T>C-66:T>C</td><td>TGCAGTATCTCAACTACGACAGGACATGATTGGGTCTGTAATATTTGCCTTGAGTAAAAGAAGATGTAA</td></tr>
<tr><td>39723701|F|0-40:T>C-40:T>C</td><td>TGCAGGTTTTCACCATCGATGGTGGGATGGTTATGTAGGTTGATTATGGATAAAATTTGCACGACTAAG</td></tr>
<tr><td>39724634|F|0-16:T>G-16:T>G</td><td>TGCAGAAGAAAAATAATGAGAAAAAGTTATGCTTTGCACCAGAAAATGAATTAC</td></tr>
<tr><td>39726586|F|0-10:C>T-10:C>T</td><td>TGCAGTACAACTTGTGCTAAATGTCAACGAGGGGTCAAACACTATAAATAATATGCTAAGTTGGTGGGA</td></tr>
<tr><td>39715662|F|0-48:A>G-48:A>G</td><td>TGCAGCATAGAAGATGGAAAATTTGGGGCAATTTTGAAGCAATGCTCTAGTCCTTTCAAATAATTAGAC</td></tr>
<tr><td>39728828|F|0-9:C>T-9:C>T</td><td>TGCAGAAACCAAGTACAAAATTCCTCTGATATAGACACCAACCGGAAGAAAAAAATCTAAGATTTACAG</td></tr>
<tr><td>39719555|F|0-22:T>G-22:T>G</td><td>TGCAGACTTTGCAGTTTTGAAGTATCAATCTATGATATTAC</td></tr>
<tr><td>39715078|F|0-29:C>T-29:C>T</td><td>TGCAGATTATAAACATTCTGACAATAAAACACTCTAAAACCATGGGCTTTATTGTTGGACTAATTTTCC</td></tr>
<tr><td>39715855|F|0-32:G>A-32:G>A</td><td>TGCAGCCACTAGGGTTTGTAAAAAGTGTAGTGGCTTATATGGTGATGGACGATCTCACTGTGAACCCCA</td></tr>
<tr><td>39719867|F|0-30:C>A-30:C>A</td><td>TGCAGCAACTAGTGAACTAAACATAGAAACCATCAAATTAC</td></tr>
<tr><td>39718706|F|0-59:T>A-59:T>A</td><td>TGCAGTTGAAATCAGCCAATCACCAAAATTCTATTTCCACTCCAAACAGCAATACGATCTAGTAACAAT</td></tr>
<tr><td>39725103|F|0-14:A>G-14:A>G</td><td>TGCAGGCATCAAAGAAAACAAAATGAATTAC</td></tr>
<tr><td>39715219|F|0-14:A>G-14:A>G</td><td>TGCAGCAAAAAAGGAGTCCCAACCATTGACAAGGAAAAAAAATAGCAATAAAAAGGCTGCTTCCGACAA</td></tr>
<tr><td>39719800|F|0-36:A>G-36:A>G</td><td>TGCAGATTCTGGATGAATCAATTCACAAGATAAAACATGAAGATTAC</td></tr>
<tr><td>39725143|F|0-21:G>A-21:G>A</td><td>TGCAGGGGCTGCTAGTGGCACGATTAC</td></tr>
<tr><td>39723485|F|0-56:A>C-56:A>C</td><td>TGCAGGGGAATTTGGCTTTGGTGACTGCTTTGTTTGTGTTTAGTTTTGACTGCGTTAGAAACATCCATG</td></tr>
<tr><td>39710729|F|0-65:T>A-65:T>A</td><td>TGCAGTTGAATGTAATTCTAACTTTGACATTGAAATCTTTTTGCTCTTGACAAATATGTTTCTCTTTCT</td></tr>
<tr><td>39722082|F|0-51:T>G-51:T>G</td><td>TGCAGATGCCAATGGTGGCAGTCAAGTCTGTTATGTAACTCCAACTCCACCTAGAAACACAACATCTTA</td></tr>
<tr><td>39716729|F|0-51:A>G-51:A>G</td><td>TGCAGGAAAGATCCTCAAGCCAAAAGAAGCTGCTACCAACACCAGTTCTGGAAGAAACATGGTTGCAGA</td></tr>
</table>

39721619|F|0-68:T>C-68:T>C	TGCAGACCTATCCCATGCAAAGAACCTCACATGGCTAGAATGATTGACGTTCCTATTCCATGGATTTTT
39718475|F|0-17:G>A-17:G>A	TGCAGTGTACAACCACTGCTACCTGGTTTTTGCTACTTGGTTTTGCAACAAGTGGACCACTTTACTAAA
39714403|F|0-5:A>G-5:A>G	TGCAGAGAGAAACCGCAAGTCTCTGTTGAATTGGATGCTGCCACTCTGGCCTGTCCCGCATGTGATTTG
39720366|F|0-28:G>T-28:G>T	TGCAGGATTGAAATTTGAAATAATAAACGAGTGTTTCCTTAC
39721523|F|0-66:T>G-66:T>G	TGCAGAATTCAGTTGGTTGGTCTGTCTGTTACTTCAAGTTCTTGTTTCTTGATGAATTGGAGCTGTTGC
39729336|F|0-12:T>A-12:T>A	TGCAGGGCCTTGTAAGGCAACACCTCTGAAAAGCTGAAAATCAAACTCCTAACGGCTCTTTAC
39719887|F|0-30:A>C-30:A>C	TGCAGCAATAAATGCTCCCATTTGTGCACAAAATATACCAAGAGGGAGCCCAAATTAC
39723076|F|0-26:T>C-26:T>C	TGCAGCTTTCTTTCTGACTTCAGCTGTGGCACGAGCAGCTCTCTCATCTACACCTCTACCACGAGGAAG
39720304|F|0-35:C>T-35:C>T	TGCAGGACCAAATCATAGTTTTCATAATATGAAGACATGCAATTAC
39727080|F|0-18:A>C-18:A>C	TGCAGCATGATCATCCTAAAAACAAAAGGCTTTAC
39722005|F|0-46:G>A-46:G>A	TGCAGATCATAGACAAAAGAAGAAAGATTATGAAGACAGGTGGGGAGAAAAAAGGATCCTTTGAAAACC
39724650|F|0-30:T>C-30:T>C	TGCAGAAGGGAAAGCTTTGCTTCAACCTTTTATATTGCCTTCCCTTGAATTAC
39728199|F|0-67:C>A-67:C>A	TGCAGAGGAGAAACTACAGAGTAGAAAATGGGCCAGCAAAGAGACGAACGTGTTGGGAAAAGAAGGCCC
39715107|F|0-37:G>A-37:G>A	TGCAGATTCGACCAAAGGTGGGCGTAGCGGCACACTAGCGTTTGATGCCATTCGGGATAGCTTGCGGAA
39720104|F|0-5:T>C-5:T>C	TGCAGTGGCGAATCTAAGGTGGGTTTTTGAATGATGAGAGTTAC
39714465|F|0-47:C>G-47:C>G	TGCAGAGATGTTGGCATAGGCCATTGATGGCAACATTTATGAGCACACGTAGGAAATTTGAAACTAGAG
39724820|F|0-45:A>G-45:A>G	TGCAGATGAAAGAAAAAAGAAAAACTTTGAAGCCCAAAGGGAAATATTAC
39722327|F|0-56:C>G-56:C>G	TGCAGCAATTGTTGGATGTGTGAATGCCATTCAATTTAGAATTAGCTCTCGTCTAACTGCTAATGCTTC
39724751|F|0-41:C>A-41:C>A	TGCAGAGGAAAAGACCCAGTACCTTATTTGAATATACGCATCAAAATATTAC
39718914|F|0-8:G>C-8:G>C	TGCAGTTTGCCCATGGATGAAGCAACAACATTGTTTGTTTGTTACTCATCCATCTCTTGGATTACTTTT
39726764|F|0-57:C>T-57:C>T	TGCAGTGTTCTTTGGTGTGCATGCGTTTGGTGAAGGGAAGGAGAAGGACAGATCTAACGCTTTTGCAGC
39729260|F|0-8:G>C-8:G>C	TGCAGCTCGCCCAGTCTGCACAAGGTCTCTTTTATGTTCAGTATTCGGTGGCGTAAAGTGCTATTACAG
39722569|F|0-38:A>G-38:A>G	TGCAGCATTTGACAACAATGGTAAGGGACAAGCTAATAAGAAATCCACTTCTCCTGGTTCTTCTGTAGT
39725305|F|0-21:G>A-21:G>A	TGCAGTGATCCTAATGGTAATGAAACCATTGAAAAGTTATGACAATATGAATTAC
39718688|F|0-66:C>G-66:C>G	TGCAGTTCTTCCGCCATTTCTATGTCTCTCTGCAAAAAGCTAGAAACAGACTCTCACCATCTTTTACGA
39718232|F|0-61:C>A-61:C>A	TGCAGTGAAACTTCTAGATGGGAAAAAGAAATTATTGCACAAAGAAAGAATTAGAAATCGACTGTTGTA
39725214|F|0-7:C>A-7:C>A	TGCAGTACAAGCAACCAACAAACATGTTAC
39719553|F|0-5:T>A-5:T>A	TGCAGTCTTGTGGTACATTCAAAACTTTGTTTATTAC
39725144|F|0-35:T>A-35:T>A	TGCAGGGGGAAGGACCCTGATGCCGAGGCACACATTCCATGGTGGTAGGTGTTTTTAC

<table>
<tr><td>39720178|F|0-7:T>C-7:T>C</td><td>TGCAGCTTTCTGTGAAAAATTCTCATTTCAAAATAAGCAAATTAC</td></tr>
<tr><td>39715509|F|0-43:T>C-43:T>C</td><td>TGCAGCAGAGTTCTTGCTCTGTTGATCATATGGAGTATCAAGGTAGAGTTTTAGGCCCCGTTTGATAAC</td></tr>
<tr><td>39727814|F|0-66:A>C-66:A>C</td><td>TGCAGTCAGCAGTTCCTATCAATCCAAGAGATTTTCTTTTTGCCACACTGTTGGGTCACCCATTTTAGA</td></tr>
<tr><td>39719996|F|0-29:C>G-29:C>G</td><td>TGCAGCATGTATATGTCTAAAGAACGTTGCTCATTTTTCATTTAC</td></tr>
<tr><td>39727438|F|0-10:C>A-10:C>A</td><td>TGCAGAGATACTATAGATCATCAATCTACTTTTATATTCAAGACCCTTTAGTGCGATTTACTTTTTCTT</td></tr>
<tr><td>39727216|F|0-31:C>G-31:C>G</td><td>TGCAGTATTTCCAGCTCCAAATAGCCTCAGGCCAGGTACTTTAC</td></tr>
<tr><td>39727733|F|0-9:T>A-9:T>A</td><td>TGCAGGGGATAGTAAGTTACGCAAAGAGCTATCGTGGAGTCACATAAAATTATATACAGAAAGTAAACC</td></tr>
<tr><td>39720137|F|0-7:A>G-7:A>G</td><td>TGCAGCTAGTTTATGATATCAGGATATTATCAACATTAC</td></tr>
<tr><td>39728180|F|0-6:T>C-6:T>C</td><td>TGCAGATGGTGTTTGCTGTTTAGTGGAATGCCCGACTATGGATGCTGTAATTCAGTCTTGATTAGTTGT</td></tr>
<tr><td>39721493|F|0-37:A>C-37:A>C</td><td>TGCAGAATGAAAGCAACAATCGCCACCACAAAAACGTAAAGTTCATGTGTGCTGATGTAACTTCTCCAG</td></tr>
<tr><td>39724154|F|0-46:C>T-46:C>T</td><td>TGCAGTGTCACCATCGACCATTTGTGGCTCACCACCGCCGTCACACCGGACTAGTGTGTTGGGTTCTGT</td></tr>
<tr><td>39719507|F|0-35:C>A-35:C>A</td><td>TGCAGACATAATGTGAACAAACCCACGATATAAAGCGAGTATTAC</td></tr>
<tr><td>39717394|F|0-5:G>A-5:G>A</td><td>TGCAGGGTGAGTACTAATTCTAGATTTTAGCCTGTGTGGTGCTTGAAACTTTTCCTAACTGCTACTGAC</td></tr>
<tr><td>39726936|F|0-32:T>C-32:T>C</td><td>TGCAGAAACAACACCAGTAACACTCATGCAAATAAACCAAATTTATTTAC</td></tr>
<tr><td>39725331|F|0-25:G>T-25:G>T</td><td>TGCAGTGGACCATGGTTGGATATTTGCTTTTACTATTTTACCCTTTTTAC</td></tr>
<tr><td>39715214|F|0-52:T>G-52:T>G</td><td>TGCAGATTTTGTTTTGAAGTGCCAGGTTGTTTCTGTTTTTTTCTTACATATTTCCTAAAGTGCATTTTG</td></tr>
<tr><td>39715005|F|0-26:A>G-26:A>G</td><td>TGCAGATGCTTCGTGCGAGGGGAAAGAAGTGCGACTATCAGCCAATATTTATATGAAGTCATAATTAGG</td></tr>
<tr><td>39717973|F|0-63:A>G-63:A>G</td><td>TGCAGTATTCTGCTTGAATTTTCATTTTTTCTGCTTTCTTGGTCACATCAATTATGATGCACAACCTAT</td></tr>
<tr><td>39714874|F|0-10:A>T-10:A>T</td><td>TGCAGATCATACTCCCGAGTTCGTTCTCGTCAACAAGGAACCAGAGCCTGATCCTGAAGATCCATCAAA</td></tr>
<tr><td>39715784|F|0-6:A>G-6:A>G</td><td>TGCAGCATTTCACTGGAGAAGTTTCCAGAGCATATCGCTCTCTGGTACTGAGTAGATACGGCATTATTA</td></tr>
<tr><td>39721586|F|0-60:G>A-60:G>A</td><td>TGCAGACATAGTTTTGATTGCTTACATTTCATAATAAGCTACATGTCATTATTTCTTTTCGATCTTTTC</td></tr>
<tr><td>39722528|F|0-15:T>A-15:T>A</td><td>TGCAGCATCCCTCCTTGCGGTTTTGCACTTATTTTCCAGTCTCTCGTTGTTAGTGGTATTCTTCCAATA</td></tr>
<tr><td>39717695|F|0-66:C>A-66:C>A</td><td>TGCAGTAAAAAAAACAAATTGCTACAGTAATACTAGTTTTTTACCTCCTCTATTTTTTCATCTAACCAT</td></tr>
<tr><td>39715674|F|0-46:C>T-46:C>T</td><td>TGCAGCATATGTGAAGAGTTGGTTATAAACTTCTAAAATATTTCCACACATAGATACAATTTCTTTCAC</td></tr>
<tr><td>39722568|F|0-19:G>A-19:G>A</td><td>TGCAGCATTTGAAAAGAATGATTGAACGTGATAGTACATAATAATTGAATTACATGACAAGTTGGAAGT</td></tr>
<tr><td>39721473|F|0-53:G>T-53:G>T</td><td>TGCAGAATATTTCCTTTTCTTTGAATCTCTTTTACCTCAAATAGGATCTTTTTGATATTTCTATATTTA</td></tr>
<tr><td>39726489|F|0-35:A>G-35:A>G</td><td>TGCAGGGTATTTATAGCGGTGAAGAGTGAAGACGCAGTGAAGACGAGCGATGAGGAGGCAGCTTCCCAC</td></tr>
<tr><td>39721673|F|0-41:T>G-41:T>G</td><td>TGCAGACTTCCAAATGCTTCAAGTTTTTGCTATTCCTAATATTATCAATTCAGCAATGCAGGCATTCAT</td></tr>
<tr><td>39715981|F|0-11:G>T-11:G>T</td><td>TGCAGCCGCACGCCACGGAAAACTCTGTCTCCTTCGATCGTCTTCCCGTTACCCAAATCCGCACCGTAA</td></tr>
</table>

<table>
<tr><td>39715042|F|0-46:A>T-46:A>T</td><td>TGCAGATGTAGAATTTGAATTGTCTACCTGTACTTCTCTTTCGTGGACATCATTATGATACACTGAATA</td></tr>
<tr><td>39714239|F|0-44:G>C-44:G>C</td><td>TGCAGACGATGTTATTTTATCTGCAAAATAACAACACCAAATTAGTTGAGTTAGCTTCTCACATAATTT</td></tr>
<tr><td>39716132|F|0-19:A>G-19:A>G</td><td>TGCAGCGATTATCGACGACAACGAGACGGCGGCGGCGGCGGCGGCGGCCAAGGCGGTGCTCTGTGTTTC</td></tr>
<tr><td>39714302|F|0-11:T>C-11:T>C</td><td>TGCAGACTGTGTAACACCTGTAGGTTTCCCCTTTTGATGGACCTGATGAAGCGATTTTTTGAAATCCCT</td></tr>
<tr><td>39729506|F|0-16:A>G-16:A>G</td><td>TGCAGGTTGACGACGCAACGACATACTCATACCACGAAATTACGTAGTTTTCTTACATCGGTTTACAGA</td></tr>
<tr><td>39717837|F|0-10:A>G-10:A>G</td><td>TGCAGTACCTATACTCATGGATTCTTCAACCCACACTAAAGCTACTTCAGTGGAAACACCTACAAATTC</td></tr>
<tr><td>39726631|F|0-24:C>T-24:C>T</td><td>TGCAGTATGTTGTTGGATCAGCTCCTCTTTGGAAGGACCATCAATAGTGATGATTTTCCCAAAGATTGA</td></tr>
<tr><td>39722087|F|0-5:C>A-5:C>A</td><td>TGCAGCTGCCTACGCTGTACGCTTCTTTTGATATTTTTTGTGACCTTGTACACACATCCCCCAATTGGA</td></tr>
<tr><td>39714242|F|0-21:A>C-21:A>C</td><td>TGCAGACGCAATGGATGGGGCAACAACGAATCCAACAATCTCATTCACATTACCAACACGAGGATAAAT</td></tr>
<tr><td>39720274|F|0-36:G>C-36:G>C</td><td>TGCAGGAAAGTATATGATCAATGCATGACCAAAGAAGAACAATACCTGCCATTAC</td></tr>
<tr><td>39714174|F|0-10:A>T-10:A>T</td><td>TGCAGACATGAGAACATTATAACATTATGTTCTTTTATGGTAGGTGACTTTACTCGGTTCGTTTGTTGG</td></tr>
<tr><td>39714396|F|0-17:G>A-17:G>A</td><td>TGCAGAGACGGCGGCGAGGAGGCAGAGGAATGCGCAGAGAGGAAGCATTTTTGGATTGTGGGAATGGGA</td></tr>
<tr><td>39720248|F|0-45:A>G-45:A>G</td><td>TGCAGCTTTCTGGGTACTTGCAGTGGCTGGAGGAGAGAGAGGATGATCATAAAGAAGATCGGAAGAGCG</td></tr>
<tr><td>39722187|F|0-13:C>A-13:C>A</td><td>TGCAGATTGGTTGCAGGAATCAGCTCCAGAATTCTGGGTGTTGGAAGCTCTGTGAGGACGATGAAATCG</td></tr>
<tr><td>46753849|F|0-39:C>G-39:C>G</td><td>TGCAGAAGAAACGTAACACCGACCCCTCACCTTCCCTTTCTGATGGGTTCCAAAGTCATGCATTTTTTT</td></tr>
<tr><td>39716318|F|0-38:A>G-38:A>G</td><td>TGCAGCTCAGATTAGGCCTTTGATTTACTGTGTTTATTATGGCTTCTTTGAATTTCAAATGTTGAAAAA</td></tr>
<tr><td>39725021|F|0-18:T>A-18:T>A</td><td>TGCAGCTGCTTCTTCTTGTGTGTCTAACCAAAAAAAAACCAAAAAGTGATTAC</td></tr>
<tr><td>39725890|F|0-68:A>T-68:A>T</td><td>TGCAGATGCCATTCGCACAATTCTTGGAATTTTCGGTGGGTTTTGTTTCTGTTTCTGTTTCTGTTTCTA</td></tr>
<tr><td>39723415|F|0-15:T>C-15:T>C</td><td>TGCAGGTTTGGAGGATGAGGGAGTGGAATTTTTGAAGAAGTGTGAGGACAAATTGGGAATTACTCGTCG</td></tr>
<tr><td>39717095|F|0-23:T>C-23:T>C</td><td>TGCAGGCATAGAGGATATGAATATGCTGGTATATGGTCCCTAAACTTTATCTTGTTACTGTTGTTTGTT</td></tr>
<tr><td>39724155|F|0-61:T>A-61:T>A</td><td>TGCAGTGTCATATGGAGAGAAAAATAAATGCTAACTGCTAAACTCAAACCTGAGGCATCACTCATTTTT</td></tr>
<tr><td>39721537|F|0-62:G>A-62:G>A</td><td>TGCAGAATTTCTTGGACTTTAGCTTTGCATGCTTCACATTGAACACTTGCATTGAGAACACAGCTCTGC</td></tr>
<tr><td>39718045|F|0-5:T>C-5:T>C</td><td>TGCAGTCAGATCCTTCAAACAATATATATAAATATATATGTATATTCACCTGTCCATGTACTTTACAGA</td></tr>
<tr><td>39713712|F|0-33:A>C-33:A>C</td><td>TGCAGAACCTATCTCATCCTCTTTTTGCTAAGGAAATTCAAGGTTTGTGGCTGATCTTTCAATTTGGAC</td></tr>
<tr><td>39714613|F|0-34:G>A-34:G>A</td><td>TGCAGAGGTATATACTATATAGTCCTGTGATCATGAATATTTAGATCTAACCGTATGATGTGAGGTGTT</td></tr>
<tr><td>39723458|F|0-49:C>A-49:C>A</td><td>TGCAGGGATATTTCTCTCAATTGGACCTGGCGATCAGAATGATACCAACCATCAGAAGCAATAGCACAG</td></tr>
<tr><td>39716905|F|0-7:G>T-7:G>T</td><td>TGCAGGAGCCATCACTATATTCATGGTTGCTTCTTTATCTGTATGGATTACGTTTTATGATCGAATAAT</td></tr>
<tr><td>39726777|F|0-31:T>G-31:T>G</td><td>TGCAGTTATTCTATTGTTCCACCTCAGTTTATTCTTGAAGAACATTTGAAGGTCCCAAACAACACCTAA</td></tr>
<tr><td>39723863|F|0-5:T>C-5:T>C</td><td>TGCAGTATCCTCAACAAACGCGCGCGTGATTGTCACTCTCCTCCCTATGCGTGTGGGATTTGCTTTTTC</td></tr>
</table>

39716850|F|0-10:T>C-10:T>C	TGCAGGACGATCAGCTCCTAGTTCATTTGAAACTCGCGTGCTTATGAAATGAAGTAAGGACAGAAATCT
39715902|F|0-45:T>G-45:T>G	TGCAGCCATGGCTTTGTTTGCTTTATTGCCCACTTTTCTTCATTTTCCTTTTGAAAAACTCAGTACAAA
39725918|F|0-53:G>A-53:G>A	TGCAGATTGAAAAGCACTTCAACGACTTTGATCTTCCCCCCATGCTATATAGAGATTATCTCTCTCTCT
39716485|F|0-41:G>T-41:G>T	TGCAGCTGGTGGTTTGTTGTTGCTGTTGCTGGTTCAATATTGTTGCTTCGTTCACTGAGATTATTGGCT
39719599|F|0-9:A>G-9:A>G	TGCAGAGCAAAGGGTGTAAAGAGAGTTTTAC
39725228|F|0-40:G>A-40:G>A	TGCAGTAATAAACTGGATGTATAACTTCAGCAAAAACTTGGGAGCTTATCGAGGGTTTAC
39715360|F|0-10:A>C-10:A>C	TGCAGCAATAAGTTGCTCTATGACATTTAGATATAGGTTCGAAAACCGTTAGATACATATTCAAAATAT
39718044|F|0-59:A>G-59:A>G	TGCAGTCAGATAAAGCCTAATTAGATGCAGAAACATAGGCTAGCCAGTTTGATATTTACAAGTATAAAA
39726536|F|0-49:T>C-49:T>C	TGCAGGTGAGTGTTTCTGATCCAAAAAAATACCCTTGAACTATAGGGAATGACATATTCTAGTGATTTT
39715239|F|0-59:T>C-59:T>C	TGCAGCAAACACTTGTCTTTTTAGTGCTCATTGTGCTTGAATTAGATTACTTGTCTTAGTTTGTAGTTC
39717866|F|0-31:T>A-31:T>A	TGCAGTAGAATAAGTAGCCATTTTAGTCATATTGCAGCATCAATATGAAGCAAAGTATATAGGTAATAA
39722247|F|0-34:G>T-34:G>T	TGCAGCAAAGTGAAATTTATTTTATCATAAAAAAGAACCTAAAAGACATGGAGAAACCAAAGACATCTT
39717452|F|0-61:A>C-61:A>C	TGCAGGTACTGATTTTTGCACAAGATCCAGGAAGTGAGGTATTTCCTCATCCTGTGGGTTTAGTCTGAA
39721236|F|0-26:C>T-26:C>T	TGCAGAAATGGAAAACAAGGAGACCGCCGCTGATGCCGATCGTCCATGGCAGTCTTACAACACTGTCTA
39725959|F|0-6:A>G-6:A>G	TGCAGCAAAAGTCAAATATGTTGCATACAAGTACAGTTTGAAGAATGGAAGGTGTAACAGTTGCATAAA
39717271|F|0-59:A>G-59:A>G	TGCAGGGAGACCTACATTTTACTCATTCTATTACCTTTTAGCTCAAAATTTTGGTTCCAAATTCCTTTT
39728166|F|0-29:C>T-29:C>T	TGCAGAATCCAATGACGAAAAAACTTTGACGCTCTCATAAGTGAAATCAGGGAGTTTGTAATAGTCTTG
39715418|F|0-34:G>A-34:G>A	TGCAGCACAATTACTATAAAGGGAAAAAGGAAAAGGCCTTAGCTTTTTTCTTTCATTTCATCACATCTC
39729557|F|0-13:G>A-13:G>A	TGCAGTTCAAAATGAATCTGTTCCCATGCTTGTCCACCTTTTGAGGACAAGAGAGGTCCTTTAC
39725009|F|0-7:G>C-7:G>C	TGCAGCTGTTAGCTGTACAAAGAAAAGTGTAGGAAAAGTAAATTTAC
39727282|F|0-34:T>C-34:T>C	TGCAGTTTGATATTCAACGAGCGAGTTTTTGGTTTCCTGGATTGCTTTTCTTTAC
46764503|F|0-14:G>A-14:G>A	TGCAGGCTCAAATAGCACTATTCCAAACTCTCCCAACGATGGTCACCTGTAACTGGTGCACAAAACAAC
39713644|F|0-19:A>T-19:A>T	TGCAGAACAAAAAAAGAGAAAAAGAGCCCTAATAAAATATATGTTCTTTACTTGGTGGATTGTTTTTAT
39716592|F|0-61:T>A-61:T>A	TGCAGCTTCTCTACCTATTCCCAACTCTGCTAGAGGCAGTGACATTCTTACGTGGCTGCCTTCTCAAAA
39727151|F|0-46:C>G-46:C>G	TGCAGGCAGATAAAGATAAACTTTGGATCACTTACAAGGTAATGCACCTCTCAGTTTTAC
39716574|F|0-57:G>C-57:G>C	TGCAGCTTCCTGTCAATAGATACACGAAGGTTCTGGGGCACACCAGCCTTTTGGCAAGGCAATGTCTCT
39726968|F|0-6:A>C-6:A>C	TGCAGAATCTAATGGCAGCAAGGACTTCATTTTCCCTCAAGATTGAGATTAC
39717030|F|0-68:C>G-68:C>G	TGCAGGATTTCAAATTCAAGGTATATATATTATGCTTTCTTACTAAGAAACACAAATACTTTAGTTTGC
39721403|F|0-61:A>C-61:A>C	TGCAGAAGGCATCAATTTGTATCTGAGCTAAACAAGACACGTGCCACTATGTCGAAACTCAAGTCAAAA

39720476|F|0-13:C>T-13:C>T	TGCAGGTACTCACCTTTTCATGGATTAC
39724742|F|0-5:A>G-5:A>G	TGCAGAGCAAAACCATAACTACATCTTTCCAAAAGGAGAATTTAC
39729119|F|0-27:C>T-27:C>T	TGCAGAATGAATCGGTAACACCTCATTCCCTCTAATTATTTCCCTGCTTGCTTTTAC
39724618|F|0-9:T>A-9:T>A	TGCAGAACATTGAACTTTAGAACGACCTATGAGATTTTTAC
39728967|F|0-20:G>T-20:G>T	TGCAGGAACAAAATGGTGGTGCTTCGGTTCCACTTCATGAGAATCAATCCCAAATTCACATTTAC
39718735|F|0-35:G>A-35:G>A	TGCAGTTGATGCCAACTTTTTCTTTTATTATTATTGTTGTTGTTTTTGTAAACTTCTTTTAC
39723761|F|0-42:T>C-42:T>C	TGCAGTAATATCTGCAATGGCAGCAAAATAAATTGATATGATTAGAAAGCTAGATATTTTTGCCCTCTT
39725885|F|0-63:C>A-63:C>A	TGCAGATGATATCTCTACCTTATAATATTTCCGAGACATGGGCACTATTACAAAAACTTCCAACACTTC
39724060|F|0-68:C>T-68:C>T	TGCAGTGATGCTATAACTGTTCTAGATCTAAGAATTCTAGCTTATCCCTTATCACCCCATTGTTATTAC
39717151|F|0-54:G>T-54:G>T	TGCAGGCCTTCGAAAGCTTTCTTTTCTCCTTCTCTTCTCTGTGAGTTGCTGCTTGCTCTTAGACGTAAT
39716666|F|0-14:C>A-14:C>A	TGCAGCTTTGAATCCTCTAGATTTTCATTGTTCAGACACTAATGATTTTACTCATAGCTCGACTCCACC
39716737|F|0-24:C>G-24:C>G	TGCAGGAAATACATAAACCGTCAGCATAAAGAATTACAAATGTTACTAAAAGCTTTGTAGACAGATCAA
39718519|F|0-37:C>A-37:C>A	TGCAGTGTTGTGAGATTTCATCTCTGTGTCATCTTCTCAAATTTGAAGCTATTTTCACGGTGCAGGAAT
39723252|F|0-21:T>G-21:T>G	TGCAGGATCTGAAGTTCAGAATTTTATAGACAGACAAAATTGGGGAGGCTCTTAGGAAGAGATAGCTAC
39724359|F|0-23:T>C-23:T>C	TGCAGTTGGGATGGAGTGGGCTGTGATCCTACCAATGGTCATGTTCTCAAGCTTGATCTTCGAAACTAT
39725859|F|0-22:T>C-22:T>C	TGCAGATCTATGATCCTATGTATCGGCACATTTCACGGTCACAAACAAATGTACCAGTACAACAATCTC
39724151|F|0-19:A>T-19:A>T	TGCAGTGTATCTCAATCAAATTTCAGAGATTCTGTTGCACGCTCGCTAAATATGTCATTTCTATTTCTT
39717471|F|0-53:A>T-53:A>T	TGCAGGTATATTTGTCATTGAGCATATAGTATATCTTGCCACAGTTTTACCATATAGGCTCTAACTCTT
39715933|F|0-21:G>A-21:G>A	TGCAGCCCATCGGCCCAATTTGGTGGCCCAAATATTGAAACCTCCGAAATTTCCGTAAGTAAGATTTTA
39724113|F|0-66:T>C-66:T>C	TGCAGTGGCAAATGAAAGCAACACTTACGAAATTGGTGAGTGAACTCCATCAGGTCATTCTTGTGTTTA
46756900|F|0-16:A>T-16:A>T	TGCAGGTTTTTCTTACAATTTCTCCGTTAGTTTCAATCCATTATTGCGTGTTTTTTATAATCTCACCCC
39718181|F|0-6:C>G-6:C>G	TGCAGTCTGCAAAATCCTATTCTTCAATTGTTCCAATTTTGCTTGGAAACAGAAAACACATAAACATAA
39715617|F|0-28:A>G-28:A>G	TGCAGCAGTAGATAAAACCTTATTAGCAAGGAATGTATGAGATGAAACCTTATTGAAACTCTTTTTTTT
39715481|F|0-11:A>C-11:A>C	TGCAGCAGAACACAAAACTTTCAAAACTAAACTAAACAAAAATAAAATAAAATAAAACCCAGAAACAGC
39716038|F|0-9:T>C-9:T>C	TGCAGCCTCTACACTGCATTCAACGTAACCTTCCTCTCTCCATCTCTATACACCTCCTTTTTTTCTGAT
39728490|F|0-19:A>C-19:A>C	TGCAGTCTAATAACCCAGTAAAAAATAATCATGAAAATCTAAAAAAAGAACACTTTAC
39720144|F|0-12:C>T-12:C>T	TGCAGCTATGGGCTTTTTCTAGTTTTAC
39719493|F|0-47:C>A-47:C>A	TGCAGACAAAGATCATACTAAGCATATGGAGAGCATCACACTCAAACCGAAGGTTAC
39715475|F|0-61:G>A-61:G>A	TGCAGCAGAAAAGGCATGAGTTTGCACCTCGATTTTACCAAAACAAGAGACAGGGAAAGACGTACTCGA

39719393|F|0-19:A>T-19:A>T	TGCAGAAGATTGAGTATTGATCAAAATTATTAC
39718581|F|0-33:C>T-33:C>T	TGCAGTTATATATTACCGGTGGAAGAAGCTTGGCTTTCAAATCTATATGTTGTATATCCAAGGAGCCTT
39724461|F|0-48:T>G-48:T>G	TGCAGTTTTCATTATTGATTTGCTTGAAATGTATATGCATTCAGAAAATAATCTCTCACCTAACTTTTA
39718496|F|0-49:T>G-49:T>G	TGCAGTGTGATATATGTTATAGTCAAGTTTTTCTGTTATCAATTCAAATTCTTAGTGGATATTCAGAGA
39719308|F|0-15:T>G-15:T>G	TGCAGAAAGACAGAGTAACAGGCAGTAACATTACCTTTCAATTGATTAC
39726857|F|0-12:A>G-12:A>G	TGCAGTTTCACTATGAACACGATACACAAACATGACAGAACCACTGACATAGTGACACATCATTCTCTA
39714445|F|0-58:C>G-58:C>G	TGCAGAGATATATATGGAGAGGCAGCTGATAACAAGAAAGATTCTAAATATATAACGTCTTAC
39715445|F|0-56:T>C-56:T>C	TGCAGCACCTCTTAGGGAGACAACATCACAACAACCCTAAATTTACTCAATTTCCTTTGGATTTTAGAG
39724907|F|0-10:G>A-10:G>A	TGCAGCAGCAGCTGGGTTTTGGCCATGGAGTTCAGATCAAAATTCCCATTTTCATTAC
39725795|F|0-36:C>G-36:C>G	TGCAGAGTCTTGATCTTGGTAGTGGAGTTGGTCCTGCGGGAGGTTTCTGTTCATTGTATTTGTTGCTTG
39722548|F|0-61:C>G-61:C>G	TGCAGCATGCAGACAAAATAAAGTGTAAAGAACGAAGAAACTTCCCAGGGCCTAATGAATACTATTATC
39724681|F|0-13:G>A-13:G>A	TGCAGAATTTCATGAAGCCATCCCAATAGTAGAAAGTTCACATGTTAC
39725231|F|0-22:T>C-22:T>C	TGCAGTACAACACAATCAGACGTCAAAGACAAGGACAAGACCAGAAAAATTAC
39713616|F|0-25:T>C-25:T>C	TGCAGAAATGGGGAATTGAATTTGATGCAGGGGCCCTGAGTCTACGCTAAAGATTTTCCCTGACATGAT
39728151|F|0-7:G>T-7:G>T	TGCAGAAGAATGTTGGTTCAATTCTTGAAAAGCAAGCAAATTTCTTCCATCTCCAAGCCCATCAACAGA
39720861|F|0-34:C>T-34:C>T	TGCAGTTGGAGATTTTGTATCAACCAATGAATAACCGACGTTTATATAGAGACTTTAC
39729149|F|0-21:T>C-21:T>C	TGCAGCAGTGAAATGTAATTTTTAGCAATCTGTGGAAGCATTTATAAGCTTCATAACAATTGTTACAGA
39718742|F|0-37:G>A-37:G>A	TGCAGTTGCAAAATCAAAATGATATATATGGGCAACAGCAAAGAAGGAAAAAATTCAAAAACATTACAT
39717250|F|0-20:T>G-20:T>G	TGCAGGGAAGAAAAGAAAAATGCAGGTTTTATCGAAGGGACACATTATAGAGCAGTTTGCAAGCTATTT
39718166|F|0-8:T>C-8:T>C	TGCAGTCTTATTATCCTTCGAACACATGACACACCAAGATGGAGATAGAGAGCAATGAGGCATCCTTCG
39723035|F|0-32:T>C-32:T>C	TGCAGCTTCTGCAAGAATGAAAAAGTAGTTGATTACCAACAATGTCCCCGAATCTAACACCAAATAAAA
39728452|F|0-42:T>C-42:T>C	TGCAGATACCCTTCATATTAGTTTTACGAATATCGGGTAGATTATATTAC
39714490|F|0-41:A>T-41:A>T	TGCAGAGCAGAATATTTTGGCTAAAATGGTGCAAGTGTAACACGATGCGAGGCAAACAACCAATTCATG
39725627|F|0-10:T>C-10:T>C	TGCAGAATGCTTTCTCACCTCTGTAAAACGAATCAAACCCATAAGAACAACTGTGTTTACTGTTTACGA
39724036|F|0-43:C>T-43:C>T	TGCAGTGAATAAAAAGGTGATTGATGAGGTTTCCATGTCACTTCAGCAAAGCAAGCATCTGTTTTGCGA
39714011|F|0-10:C>T-10:C>T	TGCAGAATCGCTATTCAAAGAAGGAATCGGCTCTAATTACTCTCGCATTTCTCTAGATCTAACTATCAC
39726824|F|0-9:C>A-9:C>A	TGCAGTTGGCACGCTTATTCCAAATGCTTGAAGGCCTCGGTTCAGCAATAGAGAAAGATGGTTTGCTGG
39724094|F|0-30:A>T-30:A>T	TGCAGTGCTGATCAGTGATCACTGATCTGCATTCTGAGAAAGCTAGATTTCCAGCTCACATGGACGAAT
39724920|F|0-18:C>A-18:C>A	TGCAGCAGTTCATTGTGTCAATTTTAC

<table>
<tr><td>39725465|F|0-26:A>T-26:A>T</td><td>TGCAGAGGGAACATGAATCAGAATGTATGTCTAGTAGAGTCAAACAGAAGAAAATTACTAGGTGATAGA</td></tr>
<tr><td>39724413|F|0-21:T>C-21:T>C</td><td>TGCAGTTTCCCCATTCTCTGCTGCACTTCTCCTTTTTGGCTTTACCTCCATTATCAAGATCAACTCTGC</td></tr>
<tr><td>39725145|F|0-24:G>A-24:G>A</td><td>TGCAGGGGGCAAAAATACACAAAAGGGTTTTAC</td></tr>
<tr><td>39724291|F|0-22:C>A-22:C>A</td><td>TGCAGTTCTGCTTGCTTTTGTCCTGCACGTTGGGGGAAAGGTAAAAATGATAATCATCTCCACTCTTCA</td></tr>
<tr><td>39727711|F|0-54:T>A-54:T>A</td><td>TGCAGGCCTGTATTTAGAATATGCAAATGAATGAGTTTCAAAAGTTTTCGACCCTTGTAACTCAGTAAG</td></tr>
<tr><td>39714371|F|0-20:A>G-20:A>G</td><td>TGCAGAGAATCCCACATATCATCATTCACATTCTCAATGCCCTAAAACAGAGCCCTTGTTTTATATGAG</td></tr>
<tr><td>39714958|F|0-50:T>G-50:T>G</td><td>TGCAGATGACTTCTGAGGTGATGCGACATCTGTTTCCAGAAAACCTGTCATTCAGAAAATTGTAGTCTC</td></tr>
<tr><td>39716685|F|0-18:A>C-18:A>C</td><td>TGCAGCTTTTCCCATCTCAAAGAGATGGAAGGATAATGGAAGAAAGAATAATAAGCAATTAGTAAAAGG</td></tr>
<tr><td>39725089|F|0-45:T>C-45:T>C</td><td>TGCAGGATGAATTGTTCAATCTTATTGTAAATTTGTGGCAACATTTCTTTAC</td></tr>
<tr><td>39714676|F|0-52:G>A-52:G>A</td><td>TGCAGAGTGAGTTCAATTTAGAGTCAGCTTCTTTGCTTTTTCCACATAATTTGGAATAAAAACCAAAAA</td></tr>
<tr><td>39713465|F|0-13:A>T-13:A>T</td><td>TGCAGAAAATGGAATGCGCCTTTGCAGAGAAGGCAAGTTGAATTGAACAGAGGAGAGGAGAGGTGAGAG</td></tr>
<tr><td>39722072|F|0-64:T>A-64:T>A</td><td>TGCAGATGATCTTGCAGCACCGAGGAGTCAGGCGATGGTGCAGTACCCATAATGGCAACTGCTATAACA</td></tr>
<tr><td>39715576|F|0-66:G>A-66:G>A</td><td>TGCAGCAGCTGCCACAAAGCTCTCCAGCTGAGACATCATAACCAACCCGATAACGGCAGCAACAGCGAC</td></tr>
<tr><td>39717722|F|0-13:C>T-13:C>T</td><td>TGCAGTAAAGTGTCCAGCAGGTCAACAATTATGCAAGCATAAAGCTATAACATTCATAACTTATAAATT</td></tr>
<tr><td>39713439|F|0-25:C>G-25:C>G</td><td>TGCAGAAAAGGAGACCTAACAACAACAAGAAGAAGCATCTGCATTTCTTCTAATCTTCATTGCTGAAGA</td></tr>
<tr><td>39722395|F|0-13:G>A-13:G>A</td><td>TGCAGCAGAATCTGTAGTCAATTGTGCTTTTTTATTTGAATCATTCAAATGCTACAATCTAGATTTGAG</td></tr>
<tr><td>39713828|F|0-27:A>G-27:A>G</td><td>TGCAGAAGCAATTTGGCTGCCTTCAAGATCCCAAAGAAGGTCTTCCTCACAGATTCATTGCCCAAGACT</td></tr>
<tr><td>39727350|F|0-65:T>A-65:T>A</td><td>TGCAGAACAGACATTTGCAGAATCTTATTTTATGTTATTTTGTTTATTATTTACTTTTCTTTTTTTACA</td></tr>
<tr><td>39722738|F|0-25:G>T-25:G>T</td><td>TGCAGCCTGCAACTCTCTCTAGTAAGGCCTCACATCTCTACTATTTTCATATTTTATATTCTCATCATA</td></tr>
<tr><td>39720081|F|0-46:C>T-46:C>T</td><td>TGCAGCCTTCTTTCAAGGGAAAAATGTATTTGTATTATTGTATGTTCCTTTGAAATTTAC</td></tr>
<tr><td>39718030|F|0-7:G>A-7:G>A</td><td>TGCAGTCGCCATTGTTATAGTTTCAATTCTTGGAATTCTATGGTGGAGAGGTTGCCTTGGACGAAAAAG</td></tr>
<tr><td>39715324|F|0-44:A>T-44:A>T</td><td>TGCAGCAAGACCGAGCAGCCGGTCATTTGTTCCGATTGCCTGAAATCTGACTCCGGAAGCCAGACCGCC</td></tr>
<tr><td>39727513|F|0-14:T>A-14:T>A</td><td>TGCAGCAAATTGACTACTTTCAAGTTTGTAGACCAAACACAAATTTCGATATTTGAGAAGTTTTACACA</td></tr>
<tr><td>39717952|F|0-56:G>A-56:G>A</td><td>TGCAGTATGGAGAGACCCCATTGCATATGGCAGCAAAGAATGGGTGCAATGATACAGCGTCGGCACTAC</td></tr>
<tr><td>39717355|F|0-49:G>A-49:G>A</td><td>TGCAGGGGTAAAGAGATTCTAATAAAACTTGTGGCACAAGCAATCTCTAGTTGTACAATGTATTGTTTC</td></tr>
<tr><td>39723231|F|0-63:C>G-63:C>G</td><td>TGCAGGAGTTCATGTAGAGTCTGATGCCATCCTAGTAGTGAAGCTTTTGACGTTCAAAGATCACGATTT</td></tr>
<tr><td>39727642|F|0-16:A>T-16:A>T</td><td>TGCAGCTGTTGGAGGGATGATCAAAATAGGATGGAACCAACTCTTTTCAATTTTCAACCACCTTTTTGT</td></tr>
<tr><td>39715234|F|0-54:C>G-54:C>G</td><td>TGCAGCAAAATCAAAGCACAAATCAAAACCGAAGAACAACAAACAAACTCAAAACAAAAATCCAATCGG</td></tr>
<tr><td>39725423|F|0-21:C>G-21:C>G</td><td>TGCAGTTTTGCTTGCCTTTGTCTTGCACCTTGGTGGAAAGGTCAAATTTACTGATTAC</td></tr>
</table>

39714808|F|0-10:T>C-10:T>C	TGCAGATATATCTTTATTCTTCAATGAATGTAAATTCAAGAAATAATGAAAAGCATTTAGCCAGTTGTT
39717869|F|0-31:G>A-31:G>A	TGCAGTAGACCATCCCAGATGGTGCGTACAGGGAGTTGCATTCAGTTTCCTTGCCTTGAGCATAAGTTA
39718386|F|0-8:A>T-8:A>T	TGCAGTGGACACGGGAGGTGTCTTTCTAATGGACTTTGTGAATGTGGAAATGGTTACACTGGCATCGAT
39720209|F|0-50:A>G-50:A>G	TGCAGCTGTGAATGATAATTAGTTGTGTTTTTTGTGTGTAGGAAGTCAGAATTTTGTTAC
39728720|F|0-11:G>A-11:G>A	TGCAGATGGTAGATATAGTAAGAAAAACACTGGTGGGTTGACTAAACTCTTTAGTTTTATTTTCTTACA
39714960|F|0-36:A>G-36:A>G	TGCAGATGAGGTTTTCTTTTTTACATTTCAAAATTCAAAGTTGAATGTTTTGCATAACATGGAGTTGGA
39717330|F|0-67:C>A-67:C>A	TGCAGGGGAGAAGTCCAAGAAAGAACGGTTGGCTGTTCTAAATATAACAACTTATTGTATCCTAAGTCA
39718377|F|0-43:G>T-43:G>T	TGCAGTGCTTGCAGTAGATGGGATATGAATATGGATGCATATAGATTGGCACCAGAGCTTGCCGAGGTG
39725193|F|0-32:C>T-32:C>T	TGCAGGTGTGTGAGAATTGGCGTTTGTGAATCCTTTAGCTCTGAGATTTTGGTTAC
39726984|F|0-32:C>A-32:C>A	TGCAGACTAGAAAGATTTTCTGACGATGAAATCATTAC
39727153|F|0-7:C>T-7:C>T	TGCAGGCCGAGAGAGGGACCAATTCTTGAACTTTCTTTTCACCTTATTATCAGTGTTAC
39713325|F|0-59:T>C-59:T>C	TGCAGCAACCGCAAATGTTCAGAAATACTCGGGATTCCTGTTGTGAGTAGTAGTACGTGTGCATATACC
39723391|F|0-32:G>A-32:G>A	TGCAGGCTCAGATGGACCAACTATCTTAGGCCGGGCATCAAGAGGGGTAATTTCACTTACCATGAAGAG
39714605|F|0-21:A>G-21:A>G	TGCAGAGGGTATGGGCTGCGTACAAAAAGAAGATGCTTGGGTCTAATCCAAGTAAGCCCTAATTCCATT
39724633|F|0-31:G>A-31:G>A	TGCAGAACTTATGATATGAATGAAAAAAGATGTATTAC
39725300|F|0-25:T>G-25:T>G	TGCAGTGACTTTGCAAATATACTTGTTTTGACTATGAACATTAC
39728293|F|0-36:G>A-36:G>A	TGCAGCTGCCATAATATCTCTATGATGTTCTCCGATGACAGTTAGTCTATTGGCCTTCGAGATTTTTAC
39726600|F|0-56:G>A-56:G>A	TGCAGTACTTCTACTTTCGTTTGCTTCTGTACAACTTCCATCGCCCTCGATCCACCGAAAAACACGGTC
39713293|F|0-50:C>A-50:C>A	TGCAGTACAGCACTCCCTCAAAGGAGCAATAGTTGCTAAGCTGCAAAAGACAAAAAAGATACAAATCCA
39715821|F|0-10:A>T-10:A>T	TGCAGCCAAGAATTGTACAGTTTTTTTACCGTCTCCATTAGAAAACCTCCTGATCCAAAAATCAGACTT
39717352|F|0-9:T>A-9:T>A	TGCAGGGGGTGGGTTTACCTGCCTATATGTCAAAAAGTTTTTGTTATTGCATCTTTATCCCACATTTCC
39727482|F|0-5:T>A-5:T>A	TGCAGTTCTAGGAGAAAAAAAATTCTTCTAGTGGAGATAATTTGGAATCTACAACACCCGGTGCTTTTC
39721759|F|0-15:G>A-15:G>A	TGCAGAGATATGTTGGCATCTACTTGTTCTATTTGAACGACTGCTTGAACTTCCATCTCCATTTTGTTT
39722108|F|0-37:C>T-37:C>T	TGCAGATGGCAGATATTTGACTATATTGGACTAATATCGTTAGGATGTCATCTGCCTATAACTAAAAAT
39718130|F|0-28:A>C-28:A>C	TGCAGTCGATGTTTTCATTGATTATTTTACTATGTTATTGGTGAATCAACATGGTATGGTATTTTCAAC
39718966|F|0-44:T>G-44:T>G	TGCAGTTTTGATTGAATTGATTTGGTGAACCCATTTTTGAAGGGTTTCGACATTGTTGTGGAGGCTCGA
39724566|F|0-18:C>A-18:C>A	TGCAGAAAAACTAAAGCACAACCTACCCATGTTAC
39716358|F|0-45:A>G-45:A>G	TGCAGCTCGTGGATAGCTCGTGGATGGGTTCTATTCATTCCAATCATTGTAGATCGGGATTGTCGGAAC
39715750|F|0-58:C>T-58:C>T	TGCAGCATTACTGTCTTGAGTAGCACATTTCTTTTTTATTTGATTTAGCAATTTGAACCAGATGGTTCC

<table>
<tr><td>39720066|F|0-13:A>G-13:A>G</td><td>TGCAGCCTATAAAATCTCAATCATATTAC</td></tr>
<tr><td>39724236|F|0-9:A>T-9:A>T</td><td>TGCAGTTCAAGGGAAAATGTAGGAATCAATACGTCGGTTTCGGTATTTTTCTCGTTCTTATAGCGGTTT</td></tr>
<tr><td>39728736|F|0-5:C>T-5:C>T</td><td>TGCAGCGGTCTTTGTCTCTGTCTTCTCTTATCCCCCCCTTATACTTTTCTGTTGATGTGGATGTTACA</td></tr>
<tr><td>39714844|F|0-33:T>C-33:T>C</td><td>TGCAGATATTTGGGCTCTTGGCTGCGTGGTATCTGAGATGTTGACTGGAACCCAGGTTTTTCTAGATAC</td></tr>
<tr><td>39716542|F|0-8:A>C-8:A>C</td><td>TGCAGCTTATTGTTTTTCATTAGGGATTTTGTTTGTTTCAAGAAGATATAGTGAAAGAAAAAGACCCTA</td></tr>
<tr><td>39723777|F|0-17:T>C-17:T>C</td><td>TGCAGTACAAATAACAATATAGCTAGTCGTATTGATTTGTACTATCCCTTATCGGATGTTTTTCAATAT</td></tr>
<tr><td>39714151|F|0-6:C>A-6:C>A</td><td>TGCAGACAGAAGGGATTTGTGATCAGGTGAAGAAAGAATATCAGATGCATCATACATATATTGATTGAT</td></tr>
<tr><td>39725087|F|0-28:C>T-28:C>T</td><td>TGCAGGATCACTTTCCTGATGCTTCTTTCATGGTGTTTAC</td></tr>
<tr><td>39717385|F|0-36:A>G-36:A>G</td><td>TGCAGGGTCCAGTGCTTCAGCCTGCGCCCGAGTCCTAGTGGGAACAAAGTCTTCAAATCGTACGGCCAA</td></tr>
<tr><td>39713533|F|0-47:C>T-47:C>T</td><td>TGCAGAAAGAGGCCTGGAGATGCCGGGGGTTTCGACGCCGGGTGGAACACGGCGAAGTTTTTCCGATGA</td></tr>
<tr><td>39722939|F|0-5:C>T-5:C>T</td><td>TGCAGCTGAATTTTGTAAAGCTGCTTCTAGGAAGGGGCTGGTTGAGCTGTTTTTAGAAACTGTTTTTTT</td></tr>
<tr><td>39725909|F|0-9:A>G-9:A>G</td><td>TGCAGATTCAGTTTCCAAGAATCAGTTTCCTTGATACTAAAGCTCCAACTTGTCAAAACTAAGGGCAAA</td></tr>
<tr><td>39716193|F|0-12:A>G-12:A>G</td><td>TGCAGCGGTGGAAGCCAACGAGGTCAAACTCCAGCCGGTGTCAGATCGGATTTGAAAGCGGTTGGTTCT</td></tr>
<tr><td>39722353|F|0-27:T>C-27:T>C</td><td>TGCAGCACCATGCGACCCAAATTTCTCTCCTTTTGAACAAATCGTTTTCAAATCACAAACTCTAGAACC</td></tr>
<tr><td>39718963|F|0-56:C>G-56:C>G</td><td>TGCAGTTTTGAATCTTCTCTTTGCCTGAGGAGCTTCAGCATTTGTCCTGTGAAGCTCAGAGTTTCTGTT</td></tr>
<tr><td>39726943|F|0-49:A>G-49:A>G</td><td>TGCAGAAATCTTGACGATCTTCATTCCTGATAGAATATCCAAACTACTCACTACTTAC</td></tr>
<tr><td>39716973|F|0-25:T>A-25:T>A</td><td>TGCAGGATCATCTTGGCCCAGTGAGTCAAGCAAAGCTTGCACGTGGTACAAGCCCGCCGTCAGCTCATG</td></tr>
<tr><td>39720161|F|0-20:T>A-20:T>A</td><td>TGCAGCTCATTTTTCTTGTTTTGCATGAAGATAATTTAC</td></tr>
<tr><td>39720207|F|0-18:C>T-18:C>T</td><td>TGCAGCTGTCAAGGATTGCAAGGATTTTTAC</td></tr>
<tr><td>39727275|F|0-33:A>T-33:A>T</td><td>TGCAGTTTACAAGTTCGTAAACTTGAAGAAAAAATTTCGATATCAGCCGATGATGTATTAC</td></tr>
<tr><td>39724628|F|0-6:A>G-6:A>G</td><td>TGCAGAACCCGGCTACGTGTACCATTGTCACGTAAGTAGCAAGTTAC</td></tr>
<tr><td>39723094|F|0-29:T>C-29:T>C</td><td>TGCAGCTTTTTCCTGTACGAAATCGAAAATCGGTTTATGCTCGTTTGGTAACGTTTTCGTTTTGTTTTT</td></tr>
<tr><td>39714583|F|0-20:C>A-20:C>A</td><td>TGCAGAGGCTCTAGTAACGTCTTTCCTCTTTTATTCTAGCAATGACGTATAATCACTGACGGAAATAGT</td></tr>
<tr><td>39714792|F|0-60:T>A-60:T>A</td><td>TGCAGATAGGAAACCTCCGGGTGTAGGGCGTGGAAGAGGAAGAGGGCGTGAGGATGGTCCTGGTGGAAG</td></tr>
<tr><td>39713983|F|0-36:A>G-36:A>G</td><td>TGCAGAATATCTTACATGAACTACCAATGCTAATGCAATTTTTGTGTGAACAAATAGAGCCATTTAGAG</td></tr>
<tr><td>39715067|F|0-21:G>T-21:G>T</td><td>TGCAGATGTTTTGATCAGATTGCATCAACTACAAAGAATATCAATTTTCAAATTTTTATTCAACTAACT</td></tr>
<tr><td>39718600|F|0-20:C>A-20:C>A</td><td>TGCAGTTCAAAAACTAAAGTCTCTGGTGAAAAGAATAAATCACAAACTATCTCGTTTAGCACAAGGAAT</td></tr>
<tr><td>39722349|F|0-20:G>A-20:G>A</td><td>TGCAGCACCAGAGTCCATCAGGAACCAACAATTCATATAATGGCTCAACTGTTCGCCATAATTCCAATC</td></tr>
<tr><td>39716619|F|0-63:A>G-63:A>G</td><td>TGCAGCTTGGAAATTGTAATTGGCCACCAATTCAGAATGGAAATTTCCATAATCTGGCCAACAATCAAT</td></tr>
</table>

39722950|F|0-27:C>A-27:C>A	TGCAGCTGATTTCCATACATATTCAATCATGTGGTCTCATTGGCAAATCATGTAAGTAAGGAAAAAGAT
39717596|F|0-52:C>T-52:C>T	TGCAGGTTAGGTCCCATCCTGCTATTATCCATTCAATTTGAAATACTGAGTTCGAAAAAGAAAGTAGCC
39723751|F|0-36:A>G-36:A>G	TGCAGTAAGCAAGAATGTACGTTCTGCATCAATAACAGGGGGTTATTTCTATCGATCTAAGACCGATCC
39718960|F|0-64:T>C-64:T>C	TGCAGTTTTCTTTATTGGCTTCTAAACATGGTGGTGGTTTTAGGCTTGGCACTGACACCGCCAGTGTTA
39729214|F|0-62:T>A-62:T>A	TGCAGTAGCTAAAGTTTTGGTTGTATGTTAGCCACAAATATTTTCAAGTTATAAACCTTATTTAC
39713531|F|0-15:A>G-15:A>G	TGCAGAAAGAGAGAGAGAGAGAGAGAAGGGTACGTATGCAAATTGTTTTGAAAACAAGAAGAGAGAGAAAG
39719385|F|0-15:C>T-15:C>T	TGCAGAAGAGAAATACGATGAGTGTCATTTACGTACACGTTAC
39721128|F|0-24:T>C-24:T>C	TGCAGAAAACATCTGGCATCACAGTTAGTCTTTTCTTTTTTCCCTTATCAAATTTTCTTTTTGATTTGA
39721802|F|0-17:A>C-17:A>C	TGCAGAGCCGAAATCGAAACTTCCTTCTCCTCTCCGTCTCTCTTCATCAAATTCAACGGAAGATTTGAA
39719962|F|0-53:G>A-53:G>A	TGCAGCAGCTTGTTTTTCGAATCCAGGGCCCCCTAAATCTGTAGTAGGCTTTCGTTAC
39721971|F|0-29:T>C-29:T>C	TGCAGATAGTTTTGGCCATCAAAACTGTATGATAAATTCCAACAGGAACATACATACAGGGACGTAAAA
39715870|F|0-13:C>T-13:C>T	TGCAGCCAGCAGGCTACCTTCCAACTCGTTCCAACACTTTGAAAGGTCCCAAAATAAATTAGGGCAAGC
39719433|F|0-11:A>T-11:A>T	TGCAGAAGTTCAGCCTGCTTTACTCGAGTTTCTTGTTAC
39714373|F|0-21:C>T-21:C>T	TGCAGAGAATCGAGAGAGATTCTGTGGGAGAAAGAAAAATGAAGGAAAAACGACTTGGAAAGAATTGGG
39714940|F|0-27:G>A-27:G>A	TGCAGATGAAAATGGAATTTTCCCTGTGGACTTGACCCCTGATCTGTTTATGTCGACCGTGGAAGGTGC
39717704|F|0-55:G>A-55:G>A	TGCAGTAAAACGGCTCACCTAGGAGAGACATGTTCAGTATTCTTCTGATTTGCAGGGCAAATTATTTCG
39725205|F|0-16:A>G-16:A>G	TGCAGGTTTCAAAAGAACAATCCCACTGTTCTAACATCAATGCGAAGCTAGATTAC
39725212|F|0-29:C>T-29:C>T	TGCAGTAAAAAAATTATGCACATAATACCCATTTCATCTAATGTAAGTTAC
39715190|F|0-19:T>C-19:T>C	TGCAGATTTGACAGGACTCTATAGGAGAAAACTCCTCGACCTGAATTCTCGACATAATAAAAAAATTAC
39719922|F|0-49:T>G-49:T>G	TGCAGCACCTGTATCAAAAGAACAAGGATGAAGAAAAAACAATTCAGTATATTAC
39725239|F|0-33:A>T-33:A>T	TGCAGTACCAATAACAGGTCATTTCATAAATGTACAAGTATTGAAATCGTTAC
39717829|F|0-8:A>T-8:A>T	TGCAGTACATTTATTAGTGCAATAGGATAAAGATCTTTATATAACTGTGTTTCTTCCATTCCATCTACT
39728591|F|0-9:T>C-9:T>C	TGCAGGATATCTTTCTGAGTTGGATCAATCACCATCAGCTTCAATTTTGGAGTCAATGCAACCTTTTAT
39721154|F|0-7:A>G-7:A>G	TGCAGAAAATTTTTGATGCCACTCATTGCCAATTAGACGATTTGTTCTATTGCATTTGCAAATAGCAAT
39714815|F|0-55:C>T-55:C>T	TGCAGATATCATAAATGGCCTCATTGTCAAGGAGCACAGCGACATCAGTGTGTTCCAAGAGGGAGTGGG
39718348|F|0-6:G>A-6:G>A	TGCAGTGCCATTGCAAGTGATTTTGGGGGAGCACCATTTGGGTAAGCATTTTTTACTCATATCATTTTC
39717411|F|0-15:G>A-15:G>A	TGCAGGGTTTGAGAAGCTCTTTTTTGCATCCCTAAAGTATATCAACTGAAAAAAAAAACCATATATGAA
39717972|F|0-62:C>T-62:C>T	TGCAGTATTCGGATTCGGGTGAGGCTAAGGCTCACGTTCTACACTTCCCTACGAAAGAACACCGGTGGA
39729035|F|0-56:G>A-56:G>A	TGCAGTGCCCCTTAGAGAAGGGTAGGGTGACCTTGAAATGTCCCTTACATTATATTGTTGCCTTACAGA

39718715|F|0-67:A>G-67:A>G	TGCAGTTGAATAAGTATGATTACGAGTTGGAAAACATTGTTTTTGATAAGAAACCACGCTTTCATTGAG
39716025|F|0-67:T>A-67:T>A	TGCAGCCTACGTCATGATATAAGAAACGTGATCATAATTTATTATACGATATAATAGTTAGTAGATATA
39726937|F|0-27:A>G-27:A>G	TGCAGAAACCCAATGGAGAAGAAGGAGAGAAAGGGAGAGGAGAGAGGATTTATGTTAC
39714731|F|0-59:T>C-59:T>C	TGCAGATAAATGGCTCGGAACAGACGTAATCTCAATCCTTGGTTCACTCGCTTCTCTACTAAGGGACAG
39713594|F|0-60:G>A-60:G>A	TGCAGAAATCACAAAGGTAGCTTTTAGAGATGGTTAGCAGTAACATATATTACTTAGCTAGTAAGTAGC
39726752|F|0-29:T>C-29:T>C	TGCAGTGGTACTTTTTAGGTTATTTCTGCTGGACCCATTCATGTAATTGTGTTCATGAAAGCTGAATTG
39729125|F|0-41:G>T-41:G>T	TGCAGGGACGGTGGCCTGGTGGAATCCACTCCACTTCCTCAGAAGATCATTTTTTCTTTAC
39722693|F|0-10:A>G-10:A>G	TGCAGCCGGAAGAGTCGAGGCGGCGGCAGAGAGGGGAGGAGCAGGGGATTTGGGCAAAGGATTTAGATT
39724571|F|0-21:A>T-21:A>T	TGCAGAAAAATATAATCAGTTATTTGAAGTTTGAACTCAACAAAATTATACCTTAC
39722739|F|0-33:T>C-33:T>C	TGCAGCCTGCACCGTTGTTCTATTGAAAGAAAATGCATCATTCTAATGCTTTCGTGCCTCTCTCTGTTT
39724604|F|0-33:A>C-33:A>C	TGCAGAAATCAAGATAACTCGCTTAGTGAGGGCAGAAACTTAC
39713604|F|0-33:T>G-33:T>G	TGCAGAAATCTGACTGATTCTCATATGGCTCATTCATTATTCATGTCAGTGTTCACGGTTGTCGGTTAC
39721909|F|0-37:G>A-37:G>A	TGCAGAGTCTACCTGCAAGCCACCCATTGCCGTTGCCGCTTTTTACGCACAGAACAGAAGCCATAGCTT
39726061|F|0-33:G>A-33:G>A	TGCAGCAGCTGCTAGAGTTGGAAAACACTTGTGGGCGATTGTTCAGAAATGTTTGTTACATCTTTTGCT
39719872|F|0-16:C>A-16:C>A	TGCAGCAAGACAGGTACGACTTCCTGTTCTGTTCTGATACCATATTAC
39716029|F|0-16:G>C-16:G>C	TGCAGCCTCAAAGGATGAAACTCGATAGGAGAAAGCTTCGAAGGGGGCTAATGCCACTAGACCAAACAA
39721970|F|0-66:C>T-66:C>T	TGCAGATAGTGAGTTTCTTTGAGTCATACTCAAAGTCAAATTAGCAGATTAGGTGTGAAAAATACTCGA
39724777|F|0-9:C>A-9:C>A	TGCAGATAACCCAAAAAAGATCAAGGTTTAC
39714038|F|0-67:C>A-67:C>A	TGCAGAATGCAGTTGCCATTCCCATTCAAACCGCATTCCTACAAGCTATAACTTCCTAGCTTCCTTCCC
39713600|F|0-20:T>C-20:T>C	TGCAGAAATCCACAGTTATCTCCTCAACAAAATAAGATATGTCACAGTTGTACAGAAGTAATGTTTCTT
39725219|F|0-6:G>A-6:G>A	TGCAGTGAATAGATTTTTCTTGGAATTGATTAGAGTTTGTTTGTATTAC
39718221|F|0-54:T>G-54:T>G	TGCAGTCTTTGAGAATGGCCCTGAAAAATAAAAGGTAATATTTCATTTGATCAATTTCTTTTTTTGTTT
39718169|F|0-30:A>G-30:A>G	TGCAGTCTCCGTTCTCCATATGAGTTCATGAATCTCTGGTTTATATATAGAATTGTTTGCAGATGGATG
39718723|F|0-10:A>G-10:A>G	TGCAGTTGAGAATTTGTCCAGCATAACTTAGTGGTTTGTCCCGTAAGAATAGTACATGCAAGCTGGTCC
39717186|F|0-15:C>A-15:C>A	TGCAGGCTATTATGCCCTAACTATACAAAAGAGAACAAGATGGAGTTTCAAATCATCTGTTCTTACATA
39717733|F|0-45:G>C-45:G>C	TGCAGTAAATTGAATTGATTTGAATGTGAATGTCTCTATTGAGTGGAACGTTTACTTTTCAGTTTTTTT
39723461|F|0-19:G>T-19:G>T	TGCAGGGATGGTGATTTTTGTGTAACCTCCATTTCCTTCTTCATACAAAATCAAAGGCAGCGAGAAAAC
39716586|F|0-62:G>T-62:G>T	TGCAGCTTCGTCCAGGTAAATTGATCCATTGATAGCCTTGCTTATTGCTGAATTTTCCTGGGGTTTTTT
39718661|F|0-29:G>A-29:G>A	TGCAGTTCGTGATCGTTGCTGAATACCATGTACATATTTCTTTATTTTCTAAACTCGTCTGAACTAACC

39713385|F|0-37:G>A-37:G>A	TGCAGAAAAAACTTCAAAAGAAAACCTCAAATTTCTCGAAAGAAGTTTTTCAGCTAAGAGAAAGAAAAA
39713910|F|0-28:C>G-28:C>G	TGCAGAAGGTTGTTGTTGTTGTTGCCGCCGCCACCGCCGCCGTTGTTTGACTGATGGGTCATCGCCGAT
39714443|F|0-28:A>T-28:A>T	TGCAGAGATAGGTAAGAAAATAAGTACCATTATGAATTTTGATCCACAGCATTTATAGTTTGTGGTTCT
39715641|F|0-50:T>C-50:T>C	TGCAGCAGTTGGGTGAGAGCGCTGATTCAGGATAACGTCTTTGAGGATAATGTCTTTGAACGATGTTTG
39713699|F|0-43:T>C-43:T>C	TGCAGAACCACTATTTGGACCAGCAGATCAGTAAGTGTTTTCTTAGACATTTCCTTTATGTTCGTTGTA
39716277|F|0-48:C>A-48:C>A	TGCAGCTAGGATTGAGCGGCCAGACAAAGACAACAAAGAGTAATAGTACTAATAAAGAGAGTTGGAGTA
39717988|F|0-56:G>T-56:G>T	TGCAGTATTTCCTTCATCCTACCTTTTCCTGTCCCGACATTGTAGATTCCGACTTTGCCAGGGGTTGCT
39715498|F|0-8:A>G-8:A>G	TGCAGCAGAGAAAGTCAATTCAGAGAGACACGATTCCAAAAGCTTGAACGACATTTAGTTACTTGTGCC
39715664|F|0-19:G>T-19:G>T	TGCAGCATAGCAGGCACTTGTAGGTAGCAGCTCTAAAATTTCTTTCAGTAAATCATAACTTGATAGGCA
39722901|F|0-20:C>T-20:C>T	TGCAGCTCCATTACTAGGCTCGAAAGGGTGTCGAGGTTGCACACCGGGAGAACCATTGAAGTGAAATTA
39723767|F|0-25:G>A-25:G>A	TGCAGTAATGTTCTAAATCTGAAACGACAATCATTGCTAGTTTATATATCAGCTGACACTTATCTGACC
39713976|F|0-23:T>C-23:T>C	TGCAGAATATACTATTTGGCATCTGTATTTGAAAATCACTCCCAAACCCATGACAAAACCACCATAGAC
39715441|F|0-8:T>C-8:T>C	TGCAGCACTCATGGATGTTTGCGATTTCCAGCCTCCATTCATGATTTCCAGCGATTTTGCAATGTGAGA
39720813|F|0-8:T>G-8:T>G	TGCAGTTATGCGGCTAGCTTTCAATTTTCACATTTAC
39718501|F|0-45:G>A-45:G>A	TGCAGTGTGGTGGACTCACCTTTGGAACAACTCATGACTTACCGGGATGTTTCAATAGGTACACTGCCT
39723547|F|0-58:A>C-58:A>C	TGCAGGTAAGTCTTTTGGTCAAGTACGCTAAAATTGTAGACATGTTACATTTTTTTTATAGCTTCCAA
39715165|F|0-56:C>T-56:C>T	TGCAGATTTAGCAAAAGATTACTCCCAAGATTTTCTCTCTGCAACAAAGCTTCTATCCAAGGAAGCCAT
39718253|F|0-57:A>G-57:A>G	TGCAGTGAAGTCTATAGTTTCTTTGTACTTTTTTCCCCTTTTTTTAGACTGAAAATTATTATTGTCTTT
39714835|F|0-5:A>T-5:A>T	TGCAGATATTCCATGCATTTCAGCAGCCTAATCTTTGTCATCACAAGATCTACAAAATTTCAATGAAGA
39724562|F|0-14:G>A-14:G>A	TGCAGAAAAAAGAAGAAAGATCCCACAAATTCTTAC
39717116|F|0-19:A>T-19:A>T	TGCAGGCCAGGACGGTAGTACTCGAAATTCACGACGTCGTCGTGGTTCGAATAGTGGGACTGAATCAAA
39726698|F|0-54:T>C-54:T>C	TGCAGTGAAAAAATGGTAAAACAACATTTTTTGTTTTTGTTTTTGTTGTTTTTTTTGGTTGAAATGCAC
39714228|F|0-7:T>C-7:T>C	TGCAGACTTTTCGGTATTTGCAGACTCCTCCAACTTGGCTTTCATCATAAACATAACAACACTCAATAA
39715402|F|0-49:G>C-49:G>C	TGCAGCAATTTTTCGGAAAAGTGCCTGCAAAGGGAAGGGTTGGCCATTAGAAAGACCGTAAAAGAATAC
39724625|F|0-42:T>C-42:T>C	TGCAGAACCCAACTTGGATGGAGGACTGACATTTGACACATTTAGGTCACGACTTAC
46754244|F|0-22:T>A-22:T>A	TGCAGACTGAGCAATCCATATATCTCTCTCTCTGTAGCTTCTCTTCTTCAAAGATAAACCCAAAAAAAA
39715532|F|0-36:A>G-36:A>G	TGCAGCAGCAACCGATGTCATTGCCTGAGACTCTTCAGCAGTCCATATATTTTTCAGAAGCTCATCCAT
39717315|F|0-24:A>G-24:A>G	TGCAGGGCGGCGCTGCCGATTCCCAAGAGAGTGAAGGATTTGATCGGACGGCTGACATTGCAAGAGAAG
39724881|F|0-33:C>A-33:C>A	TGCAGCAAGTCTTGCTCGTGTGAAGACTGCTTGCTACGTTCGTTTACCACTTTTAC

<table>
<tr><td>39729152|F|0-12:T>A-12:T>A</td><td>TGCAGCTTGAGCTGCTGGGTGAATATCCCTTGCTATTGAATCTGCTCCAGTTATCTCATCTATAGAAGA</td></tr>
<tr><td>39721411|F|0-56:C>T-56:C>T</td><td>TGCAGAAGGTAAGCTGCTATAATACACTGTTATATTGTTATTGTAAAGGAGAAAATCAGAAAACAAGTA</td></tr>
<tr><td>39719436|F|0-34:G>C-34:G>C</td><td>TGCAGAAGTTGGGAATTCATCTAATTCTGCTTGGGATTTGAGTCTTTGACGCAATCTTAC</td></tr>
<tr><td>39718927|F|0-64:G>A-64:G>A</td><td>TGCAGTTTGTCACTACCATGCATATGATTACAATTATTTGAAGCAAACATCTAAATTTATGACGGTGGG</td></tr>
<tr><td>39716505|F|0-6:T>A-6:T>A</td><td>TGCAGCTGTGCTGGCTGTGTGGGTTCCGTGTCCATGGATGTCTCGTGCGGATAACCAATCGTTGGCTAG</td></tr>
<tr><td>39721166|F|0-64:C>T-64:C>T</td><td>TGCAGAAACAGGAACATATAGATGGATGGCTCCAGAGGTGCTTTTTCTTTTTCTTTTTCTTTTTCTTTT</td></tr>
<tr><td>39725641|F|0-10:T>C-10:T>C</td><td>TGCAGAATTGTGTATGCTTTTGTCATTTTTGTTTTTGGTTTAGGGAAAGGGAATTAGAGGATACAGTTA</td></tr>
<tr><td>39720297|F|0-37:T>G-37:T>G</td><td>TGCAGGAATTCTCGGAACCTGGAGGCATCACTATCAGTACTCCCGTTTGTATTTGTTAC</td></tr>
<tr><td>39716516|F|0-23:G>A-23:G>A</td><td>TGCAGCTGTTGGTTGTGATTCCGGCGAGGGAGCAGTGTAGGCGCGGGCTAGTGCAATTTGGGTTGGTTT</td></tr>
<tr><td>39718194|F|0-41:A>C-41:A>C</td><td>TGCAGTCTGTGACTTTGAGTGATACATCTTCTTTGGTGGTGATGAAATCATAAATTCTGTCCTTCTGCA</td></tr>
<tr><td>39718222|F|0-68:T>C-68:T>C</td><td>TGCAGTCTTTGCTTCAGAAAAAAAAATTCCCACTTTTTTTGTGCAATGATAGGATGGATACCTACGTCT</td></tr>
<tr><td>39723795|F|0-13:G>T-13:G>T</td><td>TGCAGTACGTGAGGTCTCTCGTAAAAACACATTATTTGGAATTACTTGAACAAACCATGCACCACAAGC</td></tr>
<tr><td>39725100|F|0-22:A>G-22:A>G</td><td>TGCAGGCACGACAACGTGTAACAAAAAAACACTTAC</td></tr>
<tr><td>39713571|F|0-8:G>C-8:G>C</td><td>TGCAGAAAGTGGAAGAACAAACAGCGCCCACAAACCCTAAGCCGATGTGAAGGACAGAGAGGAGGGGAC</td></tr>
<tr><td>39723091|F|0-37:C>T-37:C>T</td><td>TGCAGCTTTTGCAGAGGAAGCTCAGCTAATGCCATCACATGGCAGGGAGATGCAAAAAACTTTGGCAAA</td></tr>
<tr><td>39725674|F|0-10:G>A-10:G>A</td><td>TGCAGACGCTGCCAATTATTCGAGCTTGTTCATCACCAACTTTGTGTCTGAAATGTCCTCACAAACAAA</td></tr>
<tr><td>39720023|F|0-9:T>A-9:T>A</td><td>TGCAGCCACTCGTGGTGGATTTATTAC</td></tr>
<tr><td>39724730|F|0-10:A>G-10:A>G</td><td>TGCAGAGAGAAAAAACCGATGGAGAGAAGGCAATGGATTCACGGAAATATTTACCTTAC</td></tr>
<tr><td>39721087|F|0-58:C>A-58:C>A</td><td>TGCAGGTGAGGACTGGATATTGCAGTTTTTAGATAGTGCTGGTATTTTTTACCCTCTACGATACGTCTA</td></tr>
<tr><td>39717402|F|0-15:C>T-15:C>T</td><td>TGCAGGGTTGAGGTGCCGAATCGATGTTGGAGTTGTCAATGAAGAAAATGGACAGGATCATTACTTCGC</td></tr>
<tr><td>39720252|F|0-5:C>T-5:C>T</td><td>TGCAGCTTTGGGTAATCAGACGACAGATTTGGATATATGAATATATGAGTTTAC</td></tr>
<tr><td>39721951|F|0-64:G>A-64:G>A</td><td>TGCAGATACACTCCCAGCATGCAGATACATCCCCATCAGAAGGCCCAAGTCGAGGCCGAAGATGGCGTT</td></tr>
<tr><td>39724875|F|0-28:T>C-28:T>C</td><td>TGCAGCAACGAAATGGCCAATCAGAAATTATCTGCAATCTTCTCGCTAAGATAATTGTTAC</td></tr>
<tr><td>39715992|F|0-48:T>C-48:T>C</td><td>TGCAGCCGCCTGAACTAATTCAAAAGTTTTCGCCAGCATTTCCCAAATTTTACCACTATAAGAACAGCG</td></tr>
<tr><td>39726941|F|0-29:T>C-29:T>C</td><td>TGCAGAAATAGCTTTTCATTAGAGGATACTTGTTTCATTTTAC</td></tr>
<tr><td>39713257|F|0-51:G>C-51:G>C</td><td>TGCAGAAACGGATCGAACGACAGAAGAAAACAGAGTCAGTTCTCATTCTCTGAAGACAATAACAAAGGG</td></tr>
<tr><td>39717205|F|0-19:C>T-19:C>T</td><td>TGCAGGCTGGTATAGAGCCCGCTTTTGTCCTGTTTTTCGATTGTCCTGAAGAGGAGATGGAGAGGCGCA</td></tr>
<tr><td>39714706|F|0-44:C>T-44:C>T</td><td>TGCAGAGTTGCAGCCCATTATGGGCTGCAAATTCACGTTGATAGCGCGTGAAATCTGACTTTTTTTTT</td></tr>
<tr><td>39724928|F|0-17:G>C-17:G>C</td><td>TGCAGCATATTTGCCCCGATTCGGTACTTATACAAGAAACCAAGAAGACCTCCTTTAC</td></tr>
</table>

<table>
<tr><td>39714787|F|0-54:T>C-54:T>C</td><td>TGCAGATAGACTAGACCTGCACTGACTGCGTTTTGGACATGAATCCGAAAGGTATGAATGCCTGATTGT</td></tr>
<tr><td>39713938|F|0-40:C>T-40:C>T</td><td>TGCAGAAGTCTTAGACATATTAGGCCATCATGTATCAAAACCATAGAACAAAAAAAGGGAAACTCGTAA</td></tr>
<tr><td>39716264|F|0-33:C>T-33:C>T</td><td>TGCAGCTACTGAATCTTTGGCACCATCAGCTACCGTAGTTGTGCAACACGGTTTTTCTCCAAGGAAGA</td></tr>
<tr><td>39716456|F|0-46:G>A-46:G>A</td><td>TGCAGCTGCTTTACTTCGTTATTTTCTATATTCATGCATGTGATGGCACACTTTTGCACCCATGATTT</td></tr>
<tr><td>39720744|F|0-23:A>G-23:A>G</td><td>TGCAGTGGAAAAGTATCGGAGGCAGAGGAAATGTTAC</td></tr>
<tr><td>39727454|F|0-20:A>G-20:A>G</td><td>TGCAGAGGTATGTACACATGATCTTATACCCCTTTTGTAATTTCATACATCAACGAAATTGTTTCTTAT</td></tr>
<tr><td>39719508|F|0-6:C>T-6:C>T</td><td>TGCAGACATACAAAGACACCAAGCTCTTATATAAGATATGCGTACCTTAC</td></tr>
<tr><td>39722934|F|0-35:G>C-35:G>C</td><td>TGCAGCTGAACTCCAAGCCGACGATCACTAGCAATGGCAAAGCAATTCTTCCCCACCATCGCAACTACG</td></tr>
<tr><td>39723929|F|0-29:T>C-29:T>C</td><td>TGCAGTCATGGAATGTCAACATAACATACTGTGGATGATTCTTCGACAGAAAGATCGAGATAATACAAA</td></tr>
<tr><td>39717670|F|0-16:T>C-16:T>C</td><td>TGCAGGTTTCATTTTCTTTTCGCCCTCTGCTAGAATAAAATGTTTCTGAATTTATTGTTGGCAGGCAGC</td></tr>
<tr><td>39713378|F|0-66:C>G-66:C>G</td><td>TGCAGAACTGCTGATCGAGTTTCTCAAGATTCAAACTCAAAAAACTGGAAGACGAGACTTGAATGTCCA</td></tr>
<tr><td>39718509|F|0-39:A>G-39:A>G</td><td>TGCAGTGTTCGTCTGCTGCAAGAAGAGAAAAAAACAACAAATCAATAAATCAATTCAGGCTCTAGCGGG</td></tr>
<tr><td>39726521|F|0-15:G>T-15:G>T</td><td>TGCAGGTCAAAAATAGGACCTCCCAGTTTTCAATATCCTTTGTTCAAAGAATGCACTGTTGGGGAGAAA</td></tr>
<tr><td>39716298|F|0-26:G>C-26:G>C</td><td>TGCAGCTATTGTAGCAAATGAGCTTCGTAGTTGGTTTTCCAAGATATAATGGTTTGTTAGGAATAGTT</td></tr>
<tr><td>39716344|F|0-30:A>T-30:A>T</td><td>TGCAGCTCCTGAAAACAAAAGTGAATCAGCACCTGCTGCGGTGTCGAAAACTGATGGAGAGGTTGCTGT</td></tr>
<tr><td>39717712|F|0-42:T>C-42:T>C</td><td>TGCAGTAAACCATATAATTCAGCCCATTCTTTTATTTCATCATCATCAAGGTTTCTACGCAAAAGATTT</td></tr>
<tr><td>39728688|F|0-62:G>C-62:G>C</td><td>TGCAGCGAGCATATCCTAAAAACATTTCAAAATCAGAACAATCACCAGTGAGATTTTTATATGTTACAG</td></tr>
<tr><td>39713471|F|0-35:G>A-35:G>A</td><td>TGCAGAAAATTCTAAAGTGTTTCATGAGCATCATAGTAATTTGTAGGAAAGAGATTTTTCTTCAAATTC</td></tr>
<tr><td>39724813|F|0-27:A>G-27:A>G</td><td>TGCAGATCCTGTACTGCCATGGTTGTGAATTGTGTTAC</td></tr>
<tr><td>39722497|F|0-60:A>C-60:A>C</td><td>TGCAGCATAAGTTTGACCATTCTCTTTGAATTTTCAAAATGCTCTCTTCACCACAGTATGAGCTCCCAC</td></tr>
<tr><td>39719476|F|0-24:G>T-24:G>T</td><td>TGCAGAATTATCTATTGGGTGTGTGCTGTTAC</td></tr>
<tr><td>39716884|F|0-55:G>T-55:G>T</td><td>TGCAGGAGACGGTAGCAACTGTAAGCCATGGCATATGCAAAATGGTACCTGAAAAGGCCAGTGCCCCTC</td></tr>
<tr><td>39725578|F|0-15:C>T-15:C>T</td><td>TGCAGAAGACTGCGACAACTTCTCTAATTGGTGTAAGTTCAAGCGCGTAGTATCCGTATAAATGGAATA</td></tr>
<tr><td>39726442|F|0-41:G>A-41:G>A</td><td>TGCAGGCGGCTGATAGAGGAGGTGAAGGTGGAGATGAGAAAGATGACCGGCGGAATAGGGTTTGGTACT</td></tr>
<tr><td>39716904|F|0-24:T>C-24:T>C</td><td>TGCAGGAGCATTTGTTGGGGCAAATTATTCAAATCTGTTTTTGGTATTAGTAAACCTCAAGTTTATGCT</td></tr>
<tr><td>39720462|F|0-41:T>G-41:T>G</td><td>TGCAGGGTGGTGATTAGAATCTTTGACCTTTTGGTCAAGAATATATACTTAC</td></tr>
<tr><td>39722573|F|0-58:A>G-58:A>G</td><td>TGCAGCATTTTGAAACGATAATTTTCATTTTAGATCTGTCTATTTTTGTTTGGACAATATTTGGCTGCA</td></tr>
<tr><td>39722981|F|0-8:G>C-8:G>C</td><td>TGCAGCTGGCTGACATAGCCATTGGTTACTTTGGAAATATAACTCCACAAGAAGAATATTCCACATCAA</td></tr>
<tr><td>39721416|F|0-10:T>C-10:T>C</td><td>TGCAGAAGGTTCATTGCTCTTTCGGTTTCTATTTGCATACACGTGCTTCGTATTTTCGTTCTGATCTG</td></tr>
</table>

39721993|F|0-44:T>A-44:T>A	TGCAGATCAAGAGGTCACCGGTTCGAACCCGGTTGGGCCCTCTTTTTTCATCTTTTTTTTAC
39727728|F|0-54:T>G-54:T>G	TGCAGGGCATCCCCAAGATAAATCAAACACATCTAATCAAAACAGAGAAGCGTCTCCTTTCAAGAAGTT
39716384|F|0-6:T>A-6:T>A	TGCAGCTCTTGGCTCGAACGACAGGCCATTTCGACTTCCCTACGCCATACCGTATTGAACCCACCTGCG
39724350|F|0-31:C>T-31:C>T	TGCAGTTGGACGACACAATAATTTCTTCACTCAGTGCAGTTATTGGATGAAATCATTTTTGTAGGTTTG
39715775|F|0-66:T>G-66:T>G	TGCAGCATTGCAATATAACCTTACTCTAGGCAGGAAGGACCTAAGCAAGCGCTCTCAACTCGAAATTTT
39723392|F|0-58:C>T-58:C>T	TGCAGGCTCCAACATGCTCCCAGTATCCTCATACATTTGCTGTCCACGTATCATCAGACGCAGATGTGC
39714110|F|0-64:C>T-64:C>T	TGCAGAATTTTGTAATCTTTTTTGGTACAAAATTTTATTTTGTGTATGAAAATTCAGATTTCTACTCTA
39722139|F|0-66:T>C-66:T>C	TGCAGATTAGAATGTTTCTTTCTCATATCATCATATCTAAAAATACTGTTGGACTGCCATAGCGGCTCT
39726481|F|0-34:C>T-34:C>T	TGCAGGGGTCATGCAGCAAACTTTGCAACAGAATCCCAGTTGAACAGAGACTGATCAGATTACCACAAA
39713670|F|0-53:A>G-53:A>G	TGCAGAACAGCACGCTTTGGTTATCTCGGGTTCAGCCATACATACATACATAAATTATCATTATGTTTG
39717436|F|0-41:A>G-41:A>G	TGCAGGTAATACAATTCACGCTAGGATATTCACAAATAGGGAAAAAATAGTGATAAAAGAAGGAACATA
39720281|F|0-19:A>T-19:A>T	TGCAGGAACCAAAATTCCAAATCCATGAATCACTATCATTTGTTAC
39721538|F|0-25:A>T-25:A>T	TGCAGAATTTTAGTTTACAGTACAAACTTTTCACGAGCACGTGTTAGGATTTGGGCAGTCTAGATGACA
39718459|F|0-37:A>T-37:A>T	TGCAGTGGTTATACTTTTCAAAAAAGATATGGATCTTATATAAGGCAATAACATTCTATCATGTTTACA
39722048|F|0-67:G>A-67:G>A	TGCAGATGAAAGTATACTCATTTATTATGTTCAGAGTGAATAGATGGAGAAATAAGCGGTAAAAGGAGG
39721555|F|0-5:A>G-5:A>G	TGCAGACAACTAAGATGAACTTCAAGGCTATAAAGATCCTTCCAGAAGACTTCCAAGAGATACTCCTAG
39715486|F|0-34:A>G-34:A>G	TGCAGCAGAAGTTTAGAATCAATAACAAAACCCCAACTTGTAAAAATGATAAAAAGACATTTCTATGTA
39715927|F|0-17:C>T-17:C>T	TGCAGCCCACATTCGAGCACACACACCAGAGGAAAGATACATTCTGCAATCGAATTAGTTTTTGCTGCA
39720868|F|0-12:A>T-12:A>T	TGCAGTTGTGACATACAGTAAATTTATACTTCATAATTGGTTAGATTGGTTGAATTCTTAC
39721823|F|0-26:A>G-26:A>G	TGCAGAGCTGTTCTGCCTCACCGAGGAATGCATTTTTAGTTGATTTATTACTTTGAACAGTTCGTTGAA
39724925|F|0-46:C>T-46:C>T	TGCAGCATAAGCCTTCTTCGGTAAGATGAGATGAGGGGTACACCATCGATTTAC
46760754|F|0-25:T>A-25:T>A	TGCAGACTACGTAAGCTGATTGCTTTTCCTTTTTTTTTTAC
39718867|F|0-51:G>A-51:G>A	TGCAGTTTCATGCAAAATGGCAACAAATATAAAAGGTACAATCAAGAATGAGTCTTGTGCAAGTTGGGG
39718906|F|0-56:T>A-56:T>A	TGCAGTTTGATTTCAATACAATCGATGCTGCTACTGACAATTTCTCAGAGGAAAACTGGTTGGGTGAAG
39722467|F|0-14:G>T-14:G>T	TGCAGCAGGCGAGCGAATACCTTAGCCCACTGTATAGCTCGATTAGGAGCTCATTCCCTTGCGATTTTG
39716402|F|0-47:A>G-47:A>G	TGCAGCTGAGGTACTGGTGCTGAAATTCCTAGCAAGTTGTACTCTTGATTGATATCTAAATTTTATGGT
39717568|F|0-37:C>T-37:C>T	TGCAGGTGGTTCGATAGGATTTTGGTAGGTTGGGCGACAGCTTAGAGAGAGAGGAATGATGTGAGAATT
39715116|F|0-60:G>T-60:G>T	TGCAGATTCTCGTAAGTCTCTGTCTCTATTCTTTTGTATTTAGGGTTGTTTATTTGAATTGTGTGGTGT
39714467|F|0-51:C>A-51:C>A	TGCAGAGATTCCATAGCGATGATCTCATTCATAACTACACAAACCATCACCCCAAATAATATATTCAAA

39718708|F|0-13:C>A-13:C>A	TGCAGTTGAACCGCGCCGGTGGCTGGGCGACGGCTACGGCGGCGATGGCGGAGAAAACGAGGACTCTGA
39721531|F|0-13:T>C-13:T>C	TGCAGAATTTAGATGAAATCGAATTTGGGTTTATTCAGCGAAGGAAGACTTCGGCTTGCAGTTGCATCA
39727191|F|0-22:A>G-22:A>G	TGCAGTAAATAGCCAAATTGGAAGCAGTAAGATGTGATTCACAATTCAACAGGAATTAC
39717727|F|0-26:G>A-26:G>A	TGCAGTAAATCTTCTTCCTCCCTTCTGCTTTGAATCCCATTCCAGACTTGAAGTAGAAAAAAGTTTACA
39712843|F|0-32:C>G-32:C>G	TGCAGCCGTGCTCGTGGATTGTGTTTGTGTATCTTCCAATTCCTTGGAATGGTGTAATGTATTGGCATG
39715740|F|0-35:G>A-35:G>A	TGCAGCATGGATCCTCCAACTCTTCACTATTTGATGTTTTTCTCCAACAAAATACTTCAACCATATATT
39726692|F|0-40:C>T-40:C>T	TGCAGTCTGTATCTGCTCCTTGAGGTTACGAATTTCATCCCCATATTGTTTTTGTATTTGTTATGCTCT
39718081|F|0-27:C>G-27:C>G	TGCAGTCCACACCATTCCACTTGCTTGCTTTTCTGTTACCTCATATATATGTGTTTTGGTATTATACTC
39713298|F|0-35:G>A-35:G>A	TGCAGTGTATGCGAGCAGAAGAGCCTGGAAGAAATGGGCAAAGATCGGGTTCGAAATTCGAGGCGAATA
39723075|F|0-32:T>C-32:T>C	TGCAGCTTTCTTCGAACCCTTTCCACCGCCACTGCCTTCATCGACAAACTTCTGATTTCCATGTACCTC
39718756|F|0-42:A>G-42:A>G	TGCAGTTGCGACTATATGAATGCACAAAAAAAGTAAACATATAATCAGTTTGAAAGGCAGAAAACACAG
39715950|F|0-67:A>G-67:A>G	TGCAGCCCTCAAAACTTCACGCCGTTGTCTGGTCGCAGATCGACGCCGCCGCAAGCCTTCTCCAGCAAG
39724424|F|0-44:C>A-44:C>A	TGCAGTTTCTCGCACAAATCTTTACTCAAATCATTCTCTGAAGTCATTTTCTGGACGTTTTATCTTTTG
39713837|F|0-17:T>A-17:T>A	TGCAGAAGCCAATAAACTAAAGACGAGACTTGACTCTAAGCAGTGATGTGCTTCACAAAAGGAAAATGA
39717958|F|0-65:G>A-65:G>A	TGCAGTATGTTGCTTAGTGAACTCATTTTTAGTTCGAAACTGTTCCAGATGTCTTTCCTTCCATGGTTG
39717561|F|0-37:C>A-37:C>A	TGCAGGTGGGTAACTGGCAACCAAGTTCCTGTCGTAACGAAAAGGCAAAGCCATCATGTAATATTAGGC
39728026|F|0-45:T>A-45:T>A	TGCAGCCTGCAAGAATAAATAATCAGTTGTTGATAACAGTAATATTACCAAATTTTAC
39715948|F|0-9:G>A-9:G>A	TGCAGCCCTGCCGCCTAGTCCACCAACAATTCTAGCAAAGAGTTGCTGCTTGGAGATCAGGGTTGGATC
39717266|F|0-22:T>A-22:T>A	TGCAGGGACATTCAATTGCCAATCAGAAAACCACATATAAGCCACAGTTTTTATTCTTTGAGCCGAAAT
39718834|F|0-48:G>C-48:G>C	TGCAGTTTACTTTCTTGGTTTCCAAACAGTAATGATCCAACACACAACGTTCTAATCATCTCTCTCTCT
39719864|F|0-33:T>A-33:T>A	TGCAGCAACGGATCAAACAACTTATATATATATATTTTTTTATTTAC
39713346|F|0-49:C>T-49:C>T	TGCAGCTTTTCTCGAGGAGTTTGGGTTGCTCAATCGAGAGTCGTTTGCTCGATGGTCAAGGAGTTCCTT
39715202|F|0-24:G>C-24:G>C	TGCAGATTTTCAATTTTGTACTTAGTATTCTCCATGTTACGAATTTCAATATTGCATCACACACTAATT
39726045|F|0-13:G>T-13:G>T	TGCAGCAGCAGCAGCATCTTCATTCTTTCCTCTATCCCAATTGTCACCACAAAATCAAAACTTCTCAAT
46753863|F|0-58:A>T-58:A>T	TGCAGAAGAAGCCATACGTATAAATAAACTTTTCAATCATCTTTAGTCCAGTTGGAGTAAGGTGGACAT
39717229|F|0-64:C>T-64:C>T	TGCAGGCTTGCGACCAACAGCGGCAGAGGCTACTGTTGTAAGTTGGGTAAGTTCAATAATTATTCGTTA
39720224|F|0-31:T>C-31:T>C	TGCAGCTTCCGTCACTTGACTCCACCATCATTGTATGGGTCGTTAC
39718923|F|0-39:T>C-39:T>C	TGCAGTTTGGTTTTTATCTCTTTCATTTTTTACTTCTTCTTCTTCATCATCATCATCATCATCTCT
39715657|F|0-5:C>G-5:C>G	TGCAGCATAATGATCTACATGACAAAAGCTTATTCATTTACATCAAATAATGAAGCTTGGTTGACAAGC

39716288|F|0-21:C>T-21:C>T	TGCAGCTATCTGCTTTCTAATCGTTATTCCCAAAATCTAATAATTGAAGGATCTCTTTCGATCATGCAA
39717599|F|0-8:T>A-8:T>A	TGCAGGTTTCATGGCATCATATTTCTTTGTACCTTATATCAATTCAAATGGTAAAAGCAATTTTTGGTC
39713630|F|0-27:G>T-27:G>T	TGCAGAAATTGATGAAGTTGAAACCCAGAAAGAATCTCTTCTCACTTAGTATGATATGATTGTTTGTTA
39719317|F|0-25:A>T-25:A>T	TGCAGAAAGGCAACAAAATAATAATAATTATTATTTAC
39722013|F|0-59:G>A-59:G>A	TGCAGATCCACACCAAAAAAAAGGGTATTACATTACTAACTTTGTTATTTCTGAGAAACGAATTTTACT
39725026|F|0-24:T>A-24:T>A	TGCAGCTGTTGGTGAGGCTGCATCTGGTATTATATTTTCCTCTTATGTTTAC
39717369|F|0-29:G>A-29:G>A	TGCAGGGTAATGTGGGAAAAATTTCCAATGGTATAGAGTATGAAAAATTTTCCATACTACCTACCTTCA
39715616|F|0-65:G>A-65:G>A	TGCAGCAGTACCAGGAAGTTCCCTTCTGCACGAGCGATTTTTTCTAATAGAAAATAAAAGAAAATGTTA
39715251|F|0-30:G>C-30:G>C	TGCAGCAAAGAAATTAGCAACACAAATTTGGTCGTAGCGAATTCATGGTAAGGTATCATGAGAGTGTTC
39723369|F|0-63:A>C-63:A>C	TGCAGGCGCCTGAGGTCATGGCCTATATAATCAGCAACTCGAGATAACAGTGTTCTAATTCAAAGTTAC
39729562|F|0-32:T>G-32:T>G	TGCAGAAGAATTCGAGCGCCAGATCTCTTCGGTCTTGTTCAACATCATCTGGGTACTCATTAC
39716736|F|0-14:A>G-14:A>G	TGCAGGAAATAATGACAACAGTACTAGAACTGTTATAATTGTTGTGGTGTCCATCGTTTCAGCCATCAT
39717660|F|0-45:T>A-45:T>A	TGCAGGTTGTTTCCCTCTGAAGATAGTCTGCTACCATTTTCTACTTGTTGTAGCTTGCAAATTCTAGTG
39716433|F|0-28:G>C-28:G>C	TGCAGCTGCCTATTCACTATCCTCCTACGAGTCTACGACCGTGACAGTTGGGCTCCTCCCATGTTGTTC
39728857|F|0-63:A>C-63:A>C	TGCAGCAACTACAAACAAGCCAAAACATAATACTTCAGAACCCAATAAATAAACAAAAAACTTAC
39728697|F|0-58:C>T-58:C>T	TGCAGAAAAGCGGCAGCTGAACCACAAACGCCAATTTTGCATCGAAAACATTTTTTTTCTTTTTTTTTT
39713481|F|0-48:T>C-48:T>C	TGCAGAAACAACATGTACCTGGCCTCAAGGGAGAAGTGGAAAGTCAACTTCACAATAAAGAAAATATTC
39714726|F|0-52:A>C-52:A>C	TGCAGATAAAAGACAGCTAATATGTCATGGTTTGAAAAGAAATTGAGTCCATATTTTGAATAGACCTAA
39717166|F|0-64:T>C-64:T>C	TGCAGGCGGACTCCATGGTACAGGCATCAGACCAAAACAGGAAACGCTTTATACAACTGTAGAGTAAGT
39717277|F|0-24:G>A-24:G>A	TGCAGGGAGCTTGAGGCCCTTGAGGAGTGAAAGAAGAACGCAAGGTGGGGCCTTGAAGAACAGGCAGAG
39713570|F|0-46:C>G-46:C>G	TGCAGAAAGTCAAATTTTGGCAAGACAGGAAAAAAGTGCAATTTGGCTCGTGATAAACTGAAGGGGTGT
39716690|F|0-48:G>A-48:G>A	TGCAGCTTTTGGATATAATCCATTTGAAAATTATTCGACGTGTCGTTTGCCATGTCGATATCGAAATCT
39717593|F|0-67:A>G-67:A>G	TGCAGGTTAGAAGGAGAAATATATATATTAGAATGATGTACAATTTGGATGACATGTGGATTTCTTGAA
39721942|F|0-43:T>C-43:T>C	TGCAGATAATACCGTTGAGCATCAAGATCAAGCACTTCGGTTTTTTTCTTTTGAATGAAGATTGAGGAC
39714984|F|0-47:T>C-47:T>C	TGCAGATGCAAGTTCTGCAATGAGGCTAGAAACCTGCAATCATAAATTGTCGGCCCCAGTTTAC
39724162|F|0-61:A>G-61:A>G	TGCAGTGTCTATCTATTTCAGGAAACACATCCTTCAAGCCAATCTATCTAATTTTGAAAATAACCAAAA
39728125|F|0-64:T>A-64:T>A	TGCAGAAAACAATGGTGTGAAGAACATATGAGAAGAACAATCTTAGAAGTTATATTCACCAACTTACAG
39720135|F|0-27:T>C-27:T>C	TGCAGCTAGTGCGAGAACCGCCACACATACCGTTAC
39720644|F|0-41:G>A-41:G>A	TGCAGTCAACTCCGGTGGGATTTTGATTGAGCTCGCCGGTGGATTTCTCCGGCGGGTTTAC

39715145|F|0-37:G>T-37:G>T	TGCAGATTGGTGGTTTTTGTTTGTGGGAGTGGCAAATGGGTCAAATTTGGTTGATTTCGTATTGATTTT
39728136|F|0-45:C>T-45:C>T	TGCAGAAATTTTTTCAATGCCACTCTCTTTGATCGATCCCCTGAGCCACTAGTATTCCAAGAAATAATC
39716654|F|0-20:C>T-20:C>T	TGCAGCTTTCATCTTCACCACCGCTTCTTCTTCTTCTTCTCCCCCTTTTCATTTCCCTCAATCCTTCAA
39713717|F|0-67:C>A-67:C>A	TGCAGAACGACAATGTCCCACAATTTAGAAAGTCAAAACCCATGAAAGTGGTGGTTGCTGATTGCCTCT
39721103|F|0-64:A>G-64:A>G	TGCAGAAAAAGAAATGGGTGTGTCTGTGATTTTTATAGAGAGGAGAGAGAAAGAAGAGAGAGAAAGAAG
39724638|F|0-23:T>C-23:T>C	TGCAGAAGACCTGGCCCTTGCAATGTTAC
39716557|F|0-25:T>A-25:T>A	TGCAGCTTCCAAAATTATATAATAATATGGCCTTTTGGGTCCATGCAAGATGGTTTTGTGTTTTATTTT
39727500|F|0-47:C>T-47:C>T	TGCAGATTATAAGAGCAAATTGTGCATCATAAATCTACTATCCAGAGCTCAAAACCATAGCAAGGAAAC
39715460|F|0-44:G>C-44:G>C	TGCAGCACTCAGATAAAAGTAATTCCAACATTTATACAAGACAAGATAACCGTTCTATTCAAAGATAAA
39716778|F|0-52:C>T-52:C>T	TGCAGGAAGAGGAAATTGACAAGTCTAACCAGAAACTGAAGAATGCTCTTTTCTGGTTAGACTTGCAAA
39718076|F|0-65:A>G-65:A>G	TGCAGTCCAACAAAATTGCAATTTCCTACACTCTGTTCTCCTGCTGATTATTTCATATCAACACTAATT
39718187|F|0-51:C>G-51:C>G	TGCAGTCTGCACTCACAAATGCCATATCCATATAATAAACCTTTTTTCTTTCTTACATTACACGTGGGT
39718260|F|0-47:T>C-47:T>C	TGCAGTGAATCGACGTTACGTTGTTCGGTTTCGTACCCTTATTTTCCTATCGAGGCAGCCACAACAGCA
39721676|F|0-11:G>A-11:G>A	TGCAGACTTCTGGCCTTCGGAGACGTGGGGCAGAGAGGCAAAAGCAAAGCTCTGTATGGAAATATGAAG
39727501|F|0-64:G>A-64:G>A	TGCAGGTTATAAGAGCAAATTGTGCATCATCAATCTACTATCCAGAACTCAAAACCATAGCAAGGAAAC
39721125|F|0-63:C>A-63:C>A	TGCAGAAAACAGATTTCATAGCGTCTCTGACAATCCGAATAAAGGATTTGTCATACCAGTAGGCAGAAA
39714059|F|0-62:G>T-62:G>T	TGCAGAATGTTTTTTTATCCCAAAATGTACATATAATTTGTGAGCTGCCTATTACAAGATCAGAATTTT
39715768|F|0-25:T>C-25:T>C	TGCAGCATTCTCTAAATAACCAGCTTCAGTTTGTTATAAACAGGGTGCGAATCTGGTGAAATGGGATTG
39720822|F|0-20:G>T-20:G>T	TGCAGTTCAACAGCCGCCATGTTTAC
39717914|F|0-34:T>C-34:T>C	TGCAGTAGTCACCCGCAAGGTAGCTTTCCGGCGTTGATTCAGCGACTGTGCGTGAGTACCGGCGTGTAC
39725000|F|0-12:A>G-12:A>G	TGCAGCTATGGAAGCTTTAGATTTTGGTAGAACAATCCATGGACATGCACTTAC
39725357|F|0-39:G>A-39:G>A	TGCAGTTATGAAATCAGAGTAAAAAAACTATAGTTTATAGTTCCAAAAAGGATAATATTAC
39716762|F|0-53:G>C-53:G>C	TGCAGGAACTAAGGCACATTCCGAACAAGCTGAGAAGATGAATGATATACACAGAAATGAGACGACCAA
39726790|F|0-50:T>A-50:T>A	TGCAGTTCCAAATTCCAATATAACTATACGAAGTAATTCCAATAAAAATATACTTTTACGTGTAATTAT
39713679|F|0-12:T>C-12:T>C	TGCAGAACATGATGTTTGAAGGATGTGAAAGGTTTGGGACTTTCATTTGCCAGTCTCTGGCAAGGCAGT
39718335|F|0-15:T>A-15:T>A	TGCAGTGCAGAGAATTATGCGACTACACATTTAGTATCAATTCCATGTGAATAGTGAATAGAATGAATA
39722359|F|0-51:A>G-51:A>G	TGCAGCACCTCCCGCTGCCGGAGATAGACCGACGACCCCACCGCCGGCCCGAAATCTCCGCCGTCGCAG
39717890|F|0-13:A>G-13:A>G	TGCAGTAGCAGTAACAGTAGCAGTAGCAGCAGCAGCCCAGCCCATAAATGCAGCACCAAGCAACCAAGC
39729113|F|0-56:G>A-56:G>A	TGCAGTTGATTATAATCCATTAGTAGTTGCCATCATTGTCTGATAATTGGAAATGAGATTGGGTTACAG

<table>
<tr><td>39718283|F|0-65:G>A-65:G>A</td><td>TGCAGTGAGCGTCAGTAATCATGCAGTAGATGATGTGATTACTAGATTCCCTATTCTCAAATGCCGAGA</td></tr>
<tr><td>39716437|F|0-42:G>T-42:G>T</td><td>TGCAGCTGCCTTGTTGTTTGCCTTTGGGCTAAAGCCATAGTTGCCTTTTCTGGGTCTATCTCCTCGCAG</td></tr>
<tr><td>39723649|F|0-59:A>T-59:A>T</td><td>TGCAGGTGTTATCAACCAGTACTATCAAAAAGTAATTCAATCCATTTGCTTCAGTATTTATGCTCAATA</td></tr>
<tr><td>39716921|F|0-61:A>G-61:A>G</td><td>TGCAGGAGGATTGAATCCGGATTTCACTTGGTACCTGTTTATCACATTTATTTCTAATTTGAGCGTCCT</td></tr>
<tr><td>39716644|F|0-33:C>T-33:C>T</td><td>TGCAGCTTTACTTTCTTTTCTTTATGGAAATTTCGAGTGCAAGTCTGCACTGACATTTTCTTTTTATAG</td></tr>
<tr><td>39718085|F|0-49:T>A-49:T>A</td><td>TGCAGTCCATAGGGATCTGTGTATTTTGAGAAAGACCACGTCTTTTACTTTCCCTAAATCCCCACCACC</td></tr>
<tr><td>39718658|F|0-7:T>C-7:T>C</td><td>TGCAGTTTGTAATAGAAACTAGAAGAGCTAGAATAGTATTCAAACAAAAAAGGTAAAGAAGTACAATAA</td></tr>
<tr><td>39716246|F|0-17:T>C-17:T>C</td><td>TGCAGCTAATTCCTTCCTTGGTTTCGGTACCACGGGAGATCTTGCTACTCGTAAGAGGGAGCTAGCGGC</td></tr>
<tr><td>39718247|F|0-39:T>G-39:T>G</td><td>TGCAGTGAAGAACAGAACAATGAATCGCCATGGAAGAGGTACGGAAGACGAAGTTTGGAGTAGAACTAT</td></tr>
<tr><td>39717469|F|0-59:C>T-59:C>T</td><td>TGCAGGTATATATACTTCAATCCTATGATGGCAAATACATATGTACTTGTCCCTTACATCTTACTTACC</td></tr>
<tr><td>39721557|F|0-30:A>T-30:A>T</td><td>TGCAGACAAGCAACAAAACACCAAGTTGCAAACATGAAAAAACACTTGAATCAACTAACGGAGGATGAT</td></tr>
<tr><td>39714358|F|0-30:T>A-30:T>A</td><td>TGCAGAGAAGCATGGCACCTGCGCAGCTCCTGTAATCATAGGCGAATATGATTACTTCACCACAACCCT</td></tr>
<tr><td>39724659|F|0-23:A>T-23:A>T</td><td>TGCAGAATAATTGCGTTATTGATATGAGAAGGTAAGGCCAATGCCTCTATGGCTTAC</td></tr>
<tr><td>39718602|F|0-41:G>C-41:G>C</td><td>TGCAGTTCAAAGAATCTCAAGAAAAGGATGACAGTTTTTCTGCTTGTGCTCAAAAGGGTTATGCAGGTA</td></tr>
<tr><td>39726350|F|0-65:T>C-65:T>C</td><td>TGCAGCTTTTTTTGGGGTTCAGATGAGATTCTTGAACCTTCCATTTCTTATTAGCGGTACTGATTTTAC</td></tr>
<tr><td>39716008|F|0-36:T>G-36:T>G</td><td>TGCAGCCGGTGTGGTGACTCTTGTGGCATAATGTTTTCTCACAGGCGAATCGCTCTATTTTGCTGGAAG</td></tr>
<tr><td>39717915|F|0-47:G>A-47:G>A</td><td>TGCAGTAGTCAGATATGCCTCATTTTCATCAAGGGAAGACCTGTTGAGCATGAAACGTTATTTATTGGG</td></tr>
<tr><td>39718211|F|0-26:C>T-26:C>T</td><td>TGCAGTCTTCTCCCTTTCTCTTTTTTCCTTTTCAGAAAAGAAAACTAACGTCTGTTTCGGTTAC</td></tr>
<tr><td>39721787|F|0-57:T>G-57:T>G</td><td>TGCAGAGCAGAGAAGAACCACTGTAAGTTTTATGTCTCTGATAGAACTCTCTATTGATCGTTTTTGTTG</td></tr>
<tr><td>39713361|F|0-44:C>T-44:C>T</td><td>TGCAGTACTTTCTATCATCTTTTCATCTTTATTGAGATTTGTAGCTCGACTTGGGACTCTATTCTTCAA</td></tr>
<tr><td>39714035|F|0-66:G>A-66:G>A</td><td>TGCAGAATGATGGTCAAATAAACAAATCACTATCACACTTTTGAAGGTTGGTGTTTTTATATAACAGGT</td></tr>
<tr><td>39717814|F|0-63:C>A-63:C>A</td><td>TGCAGTACAACATATGATGGATAGAAAACAAGATCAGACAAAGATATGGCGCACCATAAGGAACAAGAA</td></tr>
<tr><td>39714569|F|0-51:T>G-51:T>G</td><td>TGCAGAGGATAATAACAAGTGAAAATGGTTTTCCTTATTGAAAAGGTTGCATAAAAGTGCTTTTGAAGA</td></tr>
<tr><td>39717844|F|0-65:T>C-65:T>C</td><td>TGCAGTACGACCTCTGTGTAAAGGTGTGGGGCTGGGTGCTGTAGTGTTGGATATTAGTGGAAGCATAGT</td></tr>
<tr><td>46755915|F|0-66:A>C-66:A>C</td><td>TGCAGCTGCATTGCCAATTTAGCAAATACCAAATAGGAATTTATAAATTATAATTGAGGAAAATTCATT</td></tr>
<tr><td>39713738|F|0-34:C>G-34:C>G</td><td>TGCAGAACTTCGACATCGGCTTCACGAGGGCCATCTCGGCGGCGGCGGCGGTGGCAGCCATGGAAGAGG</td></tr>
<tr><td>39715126|F|0-50:G>C-50:G>C</td><td>TGCAGATTGAATACCAAATGGGCTGTGATCTTGCTATTCCTAATCTTGCTGATGCACTGGTCAGCTCAT</td></tr>
<tr><td>39713435|F|0-58:T>A-58:T>A</td><td>TGCAGAAAAGGAAAACCCAGATTCTACGGGTGTGATTATAGGAATTCAAGAATCGGCCTCGGTTACGGA</td></tr>
<tr><td>39717729|F|0-15:T>G-15:T>G</td><td>TGCAGTAAATGTGAGTGCCTCACTCTTTCTCACTATCTTATTCCTCATGAGCATAACATGCTCCACATA</td></tr>
</table>

<table>
<tr><td>39725261|F|0-43:G>C-43:G>C</td><td>TGCAGTCAAATGCTCTACCACTGAGCTATGGACCCATTGTTGGGCTATTGTTAC</td></tr>
<tr><td>39714082|F|0-13:T>C-13:T>C</td><td>TGCAGAATTCTTTTTCTGAACATTTTTGCTAAGTTATGCTACTGCCAATCATTTCTTACTGTTTACGGT</td></tr>
<tr><td>39715484|F|0-22:C>T-22:C>T</td><td>TGCAGCAGAAGAACAAGATAGTCTCATCAACAATATGAACATGGGTAACAATCCTTTCCTTCATAGAAT</td></tr>
<tr><td>39717488|F|0-57:G>C-57:G>C</td><td>TGCAGGTCACATGTTTTGGGCTTTTGGGCTTGGAATCCCAATCCAATAAATGGCCCAGCCCAGCCCAGC</td></tr>
<tr><td>39718235|F|0-26:G>T-26:G>T</td><td>TGCAGTGAAAGTCTTTGATTTCTAAAGAATAGAAATCAAAAATTGTATTTGTATTGATTCCATTTAGAA</td></tr>
<tr><td>39720146|F|0-20:G>A-20:G>A</td><td>TGCAGCTATTTTATCACCTAGAATCTATGAATTATATATTAGTTCCCAATTAC</td></tr>
<tr><td>39721684|F|0-32:C>T-32:C>T</td><td>TGCAGACTTTTGTTTTGTTGGAGTTTCCCTTCCATTCTATAGGACAACAGTACTTGATAACTTTTAGAA</td></tr>
<tr><td>39718140|F|0-59:G>A-59:G>A</td><td>TGCAGTCGGTTTGTTTTGAATGTGCCGAAGGAAGATCTACAGTCATTTGAAAGGATTCTGTTCCTCATA</td></tr>
<tr><td>39720020|F|0-39:A>G-39:A>G</td><td>TGCAGCCACGTTGAATAAATTTTCTCCAAGTAAAGATAAAGGAAAGGAAGCATTCATTTAC</td></tr>
<tr><td>39719335|F|0-37:C>T-37:C>T</td><td>TGCAGAAATGTTGAAAAATATATATTATAGTTTCTAGCCCAATTAC</td></tr>
<tr><td>39721093|F|0-20:A>G-20:A>G</td><td>TGCAGAAAAAAATATAAATGAAAAAACATGGTGGTATTTCCATTAGTTCTAAAAAAATAATTAC</td></tr>
<tr><td>39721458|F|0-48:A>G-48:A>G</td><td>TGCAGAATAGCACCAACTGAGTGTCAAATTCCAAAGAGATTTTATTGTAGTCCAACTCTAAAGAAAATA</td></tr>
<tr><td>39722355|F|0-34:T>G-34:T>G</td><td>TGCAGCACCCCATTGGGGACTTTTCTCAGTAACCTGGCAGACCAAACAACGACAGTGTTATTGTGGCTA</td></tr>
<tr><td>39717005|F|0-11:G>C-11:G>C</td><td>TGCAGGATGTGGCATGAAAATCTGGAATTCTTTGAATTATATATTTTGAATGATTAGCATTTCCAGCCT</td></tr>
<tr><td>39716749|F|0-8:T>C-8:T>C</td><td>TGCAGGAATAAATGGCTAAGACTTTTCTCTTCTATCAGGCACGCACAGCAACCATTTGGGACTTTTGGA</td></tr>
<tr><td>39727187|F|0-22:T>A-22:T>A</td><td>TGCAGGTTTTTCTCCGGTGCTCTCCCCGTCGTCCGGCACCGGCGACAGGTCTTTTAC</td></tr>
<tr><td>46753970|F|0-52:G>A-52:G>A</td><td>TGCAGAAGTATATTTCTCTGATCTTTTTTCTCCCTTCTTGGGTCAAATGTCTGTATCCTTACCCATGTA</td></tr>
<tr><td>39715240|F|0-66:A>G-66:A>G</td><td>TGCAGCAAACAGAAACAAGTATTATTGAGAGATTTTCATGTCAAGAACTAGAACATGTAACCACTCAAG</td></tr>
<tr><td>39715245|F|0-22:T>G-22:T>G</td><td>TGCAGCAAACTACGGCCATGGATTGGCTTTCTTTCTAGCTCCAGCTGGGTTTCAAATCCCTCCCAACTC</td></tr>
<tr><td>39718028|F|0-51:T>C-51:T>C</td><td>TGCAGTCACATAATGGAGTTACTGTTGTCATAATAGATCAATATTTTTTATTACAAAATATCATTTCAT</td></tr>
<tr><td>39728420|F|0-44:A>C-44:A>C</td><td>TGCAGTTTTGACTTCTTTGAAGATTTCCTATTAGTTCGCCTCCTATTCTCTTTCCTTGCTTCATCCTTG</td></tr>
<tr><td>39717149|F|0-57:G>T-57:G>T</td><td>TGCAGGCCTTCCAAAATGAACCATTTTTCTGAGAGTATTTGAAGAGAGAGAGAGAGAGAAAAAGAAATC</td></tr>
<tr><td>39716175|F|0-9:T>G-9:T>G</td><td>TGCAGCGGCTCGTCCACGAGCACGATGGCCCCTGCGCGATTTCTTCTCCGGCGAAAAAAGATAGATCTG</td></tr>
<tr><td>39718272|F|0-47:T>C-47:T>C</td><td>TGCAGTGACTAACTTGTTGACATTTACTTCAGCAATCCTTCCTTCTTTGATAACTTTTACAATTGAATT</td></tr>
<tr><td>39725399|F|0-7:T>G-7:T>G</td><td>TGCAGTTTTTGATCCTGTCCCTGTCAGACTCATGGTTAC</td></tr>
<tr><td>39727248|F|0-41:A>G-41:A>G</td><td>TGCAGTGTCAACTGTGAACCATCCATAGGGACCATCAACCTATGTCAGATTAC</td></tr>
<tr><td>39728076|F|0-16:A>G-16:A>G</td><td>TGCAGGTTCCTCGCTTAGTCCGGATGCATTCTGATGAGATGGAGGTTGACTTCTTTAC</td></tr>
<tr><td>39714389|F|0-65:T>C-65:T>C</td><td>TGCAGAGACATGGAATATGGAAAGGATTCTATCGTTTTCTTTTTGCTAAAGCTGTGAATCAGTGTTATC</td></tr>
<tr><td>39715912|F|0-11:G>C-11:G>C</td><td>TGCAGCCATTGGAAAGTGCGATCCGGTGCAACCTGAATGCTCGAATCCTTGGGAGAATTTTCGTACGCC</td></tr>
</table>

39716338|F|0-49:C>A-49:C>A	TGCAGCTCCCTGCTTTGATTTGTCCCGGTGTAACATTGGTGATATCTCCCCTTGTGTCACTTATTCAAG
39723705|F|0-47:C>T-47:C>T	TGCAGGTTTTGGGCCGGCCCAACTTCTAACCTAAATGAGGCCCATTGCATACGGCTTATTTCCATTTTT
39725030|F|0-18:G>A-18:G>A	TGCAGCTTCCCTGTTGTCGCAAGGCCAGGCTGTGCAGCCTCAAATTAC
46756926|F|0-27:T>A-27:T>A	TGCAGTAACAATATAAAACAAAGACTTTGAAAAAAAAAAAATAGACTTTTAGAAAAGTAGTTAC
39721326|F|0-6:G>A-6:G>A	TGCAGAGGAAATGGCATTGTTTGTTTGTATGTGGTTTTTACCTAGAGAGACTGCACTGACGAATATTGT
39721439|F|0-48:T>C-48:T>C	TGCAGAAGTTCTATGGACAATGTACTGACAATGCTTCTTCGAGACTGGTTCAAATAAACAAGAGGAACC
39714138|F|0-20:G>A-20:G>A	TGCAGACACAAAATTTACCCGACAATCAATTCAGATTGACACAAACTGCCAAACTAATAGAAAGGGCTA
39718052|F|0-64:A>T-64:A>T	TGCAGTCAGTAGAAGAGATCTAAATTAGAGGCCTTCAACTTCAACTGACACCAACCAAAAAAGCAACAA
39716680|F|0-25:C>G-25:C>G	TGCAGCTTTGTGTACCACAAAATAGCCAACAAAAAGACTAGCCATGTTACAACACTTACGGTTCTAAAT
39719419|F|0-28:G>C-28:G>C	TGCAGAAGGCCTTGATACGGTAAGGAAAGAAATATATAGCCGTTAC
39716346|F|0-44:A>C-44:A>C	TGCAGCTCCTGCAACTTCAAGATCTATATCATAATGGACATCGCAAGGAGTAAAATTTAGCTCACACGA
39727021|F|0-9:C>T-9:C>T	TGCAGATACCCTGTAAAGAACAAACGTTCTTATCCCCATTTTTTTAC
39719636|F|0-16:C>A-16:C>A	TGCAGAGGTAACTTATCTTTTCCTCTGTGTTAC
39714171|F|0-48:T>G-48:T>G	TGCAGACATCACTTTATTGAATCAACCCATAGAATAAAATAATATACATAATTTCAAGTGACAAAGAAA
39714721|F|0-68:G>T-68:G>T	TGCAGAGTTTTGTTTCCCCTCCTTGATCTCATCATAGTTGAACAAGACATGGTCTTTGGTGTAATATTG
39722125|F|0-24:C>G-24:C>G	TGCAGATGTCTTCAAACCCACCCACCAACCCACCCCACTCGCTCTCTGGCAAAGGACAAATAAGTAATT
39722310|F|0-53:T>A-53:T>A	TGCAGCAATCGCCATAATCTCGAACGCTTGCAGCCCTGCACGGACCGTATACGTACGACACGATCAGTC
39715227|F|0-57:C>T-57:C>T	TGCAGCAAAACTACAACGGTATTCCATAAATCAATGGCCGTCCAGTTTAGATTATAGCGATACCATACA
39718020|F|0-30:C>T-30:C>T	TGCAGTCAATCAAACTAAGTGGTAGATATTCGATCTACTACATCTGTTTTTTTTTCCGCTCTATTTTTG
39717745|F|0-60:A>G-60:A>G	TGCAGTAACCGGCTTCTCACCTGTTTCCAAAAATTCAAGCCATTCAATACGATTATTGGAACTTGGTAA
39719535|F|0-10:A>G-10:A>G	TGCAGACTAGAATTTGTATGTAATTCTATTTCATGTGACAACTAGATTATTAC
39721536|F|0-36:G>T-36:G>T	TGCAGAATTTCTGTCCAAATCAACAGAAATGAAAAAGGGCAATTACCCAGAGCTAAAATCTGATTTTTT
39716373|F|0-48:C>T-48:C>T	TGCAGCTCTGCAAATTGGGATCGAAGGCGTCCAATGAATAGAACCATTCGGAGCCGCCATTGTTGGGCA
46757482|F|0-63:T>A-63:T>A	TGCAGTTATACTTTGGATTTTTGATACATTACTTCCAGCCATTTTTTTCAAATTCGTGGCTAGTCGAAA
39713842|F|0-52:A>C-52:A>C	TGCAGAAGCCTTTTGGACTTTGAGAGGTAGGCCTGCATCATCTCAATCTCAAAGTAACGAAGAGAATCA
39715103|F|0-65:T>G-65:T>G	TGCAGATTCCCTCTTTACGACGTCGATTTCGGCTCCGGCCGCCCGCTCTGGGTCACTTTTCCGGCTCTC
39716015|F|0-9:G>T-9:G>T	TGCAGCCGTGTGTTGACTAATCAGACATATCCCTCCTGTTTTCTCCACTTTTCCAGCATAGGGCCTCTC
39726095|F|0-24:T>A-24:T>A	TGCAGCATTTGATTCTGAAGCTTGTACGGTTTCAGAAACTTCGCCATCTGGATAACTAGTACGAGTTTG
39713771|F|0-56:T>C-56:T>C	TGCAGAAGAAGGAGAAAATGGCGGATTCTCGAGCAGCGAACAACGAAAAGGAGAGGTGGCGTTCGGTCA

<table>
<tr><td>39714637|F|0-47:T>G-47:T>G</td><td>TGCAGAGTAACAGGCTCGACTTAGCACTCGACTTTCTGGTTTTGATGTATGTGTGATTTGATTTCTAAT</td></tr>
<tr><td>39714607|F|0-28:G>A-28:G>A</td><td>TGCAGAGGGTGGCTAACTTCCTCACTTTGTCTAGTACTTGGCTGCAACCGAAGTTCAGAGGTGCGGCAC</td></tr>
<tr><td>39718540|F|0-50:G>T-50:G>T</td><td>TGCAGTTACAAGATGACGGGTGGTTGATGGGTGTTGAAGAAGAAGAAGTAGGAGCTAGGGAGAGAGAAA</td></tr>
<tr><td>39722938|F|0-51:G>A-51:G>A</td><td>TGCAGCTGAATTTGCCTTTTCTTTTGAATCCCATTACGCAGCTTTTGATGCGCCTGGTGGTTTTCTTTT</td></tr>
<tr><td>39717234|F|0-28:A>G-28:A>G</td><td>TGCAGGCTTTGCTACGAGACTGATGGGAATGAAGGTCCTTGTTCTTGACGAAGCTGATCATTTATTAGA</td></tr>
<tr><td>39718055|F|0-34:A>C-34:A>C</td><td>TGCAGTCATATATCAATTTTATGTTTGCACCTCCATATCATCATCTCATTGCTCCGAATACTAAACAGG</td></tr>
<tr><td>39717124|F|0-49:A>T-49:A>T</td><td>TGCAGGCCATTTATCTTTTCTCAGAGGTTCGGCCGAGCAAACTAAACAAATTCTTCTGTTTTTTCTTGG</td></tr>
<tr><td>39715338|F|0-45:A>G-45:A>G</td><td>TGCAGCAAGCCAGCAACTTGGGTCGTTCCGTTTCAATCGAGTGCAAAAGAAAGTGAAGGCGAAACAGAA</td></tr>
<tr><td>39728376|F|0-30:C>T-30:C>T</td><td>TGCAGTCAATCAAACTAAGTGGTAGATATTCGATCTACTACATCTGTTTTTTTTTTCCGCTCTATTTTT</td></tr>
<tr><td>39721732|F|0-11:C>T-11:C>T</td><td>TGCAGAGACGGCAGAAGAAGAAATTAGCACAATTCTTCCAGATCCTTTGTCCGTCATGGGAGAAGAAAG</td></tr>
<tr><td>39722956|F|0-59:A>T-59:A>T</td><td>TGCAGCTGCATGTACGGAATAACGGTTTATGTCCACGTCATCAATAAATAACCATAAGTATTTTTTTT</td></tr>
<tr><td>39717152|F|0-47:C>G-47:C>G</td><td>TGCAGGCCTTGAATCCGACGAGACGAAGCCATCTCGTTGTGCACATGCTTCATCTTGTCGAGCTTCTTG</td></tr>
<tr><td>39716207|F|0-8:T>C-8:T>C</td><td>TGCAGCGTTGCCGCCGTCGTCCAGTTCGTCGCAACCCGAGCGCTGCCGCCGCGTCTTTCTCTTTCTCCT</td></tr>
<tr><td>39718329|F|0-36:G>T-36:G>T</td><td>TGCAGTGCAAAGAGCATATATATCTAGTTGACAAGAGGGTAGTGCGTATCAATACCATTTGGAAACAAA</td></tr>
<tr><td>39727561|F|0-43:A>G-43:A>G</td><td>TGCAGCAGGCGTTGATTCTTGGAAACTTGGAAGAACCTGGAGTACTAAGGAATTATGATGGAGGGGAAC</td></tr>
<tr><td>46754652|F|0-51:C>T-51:C>T</td><td>TGCAGATCATTGTGAATGACTTGATTGGAGAGAACGAGGAGGTTTGAGGATCCATATAGGGACGTTTTA</td></tr>
<tr><td>39716398|F|0-5:T>C-5:T>C</td><td>TGCAGTTGAGATCCGGAATAAGGTCGAGGACAGAGGATCATGGTCATCAAGGGCGCTCTGGAAAGCGCG</td></tr>
<tr><td>39723920|F|0-13:G>C-13:G>C</td><td>TGCAGTCAGCACTGCATCTGCATAAACCATTGAAAAAAAACAACAATCAATGACATACACTTAGAATTC</td></tr>
<tr><td>39720238|F|0-12:G>A-12:G>A</td><td>TGCAGCTTGCTTGTAACTTGAAATTTTCAGTTGCTGAGATTTTGGCACAGTACTTTAC</td></tr>
<tr><td>39723011|F|0-48:T>C-48:T>C</td><td>TGCAGCTTCAATATTTCATTGGTTATTTTATTTGGGACAATCCCTTTTTGGTGTCCAATCAAATAACCA</td></tr>
<tr><td>39718141|F|0-30:G>A-30:G>A</td><td>TGCAGTCGTATGAGGCCAGTTTGATTGGAAGAGTTCAGAATGAGAAGATTGGAGTTCAAGCAAAAATAG</td></tr>
<tr><td>39717450|F|0-57:C>A-57:C>A</td><td>TGCAGGTACGACATATTCGACGTTCTTCACTTCCAAGTGTTTCATTAGACTGATGTTCCGTTGGTTAGT</td></tr>
<tr><td>39714744|F|0-27:A>T-27:A>T</td><td>TGCAGATAAGCTAAAGTTTTGATCTCTAGGATGGCTGTTCAAGTACGGTTCCTTTGAAATAGAAAATCG</td></tr>
<tr><td>39716263|F|0-30:A>G-30:A>G</td><td>TGCAGCTACTCGGTCCTTGGCTAGAGACAAAACTTCAATGCAATAATTTCTATGCTGAAGGGGTAACAT</td></tr>
<tr><td>39717758|F|0-11:T>C-11:T>C</td><td>TGCAGTAACTTTTGCCATGGATTTATGATTTCATTCTAATGTTGATAGGTAAGTATTCCAATAAGTCCA</td></tr>
<tr><td>39725057|F|0-5:G>T-5:G>T</td><td>TGCAGGAACGAGAAGTGCGAAATAGTTTCTCTTCGATGGCTTGTGTTAC</td></tr>
<tr><td>39726037|F|0-14:C>T-14:C>T</td><td>TGCAGCAGAGAGAACTGAATGAGGCATGTATTCAGCTGCGGACTGTAATTCTTATTGTATTTGTCATAT</td></tr>
<tr><td>39715605|F|0-41:C>G-41:C>G</td><td>TGCAGCAGGGCATTTTAGACTAAAATATAAAGCAGAGAATGCCATTGCTATTTGGCTAATTGCAACCGC</td></tr>
<tr><td>39718952|F|0-11:T>C-11:T>C</td><td>TGCAGTTTTCTTCTATCGATAGCATTGGAAATGAAAAAAAAAATTCTTGAATGACTTATAAAGTACACG</td></tr>
</table>

<table>
<tr><td>39715108|F|0-8:T>C-8:T>C</td><td>TGCAGATTTGTGAACTTAGGGTTCTATCTAAATGGTGAAGGGTTCCACTATGATTTAGTTGGAGCAATT</td></tr>
<tr><td>39727514|F|0-7:A>G-7:A>G</td><td>TGCAGCAACCACAAAAGGAGGAGTTGACCTTGAGGCACTTTTCTGATGATGTTGTCACAGGTCATCAAG</td></tr>
<tr><td>39718113|F|0-5:T>C-5:T>C</td><td>TGCAGTCCTCTCCTCTAGTTGAGAAATCTGAAAATGATAACCTCTGAGGCTCTAAATTCAAGTGCAATC</td></tr>
<tr><td>39715085|F|0-15:G>A-15:G>A</td><td>TGCAGATTATTTAGTGGCAGCATGTGGTGATGGCTTTATCTCAGTGCTGCCGGTGCTCGATACACCCCG</td></tr>
<tr><td>39715867|F|0-41:T>A-41:T>A</td><td>TGCAGCCAGAGTGAAATATAAACCATCCCACAGCATTCCATTACATGATCTAAAAAAGAAAACTACCCA</td></tr>
<tr><td>39720134|F|0-13:T>A-13:T>A</td><td>TGCAGCTAGGCTGTCAACTTTCAGTCCAGACGCTTTACTTGGGCAAGTTTACTTAC</td></tr>
<tr><td>39727445|F|0-52:A>T-52:A>T</td><td>TGCAGAGCCAATTGTTTGCTGTCTCAATAGCTTCTTCACTGGATATCTGCAACAATCACTGACACTAT</td></tr>
<tr><td>39725832|F|0-43:C>T-43:C>T</td><td>TGCAGATCAAAACGACGTAGTATTTCGTCTCCCCTAGTCCAAACTCTGCTGCTTCTCTGGGAATCACCT</td></tr>
<tr><td>39713339|F|0-50:C>T-50:C>T</td><td>TGCAGCTCGAGCAACATTCTTGAGAAATGACTTGGCGGCGTGGGCAGACCCATCGTGATTGATTGAAGA</td></tr>
<tr><td>39717437|F|0-54:T>C-54:T>C</td><td>TGCAGGTAATCACCATTTGAGAAGTGGGATTGGATAATCGAATTCCAAATAAATTCGTCCTAATTATGA</td></tr>
<tr><td>39715164|F|0-25:A>C-25:A>C</td><td>TGCAGATTTAGAATCGAACTTTGAGAAACCGTTGTTCGAAGAGTGTCCATAATAATTTCGATTATTGCA</td></tr>
<tr><td>39717989|F|0-28:T>C-28:T>C</td><td>TGCAGTATTTGATTCTCCTAGATATCACTGGAACTGTATCATTTCTAATTTCGTGCTGGATTGAGCATA</td></tr>
<tr><td>39714008|F|0-15:T>A-15:T>A</td><td>TGCAGAATCCTTGTGTTTGGGAATAAGTTGTGTAAAATCTGCACACAGTGTTTCTTCTTCTTCCTGCTT</td></tr>
<tr><td>39724780|F|0-7:A>G-7:A>G</td><td>TGCAGATAATTCTTGTTCTCCCATGAGAGGGTTTAGTTTTATCGTTAC</td></tr>
<tr><td>39717861|F|0-14:C>A-14:C>A</td><td>TGCAGTAGAAATTGCTTGGATGAACCACTACAATGTGCTTACGTTAGTAATGTCCTTATTTTTTATATT</td></tr>
<tr><td>39714733|F|0-25:T>G-25:T>G</td><td>TGCAGATAAATTTAGAAAACCACAATCAGAGACTTGCTTAGCCCAACTCACATTTCACTGTGAACTATC</td></tr>
<tr><td>39724227|F|0-26:T>C-26:T>C</td><td>TGCAGTTATCTGAATACTCAGGTTCGTTTCTCCCACTTCCTTCTCTATGCTATCTAAATTAC</td></tr>
<tr><td>39713556|F|0-34:C>T-34:C>T</td><td>TGCAGAAAGGATCCAAGGGCAACATAGACCATTGCAAATTCAAACCAATATTTTGTTTTTTTTAGACAA</td></tr>
<tr><td>39714921|F|0-51:A>G-51:A>G</td><td>TGCAGATCTCTCTCAAGCTCTAACCAAATGTTTTTCTCCCCCTCTCCCAAACTTGAGGTCAAAGATGA</td></tr>
<tr><td>39717900|F|0-29:C>T-29:C>T</td><td>TGCAGTAGCTCCAGGTTCTGCCAAGGCACCCAAAAAAAGATATTTGCACCAAGTAGCCAAATTTGTTAG</td></tr>
<tr><td>39716844|F|0-60:C>T-60:C>T</td><td>TGCAGGACATGTTTTTACCATCTCAGTGGCTCCTCCCTGTTTAGTAAGAGATACTTCAATCTGGGAGAA</td></tr>
<tr><td>39726792|F|0-15:A>C-15:A>C</td><td>TGCAGTTCCCATGGCATTTATAGCTCGAAAGGATCAACAGAATATTCAACAACTGTGCTTAC</td></tr>
<tr><td>39713355|F|0-14:A>G-14:A>G</td><td>TGCAGGGGTTCGTCACGGCTCATTGGATCTGGGTGTTTGGGGGTTGCGGATTTGTGGTGGTTGCATGGA</td></tr>
<tr><td>39717674|F|0-60:G>A-60:G>A</td><td>TGCAGGTTTGATATCTAAATTGAAAACGAAGAGGTTCATGATTCAATTCATCAAAAGAAAGCCTGGAAG</td></tr>
<tr><td>39725872|F|0-14:C>G-14:C>G</td><td>TGCAGATGAAAGAACAAACCAAAATTACTCTATCCATTTCCAGTATGTGAAACTAACTTGCATTGAATA</td></tr>
<tr><td>39713584|F|0-60:A>G-60:A>G</td><td>TGCAGAAATAATAGAAAAAGAAACGGAAGAAAAGAAAGAAGATTTTAGTAAAAACGATGAAGAATAAAT</td></tr>
<tr><td>39718790|F|0-32:C>A-32:C>A</td><td>TGCAGTTGGCTGCTAGATAGAGAAAGGGAAAGCAAAAAAGAAAAAGAAAAGAAAAAAAATTAC</td></tr>
<tr><td>39714062|F|0-54:T>G-54:T>G</td><td>TGCAGAATTACAAATTTCTAAAGCTTTACCTCGTTATAATGAGCAGAAATTTCCTCATGAAGAGATACA</td></tr>
<tr><td>39717222|F|0-23:T>C-23:T>C</td><td>TGCAGGCTTCTAGATATCTTTTGTTTTTCCCACAGACGATGCCAACTGTTATGCCAAAATTCGGACAG</td></tr>
</table>

<table>
<tr><td>39718470|F|0-25:C>T-25:C>T</td><td>TGCAGTGTAATATTATGGGCAAAAGCGGGTTGGACCGGTTCGATTTCTTTCACTCCTATGTAAATACCG</td></tr>
<tr><td>39717690|F|0-19:T>C-19:T>C</td><td>TGCAGGTTTTTCTTTCTTCTCCTCCAATAATGTGGTGTAATAGTGTTTTTGCTCTTGCCTTTAGCTTGT</td></tr>
<tr><td>39715687|F|0-58:T>A-58:T>A</td><td>TGCAGCATCACAGATAGAAGAAAATACAACAGGTAAACTGTTGATGAAATATAACAACTGCTTCTAGGA</td></tr>
<tr><td>39716124|F|0-47:G>A-47:G>A</td><td>TGCAGCGATCATCTAAACATGTCCATTCCAACCTCAATACTTTTTGTGCAGGCCACAACATGCAACTCG</td></tr>
<tr><td>39717525|F|0-9:T>G-9:T>G</td><td>TGCAGGTGATGGTCGATCAACAACACGATTTCACTGTGGGTATCGTTCAATCGGCTGGTGGCTGTTTCG</td></tr>
<tr><td>39718870|F|0-17:G>A-17:G>A</td><td>TGCAGTTTCCAGCCGCAGACCCTGCAACAAAATCCACCAGCTCAAACTGAGAAAGGGAAGAAGAGAAAG</td></tr>
<tr><td>39718703|F|0-51:A>G-51:A>G</td><td>TGCAGTTGAAAAGAGTTTGTTTTTGCTTGCTATCTTGTTTACTATATGCTTATTTTCTCATTACTCCTT</td></tr>
<tr><td>39729597|F|0-57:G>A-57:G>A</td><td>TGCAGCTGTTGAAAGGGCCCAACAAAAGGTCCCCATCGCAAGAACTTTAGGACGGTCGTAGTTAC</td></tr>
<tr><td>39723153|F|0-6:G>A-6:G>A</td><td>TGCAGGGATACTTCTCAATTCAAAGGAGAACCGAACTATTTTTGGTAAAGTATTTCATAAGTGAGGTAA</td></tr>
<tr><td>39713546|F|0-51:A>G-51:A>G</td><td>TGCAGAAAGCAATGAACTTGTTTGATCAATCTAGTACTGACACCATATGGTAGAGGATGGAGGAAAGAG</td></tr>
<tr><td>39725225|F|0-22:G>A-22:G>A</td><td>TGCAGTAACAGTTAGAGATGAAGCCTTCTTTAC</td></tr>
<tr><td>39723126|F|0-60:A>G-60:A>G</td><td>TGCAGGAAATGTAAATTGATTCAAGTTGTACAATATTATCTAAGAAAGGATGGGAGAACAAGAAGGTAG</td></tr>
<tr><td>39718332|F|0-34:G>A-34:G>A</td><td>TGCAGTGCACAAGTCGAATCAAAAGCTATATTTGGTATGGTTGGAAATCTTATTCAATATCCTACAACT</td></tr>
<tr><td>39713728|F|0-51:A>G-51:A>G</td><td>TGCAGAACTAGTCTGCAAGTTAGACTGGGAGGCAAAAACTATGGCTTTAGAATCTGAAGAATTGCTGAA</td></tr>
<tr><td>39713820|F|0-39:A>C-39:A>C</td><td>TGCAGAAGATGATAACTCAAAACCACGCAACTAAACAATAGAGGTAGACAAGATTGGAGAAAAATGAAC</td></tr>
<tr><td>39714537|F|0-6:G>A-6:G>A</td><td>TGCAGAGCTTCGTGATCGGTTTTCTGACATTCGCTTTGTTCGTTTGTCTACCATAAAGTAATTTCACTT</td></tr>
<tr><td>39720442|F|0-33:A>G-33:A>G</td><td>TGCAGGGCAAATGCATCACTGATGCAGCCTTTGATTCTCTTAC</td></tr>
<tr><td>39723235|F|0-10:G>C-10:G>C</td><td>TGCAGGATAAGTTGTTATCGATTGCCATCTCTTGCCCTACAACCTGCATATGCTCTCTCCAATTGATAA</td></tr>
<tr><td>39727162|F|0-15:C>T-15:C>T</td><td>TGCAGGGATTGATTACGAGTGCCCAGAAATTGTAAGAAGTTTTTTATTTTCTTAC</td></tr>
<tr><td>39717625|F|0-14:T>G-14:T>G</td><td>TGCAGGTTCTACCATTTGATTCCTGCTATCAAAAGAAAAATAAAATTGAGAAAGCTAGAAAATAACTCG</td></tr>
<tr><td>39715510|F|0-47:A>G-47:A>G</td><td>TGCAGCAGATAAGATCAGACTCCTATTTACTGAATGGGATAGGAAACATATTTGTCTATATAGACACAC</td></tr>
<tr><td>39721835|F|0-35:A>C-35:A>C</td><td>TGCAGAGGAAGGAATTGTGCAGAGGCCACATTCTTATCCACGTGTCGAGTAAGGAAATATTCCAGAATG</td></tr>
<tr><td>39713306|F|0-10:G>A-10:G>A</td><td>TGCAGTGAGGGCGTGAGTGAGGGGCGGAATTCAGAAGGGTGATCAAGGTGGGAGGCGGAGCGCCGCCGG</td></tr>
<tr><td>39713647|F|0-50:G>A-50:G>A</td><td>TGCAGAACAAAGCAAGAGAGGGAATGCCATAAATGATAATAGATATGAAGGAACAGTTTCATATGGCTA</td></tr>
<tr><td>39713709|F|0-29:C>G-29:C>G</td><td>TGCAGAACCGATGAGATAATATAGAGAGACAACATTGCATTGCATATGACAGAAACGCATCCAATAAAG</td></tr>
<tr><td>39713610|F|0-22:A>T-22:A>T</td><td>TGCAGAAATGAATTATTGGCGAAGGAGAAACTCAAATGCTAAATAAATATCTTTTTCTGTTTCTGTGGG</td></tr>
<tr><td>39717529|F|0-45:A>C-45:A>C</td><td>TGCAGGTGATCTTTTTATCTTACTTAGGGTGATCCTTATTCTTTTATATTCAAACTTTGTAAAATGTAC</td></tr>
<tr><td>39721587|F|0-36:G>A-36:G>A</td><td>TGCAGACATCAACTGCTCCATCTAAATTGCAGAGAAGCGTCAGAAAGGAAAATCCAGTTGATTTTGAGG</td></tr>
<tr><td>39716623|F|0-64:G>A-64:G>A</td><td>TGCAGCTTGGGTCAACCACTTCCTGAAAAGACAAGGAGAGTTTGAAGATGAAAAAGAGAAAGCCGCAGA</td></tr>
</table>

<table>
<tr><td>39713957|F|0-53:A>G-53:A>G</td><td>TGCAGAAGTTTGCTTGAGAAGCATTAGTATATAGCTCGAAACTTCGAAAGAAGAGTTCTCGAATCTCGA</td></tr>
<tr><td>39716747|F|0-20:G>A-20:G>A</td><td>TGCAGGAACAAAATGCGGCCGAATGAAGGACAAGGAAAATCTTTACAATTGCCCAAAATGGTCTAAATT</td></tr>
<tr><td>39718478|F|0-61:A>C-61:A>C</td><td>TGCAGTGTACATGCACCAAGTTATAACTAGAAGAAGGCAAGTAGAAACTCTCCAGAAAACCAAAAAAAG</td></tr>
<tr><td>39722720|F|0-24:A>C-24:A>C</td><td>TGCAGCCTCGACAAATCTTTTCTAAAGCTTCTGTTGCTTTTGGCTGACTTTATTTGCTTTTCTACCTTG</td></tr>
<tr><td>39724323|F|0-44:A>G-44:A>G</td><td>TGCAGTTGCAACACAAAAGAGGGAAAATCTCTGGATTCGATCCTAAGTGTTCACCCACCCATGCAAAAT</td></tr>
<tr><td>39721180|F|0-20:T>C-20:T>C</td><td>TGCAGAAACGCCTCCACCACTGTGTATCATATTACCTACAGGCTCGTCCAGCTACAAAAACGCCTCCAC</td></tr>
<tr><td>39722591|F|0-31:C>G-31:C>G</td><td>TGCAGCCAAGATTGACTCTTGAAGATGCCTTCGGACAAAAGGGGAAAAGCAATAAGTTTTCAAAAAAAA</td></tr>
<tr><td>39713720|F|0-28:C>T-28:C>T</td><td>TGCAGAACGCATGTGTTTCATATGTATCCAGAATTATTCTTGTTTCCCCTCAAACATCAACTTGGCTTG</td></tr>
<tr><td>46755830|F|0-39:G>A-39:G>A</td><td>TGCAGCTCCTTTACGAAATTTGTGCTGATTCAAATAAGTGAGTGCTATTATTTGAACTTTCCATGCACT</td></tr>
<tr><td>39722450|F|0-26:C>T-26:C>T</td><td>TGCAGCAGCTAGGTTTGAAGCTGCTCCTCTCTCTCATCATCAATTTGAGAAGTTCAATTCCATCTCTAA</td></tr>
<tr><td>39718315|F|0-48:T>A-48:T>A</td><td>TGCAGTGATGGATTCTAATAATATATAGCTTAGATGTAAAATGTAGAATAAAGCCAACATATGCAGCCA</td></tr>
<tr><td>39728759|F|0-38:A>G-38:A>G</td><td>TGCAGGGAAAAAAATAGAATGGGTCTACATCGATGCAGACTTGTGTCGACATAGATCCCAATTAC</td></tr>
<tr><td>39715010|F|0-25:A>G-25:A>G</td><td>TGCAGATGGAAGAAGAAAGAGAAGCAAGGCGTAAAGCGGAAGAGAGATCCATAGAGTTGCAAAAACAAA</td></tr>
<tr><td>39724964|F|0-8:T>C-8:T>C</td><td>TGCAGCCATTTTTTCCTCTCAAGACCTGAAAGTTGAAATTGGCATTTAC</td></tr>
<tr><td>39718124|F|0-54:A>G-54:A>G</td><td>TGCAGTCGAAGCTGCAAAATTGGGCGAAAAGGAAACCAAGGGAAGTTTCCTTCCAAATAATTTCTTTGC</td></tr>
<tr><td>39720546|F|0-25:A>C-25:A>C</td><td>TGCAGGTTTATTTCACTAATAAAAGAACAGTAAATTAC</td></tr>
<tr><td>39723847|F|0-63:T>G-63:T>G</td><td>TGCAGTATACGTATTTTTTGTTGTATGTTTATTGCAAACTTCTAAAAGACTACTCGTAGAAATAAAAG</td></tr>
<tr><td>39713424|F|0-12:A>C-12:A>C</td><td>TGCAGAAAAGAAAATAAAAAGGGGGACAATTGGCAAGAACCATACTTATGACAAGTAAGTAGTATAGAC</td></tr>
<tr><td>39723254|F|0-15:T>G-15:T>G</td><td>TGCAGGATGAAAAGGTACTTTCGGCACCAAATAGTTGTTTCTCCCCCTCTCTATGGGAAGAAAAATTTC</td></tr>
<tr><td>39714926|F|0-57:T>C-57:T>C</td><td>TGCAGATCTGTTGAATTCCCCAAATTCCGATATAAACCCATCAACGTACCATTTCGCTCCTATGATTTT</td></tr>
<tr><td>39728903|F|0-68:C>A-68:C>A</td><td>TGCAGCTGTCTATGAGGCAAAAGAGAGAATATTCAAATAGCATCTTCAATAAAAAAAAAAAAAATTAGAC</td></tr>
<tr><td>46756920|F|0-53:C>T-53:C>T</td><td>TGCAGTAAATAAACACCTTGGTGAAACAAATATGAATGCCAGGAGAAGTAGGTCGCGCACAATATTCAA</td></tr>
<tr><td>39713419|F|0-16:G>A-16:G>A</td><td>TGCAGAAAACCAAACTGCTAACCTTGCAATTGTCAATCTTCGTAGGAGGTAGGTTTCTTCTTGAAGTGT</td></tr>
<tr><td>39717127|F|0-63:G>T-63:G>T</td><td>TGCAGGCCCATTTATGTCAGCGCAGTCCATTTTGGGAGCTTCATCCTGTTTTCTATTGGGCTTGGGCCC</td></tr>
<tr><td>39716899|F|0-37:C>A-37:C>A</td><td>TGCAGGAGATTGGTGTATGCGATCAGTTTTCTATTTTCTTGTTTACCATCTGTCATTTTGTTACATTCA</td></tr>
<tr><td>39715727|F|0-65:G>A-65:G>A</td><td>TGCAGCATGAAGCAGGACCATCCTGATCCTGATGTGACGTTCAAACTTCAAAGTAAATATCAATGGCAG</td></tr>
<tr><td>39721576|F|0-30:T>C-30:T>C</td><td>TGCAGACACTTGATGGGAAGACTCCACAACTTGGAAGAAATCAAGAACGAAACCCAAGCCAAGATCCCT</td></tr>
<tr><td>39719685|F|0-29:A>C-29:A>C</td><td>TGCAGATACATAGGGAATCTCCCTACATAAGGGCAGTCAAAGTTTTTTTTAC</td></tr>
<tr><td>39716593|F|0-43:G>T-43:G>T</td><td>TGCAGCTTCTCTATAACAAGCACCAGAATCATAATTGAGAATTGCGCACATGATTCAACTAATAAGGGG</td></tr>
</table>

39717103|F|0-43:G>A-43:G>A	TGCAGGCATTGAAGTGACCAGTGTAGAATTTGAAGGAAATGGAGATATTGGAGATGAGGAAGAACAGTT
39714725|F|0-58:T>G-58:T>G	TGCAGATAAAAACGAGAACACAAAATATGGAAACATCAAGGCGCAAATCTACAATGCATGTTAC
39725844|F|0-8:C>T-8:C>T	TGCAGATCCACGGTCTAATATGAGTACATGTTCTACATTGGGCTCCGCAGGTCCACGGCCTTATATTAC
39713915|F|0-44:T>G-44:T>G	TGCAGAAGTAAATAGAGTTGGAGGAGATGAATTTTCTGGAGTTCTTATGAATTACTCATCTTTTTCAGT
39725134|F|0-27:A>T-27:A>T	TGCAGGGATGTTGACATCCTCGTTTTCAAATGCCTTCTTTCTAACAAGAAACTTAC
39715640|F|0-23:C>T-23:C>T	TGCAGCAGTTGGATAGCCCTTTTCGGCCCTCTTCTGCGTCGGTCAGAACTGCGGTTAGTTACTGTAAAT
39715060|F|0-65:G>A-65:G>A	TGCAGATGTTGGTCAAGTATGTAATTAGGATAGGTGAACTTTGTTTTGTGGTTTTTTATTAGGATGACC
39714715|F|0-55:C>G-55:C>G	TGCAGAGTTTCATTGGTTATTCCTTGTCCTAACAAAAAGAAGACATGCACCAACACTACATGCACCCCA
39714584|F|0-26:T>C-26:T>C	TGCAGAGGCTGAGATTTGCAAAGAGATTAGAATAGGAAGCAGAGATAATTCTATTATTCTAAGATAGTG
39721245|F|0-31:T>A-31:T>A	TGCAGAAATTCCATCACAAATCAATTGCTACTCAACAAGAATCGTTTTTTTTTTCATTTGGTTGGGTGG
39713341|F|0-43:A>G-43:A>G	TGCAGCTGACTCGACCCAACCCGAAAAATTTATACCTCTCACTAGATTAGTTGTTATAGTTTATTTTGC
39718156|F|0-52:C>A-52:C>A	TGCAGTCTATGCTGGAGCTAACTTCCGCAAGCCCGTTGAACCGTACTAGATCCTGCCTGGTGAATGGTG
39720196|F|0-16:G>T-16:G>T	TGCAGCTGCATTTTCTGGTGTTAC
39718039|F|0-12:G>A-12:G>A	TGCAGTCACTTCGCTGAATGCAACCCATGCTTGCCCTCGAAGCCTTGGTGTCTTCAAGGCAACAACATC
39713417|F|0-64:G>A-64:G>A	TGCAGAAAACACAACCATCTTGCAGACTCCTCTTTGGAGAATCATAAATTAGCTGCTCACTCTTGGATC
46764225|F|0-37:C>G-37:C>G	TGCAGCTTTTACCTGTGCCCTTCCAATTTCCAACATGCAATCTCATCTCACCTCTTATAGTCTAATTCT
39720235|F|0-32:T>C-32:T>C	TGCAGCTTGAATGACTAAACAGAACGCAATCATAAAAGGAGTTTCGATTAC
39716209|F|0-6:G>T-6:G>T	TGCAGCGTCGGCTTCAGTAAGATCGTCCGAACCAAATTACAGAATTATATTTGCAAAAGCAACTGAAAA
39722140|F|0-39:T>C-39:T>C	TGCAGATTAGCACAAACGTCATGTGAAAAGTTAGGCATATAGGGGAATACAAGACCTTGGCAAAGGAAA
39715653|F|0-19:C>G-19:C>G	TGCAGCATAACTTGAAAATCTGTTGTTCCAAAACATGACATAAGGAATTCCCTGCACAATGATGATCTG
39713701|F|0-26:A>G-26:A>G	TGCAGAACCAGCGTTTGAAACTTTCCAAAGGGTTAGTTTTCAATTTTTTTTAGTTTTGGTTTGATTTTT
39716772|F|0-60:G>C-60:G>C	TGCAGGAAGAACTCAAGCAATCCAAAGAAGTAACAGTGGAAATGAAAACATTACTTTTTGCATAGAAA
39713280|F|0-34:T>C-34:T>C	TGCAGCCATGCAGGTAGGCGTTTCTGTATTATTCTGAACTTTATTCTTTTTTACTTTTTTACTGTTGAT
39714173|F|0-8:T>A-8:T>A	TGCAGACATCTTGGTCCAAGTTCCGCCTATAAATTTGCTTGCCTTGTCTACCATAAACGAGCAGCCCCT
39715311|F|0-5:C>T-5:C>T	TGCAGCAACTGAGCCTTTGCATGTGGGAGGAACCATTTTTTGCCTTTGCTTGTATGCTGCGTTTTCTTC
39714875|F|0-57:G>A-57:G>A	TGCAGATCATATTTATGAAAAATATCTAATAGTTATTATTTGAAGTAGTTTGCTAAGGTAAGTACTTTT
39713757|F|0-16:A>G-16:A>G	TGCAGAAGAAAGTTTCAGGACTTAGATTCATGTACTCAGATTCAGACATTTAGCAAGGCCATGCACAAT
39724475|F|0-49:T>C-49:T>C	TGCAGTTTTGTTGACAAAGATATTTAGAGCAATACCTGCTAAACTTGACTGGTCCACCAATTTGATTTA
39728903|F|0-66:G>A-66:G>A	TGCAGCTGTCTATGAGGCAAAAGAGAGAATATTCAAATAGCATCTTCAATAAAAAAAAAAAAATTAGAC

39713580|F|0-7:A>G-7:A>G	TGCAGAAAGTTTGTTGTATATCAGTTATAGTACAGTATACTTGAATTGCATACCAGAAAATGAAAATGG
39728654|F|0-17:C>T-17:C>T	TGCAGAAGTTTGAAATTCGATTCTTAGCTCTTAC
39713718|F|0-27:T>A-27:T>A	TGCAGAACGATGGAACCTATGGAGGTTTTTTTGTCCAAAAGTAACCCGTTTATCTAACAACGGATTGAA
39717602|F|0-22:C>A-22:C>A	TGCAGGTTATGGCGGCGAGAATCTCCGCCGTGATTGTATTGGCATTGGTGAGTTTGGCCACCGTTGATG
39720117|F|0-25:C>G-25:C>G	TGCAGCGTGTCCATGTCGACCTGTCCAAGAGGACCTGGATTCAAATTCATATTTAC
39717633|F|0-25:C>T-25:C>T	TGCAGGTTCTGTATGAACTAATGCACGTTTTCTTTCATTGATTTAGTGAAATAAGTGAAAGACAACGTG
39715823|F|0-20:T>G-20:T>G	TGCAGCCAAGGCATATGGTATATATAAAAAAATGTGAGAGGAAGTACAATAAGATTTTTTACGATATTT
39716182|F|0-52:T>C-52:T>C	TGCAGCGGCTGCTGCCGTTTGACTACGATGCGGAGAGTGAGTACCTTTTGGGTGTTCGGTATAACGTGT
39718004|F|0-38:G>A-38:G>A	TGCAGTCAAATGCTCTACCACTGAGCTATGAACCCTTTGTTGTTTTGAAACAAAAAAAAAAATAATTTTT
39722371|F|0-60:C>A-60:C>A	TGCAGCACGTTGGTATCTAAGTGATGGAGATATCCCAATTCCCAGGTACAAGGGTCCACTCCCATTGAC
39718378|F|0-39:T>C-39:T>C	TGCAGTGCTTTTTTGCATCCTTCTCCATTTCAATCAATCTGGGTTTCAGAAAGTAGTCTTCTAACGAGT
39723476|F|0-41:G>T-41:G>T	TGCAGGGCTCTAAACTTTTCTGCATGTTTGTTCTTATTTGGGTTTTTTTTTCAGTAAAGGGGTCTCCGG
39714014|F|0-29:G>A-29:G>A	TGCAGAATCGTCCTCAGCTAGACAGACTAGAGACTAAAACAAGATTCTGCAATTTGTTTCACCCATAAA
39729189|F|0-14:G>T-14:G>T	TGCAGAAGTTCGAAGAACATACTATATGGAGTATTTGGTTTGAAAGAGACCAAAAAGAACTTTTACAGA
39714017|F|0-42:A>G-42:A>G	TGCAGAATCTCAATCAGTGTTCATCCTGATCCCATTACCACTACCAACAAGCTTCAACATTTCAATTCC
39714376|F|0-46:C>A-46:C>A	TGCAGAGAATGAAATGCTTTCCCTAAAACGAGCAGGACAACGAAGACTCTGATAGGAAAAACTAGAAGA
39714325|F|0-65:T>A-65:T>A	TGCAGAGAAAAGGAGAGAATTGAAATGGAAGTGGTATGTGTAGAAGAAGATCAAATTTCAAGTTTTGAA
39714380|F|0-36:C>A-36:C>A	TGCAGAGAATTCTTAGAAACTACAATGTGGGGTGTGCAGAAGGAGTTACCGGGGATGGTGGCTTTGCTT
39716187|F|0-5:C>T-5:C>T	TGCAGCGGGAGGTTTTTTTGAATTTTATTGGAGTTTTCGTGGATTTTATCGCGTTGGAACGCGAAGGTT
39716184|F|0-66:T>A-66:T>A	TGCAGCGGCTGGGGGAGAGCGATGATCCTGGAAATCTCCTCTGAATAGTGTCTGTTGTATTTATACTGT
39715101|F|0-27:T>C-27:T>C	TGCAGATTCCACAAGGATTGTTCTGCATTTTTAGATTGTTTTTCTGGCTCAGTTATAATTGTTTGAGGC
39717439|F|0-24:C>T-24:C>T	TGCAGGTAATCTGTTCGGCATCGACGGAGGCGATCGATACGAAGCAGAAGAGTGAAAGGAGAAGTTTCT
46754969|F|0-17:C>T-17:C>T	TGCAGCAAAAAGCAAAGCCCAATGGGGTCCGCCACTGCGAAGCTTTCTGGCAAATTCGCCCTTTGGCGT
39729406|F|0-53:C>T-53:C>T	TGCAGAAGAAAGCACCATTTCAATAACTCATAGCTAACCAGAAAAGCGAATCACCTGGCTTTTAC
39715526|F|0-6:A>T-6:A>T	TGCAGCAGCAAACTCAGCATCAGTCACAGCACAAGCAAGCAGAGAAAGGTAGTTTGTATTTGAAGCCTG
39715096|F|0-33:T>C-33:T>C	TGCAGATTCAGTGCAAGGAGATCAACTCATTTATGCGGCTGCCTATTTCAACATCTTGACCACAAGCAT
39721738|F|0-35:T>A-35:T>A	TGCAGAGAGAAACAAGAAGAAGAAGAAGAAGATGGGTTTTGGTTGGAGGTTGCAGAGAGAAAAAGA
39718585|F|0-58:C>G-58:C>G	TGCAGTTATCCTGTCTATGTTCTTCTCCGATGGACTGTTGAGATTCTGAGTGCCTGGTCTATGGAACTG
39719363|F|0-18:G>A-18:G>A	TGCAGAACCAACCACCACGTCATTAC

39722868|F|0-20:C>A-20:C>A	TGCAGCTAGTATAGGACATACTTTTCTGTTCACTGTCTCCTCTCTCACCAATATTATTCAGTTTCATCA
39714320|F|0-21:A>G-21:A>G	TGCAGAGAAAAAAAATATATCAATACCAACAGGGAACAATATGATAAACCAAATGTGTGGTATGTACTT
39726970|F|0-30:G>A-30:G>A	TGCAGAATGCAGATGTTATATGGGAAAGTCGTGAAGATCCAATATGGATTAC
39716213|F|0-60:C>A-60:C>A	TGCAGCGTGGGATTTGAACAGCTCTTTTCTTGTCTAAACTTAGAATTCAGGCTGATTTATCGTTGAATT
39724447|F|0-56:T>C-56:T>C	TGCAGTTTGTCATCGGTGCTAACCTTCAAAGGGTAAGAGACCAAATACCAACTTGATTTTCCTGCTTAT
39714484|F|0-31:T>A-31:T>A	TGCAGAGCAACTTCTAAAACCAAAACTGAACTCTGAAATATATATTTGTTCTGATGAGTAGAGAAAGAG
39714603|F|0-9:G>A-9:G>A	TGCAGAGGGGTCTGGAATTTTCTCCACCAAATCAACATTTGAAAGCTTTGCCAAGCAAAGGCCAAAAAT
39714931|F|0-62:T>C-62:T>C	TGCAGATCTTATCTTGGTAAGTAGTTGAGGACTTCTTTCTCTGTTTTTCTTTATGCTCATTTTTCCTTT
39719921|F|0-5:C>G-5:C>G	TGCAGCACCTGAGGACTAACAACCTTGCGCTTAC
39713557|F|0-20:C>T-20:C>T	TGCAGAAAGGCACAAATTGACAAGTGTGTATTTCTTATGCATGAATGCATCAACCCACAAATGAATTGA
39726320|F|0-14:T>C-14:T>C	TGCAGCTTACTTATTTCACATGTTGAGTTCCTCGTTTCTAGATTGAAAATCACGTAAATAACTTGATGC
39718841|F|0-8:A>G-8:A>G	TGCAGTTTAGGCAATAATTTTATAAATCCATGTCCAAGTATATTTTTTATTATGCACATAAAATAAATA
39713543|F|0-27:T>C-27:T>C	TGCAGAAAGATGTGGCCGCCATGGCTGTGAGTCTCAGGCAGAGCGTGTGAACTGTGAAGAATGGTGGTA
39716186|F|0-65:T>G-65:T>G	TGCAGCGGGACTTACAATTCACGAAATAGTCCCTTTTGTGCCCATTGCCCAATCTTGATGATGATTGAT
39721758|F|0-36:T>A-36:T>A	TGCAGAGATATGAGAAGCTTTTCTAGAGTTGTTTCTTCTAAAACATCATTCTTCATCTCAAAATTCGTC
39713510|F|0-45:C>G-45:C>G	TGCAGAAACGACCTGGAGCGGATACGCATCATCATTCATTTGGCTCTCTAGCATACCATTTGCAGCATT
39716553|F|0-33:C>A-33:C>A	TGCAGCTTCATATTATTTCTGTTCCTAATAATACATGTGATATTTTAGTAGGATGGTCTGTTGTTGTCT
39716475|F|0-36:A>G-36:A>G	TGCAGCTGGGCTTTCGGCTCTCGAATTGCTGAAAACATCTTCTGTTGATAGTGAGTATTGAGATGAATT
39720257|F|0-21:C>A-21:C>A	TGCAGCTTTTGTTCATAAAAGCAAGTAGGAATCTCTTGTTAGATGGAAAATAGAATCACCCAGATCGGA
39720363|F|0-53:C>T-53:C>T	TGCAGGATGTAATGCTCTCTCAACTTCTAGCATAGCAACTTGGTTATGACTTACCTTAC
39721893|F|0-29:T>A-29:T>A	TGCAGAGTAAGTATGCTAAGTCGGGCAGCTAGGTTTTCTTCTGAAACTCATATGAGACATAAGATAAGA
39723165|F|0-41:A>T-41:A>T	TGCAGGAATGTAATTGAACACCCTGATGAAATGAAATACAGAAAGCTTCGCAAGGCACGACTATGAACT
39724770|F|0-49:G>A-49:G>A	TGCAGAGTGCTTGAAAACCCAACAGTTTTGGGGTATTTTCATGCCAATAGCTTAC
39718397|F|0-44:C>T-44:C>T	TGCAGTGGAGGCCTAACATTATCCCTCTGGACACCTTGTCCCTACGATAAAATTTTGGAAATTGGTGTT
39713576|F|0-6:A>G-6:A>G	TGCAGAAAGTTGAGAAGGCGCGCCATTGATGATAGGGAAGAAGACGCTAGCGAGCGCGCGAGAGTTCAG
39717922|F|0-6:A>G-6:A>G	TGCAGTATAAAGCAATGGATCTCAGGGAAAGTCCAAATGGACAACCAAGGTGTAAAAAAGACACAGAAG
39716474|F|0-66:T>G-66:T>G	TGCAGCTGGGCTGGGCTCATCAAAATTGTCCGACTAATGGAACAATGAGAAAAGGCCTGTCAACAGTAG
39726072|F|0-57:G>C-57:G>C	TGCAGCATAACCAAATATGATGTTTAGAGCCCATATGCACCATGTCGGATCGAATGAGTTACACAACCA
39716567|F|0-46:A>G-46:A>G	TGCAGCTTCCGATCGCAATGAAGGGTAAAGGGAAAGAAAAATCAATAAATAAACTATAAAATACAAGCC

39721072|F|0-63:A>G-63:A>G	TGCAGGTACCTCTGCGACAGTTACTAAGGAATAGAATTCCTCCTTGGCATCTTCCTCGTCGTTACGCTT
39716042|F|0-53:T>C-53:T>C	TGCAGCCTCCCTCCTAGCGGTTTTGCACTTATTTTCTACTCTCTTGTTACGGGTATTCTCCCAATATCC
39725133|F|0-6:G>A-6:G>A	TGCAGGGATGGTTTCGAAGGGTGGTTGGATTTAC
39714056|F|0-19:T>C-19:T>C	TGCAGAATGTGTCTACATTTGGAAGGGCAGAGGTAAAAATGTAACTTCTAGAAGTTATTTATTGTAGTT
39718251|F|0-36:G>C-36:G>C	TGCAGTGAAGCGGCGGCGAGCTTAGAAGAAGGGGAAGGGGTAGAATGGGAATATGGGAATTGGAGAGGG
39713856|F|0-31:A>G-31:A>G	TGCAGAAGCTTCTCGGTTCATTCTATGCTTGAGTATTTAGTGTTGCAAAGAGATCTTATCCCTGATCCA
39725085|F|0-38:T>G-38:T>G	TGCAGGATCAAAGCAAAATTCATCTCATGTGAAACACTTTTTTACTATGTAATTAC
39715396|F|0-23:C>T-23:C>T	TGCAGCAATTGGATTGCACATAACAACAACTTCAATGAGCAATACTTCAATAAATCATTTGTTGTTGAC
39716091|F|0-60:C>T-60:C>T	TGCAGCCTTGTCGAGATCGCTCGTCTTTCACGAGAGCTCTTGAAGGCGTCCGATCGTCATCGTCGTCGC
39717243|F|0-43:C>A-43:C>A	TGCAGGGAAACAACCTTCTAAAGGGAGAAAAAAATCAGTAAACCAAAAAAGAAAGGCCAAAACAGCAAC
39726639|F|0-51:A>C-51:A>C	TGCAGTCAAATGCTCTACCACTGAGCTAAGGACCCATTGTTGAAAGTAAATATCGCCTTTACCTTATAA
39713590|F|0-65:C>T-65:C>T	TGCAGAAATATTGAGAATAGAATTACTAAAAGTGAGGTATCTATATTTGAATGTAAGATTTCACCCTTC
39718191|F|0-46:A>C-46:A>C	TGCAGTCTGCTGATGGCTCCGAAGAACCCAAGACCAAGAAATCTTCAAGCAATGCTAAATCTGCCCCTT
39722402|F|0-9:C>T-9:C>T	TGCAGCAGACCGAACCGGACCCGAAAAGAAGAAGAAAAAAAAAACTGCTGCTGCTGGAATTTAC
39722931|F|0-59:G>C-59:G>C	TGCAGCTCTTTTTCTACTGAAGAAATTCTAGTCATTAGTTGATCTAGTGCCTCCTCAGCGACAGAAGGG
39720335|F|0-5:G>A-5:G>A	TGCAGGAGTTACCGTCGTCGTTAC
39715838|F|0-17:C>T-17:C>T	TGCAGCCACAGAATCTTCTTTTCTCCTTTTGCAATGGGGAAAAGACTGCCTAAAGCCAAGATAATGATC
39714485|F|0-68:G>C-68:G>C	TGCAGAGCAAGAACAGCACGTGATGTGCGAAACGTCGAAACCTTCTTCTTTGCAGAATCAGAATGAGAG
39714664|F|0-56:A>T-56:A>T	TGCAGAGTCCCAATAATTATCATTTTAGCTTGTCTCTATGAAGTGGATATATATATATTTTAC
39725299|F|0-43:C>T-43:C>T	TGCAGTGACCACCATACAAGTTCACATTGCGAACACGTTGAAACCTTTTAC
39714261|F|0-33:G>A-33:G>A	TGCAGACTAACCAACATTGCACCAAATGAGCTAGCACCCAAAACTTGTTTACTTGTACTGATGTAGTTC
39717792|F|0-56:A>T-56:A>T	TGCAGTAATGAATCTATAGTTGTACTGTAATAATGAATCTGAGATTATTACTCTCAATGCTGGAAGGCT
46760763|F|0-28:G>A-28:G>A	TGCAGAGAATCCCATTCCATTCTCCACAGAATTTAC
39717756|F|0-44:A>G-44:A>G	TGCAGTAACTGTCCTTAGCATTTGTAAGTTCACCAGCAAGTTTGACATTTTCATAAGAAAGTTTGGTCA
46760720|F|0-12:A>G-12:A>G	TGCAGAAGATAAAATATCCTCCCAAATTTGAGAGAGGTTAC
39715929|F|0-45:C>A-45:C>A	TGCAGCCCAGAACCAAAAGCAGAATGGAGGCAGTAACTGGAACTACGATGGCTACAATTGTCACCGTCG
39718862|F|0-30:C>G-30:C>G	TGCAGTTTCAGGCCCACAAGCCCAACCCAGCCTTTCATGTGGAAGTTCTTCGTTCCATTCTCTCCCTGT
39722135|F|0-23:G>A-23:G>A	TGCAGATGTTTCACTGAAGAAAAGTGATAGTCTAGTTATTCGCTCTAAGAGTTTGAAGTGATACTTAGT
39722480|F|0-32:A>T-32:A>T	TGCAGCAGTGAAATTAGGCATGGAACAGTGTGATGCGGTGACAGCAACAGAAACTTTGCGCACCAATGT

<table>
<tr><td>39721527|F|0-25:C>T-25:C>T</td><td>TGCAGAATTGGGGCGGTGCTGTACACGAAAAAAAATCTTCTACCCCAAGAAGGTAAGGTTTGGAAAGAA</td></tr>
<tr><td>39714022|F|0-29:A>G-29:A>G</td><td>TGCAGAATCTGTTAGTAATGAGTTGAAGGAAAACCAGAATAAGGGGGCATTGGTTCTTGGGATTTTACT</td></tr>
<tr><td>39725785|F|0-17:A>T-17:A>T</td><td>TGCAGAGGTAATGATACAAATGCAATTCAAAAGCTACTGAAATTGGAACACTCGTCTACATTCAAAGTA</td></tr>
<tr><td>39718066|F|0-52:T>A-52:T>A</td><td>TGCAGTCATGTGTGACCAGCCTTTGAATTGATTCCCACAATTTATTATTATTTTTTCTTTTTCTTTTTC</td></tr>
<tr><td>39727057|F|0-16:A>G-16:A>G</td><td>TGCAGCAAAGGCCGGCAAAACATCTCCTTCATGGCGCGACGGTGCAGGCGTTGATTTAC</td></tr>
<tr><td>39722976|F|0-39:G>A-39:G>A</td><td>TGCAGCTGCTTCTGCACAAAGCGGAGTGGTAAAAACCTGGGAAGATTTTGAGTGGACCACCTTGAGATT</td></tr>
<tr><td>39725525|F|0-30:A>G-30:A>G</td><td>TGCAGAAACTTTTGCTCGAGTCGAATCTATAGTTATAGCGATGTGGCGAAATCTCTGTGGGAACTGAAG</td></tr>
<tr><td>39715699|F|0-46:G>T-46:G>T</td><td>TGCAGCATCATTAGTGTGTCGTTCACGATCAGATCGGCACGACAGTGATAATTTTTCTTTGGTTTTCGA</td></tr>
<tr><td>39723415|F|0-6:T>C-6:T>C</td><td>TGCAGGTTTGGAGGATGAGGGAGTGGAATTTTTGAAGAAGTGTGAGGACAAATTGGGAATTACTCGTCG</td></tr>
<tr><td>39723790|F|0-66:A>G-66:A>G</td><td>TGCAGTACGACAGTTTTATATGATGCATAGCCAGCAAAAGCAGAGGCTTTATGTAGCATGTTTCTCAGA</td></tr>
<tr><td>39723805|F|0-38:G>A-38:G>A</td><td>TGCAGTAGAACCGGTTTTCTGTATATATAAATCTTCTCGCAAGGTTTCTCTGCCTTCTGCCGCAGCCTC</td></tr>
<tr><td>39722117|F|0-32:C>G-32:C>G</td><td>TGCAGATGGTTTCCAAGAAGGGAGGATATAACCAGCAGCAATGACAGAAAAGACAGAATAGTTAGATTA</td></tr>
<tr><td>39718904|F|0-47:G>A-47:G>A</td><td>TGCAGTTTGATGATTGGCAGATTCATCGCTGGACAGAAAGCAAATTTGGTAAAAACAAACAACGGACTT</td></tr>
<tr><td>39724309|F|0-15:A>T-15:A>T</td><td>TGCAGTTGACTGCAAAAAACTAATCTCCTTCAGCCTTTTCCCCTTTTTCCTGTCAGATTATAGATGAAC</td></tr>
<tr><td>39724184|F|0-38:A>G-38:A>G</td><td>TGCAGTGTTTATGCATTTTTAGTTGATGTTACTTTACCATGGTTGTACATTGCGTGGTTGTACATTGCG</td></tr>
<tr><td>39724011|F|0-25:G>T-25:G>T</td><td>TGCAGTCTGTCAATATTAGACAAAAGCAATTTACATGAGCAAGCATGTCTACAATAGTGATGTACTAAG</td></tr>
<tr><td>39720136|F|0-5:T>C-5:T>C</td><td>TGCAGTTAGTGGACTTGTTGTTCAATCTCTCTCTCTCTCGTTTAC</td></tr>
<tr><td>39718494|F|0-27:G>C-27:G>C</td><td>TGCAGTGTCTTGACGAACAAATACGAGGCACCATAACATCTCCGTATCTCGGCTTCATGACAAAATACT</td></tr>
<tr><td>39723103|F|0-35:A>C-35:A>C</td><td>TGCAGGAAAAAGTGACATCTCTAGTTCTCCTATCCACGACTTTGTTTGATAAGCATTACATCAGTGTTC</td></tr>
<tr><td>39717358|F|0-31:A>C-31:A>C</td><td>TGCAGGGGTATTGTTTTGTTTAGAGTTGATTATGATATTTTGTCTTTGGATGGAAAAGAGTCGTTGCAG</td></tr>
<tr><td>39725263|F|0-35:G>A-35:G>A</td><td>TGCAGTCAAATGCTCTACCACTGAGCTATGGACCCGTTTTTTGCTTATTAC</td></tr>
<tr><td>39725362|F|0-12:G>A-12:G>A</td><td>TGCAGTTGAGGGGCATGTTTTAGAATGTAATTAC</td></tr>
<tr><td>39728493|F|0-23:A>G-23:A>G</td><td>TGCAGTGAATTGTGAGCGAAATGAAAATGAAAAAAATATGTGCAACCAAGTTTTAC</td></tr>
<tr><td>39724795|F|0-21:C>A-21:C>A</td><td>TGCAGATATATCACAATTTTCCAATGAAACTTAC</td></tr>
<tr><td>39722078|F|0-29:A>G-29:A>G</td><td>TGCAGATGCAGAGGTGAAGGACTGAAATCACATTCCCTCATTTCAAGCACGATGCATTTCTCCCAGTTG</td></tr>
<tr><td>39726147|F|0-67:G>A-67:G>A</td><td>TGCAGCCGCCACCGGAGCTCCGATCCCGGAGACCAGCTGCCACTAAGCCTGAGCCACAACCAAGGCAGA</td></tr>
<tr><td>39720535|F|0-29:C>G-29:C>G</td><td>TGCAGGTTCTTTAGAGCCACAGATTTGAACTGCTCTACCCTTTTAC</td></tr>
<tr><td>39714455|F|0-43:T>C-43:T>C</td><td>TGCAGAGATCTATAGTTGTATTGTGTAATTTGATCTTTGTTTGTAGAAGATAAAGGAAAGACTATGTAG</td></tr>
<tr><td>39725621|F|0-62:A>T-62:A>T</td><td>TGCAGAATCCAAGAAAACTGCCCAACACATGTAACCCTAACCCACACTAAACTCTAAACCTAAAATATT</td></tr>
</table>

39725968|F|0-7:G>A-7:G>A	TGCAGCAGATAGAGACTCTCTCATTCAAAAGACCAACGACCTCGCCACAACATATATATTTTAGGATAT
39716430|F|0-65:T>A-65:T>A	TGCAGCTGCCAGCAATGCAAAGTAGAGATTGTTTATAAGAAAGTGATGAAGAAAAAGGAAAAAAATCAA
39716793|F|0-8:G>A-8:G>A	TGCAGGAAGTTACATGGAAGGAGGGGTCGGTGGATTCATTTACCCTAGTCGACAAACAAACAAAAATGA
39722892|F|0-53:C>T-53:C>T	TGCAGCTCAGTGTCGCCGCCGTCGTCGCCGCTTCTTTCCGATTCAGTGCCGCGCTCGTCTTTCTTCCGT
46755297|F|0-33:C>T-33:C>T	TGCAGCATAATGATTGATGTTAGTGATATTATGCGAACATCATAAACACTCAAGAAAGGAAAAAAATCAT
39718604|F|0-24:C>A-24:C>A	TGCAGTTCAACTAACAAACTTGACCGCTCCAGCAATACTTGACATAGAAGTGGTGAAACAAGAAATCAC
39717180|F|0-18:C>T-18:C>T	TGCAGGCTAATCCGAAATCTGTTGCTACTACTCAAACGTAGGTCAATATTGGGAATGTAATCTTGCAGG
46754977|F|0-25:T>C-25:T>C	TGCAGCAAAATCGATTGAAGATGCTTCATCGTCAATTCATCATCTTTATCCAATCCAAACTTCAAAATT
39715035|F|0-35:T>G-35:T>G	TGCAGATGGTTGAGAATCTCATGGAATTTTTGAGATAGAGAATTTTTGGGGAAACCCAAATGAGAAAAA
39718567|F|0-50:A>G-50:A>G	TGCAGTTAGCTCGAGTAAGTAATCTTCCTTAGTTACCACCTTCACTGGCGATGCTTCTTCTTCTTTGTT
39714051|F|0-49:C>G-49:C>G	TGCAGAATGTAGGAACGATATAAGAGATCACTCTAAGGTTGCTATCACACAGAATCGAATTCGGTGCGT
39716652|F|0-56:A>T-56:A>T	TGCAGCTTTCACTTTCAGATCTCATAATATTATATGATCCCTTGCCGCCCTTGTTCATGTTCATGTTCA
39716839|F|0-25:C>T-25:C>T	TGCAGGACACTTTCCTGAATCATTACGTATATCTATAAACTGATGCTGAATCATTATGTATATCTATAA
39721300|F|0-48:G>T-48:G>T	TGCAGAACTAGCTCAGGAAAGTTTATAAAAGTAATCAAAAGAGATACAGAATGAATTTTGAAAGTCGAG
39724229|F|0-37:A>C-37:A>C	TGCAGTTATGTCGCGGAGAGGGGTTCTTGGATACCAAATAGGCAATACACTATTCCTCCCCCGGGATCT
39726690|F|0-67:G>T-67:G>T	TGCAGTCTGTAGTATTATTACACTTCTTCTACTATAGAATGGCCAGACTTGGAGTTCTCCCCCTTGTGG
39717162|F|0-67:C>T-67:C>T	TGCAGGCGCCGTCGGCATCGGTGGATGACTTCATTGAAACGCAAATATTTCAACACGCCCTGGGCTGCC
39716353|F|0-22:A>G-22:A>G	TGCAGCTCGCGCCAAAACGTGCACCTCAGAGTTTTGCGAGCGAGGGCAGTAGGAGAAAGAGATTTCGTC
39725807|F|0-15:C>T-15:C>T	TGCAGATAACATTACCAACTTCGCATGGAGATTTTTGAAACAGAAATTGAGCAAAACATTTAGATTCGT
39724401|F|0-42:C>T-42:C>T	TGCAGTTTCAAATCAACTTGAACTCACTTGAACACGTTACAACAAGCTTTGAGTCATTTTGGGTGAGTT
39716166|F|0-26:C>G-26:C>G	TGCAGCGGCAGAGGGTGGATTTATTCCTACAAAGTTGGTTGTTTCCGATGTTCTTCCACAAATTACGGA
39718500|F|0-38:C>T-38:C>T	TGCAGTGTGGGTAAAGGTGATATTCTTCTACTTATATTCGTACTACTATGTGAGTTCATGGTGCTTGAT
39726944|F|0-40:T>A-40:T>A	TGCAGAAATGAAATTGATTACTCTTACGTCGTTTATTAGTTATGTTAC
39715599|F|0-17:C>G-17:C>G	TGCAGCAGGATCTGCCGCTAGTCCTAAGAACACTTCCTTAGCTGGAATCACTTGCCAAAAATTCCATGC
39716405|F|0-8:A>G-8:A>G	TGCAGCTGATCCGAGCTCTTTTACTAGATTTTGGTACACCCATGAATAGAAGTCAATTTGTTGGTTCAT
39717483|F|0-19:A>G-19:A>G	TGCAGGTATTGTATATGCCACCAACAGAGACGGCTGGATCAAAACACTGCATCCCAACGGATCTTTGGA
39727837|F|0-23:T>C-23:T>C	TGCAGTGAACTTGTATCTCAGGCTGTACGAGCATTGTGCAACATCAAATCTCTCCTTTTCTTCTTATCG
39718086|F|0-52:G>A-52:G>A	TGCAGTCCATATCACCTCTCTGTTAGTGATTTCACAAAATTTGGACGGCAAGGACCAAAATGCTCCTTA
39716335|F|0-11:G>A-11:G>A	TGCAGCTCCCGGTTGCCAATATCCCGACGCCACACTCGGCCCTGTCATTGATGCAGGTTTGTAAGACGA

<table>
<tr><td>39718758|F|0-13:C>T-13:C>T</td><td>TGCAGTTGCTAGTCTGTAACTCATAATTTGTTGGGTCCCAAGAATTGAAAAGTAAAATTTATATTCGTT</td></tr>
<tr><td>39717085|F|0-11:G>T-11:G>T</td><td>TGCAGGCAGTAGGGCAGTGGGTGGATGGCTATTTACAGTAACGGTTTAGCAATTTACAAAGGATCAAAA</td></tr>
<tr><td>39723209|F|0-29:T>A-29:T>A</td><td>TGCAGGAGGAGAAAATCTGTGTGGCTAGTTGATAAATTCTTTCGCTTCGCTTCCAATTGGTTTATCTCA</td></tr>
<tr><td>39724387|F|0-34:A>T-34:A>T</td><td>TGCAGTTGTTTGGTTCCTCTCAAGATGCATTTCTACTTGATTTATATAATATAATGTCAAGATAAGTGC</td></tr>
<tr><td>39724084|F|0-38:G>A-38:G>A</td><td>TGCAGTGCCCCAGAGAAAGCCACATTGCAGCCCATAAGGGAGATCTGTTTTCCATGATCTAAAAGTCTG</td></tr>
<tr><td>39716286|F|0-36:C>T-36:C>T</td><td>TGCAGCTATCGCGCTCTAAATCTTTGTGCTCTTTGTCCGTGTGTATCTTACATACATTTCTCAATAAAG</td></tr>
<tr><td>39713290|F|0-60:A>T-60:A>T</td><td>TGCAGGTCATGAAGCCTTCTACCAGTGTTGAACAAGGTAATAATTTATCCTTATTTATTTATTTATTTG</td></tr>
<tr><td>39721797|F|0-41:T>C-41:T>C</td><td>TGCAGAGCCAGTTTCGAAGATGAAATGCGCAAATTGATCGCTGCCGCCGCGGCGGAGGAGGTGGTCGCC</td></tr>
<tr><td>39727435|F|0-41:G>C-41:G>C</td><td>TGCAGAGAGTAATTCCCTTTTTCACCATTTTTTTGAATTCTGATTGAATCAATCTCATTTCTCAACATT</td></tr>
<tr><td>39716482|F|0-14:A>G-14:A>G</td><td>TGCAGCTGGTGGACAGTGGTGAAAGGGAGATCGTCAAGAGAAGGAGAGGGAGGTAGTGGTTGCTACTTG</td></tr>
<tr><td>39729252|F|0-42:C>T-42:C>T</td><td>TGCAGATCTTGTTTTCAGTAGATTTGAAGGCTGACTCATTTTCGTGGATTGTTAGTAACTATTAC</td></tr>
<tr><td>39727041|F|0-26:T>C-26:T>C</td><td>TGCAGATTCAAAATTTTCATATTTCCTACGATTTTTAC</td></tr>
<tr><td>39721992|F|0-15:C>T-15:C>T</td><td>TGCAGATCAAGAGGTCACCGGTTCGAACCCGGTTGGGCCCTCATTTTTATTTTTATTTTTATTTCATTT</td></tr>
<tr><td>39719748|F|0-29:G>C-29:G>C</td><td>TGCAGATGAAGGCTTCTTCTCTTTTTCATGTTAC</td></tr>
<tr><td>39722168|F|0-39:C>T-39:C>T</td><td>TGCAGATTCTCTTTCGAAAATGAATGAGAGAAACTTTGGCTACTATTCTCTTTTTTTCAGAGTCTTCAA</td></tr>
<tr><td>39728065|F|0-29:A>T-29:A>T</td><td>TGCAGGGGTTGCTATTGGCTCTTGTTTCAAACCGTTTTCACATTAC</td></tr>
<tr><td>39715269|F|0-8:A>G-8:A>G</td><td>TGCAGCAAATGATGGGCAATGGGGCCAACAGCAAGAGCATTGCTCTTATGGTGGCGGCCAAATTTGGTG</td></tr>
<tr><td>39717925|F|0-16:T>C-16:T>C</td><td>TGCAGTATACGAAAAATAGAGCACAAGCAGAGGAGCTGAGGAAGAAGCAAGGCCTGCTTGGCTGCTTGG</td></tr>
<tr><td>39723600|F|0-47:G>A-47:G>A</td><td>TGCAGGTCGTCTTCTTTTGATTCTTTTTGTAATACTGTACAAAATACGACCTCTTGGTGGATTTCTTCA</td></tr>
<tr><td>39724955|F|0-16:A>G-16:A>G</td><td>TGCAGCCATATCTATGATTGATCATGTTGTGTTAGAGGAAAACAATATTAC</td></tr>
<tr><td>39726729|F|0-14:C>T-14:C>T</td><td>TGCAGTGCATGTTTCTATTCCTAGATCCTAAATGCCGATCTAATGTTTTTCTCGATGTTTTCTTTGGTT</td></tr>
<tr><td>39726472|F|0-42:T>G-42:T>G</td><td>TGCAGGGGATGACACTTTTGACATTGTCAGCTTCAGTTCCTGTGGTTGGTGGAAATGCCATCCAGTTTT</td></tr>
<tr><td>39715997|F|0-41:C>A-41:C>A</td><td>TGCAGCCGCTATGTTCTTACTTCTTTGGTTGTTATGTCTGGCCCGTCTAAGTTTTGGCTGATTATGCTC</td></tr>
<tr><td>39722249|F|0-64:A>G-64:A>G</td><td>TGCAGCAAATAACACATACCAAAATTATTATCAACAGCGATGTTCTATTCATTTTGAAAAACAAAAATA</td></tr>
<tr><td>39729464|F|0-46:A>G-46:A>G</td><td>TGCAGGTTTAGGTTCTCAAAGGACCTTCAAAGTTTCAATATTGTCTATTAGATTCATGAACTTTACAGA</td></tr>
<tr><td>39714030|F|0-30:C>T-30:C>T</td><td>TGCAGAATGAATGGTCAAAGTACAGAATCTCGAAAAGCTTTGAACTCCGAGGGGGACCCCACGAATCCG</td></tr>
<tr><td>39716069|F|0-50:A>G-50:A>G</td><td>TGCAGCCTTACTTTCTTGATTATCAAAATTCACACTTTGCAAGATAACCAACAATGCCTTTGTGCCCGT</td></tr>
<tr><td>39722315|F|0-67:T>C-67:T>C</td><td>TGCAGCAATGGAGTCGTTGACCAGAAACGAGCCTCTTATCAGATATTGTCGGCCTTTGAGTACTGTGTT</td></tr>
<tr><td>39726106|F|0-14:T>C-14:T>C</td><td>TGCAGCCACTACTTTACAGTTTACATACATATTTTACCATGAAACCAGTTTGTCTAAAATTAGAAAATC</td></tr>
</table>

<table>
<tr><td>39722664|F|0-10:G>C-10:G>C</td><td>TGCAGCCCTAGGTTATCGATTTTCTACTTTTCAAATCCACCCACGCTTCTGTCTTCTTCTTTCATCTGC</td></tr>
<tr><td>39722821|F|0-38:A>G-38:A>G</td><td>TGCAGCGGTGATTATCGAACCATTCGGTTCCCCTTTAGAATCGAAAATCGCCAGTCGAAGTCGTGTGGC</td></tr>
<tr><td>46760984|F|0-12:C>G-12:C>G</td><td>TGCAGGGCCATACCCGATGTTCTGTTGAAAGTGAATTAC</td></tr>
<tr><td>39718951|F|0-11:T>C-11:T>C</td><td>TGCAGTTTTCTTACTTTTCTTTTTTGTGACTATTTTTTGCTGTTTAGAAACAAAAACCTTAC</td></tr>
<tr><td>39717321|F|0-54:C>A-54:C>A</td><td>TGCAGGGCTGGTATTTGTATTTGTACCATATCTAATAAAAATTCAATAATATTGCTAAAATTTGCAAAT</td></tr>
<tr><td>39716885|F|0-26:G>A-26:G>A</td><td>TGCAGGAGAGAATCGAAAGGGCTAAGGCCTTTTATAGACCTGATCTCCAGATTCTCCAACTACCCACCC</td></tr>
<tr><td>39723379|F|0-5:C>G-5:C>G</td><td>TGCAGCCGTACGTTGTTGCCGGTGAAAACGACCGGTTCAACCTTGTTCAGGAACCGTAACTTGAGATTC</td></tr>
<tr><td>39713928|F|0-39:T>A-39:T>A</td><td>TGCAGAAGTCAAAATAATGGAACGTTCAACTCGAATTATTGGTATATAAAATCACATAATAATAGATAT</td></tr>
<tr><td>39720864|F|0-7:G>C-7:G>C</td><td>TGCAGTTGGGACATAGCGAAAAAAAAATCAAGATTAC</td></tr>
<tr><td>46757389|F|0-36:C>T-36:C>T</td><td>TGCAGTGGGTCTCGATACAGTCGACGACTGTGTGGTCCTATTTGGGGCGTCGAAACGCCGTGGGTTTCG</td></tr>
<tr><td>39722656|F|0-53:T>A-53:T>A</td><td>TGCAGCCCGACGCCTACTATATTTATGGCGGAAGACCTCTCCTGCAATCTGCATTTCAAAAAGACATTA</td></tr>
<tr><td>39722613|F|0-66:G>A-66:G>A</td><td>TGCAGCCAGCTTCTTTGTAAAGTTTTCTCTGGATTTTTGCTTTGGAGAAGCTAGAAACTTATAATGTG</td></tr>
<tr><td>39727503|F|0-13:T>G-13:T>G</td><td>TGCAGATTCCCATTTTCATCAAAGGCCACAGAGCAACCAACCACCGCATGGCTTGCTAGAAGCCTTACA</td></tr>
<tr><td>39721395|F|0-51:T>A-51:T>A</td><td>TGCAGAAGGAAGCAATTGCTCTGGAGGACCAATAAAAACTATTCGTGCGTATGTGTAAATATGCTGAGA</td></tr>
<tr><td>39714846|F|0-68:A>T-68:A>T</td><td>TGCAGATCAAAACATAAGGATAGAAAAAAACCCAAAAAGAAAAGAAAAAGAGAATTGAACCCGACGTGA</td></tr>
<tr><td>39713954|F|0-36:C>T-36:C>T</td><td>TGCAGAAGTTGATAGCATCTTGTTGCACATTTATTTCCCTAAATTTTGATTATAATTTTGTTATAGGTC</td></tr>
<tr><td>39718701|F|0-39:C>T-39:C>T</td><td>TGCAGTTCTTTTGTCACACAGCATGTGCATGCATCCCATCTTACTTTGTGCTCTATTCTATTCAACAAC</td></tr>
<tr><td>39725500|F|0-60:T>C-60:T>C</td><td>TGCAGAAAAGCTCATTGTCCTGTTTCTCACCATAGAAATACTAAGGCTCTCGTTCTCCATTCGGACATA</td></tr>
<tr><td>39723897|F|0-33:G>A-33:G>A</td><td>TGCAGTCAAACTACCATGTATGCTTTTATACATGTTTATTCAGAGAGTCGGTCGAAGCTGGCAGCCCAC</td></tr>
<tr><td>39722525|F|0-18:A>T-18:A>T</td><td>TGCAGCATCCAAATTCCCAATGTTGTTGATCCGTACAGTTCCGACTTCGCCGTCCCCGTGGTCGAGTCA</td></tr>
<tr><td>39729453|F|0-14:G>T-14:G>T</td><td>TGCAGAAGAAAGCAGAGGATCATTAGTACAAGGGATTTTATCTTCATATGAGAAGGCTCTTTTAC</td></tr>
<tr><td>39716626|F|0-37:T>C-37:T>C</td><td>TGCAGCTTGGTGAGTTTCATATTCTGTGCGTTATCCTTTTCTCTACTATCATTTGGAGCCTTTGGTATG</td></tr>
<tr><td>46753678|F|0-18:G>T-18:G>T</td><td>TGCAGAAAGGGTTCACATGTCAGTGGTTGTGAACATAAAATTTATTTAGTGAATGGAGAGAGAACATCC</td></tr>
<tr><td>39713650|F|0-48:C>T-48:C>T</td><td>TGCAGAACAACCCAAACCGCACCGTTGCTCTACCGTCGGAACGACTCACTGGATACCGTGCACCTCGTC</td></tr>
<tr><td>46756868|F|0-54:G>A-54:G>A</td><td>TGCAGGTTGGATGGTTATAGGCGCAAGCCCCCCTCATTTGGAGTCGTGTGGCCTGAGCTTGAGCTACTG</td></tr>
<tr><td>39726871|F|0-29:T>G-29:T>G</td><td>TGCAGTTTTATCTCGAGATGTTCATTTTTTAGAGGAATTATCCTGTAAACTGTCATTGAACAAGAAACA</td></tr>
<tr><td>39725086|F|0-22:C>G-22:C>G</td><td>TGCAGGATCACGAAGTCCATTGCCAATAGCAAATCCCTGGCCAACGTATTTAC</td></tr>
<tr><td>39718410|F|0-55:G>A-55:G>A</td><td>TGCAGTGGCAGTGGCCTCTTTTCGGCGTTTCAGCGACTGAAGATTGTGAGTGTCGGCGACAACAAGCAG</td></tr>
<tr><td>39718979|F|0-59:T>C-59:T>C</td><td>TGCAGTTTTTCCTCTATGTTATCCCTTCTAGTATTTCAGTTGAAGGATTGAAACTAATGTTGTGTTTGG</td></tr>
</table>

39726013|F|0-39:A>T-39:A>T	TGCAGCACAGCAGCAACTCAACTCAAATCATGACTTTATATTTACTTTTTCTTTTTGTAAAGGAAATTG
39727713|F|0-48:T>G-48:T>G	TGCAGGCCTTCCTTCAAGATTCAGATTCACTGAATTGAGTGAATATGCTTTTGGCATATGTGTTTTTTA
39722498|F|0-5:C>G-5:C>G	TGCAGCATAATAACACTTCCAAGAGGGTTCGAAACACTATGGCTAGCTTGATTTATCTGGTAAAATTGT
39718313|F|0-44:T>G-44:T>G	TGCAGTGATGGAAGGCCAATGAATCAAGGGGAACAGCAATGGATTTAGTTTTATGAATATTCAATCCAA
39722230|F|0-32:G>C-32:G>C	TGCAGCAAAAGTAGTAGGCTCTTTCAAGGCCAGCGAACCTCGAATTTCCTCACGTAGCCCTTTGATAAA
39722099|F|0-59:G>A-59:G>A	TGCAGATGCTGCGTTGGCTGGAATTCAGGTTGGTTTTTATTTGACTGATAATGTCGTCTGACCTGGTGT
39724866|F|0-11:G>A-11:G>A	TGCAGCAAATAGGTGCAGATGGGAACCTTCTTTCTTTAC
39714653|F|0-42:G>C-42:G>C	TGCAGAGTAGTTTGTCTTCCAGTGAATGCACTGTAGCCCGTAGTTGTGACTTGACTTGTAAACGGTTTC
39715483|F|0-8:C>A-8:C>A	TGCAGCAGCACCGCCACCGCTACCTTCTCTTTTCACGGCATGAAGGATATGAACGCCAGATGAGACAGT
39728287|F|0-5:C>T-5:C>T	TGCAGCTCGCAGCGATCACTTCGGAGGAGCTTGTTGAAGATCCTTTGAAATCGGCAGAGCAGTCCAAGC
39718157|F|0-34:C>T-34:C>T	TGCAGTCTATTTAATATTTATCAATAACCCCCACCCAAAGGCCTCTTGAATACTAAGAATTCCTTTTAC
39718921|F|0-17:G>A-17:G>A	TGCAGTTTGGTAAGCCGGTCGGAGCTGCAAAAGAGGTGGAAATGAATAAGAAAGACACGTTAC
39724377|F|0-61:C>A-61:C>A	TGCAGTTGTGCTCACTTCTTGCATACCCTGATATCAAAGTAGTCCATGCCACCACATCCCGCGCTGGAA
39714240|F|0-7:A>G-7:A>G	TGCAGACAATTTTCAAGAAGCAAATCAGAAAAGCACTCGATCCTCAATCATTTTGCTTCTCTCATGTGC
39728192|F|0-34:G>C-34:G>C	TGCAGAGAGGGACACTTCAACTTTTCTTTATTCAGCAGATGGAAAGTCGCATTTCTCATGCAAAAAAAA
39715809|F|0-51:T>C-51:T>C	TGCAGCCAAAGTGTTTGCATATGTGAATGAAAACTGAGGGTATATATATACTGATAAAATTTTAGGGTG
39718983|F|0-31:T>G-31:T>G	TGCAGTTTTTCTTACAGGTATCTTTCTTGCATTTTTGTTTTGTTTTTATTGCTTTTTTGGAACACTAAC
39722193|F|0-39:T>C-39:T>C	TGCAGATTGTTGGTAGTGCCGAGACGCTATGAGACAATGTCTCAACGCTACCCTCTCGGGTTTGAAGGC
39716136|F|0-45:G>C-45:G>C	TGCAGCGCACCTCTTGGCGCGGTTTGCTGTCGATCTCCATCGGTCGGGGAATTGTTGCTCTGTTTTTTT
39716766|F|0-8:C>G-8:C>G	TGCAGGAACTCGAGCGCAGAAATCATGGGTGCCTCTTCTTTGTTTTTGACTTGGATTTCAAAGTCCCTT
39719710|F|0-14:T>C-14:T>C	TGCAGATCAAAGGATAGCATCCAACAACGAGAACATTGTTCTTAC
39715271|F|0-39:T>G-39:T>G	TGCAGCAAATGGCTTTGGATCCATGGTTATTTGATTTGATTTTTTTTATTTATTTTATTTTTTAC
39726784|F|0-26:A>G-26:A>G	TGCAGTTCAGAAACTCGATGAAGGCCAACTCAAATTATTCAGAAACCTCAAGAGCATTGATACTAATTC
39718792|F|0-51:C>T-51:C>T	TGCAGTTGGGATATTGAGGGACTGACACCATAATTGTTCGTTCCTTGAATACATTTGAGGAACTATTAT
39714394|F|0-7:A>G-7:A>G	TGCAGAGACGATAAGGGCCTAGTGGTTGGGGCTTCGGCCTATTACTATGACATGGACTTTGATGGCCCG
39715159|F|0-19:C>A-19:C>A	TGCAGATTGTTTTCTTCTTCTTTTGTTTTGAATAATTCAAACTTTGTTTTTCTTTTCTTTTAC
39714781|F|0-7:A>G-7:A>G	TGCAGATAGAAAAGAAATGGCTAAAATTGACAAAAATTGGAATGCTTGGAAACAAATCAAATTGGAAGC
39721908|F|0-47:G>A-47:G>A	TGCAGAGTCGTCGATTGACTGTGACGGAAGCTGAGGCATAGTTGACGGGCTGCGATGGCAAAGGTCACT
39721810|F|0-15:G>T-15:G>T	TGCAGAGCGGACGGAGATGCTTTTGGGAAGAGAGATCCGGCGGAGATTATTATTCTTCCCGTCGAACTG

<table>
<tr><td>39713264|F|0-53:T>G-53:T>G</td><td>TGCAGAATCTGCCGAAGATAAAAGTATAAGAAAGAAACTGTTGTCATTATTGATAGCTAAACATGTTTA</td></tr>
<tr><td>39719714|F|0-9:T>C-9:T>C</td><td>TGCAGATCATATAATTGACCATACGAATTTTAC</td></tr>
<tr><td>39719691|F|0-34:A>C-34:A>C</td><td>TGCAGATACTTATTTCTCTTCTTTAGTTTTTTCCAATGTGCTTAC</td></tr>
<tr><td>39726690|F|0-65:G>T-65:G>T</td><td>TGCAGTCTGTAGTATTATTACACTTCTTCTACTATAGAATGGCCAGACTTGGAGTTCTCCCCCTTGTGG</td></tr>
<tr><td>39720787|F|0-23:T>C-23:T>C</td><td>TGCAGTGTTTGCCTATTGAGATTTAGCCCGCTGACTTATTTACAAAGGCTTTAC</td></tr>
<tr><td>39723630|F|0-53:C>G-53:C>G</td><td>TGCAGGTGCCTTTGACAATAGGGCAAGTCTCAAGTTTTGCCTTTGACAATAGGCCAAGTCTCAAGCTTT</td></tr>
<tr><td>39718257|F|0-22:C>G-22:C>G</td><td>TGCAGTGAATAAAGAGATGATCCAGAGATTCCTCTTATTTTCCGCAAAGGACCCATCTATTGGGACGCA</td></tr>
<tr><td>46754560|F|0-68:A>T-68:A>T</td><td>TGCAGATAAGGTAAGAAAATGAACTACTTTTGGAAGAATTTTTCTCGTAGCATATTCATTTTTTTTTA</td></tr>
<tr><td>39727545|F|0-7:C>A-7:C>A</td><td>TGCAGCACGCGTCACAAAGTCGTCTCTTGAAGAGACGATCATGGGTGTAATGATCTGCCAAAAGAGATT</td></tr>
<tr><td>46757296|F|0-50:C>A-50:C>A</td><td>TGCAGTGCAAATATTTTTTATGTGAAGCATGTCTACAAGATTTGAGTAAGCTAAATTTGATGGTGAAGA</td></tr>
<tr><td>39721251|F|0-26:C>T-26:C>T</td><td>TGCAGAAATTTCAAAAATAGTGTGTGCGTGTGTGTGAGAGAGAGACCAAAAAGGTTTCCACGAGACAGC</td></tr>
<tr><td>39723706|F|0-32:G>C-32:G>C</td><td>TGCAGGTTTTTATATGTAGTGAAAACAGAGCAGTCAAAGGTTGATGATAATGACAAAGAACAATGTATT</td></tr>
<tr><td>39727390|F|0-27:G>A-27:G>A</td><td>TGCAGACAACTCCAATACCCGTAGATGGAGGAGAAGGTGCTTGCCCAAGCAGCATCTATTCGAATTCAT</td></tr>
<tr><td>39707645|F|0-26:A>G-26:A>G</td><td>TGCAGACACGATTTACTTCCATTTCCATTCCCCTTCATTTTTAGTCTCTGGTTTCCTTTCTCTTTGGAC</td></tr>
<tr><td>39721668|F|0-46:A>G-46:A>G</td><td>TGCAGACTGGAGAAACTAGTACATGACTCATTCAAAGGGTAAACCTATAGAAATCTATAGTTATTTATT</td></tr>
<tr><td>39725631|F|0-26:G>T-26:G>T</td><td>TGCAGAATTATGATTGAAATTGGGAAGAAAAAAGTAGAGAATATGACGTATCACGTGATGTGTGACTGT</td></tr>
<tr><td>39716095|F|0-62:A>G-62:A>G</td><td>TGCAGCCTTTGGGAATCAAACTTGGGAAGGGGAGAAACACATGTCACTCCTACACCACACAGACAATAC</td></tr>
<tr><td>39723748|F|0-12:G>C-12:G>C</td><td>TGCAGTAAGAGTGATAAAAGATGATGATCCTAAGTGGGAGCCAATTGTTTTGCCAGAAGGATCTCCGGT</td></tr>
<tr><td>39728489|F|0-25:G>A-25:G>A</td><td>TGCAGTCATCTAAATCTAGATATAGGTCATGTTACATTACAAACATTAC</td></tr>
<tr><td>39716959|F|0-26:G>C-26:G>C</td><td>TGCAGGATAGCCACACACAACTATTAGTATTACCTATAAATAGTTACCTACATAAATATAATGTTACCA</td></tr>
<tr><td>39720077|F|0-24:T>A-24:T>A</td><td>TGCAGCCTTATATGGATGTTTGGCTTTTTTTTTTTAC</td></tr>
<tr><td>39725907|F|0-36:G>C-36:G>C</td><td>TGCAGATTCAAAAAATAATAAGAAACGAGGAAACAAGGAAAAAATTGATATATATATATATATATAAAT</td></tr>
<tr><td>39717075|F|0-61:A>G-61:A>G</td><td>TGCAGGCAGATTTTGTTGGCATTCTTTTCAGCTAGGTTCTGGCTCAGCTCTGCCCTCCCAAACTCAGTG</td></tr>
<tr><td>39724483|F|0-30:C>T-30:C>T</td><td>TGCAGTTTTTGTTTTCTAGGCCCCTCCTGTCGATCGGTACTTGCACGATTTACTTCAATATGCATTGGG</td></tr>
<tr><td>39724793|F|0-39:T>A-39:T>A</td><td>TGCAGATATGAAATTTATCAAACAGATCATTACCATGTATTTTAC</td></tr>
<tr><td>39714627|F|0-32:A>C-32:A>C</td><td>TGCAGAGGTTATGGGCTGCTATAGGAAGTGGCACATGGGCTTCTATACTGTGTCCAATGAAGTTGTCAA</td></tr>
<tr><td>39720022|F|0-9:T>A-9:T>A</td><td>TGCAGCCACTCGTGGTGGATTTATTAATTAC</td></tr>
<tr><td>39724976|F|0-24:A>T-24:A>T</td><td>TGCAGCCTACCAATCACATCAACTACCTAGAAGCATTTCTTATTTTCTTAC</td></tr>
<tr><td>39722108|F|0-45:T>C-45:T>C</td><td>TGCAGATGGCAGATATTTGACTATATTGGACTAATATCGTTAGGATGTCATCTGCCTATAACTAAAAAT</td></tr>
</table>

<table>
<tr><td>46761077|F|0-28:T>A-28:T>A</td><td>TGCAGTGTGTTTAAGAATGGCATTGGAATAGATTAC</td></tr>
<tr><td>39722500|F|0-19:G>A-19:G>A</td><td>TGCAGCATAATTAGCTTAGGTAACCTTGTTTCTTCTGGTGTAAAATTGCTTGTGGTGGTGAAATAGATG</td></tr>
<tr><td>39721914|F|0-23:G>A-23:G>A</td><td>TGCAGAGTGAAAATGAAATACACGACGAGGGTTTGTTGATTAGATGATAGTGATAGTGTATTTCATTTG</td></tr>
<tr><td>39718569|F|0-37:A>G-37:A>G</td><td>TGCAGTTAGGTAGGGAGTGTGTACGTGGGGAGTTACAAATGTAACTTCTCCCTCTCTCTCACGTAAATG</td></tr>
<tr><td>39728702|F|0-25:T>C-25:T>C</td><td>TGCAGAAGTATGAATAAATTCTCTATCCTTATAGCAAAATCAAGTCAAAAGCTAGAATTTGATATTTAC</td></tr>
<tr><td>39716428|F|0-27:G>A-27:G>A</td><td>TGCAGCTGCCAGACTTTCCATTCTTGAGCATTGCAATATTCTGATTCTTTTTCCATCAAACCCGCTATT</td></tr>
<tr><td>39728409|F|0-38:C>T-38:C>T</td><td>TGCAGTTCCCTTTGTTCTTTCTTATTGATTTCCCAAGTCATCTTCTCAAGTCAAATCTGATAAACATCT</td></tr>
<tr><td>39722861|F|0-25:C>T-25:C>T</td><td>TGCAGCTAGAATAAATAAAGTGGGACACTAAAAAACTATGAACTACACAATGATTTGCAATGCATGTAA</td></tr>
<tr><td>39717975|F|0-24:T>G-24:T>G</td><td>TGCAGTATTCTTGATGCCAGCCTTTGGAGGTCCTCATCCTATCTTTGGAAGAGCTTGCTTTGGGGGAGA</td></tr>
<tr><td>46755761|F|0-18:G>C-18:G>C</td><td>TGCAGCTACTCGGTCCTTGGCTAGGGACACAAACTTCAATGCAATAATTTCTATGCTGAAGGGGTAACA</td></tr>
<tr><td>39719432|F|0-12:C>A-12:C>A</td><td>TGCAGAAGTTACCATGACTAAACTTGTGATTGATTCAATTAC</td></tr>
<tr><td>39719880|F|0-10:A>G-10:A>G</td><td>TGCAGCAAGCATTGACATTTCCAATGGTAATTGTATTTAGTTTCTTAC</td></tr>
<tr><td>39726416|F|0-7:A>G-7:A>G</td><td>TGCAGGCAAGTGCCTATGATGAGAGCGTGAGGCGCCTAGCAGGTATTGCACCTCTTGTCATCTTGCGTT</td></tr>
<tr><td>39717084|F|0-44:T>A-44:T>A</td><td>TGCAGGCAGTACATTGGCTCTAACGTTTTGGCGCCATTGCAAAATGTTTCAAAGTCAGCTCCAGATGAG</td></tr>
<tr><td>39714427|F|0-48:T>C-48:T>C</td><td>TGCAGAGAGGCATACATAACACAACAAAATATAAGGATAAAGTATATATATACTCTTCGACTATGAGGT</td></tr>
<tr><td>39713408|F|0-33:A>C-33:A>C</td><td>TGCAGAAAAATGCACAATTTGTAAATGCAGCCCACATGAATTACTCTATTATAAGGAACTTATCATATA</td></tr>
<tr><td>39721940|F|0-25:T>A-25:T>A</td><td>TGCAGATAAGTTGATTTGATGTCTTTCAACCAAAAGTTCTCAGACTTCAGAATAACAAGAATTCAATGA</td></tr>
<tr><td>39728189|F|0-18:A>C-18:A>C</td><td>TGCAGAGACTTCTTCAGAACCTGAGACTAGCATCTCAGGCTTTATGGTTGTCAAGCCATCCATGGATGC</td></tr>
<tr><td>39722426|F|0-6:G>A-6:G>A</td><td>TGCAGCGGCAGCAGCAGCAGCCCAACAAAACAGGACGCCCAATGTCTTCCAACCATGGTCACACAAAAT</td></tr>
<tr><td>39724190|F|0-43:G>C-43:G>C</td><td>TGCAGTGTTTTGCAGGAGCACTTATCTAGTGTTTATGTAATGTGTTGTACACTTCTGGTGTTAC</td></tr>
<tr><td>39724639|F|0-16:T>C-16:T>C</td><td>TGCAGAAGACTCCACATGCATATGAACAAGTGTTCTTCAATCGGAATTTAC</td></tr>
<tr><td>46757511|F|0-42:C>G-42:C>G</td><td>TGCAGTTCAATTTTTTTCTCCTTTTCTTTTCACCGCCTGAAGCTCGTTTTTTTTCTCTTTACACTTTTT</td></tr>
<tr><td>39722003|F|0-57:G>A-57:G>A</td><td>TGCAGATCATACACAAAATGATCGAGTGACTTGTGAAGATCGAGTTATGTTTGAATAGTCACTACAACA</td></tr>
<tr><td>39725276|F|0-13:G>A-13:G>A</td><td>TGCAGTCGCTATCGTTGGAAAAATCTGTAGCTGTTAC</td></tr>
<tr><td>39725775|F|0-40:T>A-40:T>A</td><td>TGCAGATGATGTAGAAGATTTCGAAGGAGTGGATGCAGAATATGTTACATCGTTTCGTTGGTATAACAA</td></tr>
<tr><td>39724872|F|0-29:C>T-29:C>T</td><td>TGCAGCAACATCTCAATCCATTACCCCTCCGAACGTCCACCGGAACATACCTCGTTTAC</td></tr>
<tr><td>39723759|F|0-60:A>G-60:A>G</td><td>TGCAGTAAGTTCACTTTGCCCACCCCAAGAGAGAGAGAGAGAGCACTCTTTGTTTAGTTGAGCTTAGAA</td></tr>
<tr><td>39721981|F|0-57:G>C-57:G>C</td><td>TGCAGATATGTTTACCAAAACACTTCCGATTGATAAGTTTGACTTATGTATGAGAATGTTGCATATTGA</td></tr>
<tr><td>39713297|F|0-43:T>G-43:T>G</td><td>TGCAGTGGTGCGTTTCAGCCCAAATCTGTGTTTCCACAAGCATTTTCTAGCCTAGATCTGGTGCTCGAC</td></tr>
</table>

<table>
<tr><td>39717426|F|0-27:C>T-27:C>T</td><td>TGCAGGTAAATTCAACAAAACCCGTATCGAAGTCAGATCAGGTTCAGCTATATCTATACCATGAATTCA</td></tr>
<tr><td>39716596|F|0-22:C>G-22:C>G</td><td>TGCAGCTTCTGACATTTTCGCACAGAGGAAGGAAGAGCTCGAGAGAGAGAGAGAGAGAGAGAGAATGAA</td></tr>
<tr><td>39724375|F|0-15:T>C-15:T>C</td><td>TGCAGTTGTCAAAATTAGAAGTTGCCTTCTCGCATTTTCCAAAAGAGTTGCATTCTGCTTCTTGATTGT</td></tr>
<tr><td>39722492|F|0-14:A>G-14:A>G</td><td>TGCAGCAGTTGGGGAAGAGGCATATTGTTGGGTTTCGTCTCTGGATGAGGTCAGCTGTGTTGATACATG</td></tr>
<tr><td>39722212|F|0-44:A>G-44:A>G</td><td>TGCAGATTTTGATGAATTGCAGTTTCACATAATCTAATTCCCTTACTCACTTTTCCAGCTGATGTTGTG</td></tr>
<tr><td>39728998|F|0-31:A>T-31:A>T</td><td>TGCAGCAACAAGTTCCTAGACATGTCTCCTCATCACTTCCCCCACTTCTTTTACTATTTTACCCTCCCT</td></tr>
<tr><td>39721655|F|0-21:T>A-21:T>A</td><td>TGCAGACTCAAAACGAATCAGTACAAACACCACAATAACTAGCATATAAGAACAAGGAAGAAAACATGA</td></tr>
<tr><td>39724400|F|0-35:A>T-35:A>T</td><td>TGCAGTTTCAAACATAACAACAATAAGTAAATAGTATTGAGCTCCATAGCTTTACTACTTTTCACTATT</td></tr>
<tr><td>39719881|F|0-21:C>G-21:C>G</td><td>TGCAGCAAGCTGTGGTGGGCCCTCCCCCATGAAAAGTTAC</td></tr>
<tr><td>39716723|F|0-38:T>C-38:T>C</td><td>TGCAGGAAACGGAGAATTTTGAAGCAGTCCACATAATCTTGGGCCCAAGCCCAAACATTTTGATTCGGC</td></tr>
<tr><td>39728493|F|0-17:G>A-17:G>A</td><td>TGCAGTGAATTGTGAGCGAAATGAAAATGAAAAAAATATGTGCAACCAAGTTTTAC</td></tr>
<tr><td>39723264|F|0-27:G>C-27:G>C</td><td>TGCAGGATGGGTATTGGAGTGTTATAAGGAAAAGGAAAAGAAAAGAAATGCAGGATGGAAAAGGGAGGG</td></tr>
<tr><td>39718099|F|0-37:C>G-37:C>G</td><td>TGCAGTCCCCTTCCATCGTTGGTGCTGCATAGTTAGGCCGCCCATCAAAGAAGAGTCTAATACTCTCTA</td></tr>
<tr><td>39717466|F|0-50:G>A-50:G>A</td><td>TGCAGGTATAGAATATTTTAGGTATGATAATGAAACTATGATGTCTTTCTGCTAATTCTACGATGTTGT</td></tr>
<tr><td>39722948|F|0-68:T>C-68:T>C</td><td>TGCAGCTGATGATGTACCTGAATGCCCTTACTCTCTAATTGCACAAAATGCAAATGAAATCTTTTTTTT</td></tr>
<tr><td>39717331|F|0-13:A>G-13:A>G</td><td>TGCAGGGGAGAATAATGCTGTTATCCTATTCTGGTTCCTAGTACTAACTACTAAGTAAATAAATATCCC</td></tr>
<tr><td>39716694|F|0-68:G>A-68:G>A</td><td>TGCAGCTTTTGTTGACTAAAGTTGATTTTATTTGGGAATCTGAAGTGTGCATTTTCTTTGTTATATCTG</td></tr>
<tr><td>39716630|F|0-68:A>G-68:A>G</td><td>TGCAGCTTGTGCTTGATGCAGAACAAGCAAGGACACAGACACACAAAAAGAGCAGAAATAAAATGCTCA</td></tr>
<tr><td>39725083|F|0-38:A>G-38:A>G</td><td>TGCAGGATAGCCGCCTACAAAAAGAATCAACAAATAATATGCCTAAACTATTAC</td></tr>
<tr><td>39719368|F|0-35:C>T-35:C>T</td><td>TGCAGAACTAAAAGTTTTCTTTTTTTGACTAAAAGCTTTAC</td></tr>
<tr><td>39720348|F|0-38:T>G-38:T>G</td><td>TGCAGGATCAAAGCAAAATTCATCTCATGTGAAACACTTTTTTTACTATGTAATTAATTAC</td></tr>
<tr><td>39725997|F|0-42:C>G-42:C>G</td><td>TGCAGCAATACAACGACTTGAAGAACAAGTTCTCTCAAAGGGCCATTTTTTATTTTACTTTTTTTTCTT</td></tr>
<tr><td>39726649|F|0-24:A>T-24:A>T</td><td>TGCAGTCATGACACAACTTCAGCCATCTACCACATATTCTTACAAATATGGAAGGTATTTAC</td></tr>
<tr><td>39722279|F|0-65:G>A-65:G>A</td><td>TGCAGCAACTAACAGCAGCACCTAAGAAATGGAAGATACCACATCTAGCTGAGCTAGAGATAGTAGTTC</td></tr>
<tr><td>46753663|F|0-62:A>C-62:A>C</td><td>TGCAGAAAGAGATAAAATAACACTTTTTTTTTCCGGTTGAAATATATGAAAAAAAAAGGAAGAAGCCAG</td></tr>
<tr><td>39721587|F|0-14:G>A-14:G>A</td><td>TGCAGACATCAACTGCTCCATCTAAATTGCAGAGAAGCGTCAGAAAGGAAAATCCAGTTGATTTTGAGG</td></tr>
<tr><td>39716005|F|0-6:T>C-6:T>C</td><td>TGCAGCTGGCTGCTGAGGTTCGTGCTCTCCTTTATGGTCTACAATTTTTGCAGCGGTTGCACATCATGG</td></tr>
<tr><td>39716206|F|0-5:T>C-5:T>C</td><td>TGCAGTGTCGAGGGGTCGATTATGGCAGCTTCAGTCTTCTCCAGCGCGCGTGGGGTTCATTTTCTGGCG</td></tr>
<tr><td>46757577|F|0-43:T>A-43:T>A</td><td>TGCAGTTCTGGAATTTTTTTTCCGTTTGTTTTTTCGCTTGTCTTTGTTTTTGTGTTTTTCTTTTTCGTT</td></tr>
</table>

<table>
<tr><td>39714547|F|0-16:T>C-16:T>C</td><td>TGCAGAGGAAGAAGATTCTTCGAAGTCTTGAGTCGTCGTGCGGCGGCGGGGGGCCGGAAAATGGGGCGG</td></tr>
<tr><td>39722358|F|0-43:T>C-43:T>C</td><td>TGCAGCACCTCCCGCTGCCGGAGATAGACCGACGACCCCACCGTCGGCCCGAAATCTCCGTCGTCGCAG</td></tr>
<tr><td>39729022|F|0-17:T>G-17:T>G</td><td>TGCAGGCAGCAACCTTGTGAAGACCATCTGTCTATGGTGTTCTCAAGGTGTAATTATAGTTTTTACAGA</td></tr>
<tr><td>39728495|F|0-43:T>C-43:T>C</td><td>TGCAGTGTACTTGGGAATTCAGTAATTATAGTTTCTGCAATTTTTTTAC</td></tr>
<tr><td>39713872|F|0-36:C>G-36:C>G</td><td>TGCAGAAGGAGAGGAAAGGAACGGAGAAAAGTGAAACCCTAGAGTGAAGTGAAACGCAGGCGGCGGCGG</td></tr>
<tr><td>39726680|F|0-33:C>T-33:C>T</td><td>TGCAGTCGAGGGAGTCCAACCCTACTACTCTCTCGTCGTCGACTGAATCCAATGAGAATGCTAGTGTCA</td></tr>
<tr><td>39728776|F|0-23:A>G-23:A>G</td><td>TGCAGTCCAGTTGAGCACGAGCAATTCCTCTCCCTCGACCTCAACCTCAAGCCCGTCCGATTATTTTCA</td></tr>
<tr><td>39725281|F|0-24:G>A-24:G>A</td><td>TGCAGTCTAATGGAGAATCTTTATGGAGGTGTTTTTTTTAC</td></tr>
<tr><td>39715896|F|0-55:C>T-55:C>T</td><td>TGCAGCCATGACTCTCACCATTGCCCCAGAAAAAATCTCTAACCAACTTCTCCATCTGCCTAATGACTC</td></tr>
<tr><td>39717805|F|0-44:A>T-44:A>T</td><td>TGCAGTAATTGGAAACCAATGAATTTTTTGAAATGGGTGAGTGCATAGAGATTGTTTTGAGGGTAAGGA</td></tr>
<tr><td>39723762|F|0-58:T>C-58:T>C</td><td>TGCAGTAATCAGGTGACTTAGAGCAATGAAGACATGAGAGACCTATTGAATCTTCTTTTGCATGTAGTT</td></tr>
<tr><td>39726253|F|0-61:T>C-61:T>C</td><td>TGCAGCTCATGAGGCGACGGCGAAATCAGCCGCTTCCCGACGCCGTTCGTTGGTTTTCCGGTCGCCGTC</td></tr>
<tr><td>39727943|F|0-13:G>C-13:G>C</td><td>TGCAGAGAGTGAAGATACTCCAGCGTCCGAGCTCTCACCGGCGTTGTGCTATCGTCATGACATATATCT</td></tr>
<tr><td>39722095|F|0-59:C>T-59:C>T</td><td>TGCAGATGCTATTTCAGAGGACCCGAATAATGTTGGCAGAGCAATGGTATGTTTTATCTCATTTATTTA</td></tr>
<tr><td>39721335|F|0-14:C>A-14:C>A</td><td>TGCAGAAGAAGGTACTCTTCATACTTTCCTTCTCTTTTAGTTCAACCATGAGGGAATCGAAACTCTAAC</td></tr>
<tr><td>39728701|F|0-7:C>T-7:C>T</td><td>TGCAGAACGTGTTAGGTCTTGTGAGATCCATGTATGCCTTGATCTGCTTGGAGGAAGATGCTTCGTTCC</td></tr>
<tr><td>39714393|F|0-15:A>G-15:A>G</td><td>TGCAGAGACGAATCAATATAAAGCTCTAACAACTCGGTTGAAGGAATATGCATTTACCAATTGAGTTAG</td></tr>
<tr><td>39727190|F|0-37:A>T-37:A>T</td><td>TGCAGTAAAGTACTCTACTAACTTATATGAGTGAAGAACATGCATAAAGTTAC</td></tr>
<tr><td>39729356|F|0-51:T>C-51:T>C</td><td>TGCAGGTTAGTTCACCCTTTTCAACTTCTAGAATTCATCCTCCATTACAATTTGATTGCAATTAC</td></tr>
<tr><td>39723127|F|0-32:C>A-32:C>A</td><td>TGCAGGAAATGTACAGACAGGATACAATAGAACCAAAACCTTAGCACATAGCAAAGCCACACAATAGTG</td></tr>
<tr><td>39713759|F|0-28:G>C-28:G>C</td><td>TGCAGAAGAACACAACTGCCTTGTAAGTGAACTCTTCCTAAGATTCTTACATATTTTGATATGCAGTCG</td></tr>
<tr><td>39727463|F|0-10:T>A-10:T>A</td><td>TGCAGAGTTTTGTGGTTTTTTACCTCCAAACACATTTTCAATCAAGCTGCCATCAATAAGATCACAACT</td></tr>
<tr><td>39725479|F|0-30:G>C-30:G>C</td><td>TGCAGAAGAAAAACTCTGTTACTAACTAATGGATTTACAAATATAGAAATGTGGGAACCGATTTTGCAA</td></tr>
<tr><td>39717487|F|0-61:T>C-61:T>C</td><td>TGCAGGTATTTTTCCCTTACTAATTGCAATGTAAGAAGGCATCATGCACATGATCAGAGGCTGAAGAAC</td></tr>
<tr><td>39723147|F|0-35:T>A-35:T>A</td><td>TGCAGGAAGGTTACCTTGTCTATGAGTGTAATGAATGGTGGAGCTGTAGTAGAACCTGTCCAAATAGAG</td></tr>
<tr><td>39715844|F|0-26:T>G-26:T>G</td><td>TGCAGCCACCATCACCGGAAAAATCATGTTACATGCCCTCTTTATTCGACTCACTCATCCAAATCCACA</td></tr>
<tr><td>39718521|F|0-29:T>C-29:T>C</td><td>TGCAGTGTTGTTTCTGAAGGGATTGAATCTCCAAAAGAAGCGCATGTAATCACGTGTGATTCAAATTGT</td></tr>
<tr><td>39714894|F|0-44:C>T-44:C>T</td><td>TGCAGATCCTATTTGGACCCCTCGTAGACTGATTGACAAGAATACGGTTTATACTATATGGCCATCATT</td></tr>
<tr><td>39713737|F|0-56:G>A-56:G>A</td><td>TGCAGAACTTACATCACTAGACAAATTTCAGATAGGTCAATTCAGGGTCGAGTTCAGGCTCAAGAGATT</td></tr>
</table>

<table>
<tr><td>46771725|F|0-63:G>T-63:G>T</td><td>TGCAGACCCACTTCTCGCCACTACCCACTTCATCACCAGCCACTACAACTGCAAGGTTTTTTTGTTTTT</td></tr>
<tr><td>39721323|F|0-39:A>G-39:A>G</td><td>TGCAGAAGAAAGAATATGAAGTGAGAAAGAGAGAGACGAAGTGAGGACTAAAGAAAAAAAAAGCAATAAA</td></tr>
<tr><td>39723936|F|0-39:G>C-39:G>C</td><td>TGCAGTCCAAAATGATGGTTTTGGATGTGCTTCCTTATCGTCAGCCGCAACTTTTCTTCGGCAAAGCTC</td></tr>
<tr><td>39723936|F|0-44:C>T-44:C>T</td><td>TGCAGTCCAAAATGATGGTTTTGGATGTGCTTCCTTATCGTCAGCCGCAACTTTTCTTCGGCAAAGCTC</td></tr>
<tr><td>39724823|F|0-10:A>G-10:A>G</td><td>TGCAGATGACAGTTGTATTTTTTTTTCTTTTTTAC</td></tr>
<tr><td>39719754|F|0-28:C>G-28:C>G</td><td>TGCAGATGAGAAGCAACCACCCACACTCCTGCTAAATTTCAGGGAGTGAGTGGTTAC</td></tr>
<tr><td>39717842|F|0-49:C>G-49:C>G</td><td>TGCAGTACCTTTGTTAGGGGGAAAATGCAAACTTCAACCCCCTCTAGTCCAAGAAATATTCCCCAATGC</td></tr>
<tr><td>39713921|F|0-64:G>A-64:G>A</td><td>TGCAGAAGTACTTAGGCAAACATCTGCAAGTCGAGGGTTCTTCAGCTCATTCGACTTTGCCTACGAATT</td></tr>
<tr><td>39719300|F|0-13:C>T-13:C>T</td><td>TGCAGAAACTTTACCAGAAAGAAGTGCGTGAATTTTCAAGGTGAACCTTTTTCATTAC</td></tr>
<tr><td>39723039|F|0-46:A>C-46:A>C</td><td>TGCAGCTTCTTGGGATTCAATCTGCCAAGTAATTCAAATTAGTAGTATTTGAAATTATTCAATTCAATT</td></tr>
<tr><td>39721655|F|0-30:A>C-30:A>C</td><td>TGCAGACTCAAAACGAATCAGTACAAACACAACAATAACTAGCATATAAGAACAAGGAAGAAAACATGA</td></tr>
<tr><td>39726133|F|0-45:G>A-45:G>A</td><td>TGCAGCCCCAAATTACCCTAACCAAAACATGGAACATAAATATCAGCATTAGATCCTTACTGAGCTTAG</td></tr>
<tr><td>39716459|F|0-68:T>G-68:T>G</td><td>TGCAGCTGGACTCAAAGCAGCTGTAAATTCTTATCCTTCCTCTGATCTTTTGCAAACTATTATGTATGT</td></tr>
<tr><td>39716510|F|0-53:C>T-53:C>T</td><td>TGCAGCTGTTCTTTTTGTTCATGGTCTCTTCTCTCTCAAACGAAGGCTTAGTGCCCGTTTGGCGTTGCT</td></tr>
<tr><td>46762442|F|0-33:A>G-33:A>G</td><td>TGCAGAAATTGCGTAACCGAACTCGATGATGACATCAATGAGGTTTATTTTGTGATTGTTGCGGACAAT</td></tr>
<tr><td>39721745|F|0-53:T>A-53:T>A</td><td>TGCAGAGAGCAACATTCATATTTAGCTGGTCAAGTACAAAAGCCAAGCTTACCTAAAAGACATATTCAA</td></tr>
<tr><td>39727757|F|0-8:T>G-8:T>G</td><td>TGCAGGTCTCTGAAGGGCAGCCAGAATTAGTTCAATTTTTTTTTCACTCACTTTCTCTCTCCTCATTCC</td></tr>
<tr><td>39715883|F|0-32:T>A-32:T>A</td><td>TGCAGCCATAGTAATCCCCCAAGGATAATTTCTTTGTGAGTGCATCGTTTTGACTTAGCAAACTCTTCT</td></tr>
<tr><td>39727039|F|0-24:A>G-24:A>G</td><td>TGCAGATTATGATTGTGGGAGATAAGAAGATAAAACTTTCATATTCATTTAC</td></tr>
<tr><td>39724395|F|0-66:C>A-66:C>A</td><td>TGCAGTTTATACCTAGAAGCTAGGGATCGAAACAAATCACCGACAATCTCTTCTGATGTTTATTATCAA</td></tr>
<tr><td>39714975|F|0-50:C>T-50:C>T</td><td>TGCAGATGATTATACCTCCCACAGGCCCGGCAGCCATATGAAATATGAAACAATAATCAATGAATGTAA</td></tr>
<tr><td>39715253|F|0-13:C>T-13:C>T</td><td>TGCAGCAAAGAATCGACCGCCATGGCCGAAGTAGAAGACGACACTCCAAGAACAGATGCTCTCTCCAGG</td></tr>
<tr><td>39719727|F|0-45:G>A-45:G>A</td><td>TGCAGATCCACAAGAACAATGTCCCGCTATATCACTGAGAAATGGGAAACAATTAC</td></tr>
<tr><td>39728902|F|0-65:T>A-65:T>A</td><td>TGCAGCTGTCTATGAGGCAAAAGAGAGAATATTCAAATAGCATCTTCAATAAAAAAAAAAAAAAATTAGA</td></tr>
<tr><td>39722420|F|0-5:C>T-5:C>T</td><td>TGCAGCAGCAACCTGATGAACATGTGCATATACACATGATGTAAGTGGGATGGACTAAAAGAGGAAAAA</td></tr>
<tr><td>46755841|F|0-10:A>G-10:A>G</td><td>TGCAGCTCGCACGTCTACATTGAATTGGCCGGAAAACACTGCTGAATAAACATAAAAATGAGTGGGTAT</td></tr>
<tr><td>39716257|F|0-9:T>C-9:T>C</td><td>TGCAGCTACTGTGCGAACCTGCAAGATCAACATGGAATGATATTTCCAACAAGAAAGTACCCAACTCAC</td></tr>
<tr><td>39726650|F|0-35:T>A-35:T>A</td><td>TGCAGTCATGACACAACTTCAGCCTTCTACCACATTTTCTTACAAATATGGAAGGTATTTAC</td></tr>
<tr><td>39714441|F|0-5:G>A-5:G>A</td><td>TGCAGGGATAGATGTATGAAAACTAACTCTAAAATCAAAACCAAATGCAGCATGGTATCGAGTTGAATT</td></tr>
</table>

39713966|F|0-33:G>A-33:G>A	TGCAGAATAATGATAATAACATGAAGAGGAAGAGGAAGAAGAAGAAGAAGAAGATGGGCTTATGGGTGG
39716048|F|0-34:C>G-34:C>G	TGCAGCCTCTCTCCCCTGGCTCCTCGTCGTCGTCCTCGTCCTCGTCTTTGTCGAGGGAAACGGGTAGAG
39728632|F|0-28:C>T-28:C>T	TGCAGTTCTGGTATCTTTGATTGAGGTACGATATTGCTCTACTCTAGCAATATATCATAATTCTTGCTC
39718037|F|0-16:G>C-16:G>C	TGCAGTCACTGATAGTGAATGGTTTCTCTCACAATTTGTGCAATTCACAAGATCTCTCAACTCCCAATC
39726548|F|0-62:T>G-62:T>G	TGCAGGTTATAGTATTCATTTACAAAGCTCAGGTTATAGCTTTCAAGTGATTTTTTTGAAAATGATTAA
39715160|F|0-59:A>G-59:A>G	TGCAGATTTAAAATCTTTAGAAGTTCCTGCTTCCACCAAAAGATAGTCAACTGTCAAAAACAAAAGTTC
39722221|F|0-47:G>A-47:G>A	TGCAGCAAAAATATCTATATAGCATATTTTTGCATTTTTTAGAAATAGGTTACAGACAACAATTTTGTC
39726318|F|0-34:C>A-34:C>A	TGCAGCTTATACTGCCCTTTTGTTCATAGTTCCCCTCGGGAGAGAGACGAGCTTGAACCCTCTGTCAAT
39722775|F|0-8:A>G-8:A>G	TGCAGCGAATCCATTGCGTGAATATGCCGTCGTCGAGGTTGTTATTGTTTTCGGAGACAAAGGAAATCA
39722221|F|0-17:T>C-17:T>C	TGCAGCAAAAATATCTATATAGCATATTTTTGCATTTTTTAGAAATAAGTTACAGACAACAATTTTGTC
39721163|F|0-26:T>C-26:T>C	TGCAGAAACACCTTGGAAAAAAACAGTGGTGTTAGAAGGATCAAGATTGGAGTCAAGCCATTTTCCCCA
39721875|F|0-14:A>T-14:A>T	TGCAGAGGGAGGGAAGTGGCGGAGGGTGACGATGAAGGCCGGTGGCAGCGCGCGTGTCTGCTCCATAAA
39723049|F|0-24:C>G-24:C>G	TGCAGCTTGCTGTCCCTAGATCAGCGCAAGATGCCCTTCTAGTCCGTCTTTGCGAATCTAAAAAGGAAA
39727538|F|0-13:T>G-13:T>G	TGCAGCACATATATGCAAGATTACATAATTTTCTTATATACATATGCCGCAATAACTTTTGGCTCCTCT
39728946|F|0-26:A>G-26:A>G	TGCAGCGTTTTGTTCGTTGGAGCGTCATTGGCGAAGAACAGTCAAGTCTACAACGGAGGTTTGTAACGG
39721169|F|0-57:A>G-57:A>G	TGCAGAAACAGTTGCAGAATACCACACAATCTCAATACAAGATGAAGTCATTCAAATATATAGGCTTTC
39728702|F|0-36:A>T-36:A>T	TGCAGAAGTATGAATAAATTCTCTATCCTTATAGCAAAATCAAGTCAAAAGCTAGAATTTGATATTTAC
39718148|F|0-60:A>G-60:A>G	TGCAGTCTAATAAATACAAAAAAGAAAAGTACTAAAAAAATACAAAAATAAAAGAGTGACAATACAAAA
39718651|F|0-34:T>C-34:T>C	TGCAGTTCGATGAGGCTCAAGTCTGATGAGGTGTTACTCTTGCTCCGACACTCGAAACGACGACTCTAT
39727994|F|0-20:A>G-20:A>G	TGCAGAGTATGGTGTCCCACAGAAATTTTGCATTATAATTTTACTTTTTAC
46760665|F|0-44:G>A-44:G>A	TGCAGCTATTAAGAGCAGTGCATGAAGCTTCTTTGTTTAGTGAGGATAATAATTTGGTTCCTCTTTGCT
39723873|F|0-37:C>A-37:C>A	TGCAGTATGCCTATAAGACAATCAACTCATAATTTACCATTAGCTTGTGCTATTAGTTTCACGAACTGC
39725362|F|0-7:G>C-7:G>C	TGCAGTTGAGGGGCATGTTTTAGAATGTAATTAC
39717797|F|0-13:G>A-13:G>A	TGCAGTAATGGATGGCCTTCCTCAAAATTCAGGAGAATGATAACAGAATATATCATTTTTAC
39717206|F|0-15:T>C-15:T>C	TGCAGGCTGTAAATTTTGCCTGCTGTACTTTCCTTTTCGTTGCCTTATTACCTATTAGGTTTATGCATG
39718527|F|0-44:G>A-44:G>A	TGCAGTGTTTTCGAAAGGAAGAAGAAGCGTGTTTTTGGAAGGAAGCGTTTTTTGGGGATTTTTTCCATA
46765472|F|0-49:C>T-49:C>T	TGCAGTTGTTTTAGCGAAGACGTAACCGACCTCCAATGCGGGCTTGTATCTTCTTGAGGACCATCTCCC
39722426|F|0-16:G>A-16:G>A	TGCAGCGGCAGCAGCAGCAGCCCAACAAAACAGGACGCCCAATGTCTTCCAACCATGGTCACACAAAAT
39718453|F|0-19:T>C-19:T>C	TGCAGTGGTATTAACCATGTCATCAACGTACACAAGTGTATGATTGAGAAGTTATGTGAATTGGGCACC

<table>
<tr><td>39715281|F|0-38:A>T-38:A>T</td><td>TGCAGCAACAATGGAGAATGGACGGAGTTTGTTCTGCAAATGCCTCCGACCCAATGGCTTCGAAAGACA</td></tr>
<tr><td>39723581|F|0-50:T>C-50:T>C</td><td>TGCAGGTCAAAGAAGCTCAAATTTCAAATTGACCTGGAAAAGAACTGTTGTCGCGGTTACTGGTAAGGG</td></tr>
<tr><td>39722491|F|0-44:G>A-44:G>A</td><td>TGCAGCAGTTGGGGGAGAGGCATATTGTTGGGTTTCGTCTCTGGGTGAGGTCAGCTGTGTTGATACATG</td></tr>
<tr><td>39728218|F|0-66:C>G-66:C>G</td><td>TGCAGATATTGCTTCAAGGGCTCTCACTGTCACAACTCACATCCATAAACAATTAGAACTATCTATCAC</td></tr>
<tr><td>39724188|F|0-51:C>A-51:C>A</td><td>TGCAGTGTTTTCTTTTCAACGCTATATATATTTTTTGTGCTAAAATACTTTCTTTCTTTCTTTCTTTCT</td></tr>
<tr><td>39714948|F|0-64:G>A-64:G>A</td><td>TGCAGATGAAGGTTATGCAATCTTCTTTCTTTTCATAATTTCAGTTTCATTTTTATTACTGACAGAGTA</td></tr>
<tr><td>39727901|F|0-5:T>C-5:T>C</td><td>TGCAGTTGTATCTGAATATATAACCAATAGAAAAGGTAGAGAAATCATATTGGCTGACACCTCAATATT</td></tr>
<tr><td>39718405|F|0-39:T>C-39:T>C</td><td>TGCAGTGGATTAGTTCGAGCTTTCGAACTATTTAGGTTGTTGTCCATCAAGAGATTGGTTACCTTGGGT</td></tr>
<tr><td>39713514|F|0-25:A>T-25:A>T</td><td>TGCAGAAACTGAAAAGCTAAAAAAAAAGGAGCCAATAAAATCTAAAATAACCTTTGTAGATGTATTTTT</td></tr>
<tr><td>39723040|F|0-60:A>T-60:A>T</td><td>TGCAGCTTCTTGGGATTCAATCTGCCAAGTAATTCAAATTAGTAGTCATTGAAATTATTCAATTCAATT</td></tr>
<tr><td>39718116|F|0-51:G>A-51:G>A</td><td>TGCAGTCCTTCATCGCCCGTTCAATTTCTTCGACAGGTCTGGGATTTCTTCGGCAGATCTGGGTTTTCT</td></tr>
<tr><td>39721531|F|0-47:A>T-47:A>T</td><td>TGCAGAATTTAGACGAAATCGAATTTGGGTTTATTCAGCGAAGGAAGACTTCGGCTTGCAGTTGCATCA</td></tr>
<tr><td>39721552|F|0-55:A>G-55:A>G</td><td>TGCAGACAACAAAAGGGGATTGGTTGATCAGTGTTTTAGAAGCAAAGGCTATGTGAGAAGGACTCAAAT</td></tr>
<tr><td>39716590|F|0-19:T>C-19:T>C</td><td>TGCAGCTTCTCCGGTGAAGTTCCTCGACGGCGCGACAATGGTGCAGCAGCTACAGTTAGCAAATCCGGC</td></tr>
<tr><td>39713646|F|0-8:A>G-8:A>G</td><td>TGCAGAACAAACCGAACTGTTTTAGATTTTGAAGTCAAAATTTTGTAAAGATTTTGTTGCAGATGAAGT</td></tr>
<tr><td>39713961|F|0-12:A>G-12:A>G</td><td>TGCAGAATAAAGAGAGGTTAGGTGTGGTTATGGTTGATCCATCGTGATCTCTGAATCTCTCATTCTCAT</td></tr>
<tr><td>39716655|F|0-40:C>T-40:C>T</td><td>TGCAGCTTTCATTAAAATTTACCTCCTACATGTTGATCCCCGTTGTAAAATATTATACTAAAATTGTGG</td></tr>
<tr><td>39718493|F|0-50:G>C-50:G>C</td><td>TGCAGTGTCTAGAATAATATTGCAAATACATCAGGCTACGATTTTTCTTCGCTGTATAGTCTGAACTAT</td></tr>
<tr><td>39715128|F|0-22:C>G-22:C>G</td><td>TGCAGATTGAATTTTAGGTGTACATATGTGAATTTTAGGTGCTGGAAACTGAAAGTTAGGTGCTGGAAA</td></tr>
<tr><td>39725048|F|0-30:C>T-30:C>T</td><td>TGCAGGAAACATTTATATTTCTTATTGACTCATTCATGATCATGAATTAC</td></tr>
<tr><td>39715903|F|0-14:A>G-14:A>G</td><td>TGCAGCCATGGGCAATGCATCTAAAGCAATCTTCTTAGCATTCTCATAGGTTTTCAATAATTTCAGATG</td></tr>
<tr><td>39720362|F|0-23:T>C-23:T>C</td><td>TGCAGGATGGATAGGTTAGGTCTTGTTTGATAACATTTTCATTTAC</td></tr>
<tr><td>39716221|F|0-23:G>T-23:G>T</td><td>TGCAGCGTTGAGCGCTGCTTTCAGTGGCCTTTCAATGCTGCCATCATTGGTTCTTAGTTGATTTCATGG</td></tr>
<tr><td>46765472|F|0-54:T>C-54:T>C</td><td>TGCAGTTGTTTTAGCGAAGACGTAACCGACCTCCAATGCGGGCTTGTATCTTCTTGAGGACCATCTCCC</td></tr>
<tr><td>39717747|F|0-58:A>G-58:A>G</td><td>TGCAGTAACGGGACAAAACCCACGAATCTGCGGCTATGAGGTGACGTCGGGACCTGCGACTGTGAGCGA</td></tr>
<tr><td>39717754|F|0-8:C>T-8:C>T</td><td>TGCAGTAACTGAAACATGTGACCAACAACAAAATTCATCGTGGGTATTTCTTCCTCACGATTTTCTTCG</td></tr>
<tr><td>39724186|F|0-17:C>G-17:C>G</td><td>TGCAGTGTTTCCCACGACGACGATGATGGCTTTGATTACGTGCTTCAAGGTTTGAATCACCATCTTGTG</td></tr>
<tr><td>39716594|F|0-21:C>T-21:C>T</td><td>TGCAGCTTCTCTGCCTATTCCCAACTCTGCTAGAGGCGGTGACATTCTTATGTGGCTACCTTCACAAAA</td></tr>
<tr><td>39726987|F|0-32:A>T-32:A>T</td><td>TGCAGACTGAAAATAGCATACTAAATCTGAAAAATGTTTTAGTTGTTCCTAATATTAC</td></tr>
</table>

<table>
<tr><td>39715458|F|0-34:T>G-34:T>G</td><td>TGCAGCACTAGTTCATTTGTTGACAGAGAATAAATTTTACAGTTACTAGAGTAATCTAGACAACAAGGA</td></tr>
<tr><td>39717129|F|0-32:T>C-32:T>C</td><td>TGCAGGCCCGTCCCAAATGAGATGGGACCCTGTGATACTTTTCAAAGTGAGGTCCTAAATATTTTATAC</td></tr>
<tr><td>39717806|F|0-17:C>T-17:C>T</td><td>TGCAGTAATTGGAGCATCTCATTCCTACGTGCAAATTTTTATCTTTCAGGTTTGGCATTCAGTTAGCAA</td></tr>
<tr><td>39725525|F|0-34:A>T-34:A>T</td><td>TGCAGAAACTTTTGCTCGAGTCGAATCTATAGTTATAGCGATGTGGCGAAATCTCTGTGGGAACTGAAG</td></tr>
<tr><td>39719111|F|0-19:C>T-19:C>T</td><td>TGCAGCAAGTGTTGATATGCTGATGACAATGATCCTTGTTTTGTTCCCTCCTGTATTCCATTTTATGAT</td></tr>
<tr><td>39726013|F|0-57:T>C-57:T>C</td><td>TGCAGCACAGCAGCAACTCAACTCAAATCATGACTTTATATTTACTTTTTCTTTTTGTAAAGGAAATTG</td></tr>
<tr><td>39722500|F|0-68:G>T-68:G>T</td><td>TGCAGCATAATTAGCTTAGATAACCTTGTTTCTTCTGGTGTAAAATTGCTTGTGGTGGTGAAATAGATG</td></tr>
<tr><td>39713824|F|0-29:G>T-29:G>T</td><td>TGCAGAAGATTTTAAATGAAACGTTTATAGATAAGCAGAAAGCTACGAAGAATTGAAGTGATACTGACA</td></tr>
<tr><td>39714019|F|0-42:C>T-42:C>T</td><td>TGCAGAATCTCATATAACTCAAGGAACCAAAAGCCAATAGTTCCATACTCCTACTATTCCAATGTGTAT</td></tr>
<tr><td>39724502|F|0-51:G>C-51:G>C</td><td>TGCAGAGTGTTGGGGTTCGTTTCCGGTGAGGTCTGAGCGTCGTATGTAACAGAGAACAAGAGATACAAG</td></tr>
<tr><td>39718406|F|0-59:C>G-59:C>G</td><td>TGCAGTGGATTGAAATGTGAGTCTTTGCTTTCATTTCTCACAATTTGGTCGTCACTTCACCTGGGCAAA</td></tr>
</table>
